# The people of the Cambridge Austin friars

**DOI:** 10.1080/00665983.2022.2090675

**Published:** 2022-09-26

**Authors:** Craig Cessford, Benjamin Neil

**Affiliations:** Cambridge Archaeological Unit, University of Cambridge, Cambridge, UK

## Abstract

The Austin friars in Cambridge was an important religious institution between the late thirteenth and mid-sixteenth centuries. Excavations have revealed well-dated and contextualised burials associated with the friary, as well as a range of material culture. The burials have been subject to a wide range of analyses including osteology, palaeopathology, stable isotopes, ancient DNA and geometric morphometrics. Significantly the distinction between clothed and shrouded burials allows members of the Augustinian order and the laity to be identified. This represents the best-understood published group of burials from an Austin friars in the British Isles and emphasises the importance of nuanced interpretation, as burial at friaries was a structured and multi-local phenomenon. These burials and other material can be interpreted in terms of both mendicant ideals and anti-fraternal criticisms.

## Introduction

**Figure 1.**
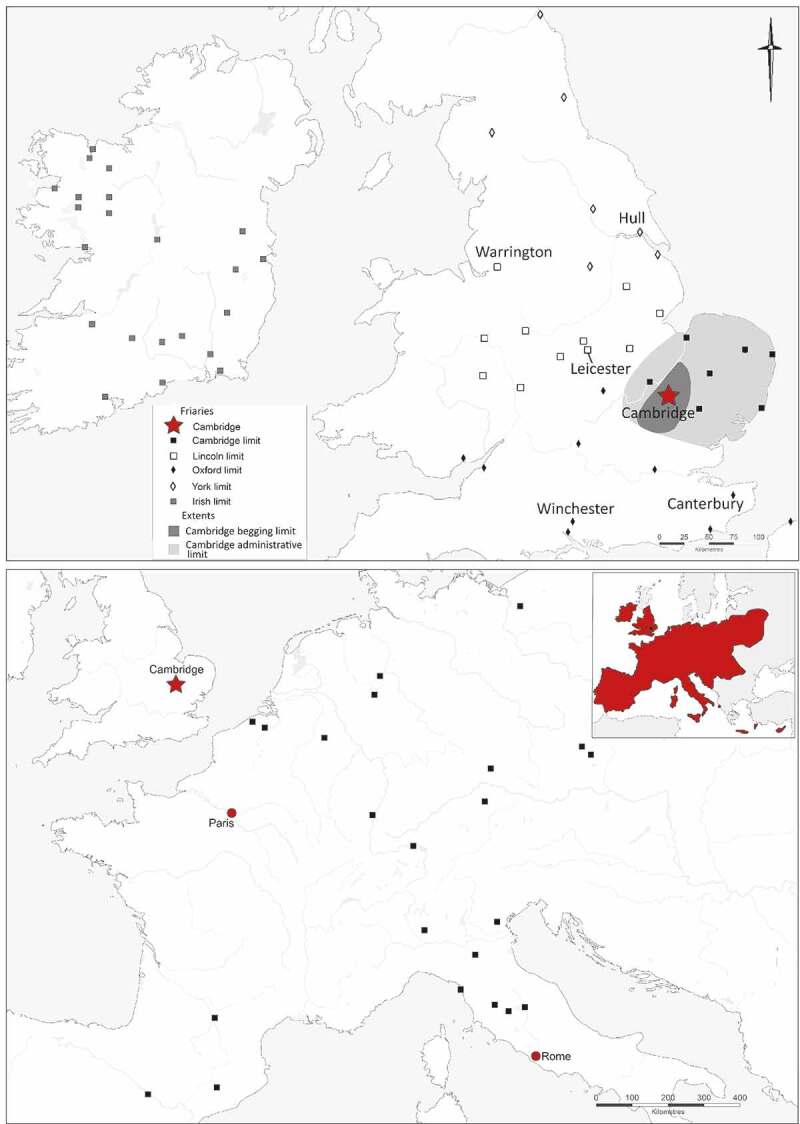
Map of the English Austin friars national province, showing the begging and administrative limits of the Cambridge friary (upper); map showing origins of Continental friars who studied at the Cambridge Austin friars, plus inset of the medieval extent of the order *c*. 1250–1550 (lower).

**Figure 2.**
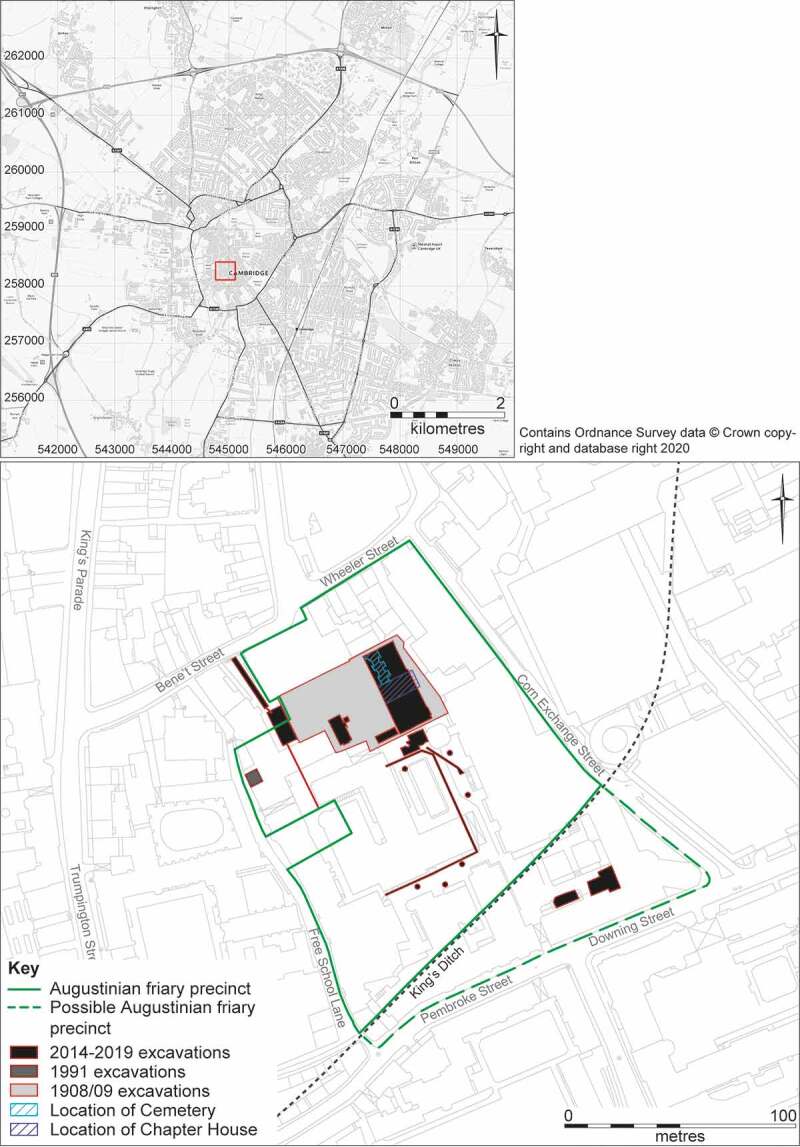
Location of the Austin friars within Cambridge (upper); archaeological excavations within the friary precinct (lower).

**Figure 3.**
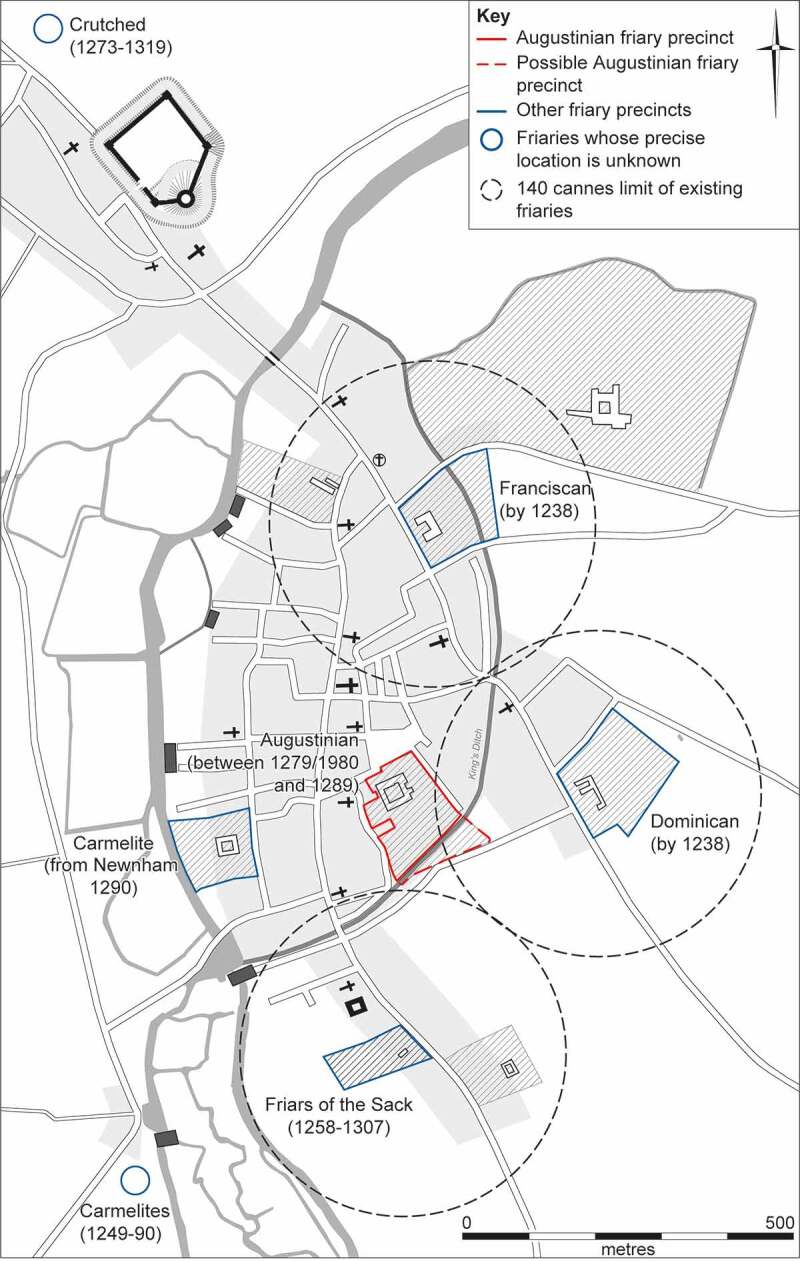
Reconstruction of Cambridge *c*. 1300, showing location of the Austin friars and other friaries. Base map by Vicki Herring for the After the Plague project, adapted by the CAU.

The Austin friars or Augustinian friary, Cambridge, was a mendicant religious establishment with a strong educational role, which was in existence for around 250 years between the 1280s and 1538 (Figures 1 to 3). Archaeological excavations in 2016–19 (Cessford 2017, 2020), plus earlier discoveries in 1908–9 (Cranage and Stokes 1921; Duckworth and Pocock 1910) (Figure 2), revealed a number of burials and sets of disturbed human remains, which form the main focus of this paper. Evidence for the friary architecture will be considered elsewhere (Cessford and Samuel in prep).

The archaeology of the people of the friary is informed by 38 burials excavated in 2016–17, supplemented by the remains of *c*. 47 skeletons recovered in 1908–9. There is also a range of material from the 2016–19 excavations, augmented by finds from the 1908–9 investigations, some of which can be closely linked to individuals in the past. Some items were recovered in direct association with skeletal remains, while others were probably closely associated with particular, albeit unknown, individuals in the past. There are also individuals mentioned in surviving medieval written sources, which record that they spent time at the friary while alive or were buried there. Individuals associated with the friary are broadly defined, encompassing anyone with a meaningful identifiable connection to it. This includes members of the Augustinian order who spent significant periods of their life there, other members of the order with briefer connections and various members of the laity. The skeletal, material culture and textual evidence are rarely directly related, except in the case of items recovered in direct association with skeletal remains, but are broadly complimentary.

The defining characteristics of the medieval mendicant movement were a commitment to poverty and begging, the mobility of its members combined with the international nature of the order and the urban locations of most friaries. These characteristics and the mendicant ideals that underpinned a friars’ life provide an important reference framework for interpreting the archaeological remains from the Cambridge Austin friars. This framework can be contrasted with medieval authors who disapproved of the mendicants, most commonly referred to as anti-fraternal criticisms.

The aim of this paper is twofold, to synthetically address several pertinent issues while at the same time providing enough general detail to permit others to make use of the results to address other questions. At one level this is simply the presentation of the results of the analysis of a moderately sized sample of burials, albeit one that is particularly strong due to the relatively close dating of many of the burials, the range of analytical techniques employed including stable isotopes, ancient DNA and geometric morphometrics and the distinction between clothed and shrouded burials. Underpinning much of the discussion are the related issues that the spatial limitations of the investigations mean that the skeletons discussed are likely to represent a biased sample and that both members of the Augustinian order and the laity were buried at the Cambridge Austin friars. This is true of all archaeological investigations of friaries, but has generally not received the attention that it deserves.

## The Cambridge Austin friars

The archaeological investigations in 1908–9 (Cranage and Stokes 1921; Duckworth and Pocock 1910) and 2016–19 (Cessford 2017, 2020) both focussed on an area towards the northern end of the friary precinct. This revealed the southern edge of the friary church, plus a cemetery located to the south of the church. At a later date the friary cloister was constructed to the south of the church, the eastern range of which overlay the earlier cemetery. The cloister walk and garth plus all three ranges of cloister buildings, including the chapter house, have been investigated. Some buildings to the south of the cloister and an open area to the west of the cloister were also identified.

The documentary evidence relating to the Cambridge Austin friars was considered by Ellis and Salzman (1948, 287–90) and published in detail by Roth as part of his survey of the English Austin friars (1966, vol. I, 250–3), with archaeological and documentary evidence summarised by O’Sullivan (2013, 75–7). Most of the documentary evidence relating directly to Cambridge used in this paper derives from these sources, with additional material from research by Rosemary Horrox and Nick Holder. On the Austin friars in England the main sources are Roth (1966), plus more recent scholarship by Gutiérrez (1984), Andrews (2006, 69–172) and O’Sullivan (2013).

The Hermit Friars of St Augustine (*Ordo Eremitorum Sancti Augustini*), commonly referred to as the Augustinian or Austin friars, were created by the Great Union of 1256, although some groups that formed this were present in England as early as 1249. The Cambridge friary was not in existence at the time of the Hundred Rolls in 1279/80 (Casson et al. 2020a, 2020b) and the earliest written reference to the friary dates from October 1289, when the Cambridge Austin friars received a royal pittance of 240d to feed the friars for three days at 4d per day, equating to 20 friars (Farris 2013, table 68; Roth 1966 vol. II, 51 no. 90).

**Figure 4.**
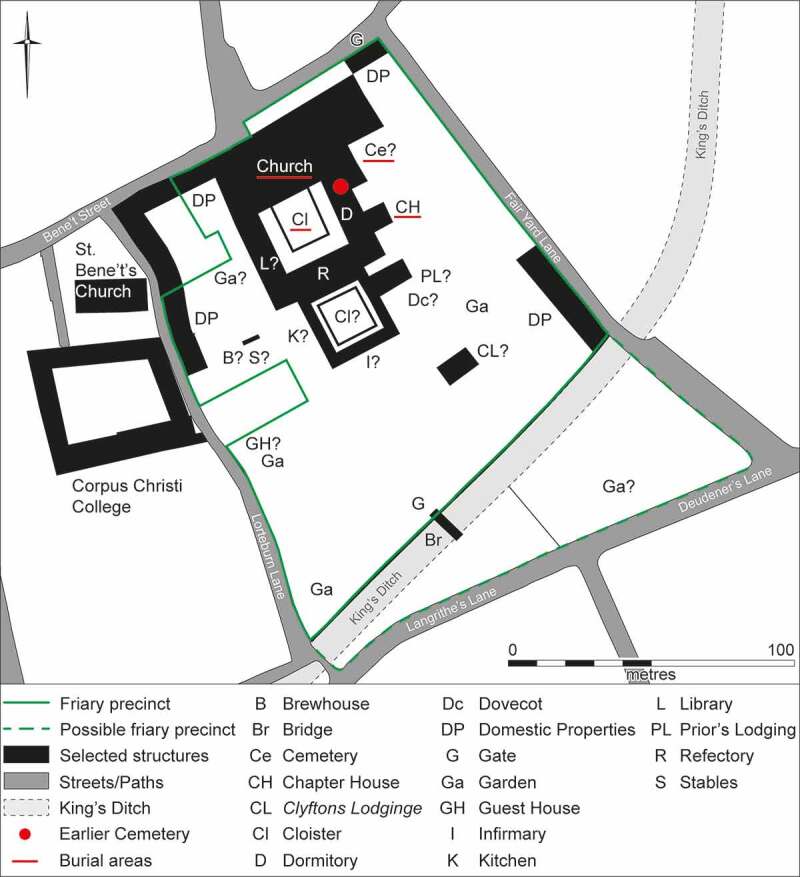
Reconstruction of the Cambridge Austin friars in the fifteenth century, showing location of investigated burial areas.

The site occupied by the Cambridge Austin friars was bounded by Bene’t Street/Wheeler Street to the north (medieval *Vicus St Benedicti*), Free School Lane to the west (medieval *Lorteburnestrata*), Corn Exchange Street to the east (medieval *le Feireyerdlane*), and Pembroke Street to the south (medieval *Langritheslane* or *Deudeneris lane*) (Figure 4). The friary acquired most, but not necessarily all, of the street block in a long-term process that took until the 1370s (Cessford and Samuel in prep).

In November 1290, the friary agreed to pay 4s per annum to Barnwell Priory who held the advowson for St Edward’s parish, as compensation for lost revenue (Clark 1907, 212–14, no. 21: Roth 1966 vol. II, 54–5 no. 97). This lost revenue would typically be from tithes and burial fees, implying that the friary had a functioning church and cemetery, although the friars’ servants still had to attend the parish church. The church was probably still being finished in 1319–24, when a rent due to the Crown was cancelled (CPR 1317–1321, 396; CPR 1321–1324, 399). In 1302 the friary was granted rights of burial, preaching and hearing confessions for those who were not members of the community (Empoli 1628, 50–2).

In 1297 there were 36 Austin friars at Cambridge(Farris 2013, table 69: Roth 1966 vol. II, 70 no. 126) and by 1326 their number had risen to 70 (Roth 1966 vol. I, 469). Chapters of the Augustinian national province were held at Cambridge in 1316, 1322 and 1323 (Roth 1966 vol. I, 485–6). Of the four orders of friars that survived after the early fourteenth century, in Cambridge the Austin friars were numerically smaller than the Franciscans and Dominicans and broadly equal to the Carmelites (Zutschi 2002). Although undoubtedly adversely impacted in terms of numbers by the Black Death and other events in the fourteenth century, the Cambridge friary remained important throughout its existence, with evidence for friars from Continental Europe studying and teaching there (see Supplementary Material: Non-English friars with links to the Cambridge Austin friars). The friary church was rebuilt or remodelled in the mid-fourteenth century and a cloister was constructed in the mid–late fourteenth century, while a second cloister may have been added in the fifteenth century (Cessford and Samuel in prep).

The friary was closely integrated into both the town, as evidenced by several late fifteenth–early sixteenth wills (Holder in prep) and other sources, and the University, where the friars mainly studied Theology and the friary church occasionally hosted University events. There was still a vibrant intellectual community in the late fifteenth and early sixteenth centuries; in the early sixteenth century several Cambridge Austin friars were important Evangelical and early Protestant figures (Ellis and Salzman 1948, 289) although there is also considerable evidence for countervailing traditional activities, such as indulgences and a *Scala Coeli* altar (Laferrière 2017, 127–31). When the friary was dissolved in 1538 only the master and three friars signed the surrender (Ellis and Salzman 1948, 290; Roth 1966 vol. II, 472, no. 1127), but as several friars had received dispensations to act as secular priests between 1536 and 1538 (Roth 1966 vol. II, 514–17, no. 1177) there were at least nine friars in the mid-1530s.

Friars were not restricted to the friary where they were based and surrounding the Cambridge friary was a region or limit, where designated friars known as *limiters* could preach, hear confessions and beg for alms (Roth 1966 vol I, 225–6). The boundaries of limits were broadly equidistant between friaries of the order and although they are not known in detail they can be estimated. In 1389 the Cambridge and Clare Austin friars were in dispute about whose begging limit the village of Hatfield Regis, Essex, which was 44 km and 36 km distant respectively, fell within. Hatfield Regis was granted to Clare, but Cambridge gained the smaller nearby villages of Chawreth (part of Broxted; 35 km from Cambridge and 27 km from Clare) and Takeley (39 km from Cambridge and 32 km from Clare) (Harper-Bill 1991, 83, no. 138). The English national province of the Austin friars was divided into four administrative limits. By 1387 the Cambridge friary was the head of one administrative limit, broadly corresponding to East Anglia. This comprised friaries at Clare (1248/9), Huntingdon (1258), Gorleston/Little Yarmouth, (1267), Norwich (1277–89), King’s Lynn (by 1296) and Orford (1295–9) (Roth 1966 vol. I, 222; vol. II, 233–4 no. 590), to which Thetford was added by 1389 (Roth 1966 vol. I, 342–6).

The English national province included friaries covering much, but not all, of England and Ireland, plus single friaries at Newport in Wales and Calais in France. The wider order covered most of Western Christendom, headed by a Prior General in Rome, and at its height there were 42 national provinces. There is considerable textual evidence for contacts and movement between England and the Low Countries, Germany and northern Italy, with more sporadic evidence for contacts with France, Iberia, the Eastern Baltic and pilgrimages to Jerusalem (Roth 1966, vol. II: see also Supplementary Material: Non-English friars with links to the Cambridge Austin friars).

The Cambridge friars followed the 1290 rule of the Augustinian mendicant order, known as the Ratisbon *Constitutions* (Cendoya 1966; Ponesse 2020), which incorporated the thirteenth-century mendicant practices of other orders of friars. These rules and practices were, in turn, largely based on those of the Augustinian canons of the twelfth and thirteenth centuries, who had developed a more flexible interpretation of monasticism. There was, however, no formal link between the Austin friars and the Augustinian canons.

### Friars and laity

Individuals buried at the friary include both members of Austin friars and lay individuals. Novices could be recruited from the age of 14 prior to the Black Death and 11 or even 10 afterwards (Roth 1966 vol. I, 136–7). Candidates were supposed to join their local friary (Roth 1966 vol. I, 138), defined as the one that their birthplace or where they spent their childhood fell within the begging limit of. Most novices were local to the town a friary was based in and almost all came from the same county. The majority were from reasonably prosperous merchant or gentry families, but not from the nobility. After a year’s novitiate individuals could join the order proper. This was followed by various educational stages culminating in a year spent at the *studium generale ordinis* of Cambridge or Oxford, although it is possible that friars from the limits of Lincoln and York studied there instead. For those that had become novices at 14 they would have been aged 19–20 when attending a *studium generale ordinis* such as Cambridge.

Some friars then continued on to academic degrees at a *studium generale*. The initial stage was effectively as a student, which usually lasted for three years but could take up to five. Each of the five English and Irish limits could send four students to both Oxford and Cambridge and those friaries themselves could add two more (Roth 1966 vol. I, 153). Each foreign province, whose numbers varied between 24 and 42 over time, could send one student and the head of the order could add three more (Roth 1966 vol. I, 153). In practice these quotas were probably never fully filled and numbers of 25–30 students at the Cambridge *studium generale* are likely (Roth 1966 vol. I, 153). Most came from England, although individuals from several Continental friaries are recorded as coming to Cambridge. These students would generally be aged in their early 20s. In 1287 the general chapter ruled that no friar was to be promoted to the priesthood under the age of 24, soon after a friar would typically have finished their initial degree. After this stage a few friars continued through a series of academic stages, that could last up to 14 years. By the final stage of *magister*, the Austin friars restricted the number preparing for the degree at any time to four: two from England, one from Italy and one from another foreign province. When not required by foreign provinces, these places were re-allocated to English friars.

In addition to its specialised educational role, the Cambridge friary also functioned as a ‘normal’ friary. This means there would be the typical presence of adult friars, including a prior, subprior, master of novices, procurator and sacristan, plus probably an almoner, hospitaller, infirmarian, librarian and porter. In 1340 the Cambridge friary had seven *limiters* (Ellis and Salzman 1948, 287: Roth 1966 vol II, 140–1 no. 340) (see above: the Cambridge Austin friars), including the prior and subprior who appointed surrogates. In 1340–5 the friary had 12 *penitentiaries*, individuals permitted to administer penance, which included the seven *limiters*, who were largely at least Bachelors of Divinity (Ellis and Salzman 1948, 287: Roth 1966 vol II, 140–1 no. 340, 174 no. 397).

In addition to the friars there were a few non-clerical lay brethren, who did not perform the full variety of liturgical tasks. The friary also employed a number of lay servants, these probably included laundresses, gardeners etc. and individuals collecting stone and timber in Cambridgeshire and Huntingdonshire (1356) and John Cook, servant and cook (1386), are mentioned (Roth 1966 vol. II, 181 no. 428, 229 no. 575). Within the friary there was a well-defined hierarchy, based principally on seniority and learning.

There were no formal criteria governing lay burial in friaries, which was largely have been based on the wishes of the deceased, the ability to pay and geographical proximity. Numerous lay individuals had close relationships to the friary and several groups can be identified from texts. These include those who gifted property to the friary, such as William de Novacurt whose grant of a messuage fronting onto *Vicus St Benedicti* was confirmed by mortmain licence in 1305 on condition that an anniversary mass was to be said for William and his daughter Margaret on 3rd August (Lincolnshire Archives, Holywell 89/1). In the Hundred Rolls of 1279/80 William de Novacurt was linked to a considerable number of properties in Cambridge and land in the town fields and the family had held significant property interests for several generations (Casson et al. 2020a). Some individuals were granted letters of confraternity, enabling them to participate in the collective spiritual merit of the order. This included Robert Norys and his wife Johanna in 1440 (Roth 1966 vol. II, 324 no. 793), other documents suggest that Norys had been involved in property transactions in Cambridge since 1415 and was a barber. There were also individuals that bequeathed gifts in their wills such as Margaret Phillis, that leased and lived in properties at the friary such as William Sengeorge and those that regularly worshiped at the friary church such as Katherine Bailey (see Supplementary Material Textual individuals with links to the Cambridge Austin friars for detail of these individuals). Some lay people who had a close relationship with the friary in life may also have chosen to continue that relationship after death, requesting burial at the friary and/or paying for friars to perform post-mortem masses to speed their soul through Purgatory.

Based on the skeletal evidence, supplemented by various texts, those buried at the Cambridge Austin friars are a relatively heterogenous group; including friars and novices linked to its operation as a normal friary, members of the order studying there due to its specialised educational role, lay brethren, servants and a range of members of the laity. Apart from the laity, and perhaps specialised servants such as a laundresses, these individuals would be exclusively male and initially aged 14 or over although this later fell to 10, and there were likely to be many individuals aged 19–23. A significant proportion of the friars were of local origin, although those studying at Cambridge were drawn from a much wider catchment, as were most if not all the laity linked to the friary. Both friars and laity associated with the friary were mainly drawn from moderately affluent social groups, although less wealthy laypeople might have paid small sums for burial in the churchyard and not left documentary traces.

### The spatial hierarchy of burial

Burial could take place at several locations within friaries and other monastic institutions of this period. This included the church, the cloister garth and walk, the chapter house, any ancillary chapels and cemeteries (Gilchrist and Sloane 2005a, 56–60). By the Late Medieval period the church was the most prestigious location for burial, with a strong spatial hierarchy within the building. The most prestigious location was the chancel, where important lay patrons and senior friars were buried. Friars also appear to have been buried in the transept, while wealthy members of the laity were buried in the nave.

The cloister, an open courtyard space enclosed by the church and three ranges of buildings, was also used for burials. At most monastic institutions burial in the central open cloister garth was a relatively uncommon, but more frequent in the covered walkway around it. Evidence indicates that both friars and laity were buried in the cloisters. The chapter house was one of the buildings around the cloister and was where the monastic brethren met on a daily basis. Burial in the chapter houses had begun by the ninth century and was the norm by the eleventh century. Burial was initially limited to the heads of monastic institutions and this continued until the thirteenth and fourteenth centuries at different institutions (Gilchrist and Sloane 2005a, 59). These burials were often quite regularly and sequentially arranged and would be osteologically relatively homogenous groups; typically all of one sex (usually male, but female at nunneries), generally mature or older individuals and in most cases members of a religious order for a prolonged period of decades. In the thirteenth century, when friaries began to be established in England, the practice of chapter house burial was still active but beginning to decline with the church becoming the favoured burial location for heads of institutions. While chapter house burials continued this was a very different practice, which was not regularly or sequentially arranged and involved heterogenous groups of individuals (see below: the Chapter House). Burial in cloisters and chapter houses was less frequent than in the church and less prestigious.

There is no evidence that the Cambridge Austin friars possessed any separate ancillary chapels. Most monastic establishments also had one or more external cemeteries where the majority of burials took place. Cemeteries were in effect the default and lowest status burial location for monastic institutions, and were where the majority of friars and members of the laity were interred.

### The friary burial population

There is no way to determine precisely how many individuals were interred at the Cambridge Austin friars *c*. 1290–1538, but it is possible to produce a broad estimate. While there are numerous caveats, assuming a mean population of 20–40 friars and a mortality rate of 25–50 per thousand each year (based on figures from other English religious institutions, summarised in Smith (2018), then *c*. 125–500 friars would have been interred at the friary over the course of its existence. Estimating the number of lay individuals buried at the friary is more problematic. These span a 236 year period (1302–1538) and the mean population of the town over this period was perhaps *c*. 3500 (Cessford in prep. a). With a mortality rate of 25–50 per thousand each year and assuming (based on late fifteenth-early sixteenth century wills) that around 1.5% of the town population was buried in the friary (Holder in prep) this suggests perhaps 300 to 600 lay burials. The overall estimate for burials at the Cambridge Austin friars is therefore *c*. 425–1100. This compares to an estimate of over 500 burials for the more extensively investigated but unpublished Kingston-Upon-Hull Austin friars (Evans 2014, 287).

## The skeletons

As less than a hundred burials and sets of disarticulated human remains have been recovered from the Cambridge Austin friars systematic and detailed comparisons with other skeletal assemblages have not been undertaken. Locally the two most important comparative assemblages from Cambridge are from the parish church of All Saints by the Castle (cemetery dated to *c*. 940/1100–1365/6 with *c*. 213 burials excavated on various occasions: Cessford, Craddock, and Gregory in prep) and the Hospital of St. John the Evangelist (burials dated to *c*. 1204/14–1467/1511 with 400 burials excavated: Cessford 2015, in prep. b). The skeletons from both these sites and the Cambridge Austin friars have been re-analysed by the After the Plague project, some of this work is reported on in this paper with the detailed results to appear in a monograph (Robb in prep. a). The largest group of excavated burials from another English Austin friars at Kingston-Upon-Hull is unpublished (Evans in prep), while smaller groups have been published (e.g. Leicester: Mellor and Pearce 1981) direct comparisons are problematic as the areas of the friaries investigated are not identical to those investigated at Cambridge.

The skeletons from the Cambridge Austin friars are only a proportion of the individuals interred there *c*. 1290–1538 (Table 1). The 88 individuals that are completely or partially represent 8–21% of the estimated 425–1100 burials (see above: the friary burial population) at the Cambridge Austin friars. The skeletons are heavily biased, with certain burial locations well represented and other largely or completely absent.

**Table 1.** Skeletons from the various areas of Cambridge Austin friars and types of analyses undertaken. Total for the early cemetery includes two entirely disarticulated individuals.

Burial area	Date	Total skeletons excavated	Estimated % of original burials	Adult	Adult male	Adult female	Non-adult	Dietary isotopes (δ13C and/or δ15N)	Mobility isotopes (δ18O & δ13C and/or Sr87/Sr86)	Cross-sectional geometry	aDNA	Radiocarbon determinations
Early cemetery	1290–1400/20	34	70–100	29	24	–	5	24	17	17	23	3
Chapter house	1330/50–1538	6	80–100	4	3	1	2	6	4	4	6	1
Cloister	1330/50–1538	47	80–100	47	41	4	1	–	–	–	–	–
Church	1290–1538	1	<1	1	1	–	–	–	–	–	–	–
Late cemetery	1400/20–1538	–	0	–	–	–	–	–	–	–	–	–
Total	1290–1538	88	8–21	81	67	5	7	30	21	21	29	4

### Burial locations

Human remains from inhumations, a small charnel deposit, disarticulated bone from graves and the cemetery soil were recovered from four areas: the church, the cloister garth/walk, the chapter house and a cemetery (Figure 4). Dating of these is based on a combination of texts, stratigraphic sequences, architectural styles, artefactual typology particularly of objects directly associated with skeletons and items in grave fills, the identification of ancient DNA from strains of *Yersinia Pestis* and Bayesian modelling of radiocarbon determinations.

Friaries, like other medieval monastic houses, typically had several burial spaces. These spaces were quite closely controlled by the friars, with discrete types and ‘grades’ of burial associated with each locale. As a result, the skeletons are likely to represent a biased sample. They represent most or all the individuals interred in some locations, while other areas where burials took place are not represented, as they have either not been investigated or were destroyed with no record. This is particularly significant, because where individuals were buried in a friary was based on several criteria. Although distinctions between each burial area were not absolute, in general different religio-socio-economic groups were more or less likely to be buried in different parts of the friary. The human remains are therefore not entirely representative of the original friary burial population, a caveat that also applies to all other investigated British friary sites. As different areas have been excavated at different friaries, straightforward comparisons are ill advised. It is also important to note that although the cemetery and chapter house (plus cloister garth/walk) skeletons from Cambridge represent different phases of the friary comparing them is not straightforward, as they will contain different elements of the friary population. Although not linked to burial location, other biases relate to survival, truncation and other human and taphonomic agencies have affected the skeletal assemblage. This includes the deliberate exhumation of one skeleton during the medieval period and the poorer survival of skeletons in shallower burials; indeed, it is possible that some skeletons have been entirely removed by later construction.

#### The church

Various discoveries from 1746 onwards indicate that burials have been encountered during building works on Bene’t Street (Cranage and Stokes 1921, 57–8). These relate to burials in the friary church, which has been either completely or almost completely destroyed by basements of later buildings. Only a single surviving cranium, recorded as coming from Mortlock’s Bank or House in the late nineteenth–early twentieth century, can confidently be identified as coming from the church. The church probably contained hundreds of burials, as the church of the Kingston upon Hull Austin friars contained 198 skeletons (Gilchrist and Sloane 2005b). As the church was the most prestigious location for burial in the friary, it is likely that all the priors plus other senior members of the order were interred there. The church was also the burial location most favoured by wealthy lay individuals. While skeletal evidence is lacking for individuals buried in the friary church there are documentary sources that shed light on some of the individuals who were probably or definitely buried there (see below: textual individuals).

#### The cloister garth and walk

Located south of the church were the cloisters, arranged around a central open area or garth that had a walkway around it. Although records are relatively sparse, it appears that burials recovered in 1908–9 occurred in both the cloister garth and walk, with the majority probably coming from the walk, and dating to *c*. 1330/50–1538 (Figure 7). A minimum of 48 individuals (55% of the individuals from the friary studied) were buried here, which probably represents most of the original total. This compares to 52 and 12 from the comparable areas of the Kingston upon Hull and Leicester Austin friars (Gilchrist and Sloane 2005b). Although all skeletal elements were initially recovered, and long bone measurements were reported on (Duckworth and Pocock 1910), only cranial elements could be identified in the Duckworth Collection, University of Cambridge.

**Figure 5.**
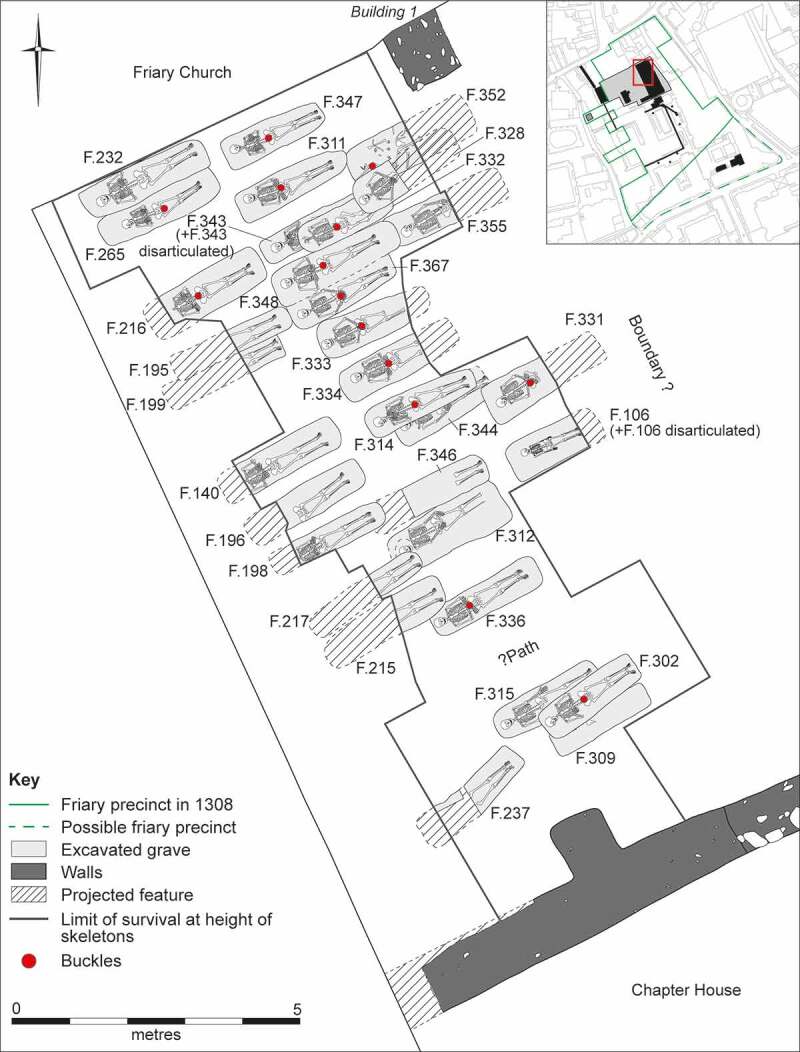
Plan of the cemetery associated with the early phase of the Cambridge Austin friars *c*. 1290–1400/20.

#### The chapter house

On the eastern side of the cloister was the chapter house. Excavations in 2016–17 revealed six burials (7% of the individuals from the friary studied), which represents either all or most of the individuals buried (Figures 7–9). Although this is comparable to the numbers of 6–9 found in other friary chapter houses (Leicester Austin friars six, Carmarthen Franciscans and Ipswich Dominicans seven, Oxford Dominicans eight, Winchester Austin friars nine: Gilchrist and Sloane 2005a, 67–8, 2005b), truncation at most other sites suggests that the original numbers there were higher.

**Figure 6.**
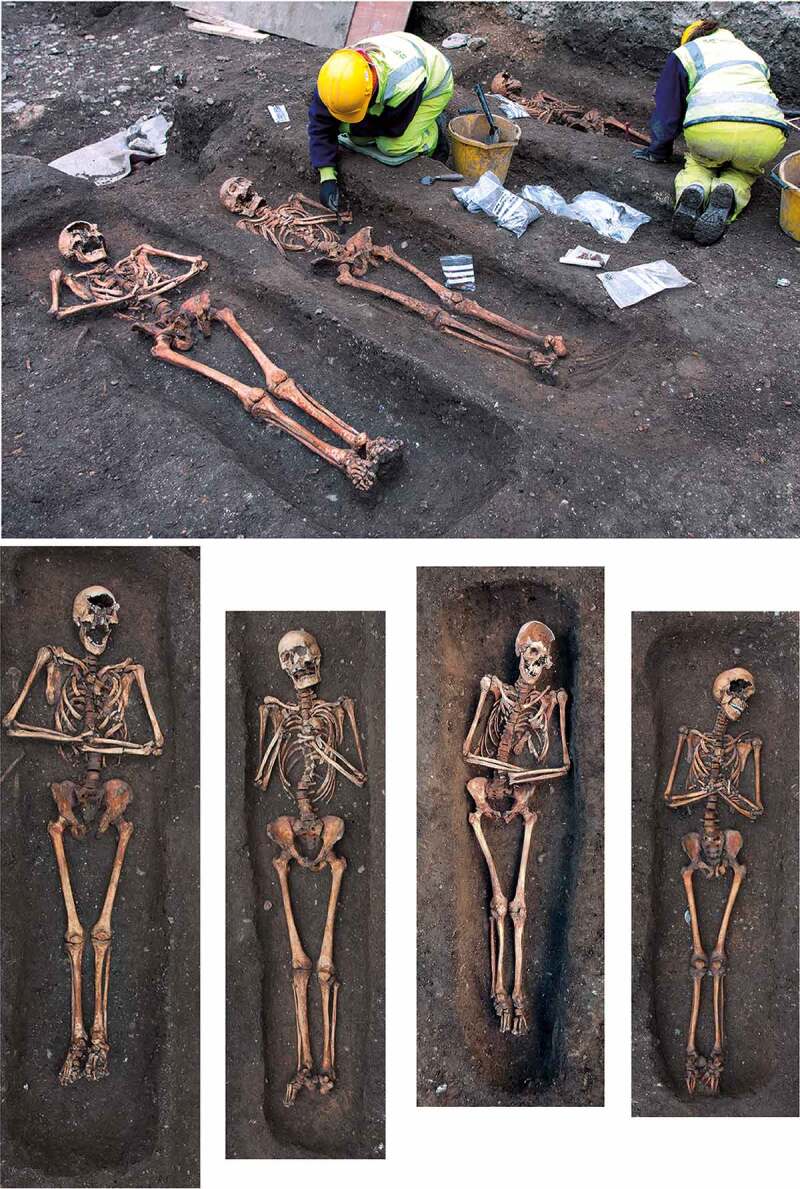
View of the Cambridge Austin friars cemetery during excavation, facing northwest, with row of burials F.348, F.343, F.311 (skeleton not visible) and F.347 from left to right (upper); individual views of the same four burials arranged to mimic their physical layout (lower).

**Figure 7.**
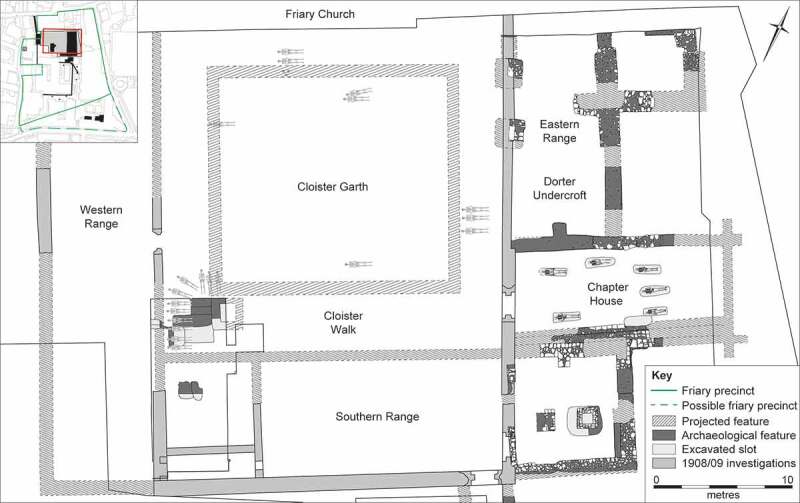
Plan of the Cambridge Austin friars cloisters of *c.* 1330/50–1538, showing burials in the cloister walk/garth and chapter house.

**Figure 8.**
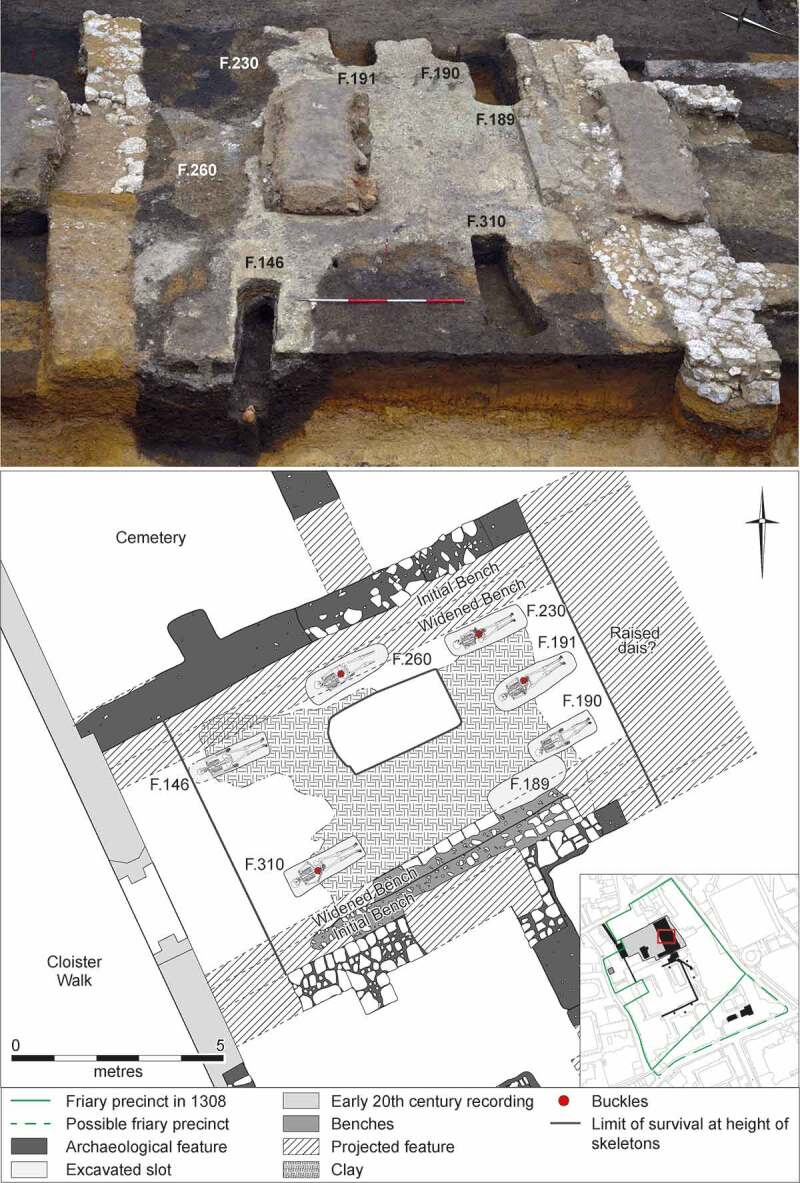
View of the Cambridge Austin friars chapter house, facing east-northeast (upper); plan of chapter house (lower).

The chapter house went through one phase of significant remodelling, when the foundations that supported benches along the walls of the chapter house were widened Only one of the chapter house burials took place in the first phase *c*. 1330/50–1425/75, with the other five dating to after the remodelling *c*. 1425/75–1538. There was also an additional grave-shaped cut that contained no human remains (F.189: Figure 8). The location of the grave-shaped cut and stratigraphic evidence indicates that the grave was originally dug during the earlier phase, when the benches were relatively narrow, and was re-opened some time after the initial interment, after the benches had been widened. As the grave was re-opened it probably represents a burial that was subsequently dug up and ‘translated’ elsewhere (Gilchrist and Sloane 2005a, 197–9).

How to conceptualise burial in the Cambridge Austin friars chapter house is not entirely clear, but it is perhaps best to conceive of chapter house burial as exclusive but not elite. Unlike in earlier monastic institutions the burials do not relate exclusively to senior friars (see above: the spatial hierarchy of burial), as is clear from the ages and sex of the individuals. Given the low number of burials, it is particularly notable that the chapter house burials are a remarkably heterogenous group. Clothed (Austin friars) and unclothed (laity) individuals, men and women and a wide range of ages at death from juveniles to mature adults who were probably aged over 60 are all represented. The reason why any particular individual was buried in the chapter house is unknowable, but it is likely that they were held in high esteem. A range of factors, such as long term suffering and incidents of violence may have led to individuals gaining a reputation for sanctity, while dying from plague was not a barrier.

Of particular note is the burial after *c*. 1500 of a woman in the chapter house. Women were not permitted in friary cloisters except on a few special occasions (Roth 1966 vol. I, 234–5) and their presence in the chapter house during life would have been an extremely rare occurrence. Textual evidence indicates that most lay individuals buried in friary chapter houses were male, but there is evidence for the burial of a few women from knightly families. Sir Hugh Despencer had his children Philip and Isabel buried at the London Austin friars chapter house in the early fourteenth century (Steer 2013, 262–3). At around the same time Peter of Huntingfield in Suffolk was buried at the London Franciscan friary chapter house and his widow Ismay was later interred near him (Kingsford 1915, 133). These women appear to have been interred in chapter houses mainly due to familial relationships, but there are no other lay burials associated with the woman buried at the Cambridge Austin friars, so she appears to have been buried there in her own right. Archaeological evidence indicate that the majority of individuals buried in friary chapter houses were either adult males or non-adults aged under 18 (Gilchrist and Sloane 2005a, 67–8:2005b: O’Sullivan 2013). Previous archaeological identifications of adult females are not certain: an individual was identified as female at the Carmarthen Franciscan friary (James 1997, 138) but the ‘evidence was not totally conclusive’ (Wilkinson 2001, 7) and another skeleton from the Winchester Austin friars is only ‘probably’ female (O’Sullivan 2013, 341).

#### Cemeteries

A cemetery of *c*. 1290–1400/20 located south of the friary church was investigated in 2016–17 (Figures 5, 6). A total of 31 articulated burials and a single heavily disturbed burial were investigated, while disarticulated material indicates the existence of at least two individuals whose graves had been entirely removed. It is possible that later truncation has entirely removed some skeletons, but the 34 burials and disarticulated human remains (representing 39% of the individuals from the friary studied) must represent either the overwhelming majority or all the individuals interred in the cemetery.

**Figure 9.**
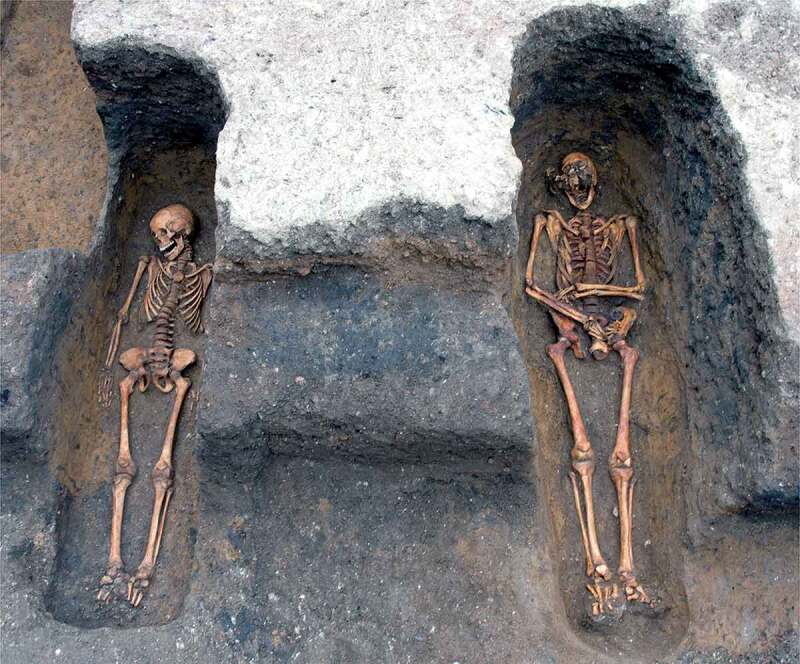
View of the Cambridge Austin friars chapter house, facing west-southwest, showing adjacent burials F.190 and F.191 cut through floor makeup, plus part of grave shaped cut F.189 upper left.

There was also a later cemetery of *c.* 1400/20–1538. The approximate location of this cemetery is known from written sources, it was moved eastwards when the cloister was completed in the early fifteenth century and was probably largely or completely destroyed with no record during the construction of the Cambridge Corn Exchange in 1874–5. While skeletal evidence is lacking for individuals buried in this cemetery there are documentary sources that shed light on some of the lay individuals who were buried there (see below: textual individuals).

### Burial practice

Most of the excavated burials were aligned west-southwest to east-northeast, probably reflecting the orientation of the friary church itself arranged parallel to St Bene’t Street. Extended and supine, the inhumations were interred in earth-cut graves with the head to the west-southwest. The 1908–9 plan (Duckworth and Pocock 1910, facing p.38) shows an unusually arranged group of eleven skeletons: six are aligned west–east, two northwest–southeast and three north–south. Re-investigation of this area in 2019 indicates that the skeletons were arranged around a feature with a substantial square or rectangular, located in the southwestern corner of the cloister walk, whose function is unknown. Most of the grave cuts were straight-sided rectangles with rounded corners, although three were distinctly trapezoidal (F.106, F.310, F.331). In the cemetery the grave cuts were 1.74–2.3 m long (mean 2.1 m), by 0.42–0.88 m wide (mean 0.58 m) and their original depth is estimated as *c*. 0.5–1.2 m (mean *c*. 0.8 m). In the chapter house the graves were rather shorter (1.66–2.2 m long, mean 1.89 m), roughly the same width at (0.45–0.95 m wide, mean 0.69 m) and noticeably deeper, with original depths estimated as 1.0–1.4 m (mean *c*. 1.2 m).

**Figure 10.**
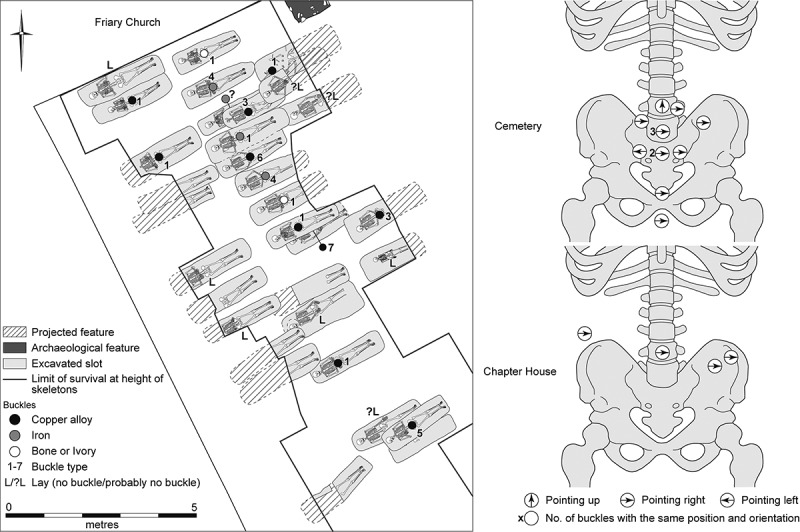
Plan of the cemetery of the Cambridge Austin friars *c*. 1290–1400/20, showing skeletons with buckles and those that definitely lack them (left); plus, locations and orientation of buckles on skeletons from the cemetery and chapter house (right).

**Figure 11.**
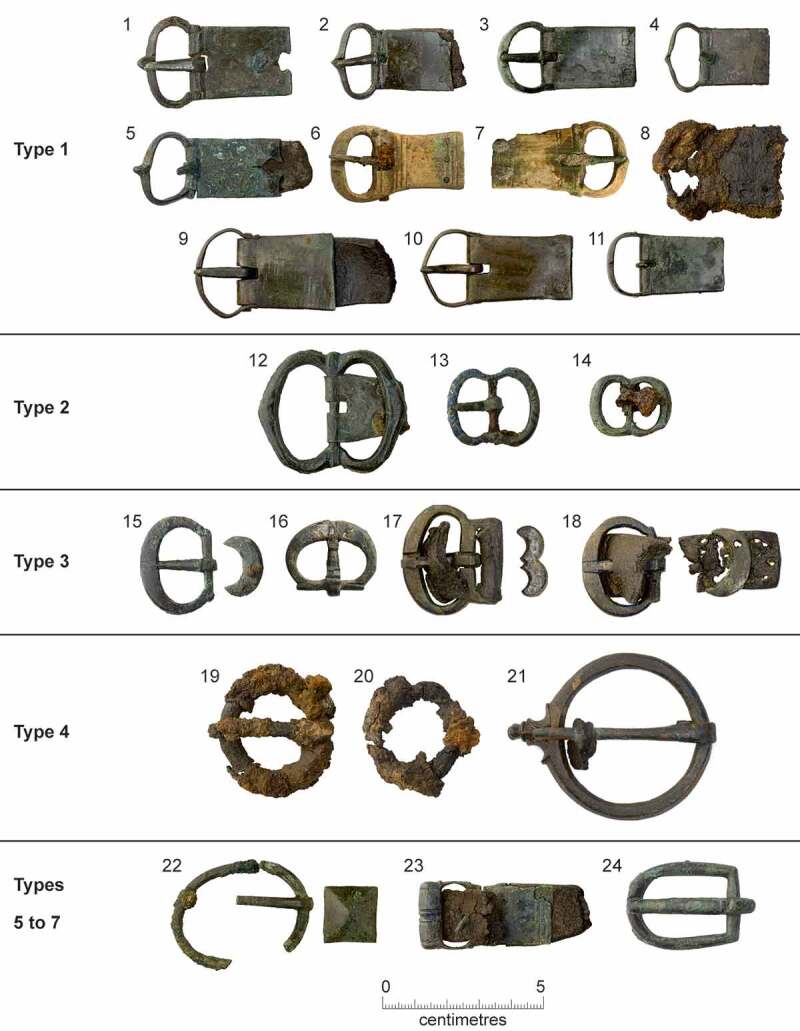
Girdle buckles from the early cemetery and chapter house of the Cambridge Austin friars associated with skeletons, plus buckles recovered in 1908–9 that were also probably associated with skeletons. Copper alloy unless otherwise stated. 1) type 1, <2053> [1430] F.216; 2) type 1, burial <2062> [1638] F.265; 3) type 1, <2107> [1888] F.334; 4) type 1, burial <2108> [1898] F.336; 5) type 1, <2111> [1970] F.352; 6) type 1 animal bone <624> [1944] F.347; 7) type 1 elephant ivory <556> [1803] F.314; 8) type 1 iron <2110 > 1949 F.348; 9) type 1, 1908–9 discovery Z 1923.1597 B; 10) type 1, 1908–9 discovery Z 1923.1597 C; 11) type 1, 1908–9 discovery 1910.273; 12) type 2 < 2042> [1465] F.191; 13) type 2 < 2056> [1507] F.230; 14) type 2 < 2073> [1787] F.310; 15) type 3A <2105> [1882] F.331; 16) type 3A <2117> [1879] F.332; 17) type 3B, 1908–9 discovery 1910.275 B; 18) type 3A, 1908–9 discovery 1910.271; 19) type 4 iron <2074> [1788] F.311; 20) type 4 iron <2106> [1899] F.333; 21) type 4 1908–9 discovery 1910.270; 22) type 5 with mount <2069> [1737] F.302; 23) type 6 < 2112> [2010] F.367; 24) type 7 < 2109> [1933] F.344.

A number of the skeletons from the cemetery, chapter house and cloister garth/walk were accompanied by a single buckle on or around the pelvis (Figures 10,11). These are girdle buckles and indicate that these individuals were buried in clothing, which was highly regularised both in terms of the types of buckles used and the garments worn. This, plus the fact that all the individuals that buckles were associated with were osteologically and genetically determined to be male and osteologically old enough to be at least novices, indicates that they are probably members of the Austin friars (Cessford et al. 2022). Based on their osteological age ranges, these individuals can be situated within the academic lifecourse of a friar (Roth 1966 vol. I, 136–77) and contemporary ageing systems such as St Augustine’s six periods of a man’s life (Burrow 1986) (Table 2). Localised green staining identified on some bones generally relates to these buckles, plus a jetton associated with another skeleton (see below: girdle buckles and a jetton directly associated with skeletons). Black and purple staining appears to correlate with the presence of darker grave fills, indicating the oxidation of other, possibly naturally occurring, metals. The one exception to this (F.336) has staining on the top of the breastbone (manubrium) and the side of the head (right parietal), which may indicate copper alloy items such as a pendant and a pin that have entirely decayed. There were also burials that definitely lack accompanying buckles, these appear to have been buried in shrouds and are probably lay individuals. Although nails and other pieces of ironwork were found in some graves, the number and location of these means that they are unlikely to relate to coffins and are probably just incidental inclusions. There is no other convincing evidence for any coffins and few if any of the graves could have physically contained coffins.

**Table 2.** Academic lifecourse of Austin friars, compared to St Augustine’s six periods of a man’s life, osteological age categories and clothed burials from the Cambridge Austin friars.

Friar academic lifecourse	Length (years)	Min. age pre-Black Death (post-Black death in brackets)	St Augustine’s six stages of life	Osteological age categories	Burials
Childhood	14, later 10	0–13 (0–9)	*Infantia*/infancy (0–7), *pueritia*/childhood (7–14)	Neonate (<0.5), Infant (0.5–4), juvenile (5–12), adolescent (13–17)	
Novitiate at local friary	1	14–15 (10–11)	*Adolescentia*/adolescence (15–28)	Adolescent (13–17)	Cemetery: F.347: chapter house F.230
Solemn profession to join order	–	15 (11)	*Adolescentia*/adolescence (15–28)		
Studying grammar or logic	1	15–16 (11–12)			
Philosophy at *studium particulare*	3	16–19 (12–15)		Adolescent (13–17), young adult (18–25)	Cemetery: F.343
Theology at *studium generale ordinis*	1	19–20 (15–16)		Young adult (18–25)	
Student at *studium generale ordinis*	3	20–23 (16–19)			Cemetery: F.314, F.332: chapter house F.260, F.310
Ordination	–	24 (24, reduced to 18 in 1507)			
Cursor: various friaries	3	24–27 (19–22)		Young adult (18–25), young middle adult (26–35)	
Lector at *studium generale ordinis*	4	27–31 (22–26)	*Adolescentia*/adolescence (15–28), *juventus*/youth or young manhood (29–49)	Young middle adult (26–35)	Cemetery: F.333, F.348, F.352
Opponency at *studium generale ordinis*	1	31–32 (26–27)	*Juventus/*youth or young manhood (29–49)		
Baccalaureate (sentences) at *studium generale ordinis*	2	32–34 (27–29)			
Baccalaureate (Bible) at *studium generale ordinis*	1	34–35 (29–30)			
Inception at *studium generale ordinis*	1	35–36 (30–31)		Young middle adult (26–35), old middle adult (36–45)	
Master regent at s*tudium generale ordinis*	2	36–38 (31–33)		Old middle adult (36–45)	Cemetery: F.216, F.265, F.331, F.344
Fully academically qualified	–	38+ (33+)	*Juventus*/youth or young manhood (29–49), *aetas senioris* or *gravitas/*elder or settled life (50–69)	Old middle adult (36–45), mature adult (46+), mature adult with marked degenerative characteristics (60+)	Cemetery: F.302, F.367; Chapter house: F.191
Some friars freed from offices, some return to original friary		*c*. 60+	*Aetas senioris* or *gravitas/*elder or settled life (50–69), *senectus*/old age or decrepitude (70+)	Mature adult with marked degenerative characteristics (60+)	Cemetery: F.311

Although some post-depositional movement is possible, the individuals heads were in a variety of positions; predominantly facing upright (14), but also to the left (6) and right (4). A variety of arm/hand positions were identified. In general, the arms/hands were arranged symmetrically, although there are exceptions. In some instances the arms were placed along the sides of the body (7), whilst in others they were crossed with the hands over either the area of the hips/abdomen (13) or chest (10), including some in positions that have been interpreted elsewhere as mimicking praying (Atzbach 2016). At the Cambridge Austin friars arms along the side of the body are generally related to burial without girdle buckles that were presumably shrouded although there are exceptions, and all arm positions are associated with burials with associated girdle buckles that are assumed to have been clothed.

An instance of ‘translated’ burial in the chapter house has already been mentioned. One grave in the cemetery had been heavily disturbed and the bones, plus a buckle indicating this was a friar, replaced in the grave (F.352). Although the lower *c*. 0.5–1.0 m of the grave had been removed by a later wall footing, the presence of leg bones (both femurs and tibiae) and the absence of many upper body elements (both clavicles, right scapula, right humerus, left radius and ulna, manubrium and sternum, and three cervical vertebrae) indicates that the disturbance of the skeleton pre-dates and is unrelated to the truncation.

Several skeletons in the cemetery were truncated by later graves and other features and most of the disarticulated human remains are explicable as deriving from these truncated skeletons. In two cases the earlier skeletons were entirely removed and parts of them included in the backfill of later graves. These therefore represent additional individuals with no *in situ* remains. One of these represents *c*. 45% of a young middle adult (F.106, context [1014]) and the other *c*. 12% of a young adult male (F.343, context [1916]). Most of the bones were placed over the thorax of the later burial, but the crania and two long bones appear to have been deliberately arranged in part of the grave to the west of the body. In most cases, it appears that the disturbed bones, or at least the larger ones, were included in the backfill of the later grave or other feature with little or no evidence for careful placement. In one case, most of the disturbed bones were in the backfill over undisturbed parts of the first burial (F.309, context [1766]). The *in situ* and redeposited material constitutes *c*. 70% of the skeleton, with the right clavicle, manubrium, sternum, both patellae and some of the ribs, and feet bones missing. This suggests a relatively thorough, but incomplete, attempt to recover disturbed bones. Once disturbance of the skeleton was recognised a portion of the grave over the undisturbed part of the skeleton was deliberately re-excavated to permit this placement.

At least thirteen individuals from the cemetery were disturbed by foundation trenches during the construction of the friary cloisters: nine involving the upper parts of the skeleton and four the lower. Some of the smaller bones including metacarpals, ribs, vertebrae and portions of humerii and scapulae were redeposited in the fill of the foundations. Other elements were placed in a charnel pit (F.397): these were principally crania and mandibles (MNI 4), but humerii and femurs were also present.

### The skeletons

Thirty-eight inhumations from the cemetery (n = 32, including the disturbed burial but excluding the two individuals only present as disarticulated material in later graves) and chapter house (n = 6) are discussed. This is supplemented as appropriate by the material from the cloister garth/walk (n = 47) and church (n = 1) recovered in 1908–9 and studied by Sarah Inskip. There was generally a high level of skeletal preservation, although a significant number of skeletons had been truncated. The following selected summary acts as a gateway into the larger, osteological discussion with differential considerations, in the Supplementary Materials (Human Skeletal Remains section). Therein burial position, preservation and taphonomy, demography, trauma (n = 7, *c*. 18%), degeneration (n = 18, *c*. 47%), enthesophytes (n = 14, *c*. 37%), inflammation and possible infection (n = 11, *c*. 29%), neoplastic disease (n = 5 *c*. 13%), endocrine (n = 9, *c*. 24%), metabolic (n = 8, *c*. 21%) and developmental disorders (n = 12, *c*. 32%) and oral pathology (n = 21, *c*. 55%) are discussed. There is also a discussion on the demography and pathology of the disarticulated assemblage and a catalogue of burials.

**Figure 12.**
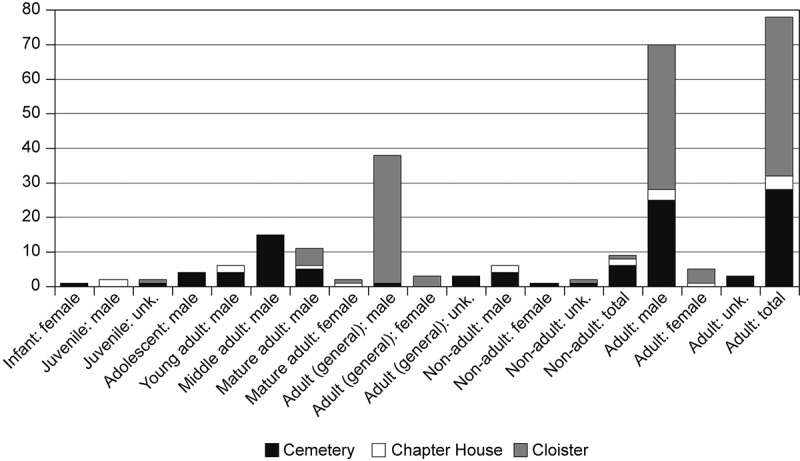
Bar chart of age and sex of individuals from the early cemetery, chapter house and cloister walk/garth of the Cambridge Austin friars. Sex identifications from both osteological and aDNA evidence, values include identified disarticulated individuals.

The assemblage stands out for its high representation of adults and males (Table 3; Figure 12). Although this is perhaps unsurprising in a friary context, elsewhere some areas of friary churches and cloisters were routinely used for female and non-adult burials (Gilchrist and Sloane 2005a, 224). Some of the adult males buried at the friary were members of the laity, as were women and girls and a boy who was too young to be novices. One morphologically mature adult genetically identified as female (F.146) was buried in the chapter house; while one infant who was genetically identified as female (F.106) came from the cemetery. There were also four females in the cloister garth/walk, so overall 7% of the skeletons were female. At broadly contemporary parish and hospital cemeteries in Cambridge 50.5% and 40.9% of skeletons were female respectively (Inskip in prep). At the Kingston upon Hull Austin friars there were 144 adult males and 56 adult females (28.0% female) (Gilchrist and Sloane 2005b).

**Table 3.** Demographic summary for the Cambridge Austin friars. Figures in brackets include probably male/female individuals. Sex identification includes osteological and aDNA information. Total for the early cemetery includes two entirely disarticulated individuals.

Sex	Infant (0–4)	Juvenile (5–12)	Adolescent (13–18)	Total non-adult (0–18)	Young adult (18–25)	Young middle adult (26–35)	Old middle adult (36–45)	Mature adult (46+)	Adult (18+)	Total adult (18+)	Total
Cemetery male	–	–	4	4	3(4)	7	8	5	–(1)	23(25)	27(29)
Cemetery female	1	–	–	1	–	–	–	–	–	–	1
Cemetery indet.	–	1	–	1	–	–	–	–	3	3	4
Cemetery total	1	1	4	6	4	7	8	5	4	28	34
Chapter house male	–	2	–	2	2	–	–	1	–	3	5
Chapter house female	–	–	–	–	–	–	–	1	–	1	1
Chapter house total	–	2	–	2	2	–	–	2	–	4	6
Cloister male	–	–	–	–	–	–	–	4	27(37)	31(41)	31(41)
Cloister female	–	–	–	–	–	–	–	1	–(3)	1(4)	1(4)
Cloister indet.	–	–	1	1	–	–	–	–	1	1	2
Cloister total	–	–	1	1	–	–	–	5	41	46	47
Church male/total	–	–	–	–	–	–	–	–	1	1	1
Friary male	–	2	4	6	5(6)	7	8	10	28(39)	58(70)	64(76)
Friary female	1	–	–	1	–	–	–	2	–(3)	2(5)	3(6)
Friary indet.	–	1	1	2	–	–	–	–	4	4	6
Friary total	1	3	5	*9*	6	7	8	12	46	*79*	88

Nine individuals were aged under 18 (10%), compared to 27% and 17% at the contemporary Cambridge parish and hospital cemeteries (Inskip in prep) and 12% at the Kingston upon Hull Austin friars (Gilchrist and Sloane 2005b). As boys could initially join as novices aged 14 and this later fell to 10, only two individuals were definitely too young to be novices (1 male, 1 female). The greatest proportion of the individuals at the Cambridge friary fall within the broader middle adult range (aged 26–45).

Seven individuals from the cemetery or chapter house (18%) have some form of trauma; the population shows a lower occurrence of fractures than a contemporary parish cemetery in Cambridge but higher than at a local hospital (Dittmar et al. 2021). Some of these traumas have great osteobiographical interest, if not for their mechanism of injury, then for their subsequent quality of life. The most noteworthy is a young adult male friar (F.332) buried in the cemetery. Bilateral leg fractures and neck fractures showed no signs of healing, indicating this trauma occurred at or near the time of death. The leg fracture pattern is clinically associated with a high impact trauma and indicates a force directed from behind and to the right of the individual. Bilateral femoral fractures carry a high risk of death, especially with the potential of associated vascular injury to the femoral arteries. Severe blood loss and haemorrhagic shock would have been real threats in this scenario. The trauma to the cervicothoracic junction connecting the neck and upper back is likely to represent an associated whiplash injury. One can only speculate on the mechanism, but being hit by a runaway cart from behind is one potential explanation. This individual either died almost immediately after the injury, or at most within a couple of weeks from complications. The legs were in normal articulation proximal to the fractures, yet distally the limbs had moved upwards against the respective proximal elements and were rotated to the right, possibly as a consequence of rigour-mortis and handling of the corpse. Given the likely state of the body, it is worth noting that this individual appears to have received a careful clothed burial indistinguishable for that afforded to other friars.

The nightstick (‘parry’) fracture, seen in the left ulna of mature adult male friar (F.191) buried in the chapter house could be due to a fall, but is more likely to relate to a defensive posture against an attack. While friaries were generally peaceful places, this potential act of violence could represent a deliberate act of anti-mendicant aggression (see below: anti-fraternal criticisms), although as friars could also be perpetrators of violence (Geltner 2012) this friar might have been either instigator or victim. There is textual evidence of more extreme violence linked to the friary as John Cook, servant and cook for the Cambridge Austin friary, was murdered and his body deposited near a windmill in the town fields in 1386 (Roth 1966 vol II, 229 no. 575).

As will be demonstrated many individuals buried at the friary bear skeletal evidence of biomechanical adaptation and disease; the latter from infections to endocrine, metabolic and developmental disorders. Bearing in mind the osteological paradox (Wood et al. 1992), this could imply that they were in relatively good health with strong immune systems that were able to mitigate, for a period, the detrimental effects of their condition(s). The group is predominantly defined by the level of salient skeletal degeneration. Excluding osteoarthritis, fifteen individuals (39%) had some form of osteogenic remodelling or porosity around and/or within articulations. The most affected element was the back, indicated by spondylophytes and schmorl’s nodes. Osteoarthritis was defined by evidence of eburnation and Diffuse Idiopathic Skeletal Hyperostosis (DISH), with femoroacetabular impingements included as a known though not definitive precursor to osteoarthritis, and this was noted in eight individuals. Although DISH is not typically considered as an osteoarthritic condition, it is included here as a rheumatological disorder with a (theoretically) similar pathogenic pathway to osteoarthritis (rationalised under degeneration in Supplementary Materials: Degeneration).

F.260, a young adult friar, had spondylolysis and intervertebral disc degeneration (IDD) between L4 and L5. Clinically common during adolescence, genetic predisposition and a higher Body Mass Index are seemingly correlated with this condition, but not physical activity. Expressions for the psoas muscle attachment were also noted on the right side of L2–L4 of this individual, which may relate to a biomechanical compensation of the lumbosacral instability.

**Figure 13.**
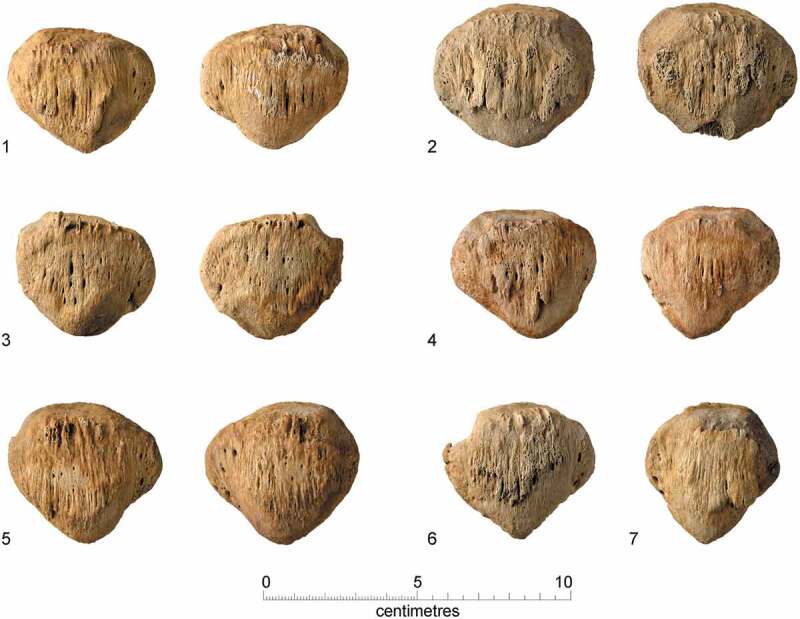
Enthesophytes on the anterior surfaces of the patellae, possibly related to praying: 1) F.195, right and left; 2) F.198, right and left; 3) F.265, right and left; 4) F.333, right and left; 5) F.367, right and left; 6) F.336, right; 7) F.311, left.

Enthesophytes, abnormal bony expressions at tendon insertion sites extending in the direction of ligament or tendon pull (Hardcastle et al. 2014), were noted in fourteen individuals, affecting the head, neck, back, upper arm, elbow, forearm, hands, pelvis, thigh, knee and ankle. Enthesophytes were seen on the anterior surface of the patellae of seven individuals (F.195, F.198, F.265, F.311, F.333, F.336 and F.367; Figure 13), five friars and two individuals whose status is unknown. Clinically referred to as ‘patella tooth’, it likely relates to a mechanically induced overuse injury (Gholve et at, 2007; Smith and Varacallo 2018). Activities such as running, jumping and kneeling repetitively strain the patellar tendon, via the contraction of the quadriceps muscle thus pulling on the patella and tibial tuberosity entheses. This is a self-limiting condition and usually resolves and becomes asymptomatic by adulthood. Sustained activities may continue to perpetuate the condition, where habitual isometric contractions of the quadriceps muscle during activities such as kneeling may maintain these expressions (Gholve et al. 2007; Smith and Varacallo 2018). One possible interpretation is that the patellae enthesophytes may be linked to friars habitually kneeling in prayer from adolescence. In the High/Late Medieval period individuals might adopt several ‘humble’ postures for prayer, including kneeling, standing, sitting or lying prostrate (Tugwell 1985). Kneeling with clasped hands was the default position and is how the Austin friars presumably prayed privately.

One friar and one individual whose status is unknown (F.217 and F.302) had enthesophytes along the medial edge of the femoral linea aspera, whilst the friar (F.302) also had medially directed enthesophytes over the lesser trochanter. The former corresponds to the insertions of adductor magnus and adductor longus muscles, which flexes, adducts and medially rotates the thighs at the hips. The latter corresponds to the activity of the iliopsoas muscle, which flexes the trunk and thigh at the hip and externally rotates the thigh at the hip. These expressions may relate to the habitual isometric contraction of muscles during activities such as horse-back riding (Supplementary materials: Enthesophytes). There is considerable evidence for horses at the friary (horseshoes, horse bones and stables), supporting this interpretation.

A mature adult female was buried in the chapter house (F.146), based on the presence of a jetton and coin in the grave she was interred after *c*. 1500. The woman shows signs of age-related degeneration in the shoulders and lower back, as well as conditions affecting her teeth including tooth loss, caries and calculus. There were also bony formations that developed in response to habitual loading, which usually develop over extended periods of time. Her skeleton was singularly noted for rugosity and enthesophyte formations, at the tuberosities of both humerii and interosseous borders of both forearms and the fingers of the right hand. All these expressions probably relate to a habitual activity involving resisting a downward pull. This suggests an active lifestyle, while burial in a chapter house is typically indicative of relatively high social status. Either this woman still undertook considerable physical activity despite her social status, or some other factor such as perceived sanctity meant that she was buried in the chapter house despite being of lower social status. This woman also suffered from a serious long-term health condition. She had notable bilateral bone formation around the tibiae and fibulae of both legs, particularly distoanteriorly, taking a swollen appearance. This possibly relates to an infection, aggravated by long periods of standing. Various possible causes exist, including a haematologically derived osteomyelitis or osteitis (possibly via chronic leg ulcers). A jetton found on the medial side of right tibia is probably linked to this condition (see below: Girdle buckles and a jetton directly associated with skeletons). The evidence for habitual activity in the arms would have exacerbated the venous inefficiency, where load/weight would have increased the vascular pressure in her legs thus causing greater damage and making the condition chronic.

**Figure 14.**
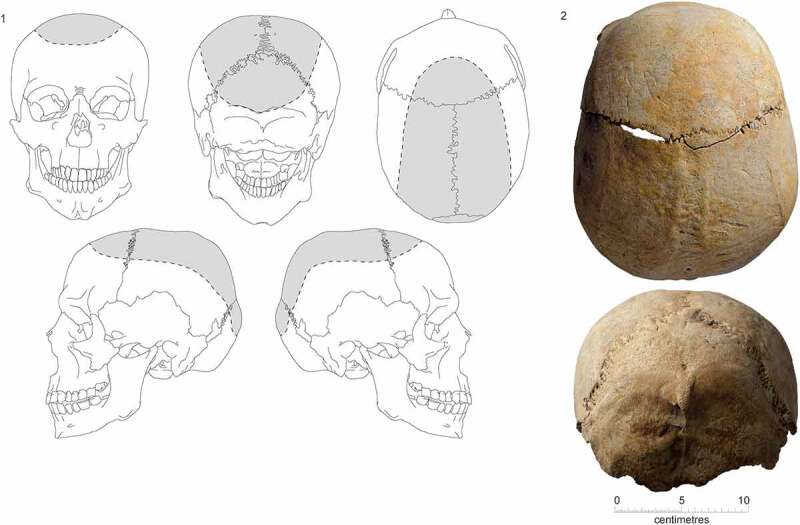
Inflammation and sclerosis on cranium: 1) reconstruction of typical extent shown in grey; 2) photographs of crania F.309.

Seven individuals (18%) have variable degrees of well delineated inflammation and sclerosis, confined to the calotte region of their skulls (Figure 14). There appear to be different levels of severity, where three individuals (F.328, F.343 and F.355: one friar, one lay and one probably lay) have periosteal reactions only, whereas four individuals (F.191, F.260, F.309 and F352; three friars and one status unknown) also have sclerotic bone and venous sulci involvement. Combined with an absence of extracranial periosteal reactions one possible cause of the more severe cases may relate to tonsuring, where the hair was shaved or clipped from the crown of the head, with an unclean blade.

This could have lead folliculitis, a severe form of bacterial infection (Karthikeyan 2009). Tonsuring symbolised obedience to a religious lifestyle and visibly marked a separation from the laity. Austin friars were tonsured every 15 days from Easter to late September and then eight times before the following Easter (Andrews 2006, 126), which would usually equate to 18–20 times per year.

Another possibility is a form of ringworm caused by an aerobic fungi infecting skin and hair, which is easily transmitted through touch (Piggott and Friedlander 2012). Sources of this infection can come from humans and animals such as dogs and horses. Whilst both have contagious potential, only horses are known to have been present at the friary. This infection is likely to have been common in medieval England, and was known as *Tineas* during this period. Differentially, these reactions maybe related to haematologically disseminated skeletal tuberculosis, particularly for individual F.343, where hypervascularisation of the vertebrae were noted (see Supplementary Materials: tuberculosis).

Three lay individuals (F.312, F.315 and F.343), two adolescents and a young adult, had salient indications of skeletal tuberculosis, which is usually secondary to pulmonary tuberculosis. Tuberculosis was probably relatively common during this period. Roberts (2009) notes that there was an increase in infection compared to the earlier medieval period, and tuberculosis known to be prevalent in the seventeenth to nineteenth centuries, being fatal for about 15% of the UK population in the 1680s (Roberts 2020). Although this has been rationalised as a result of increased population density and resulting reduction in ‘sneezing distance’ (essential for bacterial transmission) even rural populations such as Wharram Percy had notable case numbers, maintained by social and economic links (Mays et al. 2001).

Individual F.140, a young middle adult lay individual, was noted for bilateral striated periosteal new bone over the femorae and the interosseous surface of right tibia, together with bilateral lesions over the anterodistal aspects of the tibiae. This is concordant with a treponemal infection (Walker et al. 2015), but without the skull, this is a tenuous diagnosis (for other possibilities see Supplementary Material: Possible bacterial infection). Eight individuals, six friars and two members of the laity, were noted for cribra orbitalia (F.106, F.232, F.310, F314, F.333, F.336, F.347 and F.352).

Two individuals, both friars, showed evidence of endocrine disorders. F.352, a young middle adult male buried in the cemetery whose skeleton had been heavily disturbed, had bilateral cribra orbitalia, ossified costochondral cartilage and bilateral medial torsion (anteversion) of the femora. Although ossified costochondral cartilage is considered an age-related change in individuals over 40 years of age, it is unusual in younger people and can often be associated with malignancy, autoimmune and endocrine disorders, particularly of the thyroid such as Graves’ Disease. Due to this individuals’ estimated age, there may be a pathogenic link with the ossified costochondral cartilage and femoroacetabular impingement (FAI), as there is also a known pathomechanical association between femoral anteversion and an onset of FAI. Differentially, there is also a secondary association between hip malformations and thyroid malignancy/disorder, which is also associated with premature costochondral ossification. A juvenile novice buried in the chapter house (F.230), had extraordinarily large lesions in the skull, left arm, right collar bone, left leg and right femur. Several possible causes exist, but these are probably linked to a serious endocrine disorder osteitis fibrosa cystica, with tuberculosis or Gorham’s syndrome also possible. Osteitis fibrosa cystica is commonly defined by long standing end-stage hyperparathyroidism, a tumour on the parathyroid glands is usually responsible. This results in elevated levels of parathyroid hormone, which in turn causes excessive resorption of bone. Whatever the cause of the skeletal changes, the presence of *Yersinia pestis* aDNA (see below: ancient DNA) indicates that this individual probably died of plague, with their pre-existing condition possibly a contributory factor.

There were five possible cases of gout, affecting two friars, one member of the laity and two individuals whose status is unknown, which may be linked at least in part to diet (see isotopic and faunal evidence below) (see Supplementary Material: gout). Three possible cases of a scorbutic episode (scurvy) were identified, affecting one friar, one member of the laity and one individual whose status is unclear (see Supplementary Material: vitamin C deficiency (scurvy)).

A not uncommon range of rarer conditions that were noted included distal symphalangism (five instances), spina bifida occulta (five instances), craniosynostosis (two instances). Individuals had lumbosacral transitional vertebrae (two instances), possible Crowned Dens Syndrome (two instances), a sternal foramen (one instance), an accessory transverse foramen (one instance) and an abnormal temporal styloid process (one instance).

Twenty-nine individuals had teeth present. In terms of oral health, the results were broadly typical of the period. The most salient trait was dental calculus (n = 19), which can be considered to reflect rudimentary oral hygiene. There did not appear to be a correlation of increased calculus with age. Carious lesions (n = 8), antemortem tooth loss (n = 6) periapical abscesses (n = 4) and periodontal disease (n = 2) are all pathogenically correlated. Seven individuals were noted for enamel hypoplasia, suggesting a poor growth environment during development.

Sediment samples indicate that infection with the intestinal parasite roundworm *(Ascaris lumbricoides)* was relatively common, affecting over half the individuals examined (Wang et al. Forthcoming).

### Stable isotopes

#### By Alice Rose

**Figure 15.**
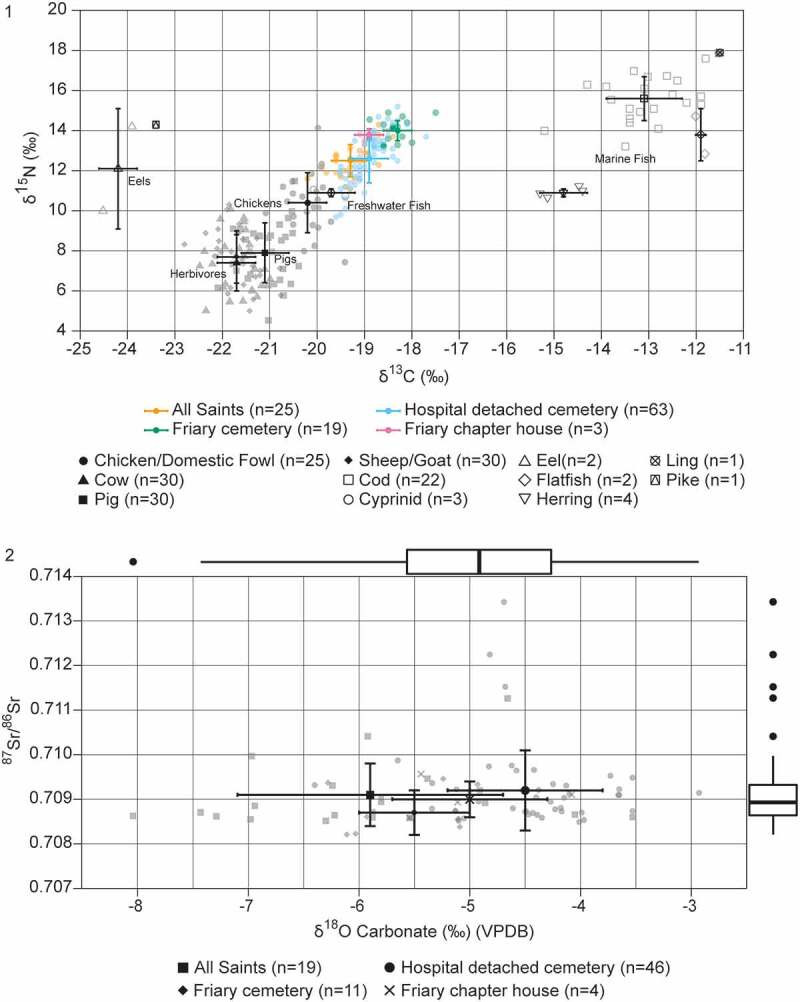
Stable isotopes: 1) scatterplot with mean and 1SD values, showing the Austin friars plus Hospital of St John and All Saints by the Castle parish in Cambridge adult male rib collagen δ^13^C and δ^15^N values, distinguishing by cemetery. Also shown are faunal bone collagen δ^13^C and δ^15^N values from medieval Cambridge (see Rose 2020; Rose and O’Connell in prep. for data); 2) scatterplot with mean, 1SD and boxplot showing the Austin friars plus Hospital of St John and All Saints by the Castle parish in Cambridge enamel δ^18^O _CO3_ and ^87^Sr/^86^Sr values, distinguishing by cemetery. Adapted by the CAU from original by Alice Rose.

Stable isotopes relating to diet (δ^13^C and δ^15^N: dentine from second premolar or second molar n = 20, rib n = 28) and mobility (δ^18^O_CO3_ n = 21 and ^87^Sr/^86^Sr n = 16) from individuals buried at the friary were analysed and compared to individuals from broadly contemporary parish and hospital cemeteries in Cambridge by the After the Plague project, using the standard methodologies of that project (Rose and O’Connell in prep; Rose. 2020). All friary individuals from the 2016–19 excavations that were >50% complete and had suitable tissues present were sampled for isotope analysis. δ^13^C and δ^15^N values from both rib collagen, which relates to diet in the years before death, and dentine collagen, representing childhood diet, were measured, allowing lifecourse changes to be considered (Sealy 1995) (Table 4; Figure 15, upper). There was an almost uniform distinct positive shift in δ^13^C and δ^15^N values between childhood and adulthood in the men from the friary cemetery (dentine mean δ^13^C: −18.9 ± 0.4‰, δ^15^N:12.4 ± 1.0‰, n = 13, rib mean δ^13^C: −18.3 ± 0.3‰, δ^15^N:14.0 ± 0.5‰, n = 19), which is not present at the other cemeteries in the town (parish male dentine mean δ^13^C: −19.5 ± 0.3‰, δ^15^N:11.9 ± 0.9‰, n = 14, rib mean δ^13^C: −19.3 ± 0.4‰, δ^15^N:12.5 ± 0.8‰, n = 25; hospital male dentine mean δ^13^C: −19.1 ± 0.5‰, δ^15^N:12.1 ± 1.6‰, n = 31, rib mean δ^13^C: −18.9 ± 0.4‰, δ^15^N:12.6 ± 1.2‰, n = 63). The rib collagen isotope values of men from the friary cemetery are characterised by a much smaller range and comparatively higher δ^13^C and δ^15^N values than those from the other contemporary Cambridge cemeteries (friary range δ^13^C: 1.4‰, δ^15^N: 1.7‰; parish range δ^13^C: 1.8‰, δ^15^N: 3.2‰; hospital range δ^13^C: 1.9‰, δ^15^N: 5.5‰). Although dietary interpretation is complex, the higher δ^13^C and δ^15^N values probably relate to greater levels of marine fish consumption and are theoretically compatible with an entirely or largely meat free diet at the friary (other animal proteins such as eggs, milk and cheese can also result in high δ^15^N values). The values could indicate a relatively homogenous institutional friary diet, although as they are similar for men interpreted as friars and as members of the laity this suggests that these groups shared broadly similar diets indicating that the friary diet was not particularly unusual. Although only a small number of individuals from the chapter house were analysed (dentine collagen δ^13^C and δ^15^N n = 4, rib collagen δ^13^C and δ^15^N n = 5), the results are broadly similar to those from the earlier cemetery, although they did not exhibit the dramatic positive shift in δ^15^N values between childhood and adulthood seen in the cemetery individuals. The sole adult woman had a rib collagen δ^15^N value comparable to the men (female 13.6‰; male chapter house mean 13.8 ± 0.3‰ n = 3). This was higher than those for most of the other analysed adult women studied from the local parish (12.3 ± 0.5‰ n = 13) and hospital (12.2 ± 1.0‰ n = 44)), but not exceptional as four women from the hospital had higher values.

**Table 4.** Summary statistics for dentine and rib collagen δ^13^C and δ^15^N values and tooth enamel δ^18^O_CO3_ and ^87^Sr/^86^Sr values for individuals from the friary. Isotopic values in teeth should be representative of the same growth period in all individuals so the data is not separated by adult/non-adult.

		Cemetery all	Cemetery adult	Cemetery non-adult	Chapter house all	Chapter house adult	Chapter house non-adult
Dentine collagen δ^13^C (‰) (VPDB)	No.	16	13	3	4	4	0
	Mean	−19.0	–	–	−19.0	–	–
	SD	0.5	–	–	0.5	–	–
	Median	−19.0	–	–	−19.0	–	–
	Min	−20.0	–	–	−19.7	–	–
	Max	−18.3	–	–	−18.4	–	–
	Range	1.7	–	–	1.3	–	–
Dentine collagen δ^15^N (‰) (AIR)	No.	16	–	–	4	–	–
	Mean	12.1	–	–	12.8	–	–
	SD	1.4	–	–	2.1	–	–
	Median	12.3	–	–	13.6	–	–
	Min	9.3	–	–	9.8	–	–
	Max	14.0	–	–	14.3	–	–
	Range	4.7	–	–	4.5	–	–
Rib collagen δ^13^C (‰) (VPDB)	No.	23	19	4	5	4	1
	Mean	−18.4	−18.3	−18.9	−19.1	−18.9	−19.7
	SD	0.4	0.3	0.6	0.4	0.3	0.0
	Median	−18.4	−18.4	−18.9	−19.1	−19.1	−19.7
	Min	−19.7	−18.9	−19.7	−19.7	−19.1	−19.7
	Max	−17.5	−17.5	−18.3	−18.5	−18.5	−19.7
	Range	2.2	1.4	1.4	1.2	0.6	0.0
Rib collagen δ^15^N (‰) (AIR)	No.	23	19	4	5	4	1
	Mean	13.8	14.0	12.9	13.6	13.7	13.3
	SD	0.9	0.5	1.6	0.3	0.2	0.0
	Median	14.1	14.1	13.4	13.6	13.7	13.3
	Min	10.6	13.2	10.6	13.3	13.5	13.3
	Max	14.9	14.9	14.2	14.0	14.0	13.3
	Range	4.3	1.7	3.6	0.7	0.5	0.0
Tooth enamel δ^18^O_CO3_ (‰) (VPDB)	No.	17	14	3	4	2	2
	Mean	−5.6	–	–	−5.0	–	–
	SD	0.6	–	–	0.7	–	–
	Median	−5.5	–	–	−5.3	–	–
	Min	−6.5	–	–	−5.5	–	–
	Max	−4.2	–	–	−4.1	–	–
	Range	2.3	–	–	1.4	–	–
Tooth enamel ^87^Sr/^86^Sr	No.	12	10	1	4	2	2
	Mean	0.7087	–	–	0.7090	–	–
	SD	0.0004	–	–	0.0004	–	–
	Median	0.7086	–	–	0.7090	–	–
	Min	0.7082	–	–	0.7086	–	–
	Max	0.7095	–	–	0.7096	–	–
	Range	0.0013	–	–	0.0010	–	–

δ^18^O_CO3_ and ^87^Sr/^86^Sr values from tooth enamel (second premolar or second molar) were also analysed (Figure 15, lower). These isotopes can indicate whether the isotopic signature of the enamel, indicating childhood water consumption, matches the expected signature of where they were buried, allowing for interpretations of mobility and childhood origins (e.g. Bentley 2006; Pederzani and Britton 2019). The estimated ‘local’ ^87^Sr/^86^Sr value for central Cambridge is 0.7080–0.7090 (Evans, Chenery, and Montgomery 2012; Evans et al. 2018). Cambridge is within the lower rainfall zone of the British Isles (Evans, Chenery, and Montgomery 2012) and a suggested min-max range for δ^18^O_CO3_ (VPDB) for Cambridge is currently −5.3‰ to −4.1‰ (Evans et al. 2018), although δ^18^O values can vary greatly (Lightfoot, and O’Connell 2016). The δ^18^O_CO3_ values (friary cemetery mean −5.6 ± 0.6‰, n = 17; friary chapter house mean −5.0 ± 0.7‰, n = 4) and ^87^Sr/^86^Sr values (friary cemetery median 0.7086, n = 12, friary chapter house median 0.7090, n = 4) from the friary are tightly clustered, indeed more so than the contemporary Cambridge parish and hospital cemeteries (Figure 15, lower). When considered as a whole ‘friary’ group there are no outliers, also in contrast to the parish and hospital cemeteries. The simplest explanation is that the individuals, including both friars and laity, are all of local childhood origin, indicating that although the friary was an international institution, it still attracted the majority of its associates from a local catchment area. However, interpretations are hindered by the huge potential for variability in δ^18^O_CO3_ values (Lightfoot, and O’Connell 2016) and the homogeneity of the estimated bioavailable ^87^Sr/^86^Sr baseline for East Anglia (Evans, Chenery, and Montgomery 2012; Evans et al. 2018), meaning evidence of short-distance, and even some longer-distance migration from areas with similar geology, may be masked.

### Ancient DNA

#### By Christiana L. Scheib

Ancient DNA (aDNA) from the teeth of 28 individuals buried at the friary was analysed as part of the After the Plague project and compared to individuals from broadly contemporary parish and hospital cemeteries in Cambridge, using the standard methodologies of that project (Hui et al. in prep). Given the relatively low overall number of skeletons from the 2016–19 excavations all individuals with surviving teeth were sampled. In addition to the human aDNA, all samples were screened for pathogen aDNA.

The genetic sex was identified as male (XY, n = 21), consistent with male but not female (XY but not XX, n = 5) or female (XX, n = 2). This generally supported the osteologically identified sex of adults, including several instances where the osteological identification was only probable or possible. The osteological identification of one individual from the chapter house as female was confirmed (F.146), while another individual from the chapter house osteologically identified as possibly female was shown to be genetically consistent with male (F.310). Two non-adults where sex could not be identified osteologically were genetically sexed, an infant (F.106) initially identified as possibly male using methods outlined by Schutkowski (1993) was genetically consistent with female. The friary had the highest deviation from an equal sex ratio of any medieval burial ground studied from High/Late Medieval Cambridge.

Maternal haplogroups were identified for 23 of the 28 individuals, the variability is broadly what might be anticipated and none of the haplogroups is unexpected for this context. There is no aDNA evidence suggesting that any of the individuals are particularly likely to be of non-British origin and as a group they are no more ‘exotic’ than the individuals from the contemporary Cambridge parish or hospital cemeteries. Fifteen individuals from the friary had high enough quality aDNA to analyses genetic relatedness, this demonstrated that there were no first- or second-degree relations within this group. This contrasts with the contemporary parish cemetery, where both first- and second-degree relations were identified, but is similar to the hospital cemetery. A lack of comparative aDNA studies means that it is impossible to know how the absences of non-local individuals and genetic relations compares to other religious institutions of the period in England.

One individual from the cemetery tested positive for *Yersinia pestis* (F.355), the bacterium that causes plague. The strain identified was ‘potentially identical to other Black Death genomes’ (Spyrou et al. 2019, 3), indicating that this individual probably died of plague in 1349 (Cessford et al. 2021). Three individuals from the chapter house who died *c*. 1450–1538 also tested positive for *Y. pestis* (F.190, F.230, F.310), indicating that they probably died of plague in one or more later outbreaks (Cessford et al. 2021; Spyrou et al. 2019). Unfortunately, two of the samples were too low coverage to determine if the strains from the three individuals were identical or not. While underlying health conditions may also have been a factor in some cases, the presence of *Y. pestis* aDNA in teeth indicates septicaemia. Without antibiotic intervention, vulnerability to death increases to 50–90% of cases (Sexton and Stout 2018).

### Geometric morphometrics

#### By Bram Mulder

Analysis of the structural properties of skeletons can be useful in interpreting past lifestyles. Cortical and trabecular bone structure in both humerii and one femur and tibia from 22 individuals were quantified, using micro-computed tomography, and compared individuals from broadly contemporary parish and hospital cemeteries in Cambridge (Mulder 2020; Mulder, Saers, and Stock. in prep; Mulder et al. 2020). The amount of bone tissue in the skeleton is affected by numerous factors, such as genetics, diet and hormonal status. In the friary there was a tendency to have more tissue in both the cortical and trabecular compartments of the limbs, but this was rarely of a meaningful order as determined by statistical comparisons between the friary, parish and hospital sites. While the individuals from these three studied sites may have differed slightly in lifestyle and biological background, they appear strikingly homogeneous, especially when interpreted within the context of overall human skeletal variation (e.g. Ruff 2018).

**Figure 16.**
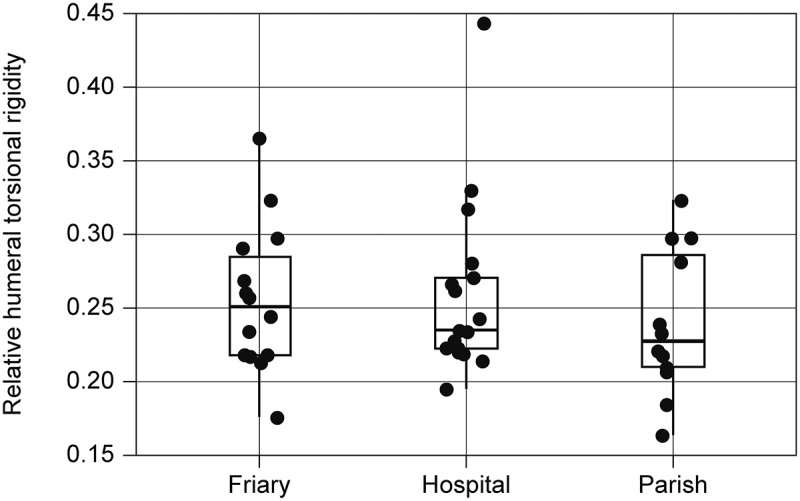
Relative humeral torsional rigidity. Comparison of rigidity of the left humerus, relative to femoral rigidity, in males from the Austin friars plus Hospital of St John and All Saints by the Castle parish in Cambridge (see Mulder 2020 for values). Adapted by the CAU from original by Bram Mulder.

In interpreting the contribution of activity to skeletal morphology, a bone’s mechanical competence is more relevant than tissue quantity alone. Strength or rigidity is therefore often used as a proxy for activity history. In evaluating torsional rigidity of the long bones for the three sites, humeral properties were divided by femoral values, to reduce the effect of systemic differences between individuals. This revealed that the distributions of the friary, hospital and parish sites were also very similar in this analysis (Figure 16). A comparison of bilateral asymmetry in humeral rigidity also showed little variation between groups, with average asymmetries between 7–11%. The one adult female from the chapter house (F.146) had only a single femur sufficiently preserved for analysis, this presented with greater rigidity than any of the other females in the comparative sample (see above: the skeletons).

The homogeneity in the results suggests a lack of profound lifestyle differences between those buried in the friary, parish and hospital. Major variations in upper limb involvement in habitual tasks would be expected to have generated more contrasting skeletal morphologies.

### Scholars at the friary?

#### By John E. Robb

Analysis of individuals buried at the Hospital of St John in Cambridge has identified a number of adult men with markedly symmetrically and often relatively lightly built humeri. This symmetry, supported with other evidence, has been interpreted as indicating relatively low levels of habitual physical work during the formative teenage years and at the hospital may be linked to the presence of ‘poor scholars’ from the University (Robb in prep b). Two adult men with similar marked symmetry of the upper limbs were identified at the friary, both of them from the early cemetery and accompanied by buckles and interpreted as friars (F.334 and F.344). Most of the individuals (18 of 20; 90%) who received clothed burial at the friary did not fall into this category. This suggests that despite its educational role, skeletally identifiable ‘scholars’ were in a minority at the Cambridge Austin friars and in terms of physical activity most friars were indistinguishable from the bulk of the local population, at least in the investigated burial areas.

## Life threats

It is rarely possible to identify the cause of death, with its specific modern medico-legal connotations, from archaeological burials. So, it is preferable to consider statistically probable life threats. Texts of the period are also largely mute about specific individuals, although we know that one friary servant was murdered and it is likely but unprovable that the prior John de Comberton who died 1348–51 was a victim of the Black Death.

An exception to this silence is where individuals test positive for *Yersinia pestis*, as the evidence of septicaemia indicates they are likely to have died of plague. This is the case for four individuals at the Cambridge Austin friars, representing 14% of those tested and *c*. 30−40% of those tested and likely to have died during the Second Plague Pandemic (Cessford et al. 2021). It is also almost certain that an individual with bilateral leg fractures died due to complications of this trauma. Identifying significant life threats for five individuals out of 40 (12%) buried in the Cambridge Austin friars cemetery and chapter house is a valuable achievement, especially as later truncation severely limits the possibility for some individuals.

More speculatively, and less certainly, potential life threats can be suggested for a number of other individuals (Table 5). Although old age is not strictly speaking a cause of death, four individuals that were probably aged over 60 can be thought of as dying of ‘natural causes’, in the sense of current understandings from clinical/medical research where degradation of cellular and tissue homoeostasis, predisposed by oxidative damage, telomere shortening and genomic instability causes malfunction and an increased vulnerability to death.

**Table 5.** Possible life threats and contributory factors for individuals buried in the Cambridge Austin friars cemetery and chapter house.

Possible life threats and contributory factors	F.	Type of individual
Blood loss and haemorrhagic shock or later complications from bilateral leg fractures. High energy trauma, possibly caused by cart accident or fall from height	332	Young adult friar, likely to be student
Plague	355	Young middle adult male benefactor
Plague	190	Juvenile male, child of benefactor
Plague, a pre-existing condition that caused large skeletal lesions was probably a contributory factor	230	Juvenile novice
Plague	310	Young adult friar, likely to be student
Natural, old age related	311	Mature probably old friar
Natural, old age related	334	Mature probably old friar
Natural, old age related	336	Mature probably old friar
Natural, old age related. A long standing condition, probably a systemic infection affecting the legs, may have been a contributory factor	146	Mature probably old woman, benefactor
Childhood condition or disease, including infectious diseases such as pneumonia, malaria, diarrhoea and measles	106	Infant girl, child of benefactor
Tuberculosis	312	Adolescent male, child of benefactor
Tuberculosis	315	Young adult male, benefactor
Tuberculosis	343	Young adult friar, likely to be student
Complications from violent episode or fall that resulted in a healing parry fracture	191	Mature adult friar
Bacterial infection, possible treponemal disease or Paget’s disease of bone	140	Young middle adult male, benefactor
Endocrine disorder, possibly Graves’ Disease	352	Young middle adult friar, possibly undertaking advanced studies

At the other end of the age spectrum an infant girl may well have died from one of the common causes of infant and childhood mortality including pneumonia, malaria, diarrhoea, measles and obstetric issues (Robb et al. 2021, Table 2). Tuberculosis was a common life threat in the past, so three individuals who show signs of it may have died of it.

In total suggestions concerning life threats can be made of 16 individuals: 40% of the total of individuals from the cemetery and chapter house, rising to 50% of the 32 individuals with a reasonable degree of skeletal survival. The single infant death is undoubtedly an underrepresentation for High/Late Medieval Cambridge as a whole, and there were no evidence deaths of either mothers or neonates during childbirth, as are attested as a life threat at other local cemeteries. There are also a wide range of other life threats that are likely to have been common during this period that are rarely or never identifiable on the skeleton: including, chickenpox, cholera, cowpox, dysentery, fevers, malaria, measles, smallpox, strokes, suicide and typhus (Robb et al. 2021, Table 2). While the list is Table 5 is unlikely to be entirely accurate, it focuses attention on causes of death, a topic that is rarely addressed in human osteology.

## Textual individuals

### By Craig Cessford and Nick Holder

The skeletal remains from the friary represent only a small and biased portion of the individuals buried at the friary. Additionally, many individuals associated with the friary were not buried there. There are, however, several hundred named individuals with textually attested associations with the friary, some of whom belong to groups that are absent or underrepresented in the skeletal population. These individuals can therefore usefully supplement the information provided by the skeletal evidence, (further details are included in the Supplementary Materials Section: textual individuals with links to the Cambridge Austin friars).

Although there are a few friars whose lives can be reconstructed in detail, they are almost all individuals who were not buried in Cambridge. In contrast, most of the textually attested friars that are likely to have been buried in Cambridge comprise little more than a name and a date. One exception to this is the prior John de Comberton, who died 1348–51 and would have been buried in the friary church, which has not been investigated. John was probably born in or near Cambridge in *c*. 1300/10 and would have been a novice at Cambridge, but was ordained a deacon at Ludlow in 1328. By the early 1340s he had returned to Cambridge, and he was prior *c*. 1343–8 dying by 1351 at the latest. If his skeleton had been excavated John would been found in a location indicating his high status and clothed with a surviving girdle buckle identifying him as a friar. Ancient DNA would have identified him as male or consistent with male but not female, and he would have been aged as either a Young Middle Adult (26–35), Old Middle Adult (36–45) or Mature Adult (46+), compared to his documented age of 33 to 51. His isotopes would identify him as probably local, with no evidence of his time spent away from Cambridge, and he would have the enriched diet typical of the friary. His skeleton might also show evidence for injuries or diseases that were not recorded in any surviving document, and given when he died it is possible that he would have tested positive for *Y. pestis* aDNA. It can therefore be seen that there is considerable overlap between the potential skeletal and textual evidence, but that both sources can also provide information that the other source cannot.

Friars who spent some time in Cambridge but were not buried there include the Continental scholar Simon von Brünn (1370s–1433+), who probably spent three years at the Cambridge in 1419−22 while in his forties. Although a number of Continental friars spent time at Cambridge (see Supplementary material section non-English friars with links to the Cambridge Austin friars) few if any died there. This helps in part explain why the stable isotope and aDNA evidence from the Cambridge Austin friars lacks evidence for exotic origins. As Simon lived for several decades after he left Cambridge his skeleton and its stable isotopes would be unlikely to preserve any identifiable traces of his time there. The same is also true of the most famous English friar known to have spent time in Cambridge, the theologian and historian John Capgrave (1393–1464), from King’s Lynn.

Three individuals left surviving wills requesting burial in the Cambridge friary: Thomas Walsyngham (a clerk from Norfolk who lived some distance from the friary in the parish of St John Zachary and died 1410) and Thomas Priour (a wealthy landowner at member of parliament from Essex who does not appear to have ever have lived in Cambridge and died 1413) in the church and William Sengeorge (a student and later fellow at King’s College who lived in a house at the Austin friars in his later years and died *c*. 1514) in the cemetery. There is also evidence that another individual associated with King’s College William Turner was also buried in the cemetery, as his grave is mentioned in a will of 1519. None of these individuals were buried in locations that have been excavated, but they potentially have comparable life histories to some of the adult male lay individuals that have been recovered.

The intriguing burial of a woman in the chapter house *c*. 1500–38 raises the question of the relationship between woman and the friary. Two townswomen who had links to the friary in life, but were probably not buried there, were Katherine Bailey (died 1470s) who was miraculously cured of blindness and Margaret Phylips (died 1524) who bequeathed a cushion to make an altar cloth. While friaries were male institutions they played an important role in the lives of many women, as exemplified by both the skeletal and archaeological evidence.

## Material culture and individuals

The material culture recovered during the excavations has been described in detail in the excavation reports (Cessford 2017, 2020) and will not be repeated in full. Instead, the focus is on how this material relates to individuals at the friary. Clothing, particularly buckles, has already been published (Cessford et al. 2022) but will be summarised, and material that is principally architectural is dealt with elsewhere (Cessford and Samuel in prep). Although most of the material culture associated with the friary presumably relates to the Austin friars, it is possible that some of it relates to lay individuals. This is particularly true of periods when building works were taking place, as significant numbers of outside craftsmen would be present on site and material was often deposited. There are also issues of residuality, with friary foundations and graves disturbing earlier domestic features and redepositing material. Deposits that date to the Dissolution are also problematic, as material relating to the friary may be mixed with items linked to those undertaking the demolition. This is true of the sole large group of material that may be associated with the friary (pit F.728).

In terms of local comparisons, large assemblages of material culture contemporary with the friary have been recovered from a number of domestic properties excavated at the Grand Arcade site immediately to the east (Cessford and Dickens 2019). Investigations at Corpus Christi College to the west have been on a smaller scale, but include collegiate material (Newman 2018). In the same street block, excavations of domestic properties at Hostel Yard revealed a series of sixteenth century assemblages, associated with wealthy burgess household(s) (Cessford 2005).

A few items can be directly linked to the excavated skeletons, these include a number of girdle buckles and a jetton. While a wide range of other items were recovered from grave fills these are all interpreted as incidental inclusions, representing either residual material already present in deposits before graves were dug or the inclusion of contemporary midden material. Whilst linked to specific skeletons, the girdle buckles should be viewed as simultaneously corporate and individual items. Other items while not linked to specific skeletons can nonetheless be linked to individuals, albeit anonymous ones. The clearest example are two glass roundels from windows decorated with initials, as these probably commemorate friary benefactors and represent the initials of their names. The two lathe-turned bone styli would have been writing tools held in the hand and are therefore in a sense personal items. Although not exclusively ecclesiastical they were probably used by friars, whether the styli would have used communally or by specific individuals is uncertain although it is tempting to think of the stylus with a decorated silver sleeve as being used by one person. Other items that are also likely to be used by specific individuals include an iron hammer and a copper alloy forked spacer from a composite strap-end, with an acorn shaped terminal, although these are more likely to relate to lay individuals rather than friars. Most other material such as pottery and glass vessels are likely to have been communal items, although a flower incised on a redware jug after firing could have served to identify it as a personal item. This incised design would also have been created aby a specific individual and the same is true of the lines of a board game such as Nine Men’s Morris incised into a stone from the cloister.

### Girdle buckles and a jetton directly associated with skeletons

Twenty skeletons excavated in 2016−17 had copper alloy, iron, bone and ivory buckles near the pelvis, while a further eight buckles recovered in 1908−9 also appear to have been associated with skeletons (see Figure 11). These are interpreted as girdle buckles that indicate clothed burial of members of the Austin friars (Cessford et al. 2022). Mendicant clothing, including girdles, was distributed to friars for their personal use, so they had a degree of control but did not own the items and they were returned to the house upon their death (Ponesse 2020, 405) apart presumably from any items they were buried in. The buckles are relatively small and simple, in keeping with mendicant ideals of poverty. Although there is some variation in the form and material of the buckles, it appears that they were supplied by the Order rather than being acquired by individuals. This particularly applies to the three commonest types of buckles: D-shaped frame with a rectangular plate (11 examples), oval or D-shaped buckle frames and crescent or double-crescent mounts (four examples) and symmetrical double oval frames (three examples). The frequency of these forms suggests that they were supplied by the Cambridge Austin friars, with the three different forms probably relate to change over time. There were also a number of other buckle forms represented by single buckles, one possible explanation given the mobility of friars is that these come from girdles issued at other friaries to individuals who travelled to Cambridge. A buckle that is unusually made from ivory is likely to have been manufactured in Paris and may have been brought to Cambridge by a friar who studied there in the early fourteenth century.

The buckles predominantly point to the right side of the deceased (16 instances). The single instance of a buckle pointing to the left is associated with an individual who appears to have been left handed, while the single instance of a buckle pointing upwards is associated with an individual who died in a traumatic accident. The two buckles with atypical alignments suggest that the girdles individuals were buried with were probably those they wore in life.

Several buckles from the Cambridge friary have crescent shaped mounts; these are uncommon nationally and the only other site where they appear to be relatively common is the Kingston-Upon-Hull Austin friars (Goodall in prep). More generally there are pronounced commonalities of buckle type between the Cambridge, Kingston-Upon-Hull (Goodall, A. in prep: Goodall, I. in prep) and Leicester (Clay 1981) Austin friars, suggesting a potential degree of shared material culture throughout the Augustinian national province.

Although the girdle buckles were recovered associated with individual skeletons they should be interpreted primarily as corporate objects associated with the Cambridge Austin friars as an institution and the broader Augustinian national province. They are not however exclusively corporate items, and they would simultaneously have been closely associated with specific individuals.

**Figure 17.**
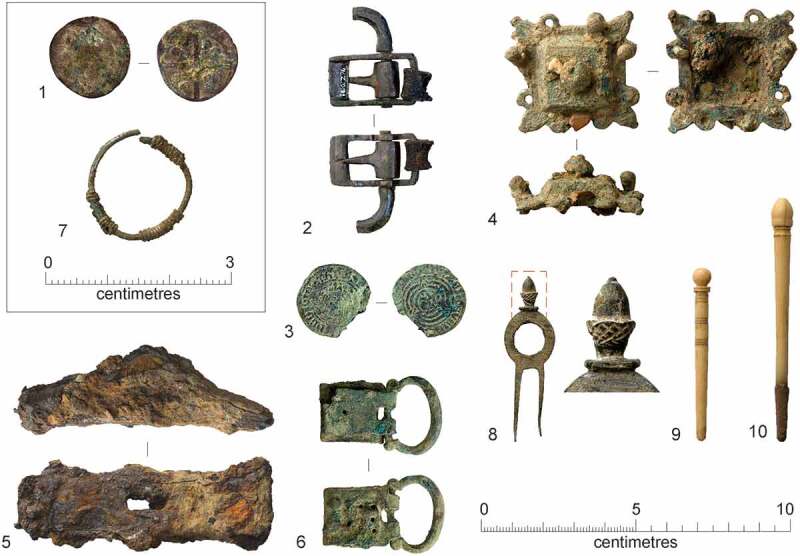
Metalwork and worked bone associated with the Cambridge Austin friars: 1) silver penny of Edward IV (1461−83) from grave fill <2102> [1163] F.146; 2) locking buckle probably associated with purse 1908–9 discovery 1910.274; 3) Nuremberg jetton from grave fill <2024> [1524] F.146; 4) copper alloy book clasp <2021> [1122] F.135; 5) iron socketed hammer head, possibly a ‘pitching-hammer’ <2092> [1960] F.406; 6) oval or D-shaped copper alloy buckle <2000> sf. 100; 7) plain white metal wire hoop, possibly a silver finger ring <2061> [1502] F.255; 8) forked spacer from a composite strap end <267> [3168] F.578; 9) bone styli <876> sf. 110 [1435] F.409; 10) bone styli with copper alloy point enclosed within a silver sleeve <202> [1163] F.146.

A sixteenth-century jetton manufactured in Nuremberg was found next to the lower right tibia of an adult woman (F.146) buried in the chapter house (Figure 17, 3). Although the upper fill of the grave contained a range of material the lower fills near the skeleton were devoid of any other material, so the jetton is unlikely to be an incidental inclusion. The legs of the skeleton showed evidence for a long-lived and severe health condition (see above: the skeletons). In this context the jetton may indicate a form of popular folk magic (cf. Gilchrist 2008), rather than its usual function of being used for calculations on a counting board. The jetton is of the anonymous Rose/Orb type with (large orb) of *c*. 1500−1580s (cf. Mitchiner 1988, 377−81, nos. 1190−1226), The orb surmounted by a cross was a symbol of authority, representing Christ’s dominion over the world, and it is possibly this influenced the choice of this jetton. The woman was apparently buried in a shroud, suggesting that the jetton was added after the body was washed and during the shrouding process, which probably took place at her home and involved members of her family. Less probably it might have been held in place by a bandage or similar item that was not removed after death.

### Ceramic and glass vessels

#### By David Hall† and Vicki Herring

**Figure 18.**
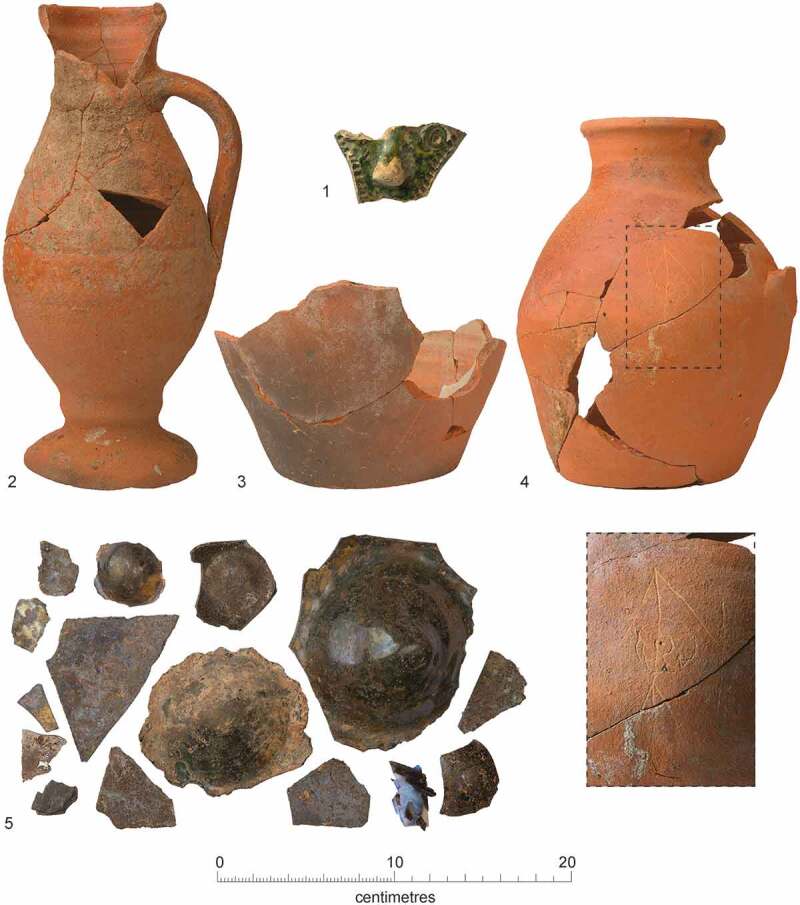
Pottery and glass associated with the Cambridge Austin friars: 1) Grimston ware face jug <787> [1487] F.396; 2–4) redware jugs, one with a post-firing incised flower decoration, from Dissolution assemblage <461> [3555] F.728; 5) fragments of glass vessels from Dissolution assemblage <464> [3555] F.728.

The pottery from deposits associated with the friary is typical of the fabrics and forms found at other sites in Cambridge, with no significant difference to the material from domestic or collegiate sites. Fourteenth–fifteenth-century vessels recovered include a Grimston ware face jug (Figure 18, 1), two Essex Redware jugs, an Ely ware jug and a coarseware bowl in a buff sandy fabric. A possible Dissolution group includes four redware jugs, one with a post-firing incised flower decoration (Figure 18, 2–4), and an iron glazed cup.

Shards from four glass vessels were found; two fourteenth-century English potash goblets and two later fifteenth–sixteenth century clear vessels, which are probably of Venetian and Eastern Mediterranean origin. The goblets are other glassware are paralleled by finds from domestic and collegiate contexts in Cambridge. Glassware does appear to be more common at the friary and college; this can be crudely assessed by comparing the ratio of fourteenth-century goblets to thirteenth–fifteenth-century pottery sherds. The ratios are friary 1:328, college 1:357 and domestic 1:3200. This crudely suggests that glassware was an order of magnitude more common at the friary and college than on domestic sites. This probably changed in the sixteenth century, at least in wealthy burgess households. The probable Dissolution group from the friary included six glass vessels (two beakers/flasks, one small flask/phial, one beaker/goblet, one stemmed or pedestal goblet and one bottle) (Figure 18, 5) compared to five pottery vessels. A backfilled timber cesspit of broadly similar date from the Hostel Yard site contained twenty ceramic vessels and nine glass vessels including two tankards or jugs (one probably of Venetian origin) and one flask.

### Metalwork

#### By Martin Allen, Craig Cessford, Andrew Hall and Justin Wiles

Excluding buckles, relatively small assemblages of items of copper alloy and iron were recovered. Some of these are of interest given their association with the friary. A silver penny of Edward IV (1461−83) was recovered from a grave fill (Figure 17, 1) and a late fifteenth–sixteenth century copper alloy locking buckle with rectangular frame was discovered in 1908–9 (Figure 17, 2). Worn as belt buckles, the arms of locking buckles acted as hooks to suspend a purse from (Williams 2018). Purse frames are known from several friary sites and the purses could have been used to hold collected alms. While Franciscan friars were not supposed to receive coins or money (Franciscan Rule, Chapter 4) this was not the case for the Austin friars. They were allowed a small financial allowance or *peculium* (little pebble) (Roth 1966, vol. I, 187, 216) There was also one English jetton of *c*. 1280–1345 and two sixteenth-century Nuremberg jettons, principally used for calculations on a counting board.

A very fine and decorative fifteenth–sixteenth-century square or diamond shaped mount from a Dissolution context is probably the central boss from a leather book binding (Figure 17, 4). While book mounts of this date are regularly found on both domestic and collegiate sites in Cambridge, the quality of this boss is unparalleled. The friary is known to have had a library, and a collection of books from the friary are held in the Vatican Library (Crook 1983; Ker 1978, 24). They are physically similar to contemporary college collections and are mainly good quality large library books, some of very high quality. They were procured by various means, and some were rebound while they were still at the Cambridge friary.

A heavily corroded fifteenth–sixteenth-century iron key with solid stem projecting beyond the rectangular bit and circular bow was recovered. There were probably many locks and keys associated with the friary. The Cambridge friary rules of 1438 stated that ‘neither student nor conventual [member of the friary] to have a key to the library without knowledge of the librarian. Any holder of such a key must pay the librarian 12d for the repair of books and when leaving [Cambridge] must return the key to the librarian and no one else’ (Roth 1966 vol. II, 321–2, no. 788).

Two iron tools were recovered from friary period deposits: a punch or awl and a socketed hammer head (Figure 17, 5). The hammer is a finely made tool, possibly a ‘pitching-hammer’ used for stone working. Wear through use may have made it too light to be an effective tool, which could be why it was discarded. The hammer comes from deposits associated with the construction of the cloister and is likely to have belonged to a craftsman employed at the friary, rather than a friar.

Apart from the girdle buckles (see above: girdle buckles and a jetton directly associated with skeletons) few other dress accessories were recovered. There were two other copper alloy buckles, which were not particularly similar to those found associated with skeletons (Figure 17, 6). A plain white metal wire hoop of 15 mm diameter, with three neatly spiralled lengths of smaller gauge wire wrapped around it is possibly a sixteenth century silver finger ring (Figure 17, 7). This came from a Dissolution period deposit, so it is impossible to tell if belonged to a friar or to someone involved at the site afterwards. One distinctive find was a fifteenth–sixteenth-century oval copper alloy forked spacer from a composite strap-end, with a terminal in the shape of an acorn (Figure 17, 8). Acorn terminals were common elements on a range of Late Medieval dress accessories and are the most common form of terminal on forked spacers (Cassels 2013, 67–8). The acorn possessed symbolic meanings that may include attempts to express and negotiate female religious identity, or satirise them, and fertility symbolism (Cassels 2013, 173–81, 196). This is unlikely to have been an item of dress worn by a friar and may relate to the presence of laity within the friary. Several Late Medieval horseshoe fragments and horse bones were found.

### Worked bone

#### By Ian Riddler

In addition to the two ivory and bone girdle buckles (see Figure 11) (Cessford et al. 2022) two late twelfth–sixteenth-century lathe-turned bone styli were found. One with a lightly tapered shaft of circular section, decorated with three bands of triple lateral lines, with a double moulding above surmounted by a globular terminal that originally had a bone point is relatively typical of this type of object (Figure 17, 9). The other has a lightly tapered circular sectioned shaft, surmounted with a double moulding and an acorn knop at the terminal (Figure 17, 10), which may share symbolic associations with the forked spacer already mentioned. This example is more unusual, as it has a silver sleeve with a decorative upper edge forming a continuous triangular pattern, enclosing its copper alloy point. Such sheaths are rare, it has been suggested that a less ornate silver-gilt sheath on a stylus from Southwark may have belonged to an aristocratic household or was designed to impress and to underline the gravitas of the person whose business was being transacted (Egan 2005, 123).

Five similar but relatively plain bone styli were found at the domestic Grand Arcade site (Cessford and Dickens 2019, 170), while a finer and more decorated example was found at Corpus Christi College (Newman 2018, 59–60). If they were used as styli this suggests that writing was relatively common, although it is possible that they were also used for other functions

### Stone

#### By Mark Samuel

**Figure 19.**
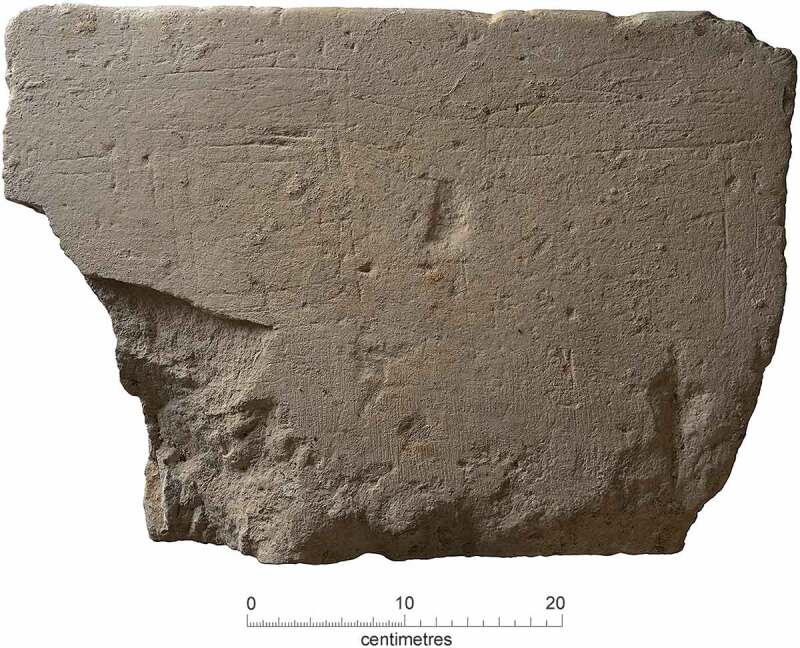
Stone block reused in the fourteenth century, probably in the cloister, where it had a nine men’s morris board scratched into it <905>/<921> [1380] F.202.

Although architectural stone is dealt with elsewhere (Cessford and Samuel in prep), a plain-chamfered plinth block stylistically dated to *c*. 1280–1300 appears to have been reversed and reused, perhaps as a dwarf wall in the cloister in the mid/late fourteenth century. Scratched into the stone are lines for a game such as Nine Men’s Morris (Figure 19), suggesting that the stone was used as a seat. This would have been located either against the cloister wall or in the stylobate of the cloister arcade, where individuals passed the time playing games. Such boards appear to be a common feature of monastic institutions (Hall 2016). A survey of 1544 which values various materials mentions gravestones (TNA, E 318/14/657, m. 5), but no fragments of these were recovered.

### Window glass

#### By Vicki Herring

**Figure 20.**
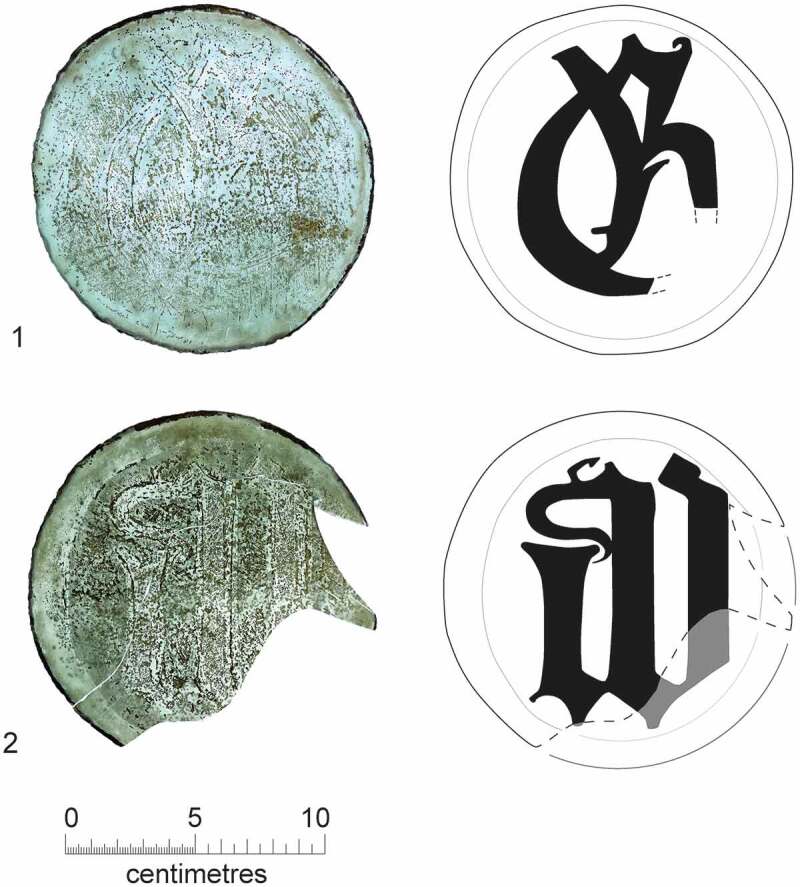
Glass roundels from windows recovered from the Cambridge Austin friars in 1908–9 with Blackletter or Gothic script. 1) probably G or B (G or B) Z 41520.1; 2) M (W) Z 41520.10.

Window glass is dealt with elsewhere (Herring in Cessford and Samuel in prep), but in terms of people it is worth noting two 135 mm diameter fourteenth–fifteenth-century green glass roundels with faint traces of paint. These are letters in Blackletter or Gothic script of *c*. 1150–1590, with a W(M)and either a B (B) or G (G) (Figure 20). These roundels probably commemorate friary benefactors.

### Food

#### By Craig Cessford (textual evidence), Sheila Hamilton-Dyer (fish and bird bone), Vida Rajkovača (faunal remains) and Ellen Simmons (plant remains)

Meat was initially forbidden to members of the Augustinian order, with potential exemptions for the infirm, aged and those doing manual labour, but this prohibition appears to have been rescinded in 1377 (Roth 1966 vol. I, 195). Eggs, cheese and products made from milk were permitted. The friars probably grew fruit, vegetables and herbs in some of the extensive friary gardens and in 1538/9 there was a dovecote in one of the gardens, supplying pigeons, doves and eggs to eat. It is also possible that the friary raised chickens and pigs, as this was common in the town at this time. The friary received cereals as bequests in wills, this included wheat from Balsham and Borough Green and barley from Balsham, Bottisham, Great Shelford and Westwick (Moorman 1952, appendix F). There is no evidence for the presence of fishponds and the friary did not possess any agricultural holdings, so it is likely that much of the food was bought at the nearby marketplace.

Contexts definitely associated with friary were not rich in animal bone. These are dominated by sheep/goat and cattle and the absence of head elements indicates that meat joints or carcase portions were delivered to site. Pig, rabbit, chicken, mallard/domestic duck, domestic goose, cod, pike and common ling are present in smaller amounts. Shellfish are represented primarily by European flat oyster, including *c*. 70 in one thirteenth–fourteenth century pit, and a few blue mussels are also present.

There was one large group that appears to relate to the Dissolution or soon after and may be representative of the food eaten during the latest stage of the friary or by those working at the site after (Table 6). It appears that joints were processed off-site, delivered ‘dressed’ and that no further butchery was carried out on site. The assemblage is almost entirely composed of sheep/goat (58.2%), cow (6.5%), pig (4.9%) and chicken remains including Galliformes (20.3%). Smaller quantities of rabbit, domestic goose, mallard/domestic duck, teal, common crane, domestic pigeon/rock dove, eel, cod, cyprinid, perch were present, as were European flat oysters. The joints from the larger species represent nearly 90 kg of meat and it is likely that the total for the entire assemblage is over 100 kg. The meat is dominated by mutton, particularly leg and shoulder joints, with beef and pork also significant. This could represent around 300 person days of typical Late Medieval meat consumption. In terms of broader patterns, in the fourteenth–fifteenth centuries beef accounted for over half of the meat eaten at sites in and around Cambridge, with the highest rates (67–84%) recorded for colleges, religious institutions and the village of Cherry Hinton (Higbee in Cessford in prep. a). Mutton consumption was highest at domestic and religious sites in the town (23–31%), and twice as much pork was eaten at colleges than elsewhere. At colleges, religious institutions and domestic sites in the town the meat-based diet was mostly mutton, pork and chicken, but in the suburbs and village of Cherry Hinton much of the meat came from older animals past their prime, marking a shift in slaughter patterns linked to changes in livestock husbandry within the rural hinterland.

**Table 6.** Minimum number of butchery units and meat weight calculations for bone and shell from Dissolution or early Post-Dissolution pit F.728.

Species/meat	Joint	Total no.	MNBU	Estimated meat weight (kg)	Total estimated meat weight (kg) by meat type
Cattle/beef	Chuck and blade	2	1	10.9	13.2
	Shin	2	1	2.3	
Sheep/mutton	Leg	29	10	25.0	62.3
	Shoulder	22	11	35.2	
	Scrag	14	1	0.3	
	Loin	6	1	1.0	
	Best end of neck	10	1	0.8	
Pig/pork	Shoulder	2	1	5.2	13.4
	Leg	1	1	8.2	
Rabbit	–	5	2	*c*. 1	
Chicken	–	60	3	*c*. 10	
Domestic goose	–	19	1	*c*. 0.1	
Mallard	–	4	1	*c*. 0.1	
Teal	–	1	1	*c*. 0.1	
Crane	–	1	1	*c*. 0.1	
Domestic pigeon/rock dove	–	1	1	*c*. 0.1	
Eel	–	6	1	*c*. 0.1	
Cod	–	3	1	*c*. 0.1	
Cyprinid	–	4	1	*c*. 0.1	
Perch	–	1	1	*c*. 0.1	
Oysters	–	60+	30+	*c*. 1.0	
Total		120	33	100+	

For cereals free threshing wheat (*Triticum aestivum/turgidum* s.l.) is the predominant crop type, represented as both grains and rachis internodes. A large proportion of the rachis internodes are identifiable as bread wheat (*Triticum aestivum* s.l.), indicating that the free threshing wheat grains are likely to be bread wheat. Other cereal types present are hulled barley (*Hordeum* sp.) with asymmetrical barley grains typical of the lateral spikelets of six row barley (*Hordeum vulgare*), rye (*Secale cereale*) and oat (*Avena* sp.) with grains still contained within floret bases identifiable as cultivated common oat (*Avena sativa*). Pea (*Pisum sativum*) is also present.

The richest wild or weed seed assemblage includes abundant seeds of the typical crop weeds: field gromwell (*Lithospermum arvense*) and stinking camomile (*Anthemis cotula*), some of which was in the form of whole or partial seed heads. Other segetal taxa commonly associated with fertile disturbed soils and cultivation are black bindweed (*Fallopia convolvulus*), chickweed (*Stellaria media*), corn cockle (*Agrostemma githago*) and brome/rye grass (*Bromus* spp./*Lolium* spp.). Also present were seeds of taxa commonly associated with wet or damp soils, such as rushes (*Juncus* spp.), great fen sedge (*Cladium mariscus*) and potentially many of the species of sedges (*Carex* spp.). A smaller assemblage also includes plants of fertile disturbed soils and cultivation such as orache (*Atriplex prostrata/patula*) and stinking camomile along with spike rush (*Eleocharis* sp.), commonly associated with wet soils.

The potential Dissolution assemblage contained hulled barley (*Hordeum* sp.), free threshing wheat (*Triticum aestivum*/*turgidum* s.l.) and oat (*Avena* sp.) grains. A single charred seed and a single uncharred seed of fig (*Ficus carica*) are also present. The small assemblage of wild or weed plant seeds includes segetal taxa, such as fat hen (*Chenopodium album*), orache (*Atriplex prostrata/patula*), corkcockle (*Agrostemma githago*), cornflower (*Centaurea cyanus*) and stinking camomile (*Anthemis cotula*). Also present are the wet or damp soil taxa rushes (*Juncus* spp.), black bog rush (*Schoenus nigricans*) and potentially many of the species of sedges (*Carex* spp.).

The food remains from the Cambridge Austin friars are potentially relevant to both the dietary ideals and rules of the Augustinian order and the stable isotopes from the skeletons. Directly correlating these three elements is however problematic. At the most basic level there is no way to accurately relate the animal, bird and fish bone and the cereals recovered archaeologically to their overall dietary contribution. While the stable isotopes suggest greater levels of marine fish consumption and these could relate to religious dietary rules, recovery issues due to the relatively small size of fish bones mean that there is no way to accurately compare the results from the Cambridge Austin friars to domestic sites in the town.

### Fuel

#### By Ellen Simmons

**Figure 21.**
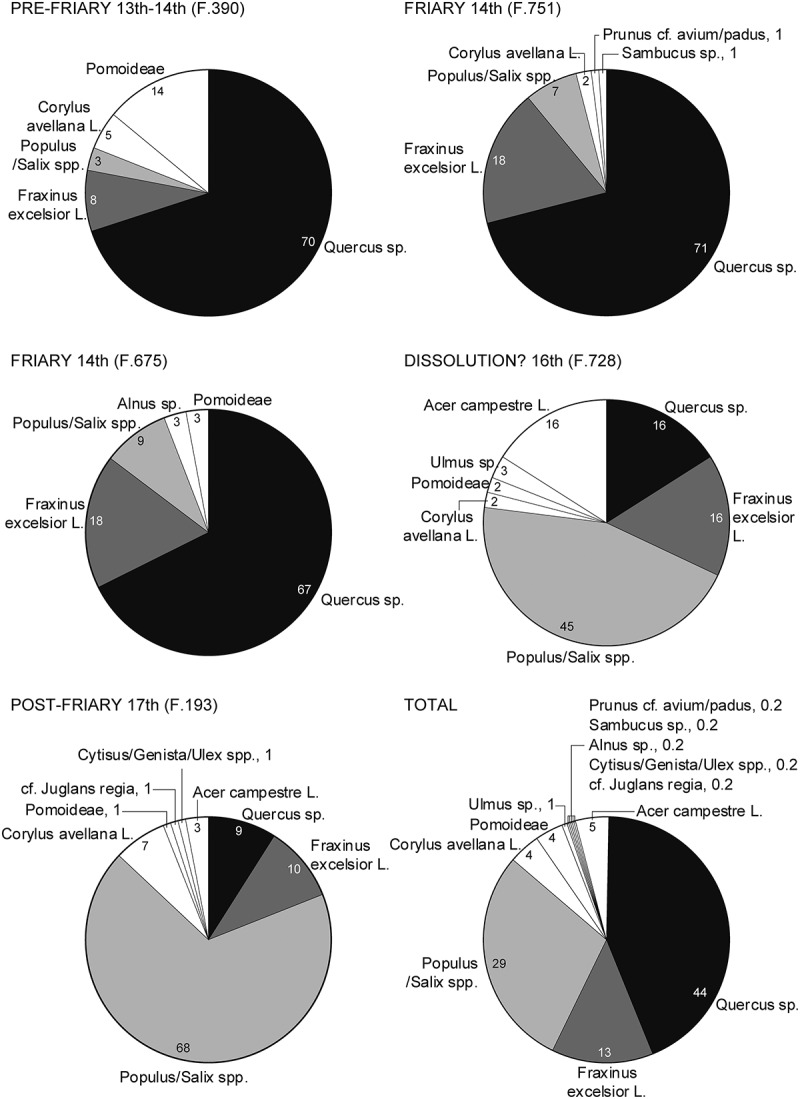
Wood species of charcoal from samples from the Cambridge Austin friars site.

There is no evidence that either peat or coal were used as fuels, although the possibility cannot be entirely excluded. Wood is likely to have been the main fuel and charcoal analysis indicates the types used (Figure 21). One friary period sample produced a relatively diverse range of taxa, including wild/bird cherry (*Prunus padus/avium*), oak (*Quercus* sp.), hazel (*Corylus avellana*), poplar/willow (*Populus/Salix*) and ash (*Fraxinus excelsior*). Weak ring curvature and tyloses were observed on a number of the oak fragments, indicating the use of mature trunk wood. Strong and intermediate ring curvature was observed on wild/bird cherry, ash and hazel fragments, indicating the use of intermediate and smaller branches or twigs. A second friary period sample is composed of predominantly ring porous taxa morphologically similar to either oak (cf. *Quercus* sp.) or ash (cf. *Fraxinus excelsior*).

The rich Dissolution assemblage indicates the presence of a diverse range of taxa, with elm (*Ulmus* sp.), oak (*Quercus* sp.), hazel (*Corylus avellana*), poplar/willow (*Populus/Salix*), field maple (*Acer campestre*) and ash (*Fraxinus excelsior*). Weak ring curvature and tyloses were observed on two of the oak charcoal fragments, indicating the use of mature trunk wood. Weak, intermediate and strong ring curvatures were noted on various taxa, indicating the use of a mix of smaller branches or twigs, intermediate sized branches and larger branches or trunk wood. Reaction wood was noted in the vessel cavities of one of the poplar/willow fragments, indicating the use of branch wood.

## Poverty, mobility, internationalism and urbanness

The defining aspects of the mendicant lifestyle were the commitment to poverty and begging, the mobility of its members combined with the international nature of the orders and the urban locations of most friaries. Definitions of poverty varied between the mendicant orders and over time. The Franciscans espoused an austere version of this, which began as the doctrine of the Absolute Poverty of Christ based on the idea that Christ and the apostles had no individuals or shared property (Lambert 1961). This is reflected in the idea that ‘The brothers shall appropriate nothing to themselves, neither a house nor place nor anything. And as pilgrims and strangers in this world … let them go confidently in quest of alms’ (Rule of St Francis Chapter 6). Although declared heretical in 1323, a Papal bull of 1330 maintained the principle of absolute poverty for the individual friar and the community; but granted them the necessary use of movable objects. There were particular issues with coins and money, and they were not supposed to receive them (Rule of St Francis Chapter 4). They were also encouraged to fast, forbidden to wear shoes or ride horses unless compelled through necessity or infirmity (Rule of St Francis Chapters 2–3). Following the example of Francis, instead of belts they fastened their clothing with simple white ropes or cords known as *cinctures* with three knots symbolising poverty, chastity and obedience. Augustinian poverty was less austere than that of the Franciscans. Although most property was held communally, they were allowed some private personal funds known as a *peculimum* (Gutiérrez 1984, vol. I, 95–6).

In terms of the discoveries from the Cambridge Austin friars, there are a number of items that can be related to the idea of mendicant poverty (architectural aspects of this are dealt with elsewhere (see Cessford and Samuel in prep). The presence of girdle buckles contrasts with the Franciscans, whose *cinctures* lacked any buckles. Clothed Franciscan burials should therefore lack girdle buckles, which appears to be confirmed at excavated Franciscan friaries in England (Gilchrist and Sloane 2005b: O’Sullivan 2013). The buckles from the Cambridge Austin friars are relatively plain and the majority are of a restricted range of types which would fit with ideas of poverty (Cessford et al. 2022). The ivory and bone girdle buckles are unusual items, that were presumably relatively expensive as they were individually produced rather than their copper alloy counterparts which were mass produced in moulds. These buckles can be viewed as ostentatious, albeit in a relatively subtle manner.

The item that most challenges ideas of poverty is the lathe-turned bone stylus with a silver sleeve. Such sheaths are rare, expensive, functionally superfluous and difficult to reconcile with even a relaxed definition of poverty. While it is possible that the stylus belonged to a member of the laity this is not particularly likely, it could however have been a gift. There is also evidence for the presence of coins at the friary and a locking buckle probably associated with purse, these may be linked to the friars begging for alms and the *peculimum* plus the fact that they had to engage with the local market economy, buying produce on the open Cambridge market. The diet of the friars, which could challenge ideas of poverty is considered below.

Several horseshoe fragments and horse bones were found and there is evidence from the skeletons that may indicate horse riding (see above: the skeletons). Franciscan friars were forbidden by their rule to ride on horseback, unless compelled by manifest necessity or infirmity (Rule of St Francis Chapter 3). While most Austin friars probably travelled on foot there was nothing forbidding them to ride, although the rules stated that they were not to enter a town on horseback. In 1356 there is a reference to the friars’ servants using a cart and three horses to fetch food plus stone and timber (Cal. Pat. 1354–8, 439). The 1544 survey of the friary includes six stables that were being leased (TNA, E 318/14/657, m. 5), these probably refer to stabling space in one or two stables rather than six separate buildings. It is unlikely that these stables were developed solely for leasing, suggesting that the friary also made use of them.

Although the Augustinian order was an international organisation and there is textual evidence for friars from the continent studying at the Cambridge friary, the archaeological evidence points to a largely local institution. In terms of both architecture (Cessford and Samuel in prep) and material culture the overwhelming bulk of the evidence speaks of local connections. In part this is unsurprising, the Cambridge friary functioned as both an ordinary friary and a broader educational institution. The majority of those studying there would have come from the local region, and it should be recognised that the surviving texts are more likely to be concerned with long distance connections that required formal permission. The stable isotopes and aDNA evidence both indicate that both the members of Austin friars and laity are no more ‘exotic’ than the groups from other High/Late Medieval Cambridge. This should not, however, be taken as evidence of a lack of mobility. While texts provide considerable evidence for mobility, this can be characterised as friars often having a ‘boomerang’ lifecourse. While individuals may have travelled considerable distances as friars, they often returned to close to where they originated. This would apply to John de Comberton (Comberton/Cambridge – Shropshire – Cambridge) and John Capgrave (King’s Lynn – Norwich – London – Cambridge – King’s Lynn – Rome – King’s Lynn – Winchester – Oxford – King’s Lynn) (see Supplementary Material: textual individuals with links to the Cambridge Austin friars). Given what is known of their lives, the teeth and bones of Comberton and Capgrave would produce isotopically ‘local’ signals that would not hint of their time spent elsewhere.

While the Cambridge Austin friars is undeniably in an urban location there is relatively little in the archaeological remains that directly informs upon this. Compared to material from other local sites the animal bone appears distinctively urban in character (Higbee in prep), with evidence that meat joints or carcase portions were delivered to the friary. Similarly, the wood used as fuel may well have differed from that used in rural contexts, but a lack of comparable analysed material prevents certainty.

## Anti-fraternal criticisms

Although mendicant ideals and practices provide one potential perspective to view the archaeological evidence of the Cambridge Austin friars from, these were not universally accepted in medieval society and other views can provide a useful alternative. The rise of the mendicant orders rapidly produced an anti-fraternal or anti-mendicant reaction among sections of both other religious groups and the lay population (Brim 1990; Geltner 2012). Some of these opposed the mendicant orders on principal, while others objected to what they perceived to be poor behaviour. Although this can be traced back to mid-thirteenth century Paris, a distinctively English version can be identified from the mid-fourteenth century onwards with the *Defensio curatorum* (1357) of Richard FitzRalph (*c*. 1300–60) (Trevisa 1925). This criticism was developed throughout the Late Medieval period, anti-mendicant authors criticised friars both on general theological grounds and more specifically that in practice they did not behave well. FitzRalph and later authors argued that the parish church was the correct place for confession and burial rather than friaries and that friaries diverted money that should go to the parish. There was also criticism focused on learning, burial, buildings, books, clothing, diet and relationships to women and children, many of which are particularly relevant to the Cambridge friary. The nightstick fracture on a friar may be linked to anti-fraternal violence, a particularly extreme form of ‘criticism’, although just because an individual is a friar does not mean this was necessarily a factor in a specific incident.

Perhaps the most directly relevant criticism was that friars encouraged individuals to be buried in their friaries rather than parish churches, but were only interested in attracting individuals who could financially benefit them. As the critic John Gower (*c*. 1330–1408) wrote, ‘both life and death bring money to them’ (Stockton 1962, 182). While this charge could be laid against all monastic orders, the combination of the mendicants espousal of poverty and their institutional wealth gave extra weight to such charges. There is clear evidence that members of the laity were buried at the Cambridge Austin friars. The stable isotope evidence indicates that these individuals all had relatively enriched childhood and adult diets compared to values for High/Late Medieval Cambridge as a whole, potentially supporting anti-mendicant criticisms that friars were only interested in relatively wealthy individuals.

While much anti-fraternal criticism focussed on buildings, this is considered elsewhere (see Cessford and Samuel in prep). One semi-architectural element that is relevant in a personal context are the glass roundels with letters. These roundels probably commemorate friary benefactors, and decorated windows where an image of the benefactor or their name were displayed were a particular target of British anti-fraternal ire. This includes *The Vision of Piers Ploughman* of *c*. 1370–90 and the late fourteenth century *Pierce the Ploughman’s Crede*. In the former a Franciscan friar tells Lady Mede that: ‘“We have a window in the works, already way over budget, if you would like to glaze the gable and engrave your name there, your soul shall surely ascend celestially” “Oh if I knew that”, said the woman, “there wouldn’t be a window or an altar that I wouldn’t make or mend and adorn with my name, so that everyone would see I am a sister of your house”’ (Calabrese 2020, 33).

Friars are also accused of having an insatiable desire for learning and the academic title of master, this was viewed as leading them to neglect their other duties and became a British anti-fraternal commonplace. This is obviously generally relevant to the presence of large Austin friars in Cambridge. More specifically it was a common complaint that friars bought too many books, particularly those of high quality ‘For the beste book in oure hous, theigh brent gold were the leves’ in *The Vision of Piers Ploughman* (verses 264–7). This was perceived as denying these books to other groups, such as members of the university. Surviving volumes from the Cambridge Austin friars in the Vatican Library are mainly good quality large books, some of very high quality, and the copper alloy mount that is probably the central boss from a leather book binding is of particularly high quality compared to other local examples.

Both the Rule of St Augustine and Ratisbon Constitutions deal with food in some detail. Moderation and fasting were encouraged, and the Constitutions stress the poor quality of the food and the exhausting fasts. In contrast British anti-fraternal texts frequently portray friars as gluttons, with Chaucer’s summoner in the Canterbury Tales (1380s) stating that friars and flies eagerly swarm around food and False Seeming in *The Romaunt of the Rose*, translated into English in the 1360s, states that friars always seek ‘delicious morsels of food’ (de Lorris and de Meun 1971, 11,037–47). The δ^15^N values for the friars are higher than typical for High/Late Medieval Cambridge, which probably relates to greater levels of marine fish consumption, supported by the recovery of bones of cod and common ling. While anti-fraternal authors might see this as evidence of gluttony, the consumption of fish especially on days when meat was forbidden would have been viewed by others as a sign of piety.

Friars as confessors visited women in their chambers, leading to anti-fraternal sexual innuendo and allegations of lewd behaviour. By seducing married women friars steal property and ‘plunders [men’s] prerogative over women’ (Stockton 1962, 186) and by usurping a husband’s role and filling the ‘paternal halls’ with ‘growing progeny’ disrupts and destroys the family’s bloodline. Both Chaucer and Wyclif allege that friars were like peddlers and carry knives, pins and other small goods to barter for sex ‘for women … to gete love of hem, and to have many giftis for little good or nought’ (Benson, L. 1987, 233–4). While linking knives and pins from the friary site to inappropriate sexual behaviour is impossible, the burial of women in the cloister and particularly the chapter house could suggest close personal relationships. Any impropriety would, however, be archaeologically invisible and would have been disputed by the mendicants and their supporters.

Various fourteenth-century authors accused mendicants of influencing and ‘stealing’ young children. Gower alleged that they used bribery and entreaty to entice ‘thoughtless boys, who do not possess mature judgment, into taking the vows of their order’ (Stockton 1962, 189). FitzRalph even contends that friars’ theft of children impacted enrolment at universities. Prior to the Black Death novices had to be aged at least 14 when recruited into the order, afterwards this was reduced to 12 and subsequently 11 by the late fourteenth century, with suggestions that it could occasionally have been even younger afterwards (Roth 1966 vol. I, 136–7). One burial in the chapter house (F.230) radiocarbon dated to 1475−1536 was a boy who received clothed burial and is therefore likely to have been a member of Augustinian order who died aged only 11−12. The boy suffered from a serious medical condition, with extraordinarily large lesions in the skull, left arm, right collar bone, left tibia and right femur, but almost certainly died of plague as they tested positive for *Y. pestis*. Medieval anti-mendicants might view F.230 as evidence of the Cambridge Austin friars ‘stealing’ children and diverting them from the University.

While the anti-fraternal literary tradition represents one strand of thought in Late Medieval England it is unclear how widespread this was, and there is countervailing evidence such as gifts in wills to friaries, including the Cambridge Austin friars. All the phenomena that can be interpreted in anti-fraternal terms could also be viewed as perfectly acceptable practices by mendicants and their supporters. It is therefore important to consider both viewpoints.

## Conclusion

The skeletons of both friars and members of the laity buried at the Cambridge Austin friars *c*. 1279/89−1538 provide considerable insight into how these individuals lived and died. This can be supplemented by documentary and artefactual evidence, occasionally linked directly to specific skeletons but also providing information on individuals and groups absent from the recovered skeletons. The three types of evidence largely relate to different individuals that form overlapping communities, which ultimately only represent a minority of the group that originally had a meaningful connection to the friary. All the investigated groups of burials are mixed, with both men and women, a wide range of ages and both clothed and shrouded burials. The fact that clothed burial appears to distinguish members of the Austin friars from laity is particularly significant, as it means that the two groups can in some respects be considered as separate populations. It is also important to remember that burial at friaries occurred contemporaneously in several locations and that where individuals were buried in a friary was based on a range of factors. As a result, simplistic comparisons are inappropriate. It is also important to remember that characteristics are not immutable. For example, two individuals of identical calendar age buried in the early cemetery and the chapter house would be at different stages of their Austin friars lifecourse, due to the changing age at which one could become a novice. These skeletons and the other remains can be interpreted both in terms of mendicant ideals and anti-fraternal criticisms.

In many respects the skeletons from the friary, both Austin friars and laity, are remarkably similar to those from other contemporary burial grounds in Cambridge. Geometric morphometrics indicate a lack of major lifestyle differences between those buried in the friary, parish and hospital, suggesting broadly similar levels of physical activity. While other aspects such as stable isotopes linked to diet were unusual they still fall within the general range of other burial groups.

The burials from the cemetery are in a sense unproblematic, although by no means representative or unbiased, as this was a relatively common location for burial where a wide range of individuals were interred. In contrast burial in the chapter house was a much less common option and the heterogenous group interred there are unlikely to be particularly representative of the overall population buried at the friary during this period.

Future research on burials from friaries and other medieval religious institutions needs to fully address the related issues that those interred typically include both members of religious orders and the laity and that burial took place in multiple locations with varying proportions of different social groups buried in the chapel, cloister walk and garth, chapter house, ancillary chapels and cemeteries. Attempting to distinguish members of religious orders from the laity can be difficult, and the distinction between clothed/religious and shrouded/lay burials found at the Cambridge Austin friars does not appear to be replicated elsewhere, but needs to be more thoroughly attempted. By comparing friary populations to contemporary groups from other burial grounds in a town it should be possible to consider this statistically at a population level, even if the status of individuals remains ambiguous. Similarly analysis needs to take more account of burial location as a factor.

## Supplementary Material

Supplemental Material
