# Supplementary material for: The people of the Cambridge Austin friars
Source: Archaeol J (Lond). 2022 Sep 26;179(2):383–444. doi: 10.1080/00665983.2022.2090675 (PMC9580237; doi:10.1080/00665983.2022.2090675)
Supplement: Supplemental Material [file RAIJ_A_2090675_SM0285.zip › Supplementary text and figures/Supplementary text.docx]

The People of the Cambridge Austin friars Supplementary Material

Craig Cessford and Benjamin Neil

With contributions by Craig Alexander, Martin Allen, Esther Cameron, Andy Hall, Nick Holder, Quita Mould, Ian Riddler, Christiana L. Scheib and Justin Wiles

The People of the Cambridge Austin friars Supplementary Material

# Textual individuals with links to the Cambridge Austin friars

By Craig Cessford and Nick Holder

Details linked to the Austin friars are principally from Roth (1966). Details of individuals associated with the University are largely derived from *ACAD: A Cambridge Alumni Database* (<https://venn.lib.cam.ac.uk/>), which incorporates the volumes of *Alumni Cantabrigienses* (Venn and Venn 1922a; 1922b; 1924; 1927), *A Biographical Register of the University of Cambridge to 1500* (Emden 1963) and other sources.

## John de Comberton, prior (c. 1300/10–48/51)

John de Comberton or one of his forefathers presumably come from the village of the same name, about 7km southwest of Cambridge. This was a moderately sized village with 50 properties in 1279/80 and 57 inhabitants assessed for tax in 1327. A Robert de Cumberton/Comberton granted a messuage to the friary in 1335 (Roth 1966 vol II, 121 no. 299). Robert is mentioned in Cambridge documents of 1299–1347 and is described as a baker and bailiff (1300/1; GBR/0268/CCCC09/18/15). John de Comberton may well be the son of Robert, although there is no actual proof this type of familial background would be typical for a friar. John de Comberton is first mentioned on 24 December 1328, when he was an Austin friar at Ludlow, Shropshire, *c.* 190km west of Cambridge, and was ordained a deacon by the bishop of Hereford (Capes 1912, 103). The Ludlow friary had been founded in 1254 and appears to have been thriving at this time (Faraday 1991, 60). The diaconate was a stage to becoming a priest and John had presumably already been a novice at Cambridge and undertaken his initial studies and first degree within the Cambridge limit. This indicates that he was in his mid-twenties and had been born just after the start of the fourteenth century. By the early 1340s he had returned to Cambridge, although as he was a reasonably senior figure by then he had probably been there for some time. John was appointed one of seven *limiters*, licensed to preach, hear confessions and beg within a defined area within the overall territory associated with the friary. By March 1343 he was prior of the Cambridge friary and was appointed a *penitentiary* and permitted to administer penance (Roth 1966 vol II, 141 no. 340). This was renewed in July 1348 (Roth 1966 vol II, 174 no. 397). By 1351 there was a new prior, John Tulyet (Roth 1966 vol II, 174 no. 397), suggesting that John de Comberton had died in his late forties in the intervening period, quite possibly during the Black Death in 1349.

## Thomas Walsyngham, clerk (Mid/late fourteenth century−1410)

Thomas Walsyngham, a clerk, was probably born in in north Norfolk, *c*. 90km northeast of Cambridge. In 1409 he leased a tenement in Milne Street and drew up a document dated 1 November 1410, indicating that he was still active (GBR/0268/CCCC09/16 C/99, GBR/0268/CCCC09/16 C/101). He wrote his will soon after on 5 November 1410 and he died later that month and the will was granted probate on 6 December (TNA, PROB 11/2A/366). Thomas’ will suggests that he had a close relationship with the Cambridge friary during his life, presumably attending preaching or going to confession there. He requested burial in the choir of the church of the Austin Friars and paid for a dole to poor people attending his vigil and funeral (40s). He left money to the Austin Friars (50s 4d), the four friaries of Cambridge (10s), St John Zachary for any forgotten tithes (20s) and Holy Trinity Cambridge (53s 4d). He also left bequests to the Augustinian priory at Walsingham and Creake Abbey, which are less than 10km apart in north Norfolk, presumably reflecting his origins. His bequest to St John Zachary reveals his Cambridge parish, which included part of Milne Street. One possibility is that Thomas was a priest at the church.

## Thomas Prior, landowner (1355−1413)

Thomas Prior probably came from Hatfield Regis, now Hatfield Broad Oak, in Essex *c*. 45km south-southeast of Cambridge. He may have been one of two sons, both named Thomas, of Thomas and Isabel Prior mentioned in 1355 (Roskell, Clark and Rawcliffe 1993, vol.4, 142–3). Thomas married Joan the daughter of Sir Edmund Vauncy of Westley Waterless, Cambridgeshire, before 1390 and in the same year was removed as *verderer* [a judicial officer] of the royal forest of Hatfield, as he was ‘too much busied elsewhere’. Joan inherited the manors of Westley Waterless and Lockleys in Welwyn, Hertfordshire, in 1390 on the death of her brother. In 1397 Thomas acted as a *feoffee* [trustee who held an estate in land for the use of a beneficial owner] of Saddlebow manor, Norfolk, and witnessed a deed relating to lands in Cambridgeshire. In 1398 he was still based in Essex, but by 1401 was living at Westley Waterless, when he obtained a licence to have a private oratory or chapel for private worship there. He was Member of Parliament for Cambridgeshire in 1402 and soon after was sued by two creditors from Lincolnshire. In 1403 he travelled to Picardy, probably on royal business. In 1404 he was tax controller for Cambridgeshire and in 1404 Joan became coheir of estates in Huntingdonshire at Great Gidding, Great Raveley, Luddington and Sawtry. The first evidence for a close connection to Cambridge dates to 1405, when Thomas held land at Impington just north of Cambridge with hunting rights. Then in 1407 he attended the elections for Parliament held in the county court at Cambridge. Thomas acted as trustee for several local gentry estates, suggesting that he was accepted into the community of the shire. In 1411 he was convicted and fined for breaking into the royal park at Little Hallingbury, Essex, and hunting and stealing a sorel (a male fallow deer in its third year) and a doe, and on a different occasion taking two fawns.

Priour made his will at Great Raveley on 14 September 1413. He had died by 18 October when probate began, and this was completed on 24 November (TNA, PROB 11/2A/420) and was possibly in his seventies when he died. He asked to be buried in the conventual church of the Austin friars at Cambridge. At that time, his manor of Westley Waterless was valued at £6 13s.4d. a year, while his holdings in Huntingdonshire and Essex were worth £43 a year. Among his bequests were sums of money, vestments and service books which he left to the churches of Hatfield [6s 4d, his breviary and a green cloak] and Westley Waterless [20s], to the Benedictines at Hatfield Regis Priory [£1 3s 4d], and to the prior of Anglesey [a gold signet ring, probably to John the prior of Anglesey Abbey documented in 1411]. His house at Hatfield was to be sold after his widow’s death, with the proceeds to pay for masses for their souls. The widow was also to keep for life her share of all his goods, and silver plate and received his half-share in land in Westley Waterless, which mentions cornfields, plough-teams, horses and oxen. Priour’s daughter, Elizabeth, and a kinsman Henry each received £20, while others including the priest John Seveney and a John Lysel were to receive cloaks. His wife Joan rapidly remarried to John Hore of Childerley, whom had been an overseer of Thomas’s will.

There is no known connection between Thomas Prior and the Cambridge Austin friars prior to his death. He came Hatfield Regis in Essex and in 1389 the Cambridge and Clare friaries were in dispute about whose begging limit this village fell within (Harper-Bill 1991, 83, no. 138). Moving to Westley Waterless *c*. 1398–1401 brought him closer to Cambridge but is still 17km distant. It is only in 1405/07 that there is evidence for close contacts with Cambridge, possibly beginning with his interest in hunting.

## Simon von Brünn, friar (1370s–1433+)

Simon von Brünn (Brno, Czech Republic, over 1200km east-southeast of Cambridge) must have been born in the 1370s and was licensed to hear confessions in 1400 and 1402. In 1409 he was appointed *vicarius generalis* for the provincial chapter of Bavaria. In August 1419 in his forties, he was sent to be a student in Cambridge (Roth 1966 vol II, 288 no. 713). It was probably here that he copied *The Art of Preaching* by the Irish Austin friar Geoffrey Shale, who obtained his master’s degree at Cambridge, which he took back with him to the continent. He returned to Brno acting as *vicarius generalisagain* for Bavaria in 1425 and was probably in Brno when the friary was severely damaged during the Hussite attack of 1428. In 1433 he was provincial lector in Vienna (Austria) and in 1443 he was a lector dealing with a case in Breslau (Wrocław, Poland). Simon probably spent three years studying in Cambridge in 1419−22 while in his forties, only a small part of his life considering he lived into at least his sixties. Like most Continental, and indeed English, friars he passed through Cambridge.

## John Capgrave, friar (1393–1464)

John Capgrave (21 April 1393–12 August 1464) the theologian and historian himself stated ‘My cuntre is northfolke, of the town of lynne’ (King’s Lynn, then known as Bishop’s Lynn *c.* 65km north-northeast of Cambridge) (de Meijer 1955; Fredeman, 1979; Winstead 2007). Of his youth he later recalled the capture of some Scottish fishing boats by men of Lynn (1400), a comet (1401), Princess Phillipa leaving for Denmark from Lunn (1404), a severe winter (1407), floods (1413) and beggars stealing three children (1416). There may have been a family link to the Austin friars, as another John Capgrave who was supposedly his uncle was a friar at Oxford in 1390. John Capgrave joined the Austin friars at Kings Lynn *c*. 1403–10 and probably spent some time studying at the Norwich friary, perhaps *c*. 1412–5 as he mentions a fire there in 1414. He was ordained in 1416/7 aged 24. He then studied Theology in London 1417–20 and on 8 April 1421 was promoted from cursor to lector. He was then appointed to study Theology in Cambridge on 13 April 1422, undertaking his opponency in 1422 and was promoted to the baccalaureate on 20 March 1423. He probably incepted (graduated) in 1425 and remained at Cambridge as a regent until at least 1427. It is unclear where Capgrave was after this until he travelled to Woodstock in 1439. He had probably returned to King’s Lynn by 1440 if not earlier, as he dedicated a work written then to the Premonstratensian abbot of nearby West Dereham. Capgrave mentions in his *Liber de Illustribus Henricis* that he visited Eton (1440) and Cambridge when the foundation stone of King's College was laid (1441). By 1 August 1446 he was prior of the King’s Lynn friary, when he showed the king around the friary. He visited Rome in 1447–52 (probably 1449–50) over 1500 km from King’s Lynn, probably travelling via Austin friars such as Paris and Lecetto. He was elected prior provincial of the English order at Winchester in 1453. He served in this role until 1457, which probably involved him in some travel including to Oxford in 1456. He died at the King’s Lynn friary in 1464 aged 71 and was buried there.

Although religiously orthodox Capgrave supported religious reform and was liberal thinker and innovator. He was interested in good governance, lay spirituality, the definition of orthodoxy and the status of women. Capgrave addressed a verse life (*c*. 1445) of one of the most popular saints of the time, Katherine of Alexandria. Although it has been argued that Capgrave disapproves of devotional imager it is now thought that he supported their appropriate use and distinguished this from pagan veneration (Gayk 2010; McCoy 2012). In his life of Katherine of Alexandria, Capgrave uses wheel imagery several times in a way that is deliberately linked to visual imagery (McCoy 2012), such as the floor tile recovered from the Cambridge friary (see above). Like Simon von Brünn, Capgrave passed through Cambridge. Although Capgrave led a mobile existence as a friar, he ultimately ended up at the friary where he initially served his novitiate.

## Katherine Bailey, worshiper (early/mid fifteenth−late fifteenth century)

Henry VI (reigned 1422−61 and 1470−1) was informally regarded as a saint and martyr after his death until the early sixteenth century and various miracles were attributed to him. One of these, which was investigated but not verified, relates to Katherine Bailey, a Cambridge woman from St Edward’s parish who had been blind in her left eye for seven years (Knox and Leslie 1923, 135−6, no. 99). Katherine heard mass at the Austin friary every day at dawn. One day whilst kneeling at mass a mysterious stranger told her to bend a coin for King Henry. Even though she had no money with her, she decided to do so and was cured. As far as can be determined the story appears to relate to a literal rather than metaphorical case of blindness and is presented as such. Katherine appears to have been married to John Bailey, who is mentioned in a document relating to the parish of St Andrew the Great (1465) (GBR/0268/CCCC09/10/33) and had a tenement in St Edward’s parish (1470 and 1477) (GBR/0268/CCCC09/09/33, GBR/0268/CCCC09/09/36). The bending of a silver coin was closely associated with the cult of Henry VI, more broadly coin bending occurs in thirteenth to late fifteenth century miracle stories and this is supported by archaeological evidence indicating links to saintly shrines, foundation deposits, agricultural petitions and communal identity (Kelleher 2018).

## William Turner, cleric (c. 1450−early sixteenth century)

A Cambridge clerk called John Nede drew up his will in November 1519 (KCC, ledger book, i, f. 249). He requested burial in King’s College by the grave of Stephen Woode, however he also mentions an alternative burial option at the Austin friars by William Turnar. This was probably William Turner born *c*. 1450 who undertook his first degree at Cambridge in 1465−8/9 and then a Bachelor of Theology in 1486/7−90 (Emden 1963, 599; Venn and Venn 1927, 278). He had ecclesiastical posts at churches in Little Rayne (Essex, 1470), Ealing (Middlesex, 1478), Stanford le Hope (Essex, 1488), St Mary Staining (London, 1498) and Mount Bures (Essex, 1499). William also gifted 12 books to the University library and a chest to the Schools 1493/4.

## William Sengeorge, scholar (c. 1480−1518)

William Sengeorge or St George was born *c.* 1480, attended Eton and was admitted to King’s College gaining his initial degree in 1500 and his Master of Arts in 1504 (Emden 1983, 502; Venn and Venn 1927, 4). He was a fellow until at least 1507 and was later the college ‘receiver’, possibly dealing with its estates. He drew up his will in 1514 and may have died in 1518 (CUL, Vice-Chancellor’s Ct, will register, i, ff. 29v–30v). By 1514 he was living in a rented house within the Austin friars as his executors had authority ‘to sell the years of my house [the remaining lease] within the Austin Friars’. He wanted to be buried at the friary ‘without the church door before our Lady of Pity’, presumably in the cemetery beside a chapel with that dedication containing a pietà or sculpture of the Virgin Mary cradling the dead body of Jesus. William left detailed funeral and post-mortem mass arrangements, including 4d for every friar who sings dirige and mass on day of burial, a trentall of masses on the following day, doles to the poor and further details for a month's mind (a second funeral service a month after burial). There was also a separate payment to King’s College for chantry masses for two years (53s 4d). Further bequests for separate trentalls of masses for Thomas Asthwell and wife, Harry Vesey, Richard Sengeorge (possibly his father), Richard Tanfeld[?] and others. He also left money to the Austin Friars (10s) and Grey Friars (10s). He bequeathed a silver pot to his mother, and sums of money to clerks or academics including master William Wylton (£1) and his executors master Robert Nole and master Holbys (£1 6s 8d each).

## Margaret Phylips, worshipper (late fifteenth/early sixteenth century−1524)

In 1524 Margaret Phylips requested burial in St Bene’t’s churchyard, indicating that she lived in this parish adjacent to the friary (CUL, Vice-Chancellor’s Ct, will register, i, f. 37v). She bequeathed St Bene’t’s 12d and the Austin Friars 3s. She left her kirtles (gowns) to ‘my kyndswomen of Carlton’, suggesting that she came from this village 22km southeast of Cambridge. As well as money she left the friary a cushion to make an altar cloth for the chapel of Our Lady of Pity (see above), presumably a friary chapel she prayed in, or attended confession at, during her life. Her executors include her brother-in-law Nicholas Symons, possibly the same as Nicholas Symond a goldsmith who leased property in Cambridge in 1507 (GBR/0268/CCCC09/34C/22, GBR/0268/CCCC09/09/40).

# Non-English friars with links to the Cambridge Austin friars

By Craig Cessford

The list is derived from Roth (1966) and is arranged in chronological order.

1358 Galvanus of Padua [Italy] and Gerard Aymerici [later linked to the province of Provence, southeast France], followed by Raphael de Lucca [Italy] and Louis de Valle Speciosa [Schönthal, Bavaria, Germany] to read the sentences at Cambridge. Galvanus is know to have returned to Italy and Gerard to Provence. Louis appears not to have gone to Cambridge. Roth 1966 vol. II, 185 no. 436.

1359 Fr Mathew de Podiolo [Reggiolo, Italy] to read the sentences at Cambridge. Podiolo is known to have returned to Italy. Roth 1966 vol. II, 189 no. 447.

1368 Fr. John Tantucci of Siena [Italy], to be student at Cambridge. Roth 1966 vol. II, 203 no. 498.

1378 Start of Western Schism

1384 Fr. Dominic of Monte Policiano [Montelupicano, Italy], province of Siena, to be student at Cambridge. Roth 1966 vol. 2, no. 559.

1385 Fr. William of Toulouse [southern France] to be student in London or if impossible in Cambridge. Roth 1966 vol. II, 228 no. 572

1388 Fr. Mathew of Padua [Italy] to be student at Cambridge. Roth 1966 vol. II, 236 no. 592.

1389 John of Herford [Erfurt, northwest Germany] and John de Stargard [northwestern Poland], province of Saxony, to be students at Cambridge. Roth 1966 vol. II 236 no. 592, 243 no. 604.

1388 Fr. Paul of Milan recorded as being at Cambridge. Roth 1966 vol. II, 237 no. 594.

1388 Fr. Louis of Modena [Italy], bachelor, to read the sentences at Cambridge. He may stay in London during Lent. Roth 1966 vol. II, 238 no. 595.

1389 Fr, William of Perugia [Italy] to be student at Cambridge. Roth 1966 vol. II, 240 no. 599.

1389 John Corro, province of Cathalonia [Catalonia, northeast Spain] and Bernard Peris, province of Toulouse, to be student at Cambridge. Roth 1966 vol. II, 243 no. 604.

1390 John of Landshut [Germany], province of Bavaria, to be student at Cambridge. Roth 1966 vol. II, 244, no. 605.

1391 Fr. John of St. Thomas, province of Spain, to be student in Cambridge. Roth 1966 vol. II, 248 no. 616.

1391 Wynand of Lippstadt (Lippia) [northwest Germany] to be student at Cambridge. Roth 1966 vol. II, 507 no. 1167.

1392 Fr. Bernard of Constance [Konstanz, Germany] to be student at Cambridge, but actually student at Oxford in 1393. Roth 1966 vol. II, 249 no. 619.

1392 Fr. Peter of Leithomischl province of Bavaria to be student at Cambridge. Roth 1966 vol. II, 249 no. 619.

1414 Henry Offenburger of Strasbourg [eastern France], province of Rheno-Suebia, to be student at Cambridge. Offenburger is known to have returned to Strasbourg. Roth 1966 vol. II, 281 no. 690.

1417 End of Western Schism

1419 Peter Barrenii of the province of Toulouse [southern France] to lecture on sentences at Cambridge. Roth 1966 vol. II, 288 no. 713.

1419 Simon de Brunn, province of Bavaria, to be student at Cambridge. Brunn is known to have returned to Germany (see above). Roth 1966 vol. II, 288 no. 713.

1433 Richard of Ghent [Belgium], province of Cologne, to be student at Cambridge. Roth 1966 vol. II 315 no. 766.

1439 Arnold of Bruges [Belgium] and Theoderic Beylare [Marienthal, western Germany] to be students at Cambridge. Roth 1966 vol. II 323 no. 791.

# Radiocarbon dating and Bayesian analysis

By Craig Cessford and Craig Alexander

Four skeletons from the Austin friars were radiocarbon dated. Three of these came from the cemetery and formed a stratigraphic sequence, while the fourth is from the chapter house. In all cases the material dated was human rib bone. The samples were radiocarbon dated at the SUERC radiocarbon laboratory, following their standard procedures (Dunbar et al. 2016). Analysis was undertaken using OxCal v.4.3 (Bronk Ramsey 2009; Bronk Ramsey and Lee 2013) and the IntCal13 calibration curve (Reimer et al. 2013). (Table S1). For a number of reasons, the date ranges are older than the actual date when the individuals died. These include bone turnover during life and the consumption of marine fish, as reflected in the stable isotopes (see Stable Isotopes section in main paper). Allowance can be made for these factors (Table S2), although it should be noted that these represent best fit approximations for phenomena that are not well understood (for a fuller description of methods see Cessford and Alexander in prep.). The results were subject to Bayesian analysis (Bronk Ramsey 2009). This incorporated the textually attested start (1290) and end (1538) dates for burial at the friary and the second and latest dated burials from the cemetery post-dating a skeleton that tested positive for *Yersinia pestis* aDNA and are therefore later than 1348. It also included the chapter house burial being later than those from the cemetery, although at a practical level this had little, if any, impact. The Bayesian modelling was successful, with an individual agreement index (Aoverall) of 86.8.

# Human skeletal remains

By Benjamin Neil

This is an intra-site discussion of the inhumed and disarticulated human remains from the New Museums site, Cambridge. There were thirty-eight inhumations across the cemetery (n=32) and chapter house (n=6) with a range of five to twenty-five individuals from the disarticulated assemblage. The inhumed burials are discussed under twelve headings: burial position, preservation and taphonomy, demography, trauma (n=7, 18%), degeneration (n=18, 47%), enthesophytes (n=14, 37%), infection (n=11, 29%), neoplastic disease (n=5, 13%), endocrine (n=9, 24%), metabolic (n=8, 21%) and developmental disorders (n=12, 32%) and oral pathology (n=21, 55%). Thereafter a discussion on the disarticulated assemblage concerning its demography and pathology.

The criteria for aetiology (cause/initiation of trauma/disease) and pathogenesis (proliferation of disease) are reviewed to offer *possible* mechanisms of the conditions discussed, based on the available evidence; they are by no means definitive, wherein a range of differential considerations are explored.

When considering the *osteological paradox* (Wood et. al. 1992), many individuals in this assemblage bear skeletal evidence of disease. This implies that they were in relatively good health with strong immune systems that were able to mitigate, for a period, the detrimental effects of their condition(s). For example, the range of those with an inflammatory or possible endocrine disorder constitutes 60% of the cemetery/chapter house population. This may suggest a divergent nutritional behaviour from the wider community. Clinically, there are strong indications that nutrition plays an important role in disease risk in the fields of cancer, metabolic, cardiovascular and infectious diseases and neurological disorders (Lundstrom 2020). Bovine milk for example can mitigate bacterial infections and inflammatory diseases (Bowen and Pearson 1993; Lundstrom 2020). Although in its infancy, there is emerging data that suggests that diet can have a negative impact on the gut microbiome, which can lead to metabolic diseases (Lundstrom 2020; Noronha et al. 2019) such as gout, discussed below. Activity however can slow down the progression of many diseases, especially metabolic, where exercise positively affects nearly all the organ systems in the body (Gabriel and Zierath 2017). Evidence for activity in this population is inferential, based on the location and quality of degenerative characteristics, demonstrated in 47% of this assemblage.

## Methodology

Sex estimation was accomplished by identifying dimorphic dimensions using methods outlined by Buikstra *et al*. (Buikstra, Ubelaker, and Aftandilian 1994), Norén (Norén et al. 2005), and Rogers (Rogers 1999) and estimated according to Table S3. Metric dimensions supplemented these estimations following data outlined by France (1998). Age at death estimation (Table S4) was based on the degree of epiphyseal and apophyseal union (Cunningham et al. 2016), patterns of dental eruption (AlQahtani, Hector and Liversidge 2010) and skeletal degeneration (Brooks and Suchey 1990; Buckberry and Chamberlain 2002; Calce 2012; Falys and Prangle 2015). Precise ageing of older individuals is problematic. For individuals that were likely around or over 40 years of age, age estimates were made from the sternal end of the clavicle (Falys and Prangle 2015), which has been shown to be good at identifying individuals over the age of 60 years (Blom et al. 2018). This is not conclusive, but is useful in indicating individuals that might be considered elderly.

Teeth were catalogued using the Universal Notation System and recorded using the notations outlined in Table S5. Stature was estimated using data compiled by Trotter (Trotter 1970) and Mays (Mays 2016). The overall completeness of a skeleton was calculated according to the percentage of elements present (Rowbotham, Blau, and Hislop-Jambrich 2017). The level of fragmentation was assessed using the specialist’s own scoring system (Table S6). Stages of preservation followed the notation system developed by Mckinley (2004) (Table S7). Disarticulated, fragmented bone was recorded according to zonation criteria set out by (Knüsel and Outram 2004). Bone dimension was measured using a bespoke osteometric board and a 150mm digital sliding calliper (with a resolution of 0.01mm and accuracy of ± 0.02mm). The assemblage was studied for traumatic and pathological change, using paleo-pathological and modern clinical examples to inform on the health status of this sample.

## Position of inhumation burials

The physicality of the corpse within an inhumation can inform on ritual and the significance placed on the presentation of the body in their final resting place. All individuals across the cemetery and chapter house were aligned southwest-northeast, with the head in the southwest. Those in the cemetery were regimentally interred in three intercutting rows along a northwest-southeast axis. Truncation was predominantly seen along the ‘outside’ margins of the western and eastern rows.

Tables S8–10 summarise the physical attitude of the inhumations (Figures S1–3). All individuals were interred in a supine position. Lower limbs were always in an extended position and predominantly drawn together. The head and upper limbs were the most variably placed. The head was predominantly in a neutral position and the upper limbs were preferably placed over the thorax or abdomen/hips. There was general symmetry in the limb placement except for F.230 whose hands were placed together against the left side of the body.

There was a notable difference between cemetery and chapter house in the position of upper limbs; where the former group had their upper limbs positioned predominantly over the body (n=20), the latter were tight against the body (n=4). However, the comparison is skewed by the ratio between the two burial grounds, where three individuals from the cemetery also had their upper limbs straight.

During the period between the ninth and twelfth century, the position and condition of the body, and especially the head, became important (Daniell 1997). Although there is no archaeological evidence in these burial grounds to suggest the fixation of the head in an upright position (such as a head niche or stones), being the predominant position suggests that it may have had symbolic meaning (e.g., have a direct line of sight to the risen Christ). Arm position in this assemblage seems to follow an observation that towards the end of the Middle Ages, burial position (represented on monumental brasses and sculpture), was most frequently depicted with the hands in prayer, followed by a crossing of the upper limbs over the chest, and finally the hands over the groin (Daniell 1997). Five of the ten individuals whose upper limbs are positioned over the thorax (F.232, F.302, F.314, F.434, F.347) could be interpreted as having their hands together in prayer.

## Taphonomy and preservation

Localised green staining over the bone occurred in eight individuals (F.146, F.216, F.302, F.332, F.336, F.352 and F.367) (Table S11). This relates to the interaction of oxidative agents in the soil (oxygen, carbon dioxide) interacting with copper alloy objects in proximity to the bone. Any moisture, percolating water and increase in temperature would have accelerated this process (Dupras and Schultz 2014). The anatomical location was varied, six individuals (F.302, F.332, F.334, F.336, F.352 and F.367) had staining around the lumbosacral region and top of the thigh and on limb bones in close proximity, which related to the buckles that were associated with them. F.336 had copper alloy staining on the right parietal (side of the head), over the manubrium (top of the breastbone) and anterior surface of T5 (vertebra) and around the left wrist (radius and lunate). This alludes to three separate items of copper alloy, including a buckle. The item over the manubrium may reasonably have been a pendant that came to rest over the vertebra as the body decomposed. The item on the right parietal may have been some form of pin.

The inhumed assemblage was generally assessed to have a low level of skeletal fragmentation and a high level of skeletal preservation. Seventeen individuals (53%) in the cemetery were truncated to varying degrees (Table S12). The plane of truncation is inferred, based either on elements removed or the alignment of the intruding cut. The dominant form of truncation was axially (n=11), from a vertical cut across the body. A vertical cut along the body was likely responsible for the inferred sagittal plane of truncation.

## Demography

For a definition of the age and sex categories used here, see the methodology outlined in the catalogue. Age and sex identifications for the cemetery and chapter house, excluding attributions based on aDNA and disarticulated individuals are presented in Figures S4–5.

## Stature

Tables S13–14 illustrate the average, geometric mean, median and range of stature values of each age category within the cemetery and chapter house assemblages. Stature was calculated according to criteria outlined by Trotter (Trotter 1970). Across both the cemetery and chapter house, 28 individuals (74%) were assessed. Of these, 22 were based on maximum femoral length, and six on maximum humeral length. ??comment on values.

## Trauma

Seven individuals (18%) have some form of possible or salient mechanical trauma involving axial and appendicular elements (Table S15). The sacrum is a sexually dimorphic element, where there is a greater degree of continuous curvature in males, which can include a slight forward projection of the coccyx (Saukko and Knight 2016). Individual F.232 had an abnormal forward projecting sacrococcygeal angle, which neared 90°. This may have been idiopathic or resulted from a flexion fracture that subsequently healed. Flexion fractures at the sacrococcygeal junction result from external trauma such as a fall or a direct blow to the area (Maigne, Doursounian, and Jacquot 2020). Anatomically, the coccyx is a comparatively small and vestigial part of the vertebral column; whist there is an opinion that it is a comparatively redundant structure (Lee et al. 2016) it does serve as a significant attachment site for the pelvic diaphragm (Tortora and Derrickson 2014). Whether idiopathic or trauma related, abnormal sacrococcygeal angles are associated with coccydynia, where strain to the pelvic floor muscles and irritation of the surrounding soft tissues can cause focal pain (Nathan, Fisher and Roberts 2010). Clinically, the pain is typically worst when sitting and may be momentarily worse when moving from sit to stand (Foye, Shah and Sinha 2017). In cases where the fractured coccyx has been anteriorly displaced, cerebrospinal fluid leakage, peroneal numbness, and bowel and bladder dysfunction can occur (Cawley, Power and Murphy 2016).

Individual F.311 had three rib fractures to the right side of their thorax. Two of the more superior fractures between the fifth and seventh ribs had healed, albeit misaligned, yet the fracture to the tenth rib remained ununited.

Clinically, most rib fractures occur as a result of blunt force trauma or a fall (Anavian, Guthrie and Cole 2009) though are also associated with osteoporosis (Roberge, Morgenstern and Osborn 1984) and severe and sustained coughing (Kawahara et al. 1997). Generally, fractures of ribs four through nine may be associated with underlying injuries to the heart, lungs, or bronchi whilst fractures involving ribs 5 through 12 are at risk for solid organ injury in the abdomen (Skubic, Okafor and Nehra 2018).

Normally, most rib fractures heal without any long-term sequelae; they reside in a well vascularised periosteal and muscular sleeve, which provides an optimum environment for healing and stabilisation (Anavian, Guthrie and Cole 2009). However, the displacement and non-union of the rib fractures indicates a more complex injury with consequences for a slightly reduced hemithorax volume and stability. With the non-union, it was likely that mobility of the healing fracture exceeded the strain tolerance of the bone-healing unit, thus preventing fusion (Edwards and Hunt 2018). Clinically, there is ample evidence that rib fractures cause significant pain (Bugaev et al. 2016) and interruption to quality of life at 30 and 60 days (Marasco et al. 2015) if not beyond. Pneumonia is a significant risk factor where pain can prevent full inflation of the lungs, so allowing the far ends of the lungs to collapse (Brasel et al. 2006: Musonza and Todd 2018).

That individual F.311 had osteoarthritic changes in their pectoral girdle, back, left elbow and right knee may suggest they had a lifestyle (where these joints were habitually loaded) that increased the risk factor of these fractures.

F.332 is perhaps the most extraordinary individual in this assemblage regarding the number and mechanism of their traumata. They had neck fractures, bilateral leg fractures and a healed collar bone fracture (??Figure). A complex fracture pattern is seen in the bilateral mid shaft fractures to both femurs. In the right leg, a small butterfly segment on the posterolateral side is seen with comminution posteromedially, indicated by the absence of diaphyseal fragments. The fracture pattern continues in a spiral fashion anteriorly. In the left leg, a small, incomplete butterfly fracture is seen posteriomedially with comminution posterolaterally; again, indicated by the absence of diaphyseal fragments. The fracture continues in a transverse fashion anteriorly. The small size, convexity and acute angle of the butterfly segments indicate that it faced the impact side (Reber and Simmons 2015). This suggests the force was directed from behind and to the right of the individual. This type of fracture is commonly associated with a high impact trauma and according to clinical data most frequently occurs in young males (Court-Brown et al. 2015), as in this instance.

In clinical settings, bilateral femoral fractures carry a high risk of death, especially with associated vascular injury (Court-Brown et al. 2015; Lane, Nahm and Vallier 2015). A recent study suggests that blood loss from the femur itself may not be significant in the initial period following injury (Wertheimer et al. 2018). However, this study excluded patients with associated vascular trauma, which would have been a possibility with this individual. Depending on size and weight, the average human adult has nearly 5000ml of circulating blood (Sharma and Sharma 2020). It is estimated that 1000–1500 ml of blood can be lost in a unilateral closed fracture of the femur (Lee and Porter 2005). In severe injury, rapid blood loss of around 25% (1250ml) volume causes irreversible haemorrhagic shock (Türker 2019). This corresponds to a class II haemorrhage which would result in tachycardia, tachypnea and decreased pulse pressure (Papadakos and Gestring 2015).

The healing process would have been complicated if the individual were not properly immobilised and the fracture was not reduced (i.e., realigned). Powerful muscles that attach to the femur would have acted to deform the fracture: the gluteus medius and minimus muscles would have abducted the proximal half of the femurs and the iliopsoas would have flexed them, making it bulge up and out to the side. The distal segments would have been pulled towards the midline by the adductor muscles. There is no evidence of osteogenic remodelling or healing around the fracture margins, which indicates that if this individual did not die from shock, they would have survived for no more than a couple of weeks post injury (Tortora and Derrickson 2014).

This individual’s trauma to the cervicothoracic junction similarly had an absence of osteogenic remodelling around the fracture margins. They are generally characterised as closed/incomplete fractures through both body and lamina. The energy of the fracture (??Figure) through the thoracic body was dissipated by the foramen of the anterior internal vertebral venous plexus. It is generally difficult to ascribe a mechanism of injury from fracture pattern alone, though likely causes are hyperflexion and hyperextension (Court-Brown et al. 2015). Clinically with concurrent spinal fractures, there is a high index of suspicion of whiplash as the mechanism of injury (Dash 2019). It is a reasonable suggestion that this may have resulted from the same impact that caused the femoral fractures.

The lateral (acromial) end of the left clavicle has a contour abnormality on the articulating and inferior surfaces, extending 20mm medially. It is defined by an area of scalloped, porotic new bone. These regions serve as an attachment area for the Acromioclavicular, Trapezoid and Conoid ligaments. The abnormality may indicate trauma and /or a chronic overuse injury of the acromioclavicular joint (such as the habitual lifting and carrying of objects overhead). If a fracture, it would have clinically been defined as ‘type I’ (Holder, Kolla and Lehto 2017) being stabilised by the surrounding ligaments as it healed.

Individual F.344 had a supra-acetabular cyst in the left hip (??figure). Cysts in this region can result from several disorders, such as trauma, avascular necrosis of the femoral head, osteoarthritis and dysplasia (Mays 2005; Yukata 2015). The acetabulum of this individual otherwise appeared normal with no indication of dysplasia, or osteoarthritis. Thus, the likely mechanism of this cyst was a tear or separation of the labrum (a fibrocartilagenous extension of the acetabular rim) (Mays 2005) caused by an acute or chronic injury resulting from vigorous hip flexion and extension. Clinically, this is seen in relation to a variety of sports injuries, such as sprinting (Ikeda et al. 1988). The mechanism of cyst development results from a loss of congruity between the femoral head and acetabulum, which causes increased intraarticular pressure (Magee and Hinson 2000). This increased pressure forces synovial fluid through the tear via a one-way valve mechanism (Yukata 2015). The acetabular labrum contains nerve endings, where clinically there is a presentation of acute hip and groin pain (Groh and Herrera 2009).

Individual F.191 had a healing isolated fracture of the left ulna; clinically, these injuries are considered uncommon (Cai, Yan and Giddins 2013; Dymond 1984; Mackay, Wood, and Rangan 2000). Known as ‘night stick’ fractures, they are caused by direct low energy injury as the arm is raised to protect he body from a blow (Jong and De Jong 1989; Sauder and Athwal 2007; Streubel and Pesántez 2015). In the palaeopathological literature, they have classically been referred to as ‘parry’ fractures (Lovell 1997) for the inferred mechanism by which the injury is caused. Due to the implication of interpersonal and/or extramural violence it is a natural requirement to differentiate from other mechanisms of forearm injury, such as a fall onto an outstretched hand or from height. These can be typologically differentiated, for example, a Monteggia fracture-dislocation results in proximal oblique ulna fracture and dislocation of the radial head at the elbow and a Galeazzi fracture-dislocation results in proximal radial fracture and dislocation of the distal ulna from the carpus. Colles/Smiths fractures occur at the distal metaphysis of the radius. This fracture bears the criteria of a ‘parry’ fracture where the ipsilateral radius was unaffected, the fracture line was <45°, was located below the midshaft and had minor misalignment (Judd 2008). The energy of the impact is also a defining perimeter, where a higher energy fracture of the ulna diaphysis often results in non-union (Szabo and Skinner 1990).

## Degeneration

There was evidence for degenerative conditions on various regions of the skeleton, principally the back (Table S16: Figure S6). This assemblage is predominantly defined by the level of salient skeletal degeneration. Excluding osteoarthritis (discussed below), fifteen individuals (F.146, F.191, F.196, F.198, F.232, F.260, F.302, F.310, F.311, F.333, F.334, F.336, F.348, F.355 and F.367) had some form of osteogenic remodelling or porosity around and/or within articulations. This comprises c.40% of the assemblage. The most affected element was the back, indicated by spondylophytes and Schmorl’s nodes.

Spondylophytes were observed in nine individuals (F.191, F.260, F.302, F.311, F.331, F.333, F.336, F.344 and F.367). Four mature adults were affected, three old middle adults, one young middle adult and one young adult. Spondylophytes are osteophytes specific the vertebral column and are considered to be a general indicator of increased age (van der Kraan and van den Berg 2007). However, it is unclear whether formation serves to stabilise the joint as part of a normal remodelling process or develop and contribute to the pathology of joint dysfunction (Goldring and Goldring 2016). Biologically, it is likely that mechanical stimuli are transcribed to biochemical factors, which initiates the process of chondrogenesis, i.e., the forerunner of bone formation (see following discussion under OA and DISH for an expanded rationale). F.260 is an important exception to the age degenerative model (see discussion under other observations, below). Although the pathogenesis is still debated, range of movement and pain management concerning the spine is often discussed. Little attention however is given to the relationship of the surrounding soft tissues. In the case of Individual F.302, T8 has an anatomical relationship to the lungs and heart; depending on the size, spondylophytes can have a physiological and pathogenic effect on these tissues such as mechanical compression, irritation and trauma. When affecting the heart, this can lead to dyspnea (Tzikas et al. 2016), and when affecting the lungs, it can cause osteophyte induced lung fibrosis (considered asymptomatic, yet chronic) (Salvatore et al. 2017; Smith et al. 2017).

Schmorl’s nodes were observed in ten individuals (F.146, F.191, F.198, F.310, F.314, F.331, F.336, F.348 F.355 and F.367). Six mature adults, two young middle adults and two young adults were affected. The condition is essentially a physiological process rather than a disease and often inevitable where a constant erect posture puts continuous increased pressure on the spine (Ombregt 2013). Loading this anatomical unit only adds to this process. Functionally, it is a herniation of nucleus pulposus through the cartilage and bone into the body of the adjacent vertebra. Though these lesions are often asymptomatic, they are highly heritable (Williams et al. 2007) and can cause back pain when the herniation into the vertebral marrow irritates a nociceptive system (Kim and Jang 2018).

Porosity on subchondral bone was noted on eight individuals (F.146, F.196, F.217, F.232, F.311, F.334, F.336 and F.367). Four mature adults, two old middle adults and two broadly categorised adults were affected. As a marker of joint degeneration, and specifically osteoarthritis, the presence of porosity on subchondral bone has been debated for its relationship and significance (Rothschild 1997). However, it has been consistently recorded in conjunction with degenerative joint disease in the palaeopathological literature. Clinically, the appearance of porosity is considered the first feature of osteoarthritis (Findlay and Kuliwaba 2016; Goldring and Goldring 2016) and in general is the most frequent feature of this condition in young adults (Zampetti et al. 2016).

Osteoarthritis (OA) has traditionally been considered a mechanically degenerative disease, characterised in extreme cases by the loss of hyaline cartilage. However, it has more recently been described as a whole organ disease (Hardcastle et al. 2014), additionally indicated by osteophytic and enthesophytic involvement where common molecular pathways regulate bone formation in response to abnormal mechanical joint loading (Arden et al. 2018). Clinically, obesity and trauma have been shown to contribute to its progression (Winburn and Stock 2019). Here, osteoarthritis was defined by evidence of eburnation. Femoroacetabular impingements are also included as a known (though not definitive) precursor to osteoarthritis. Diffuse Idiopathic Skeletal Hyperostosis (DISH) is discussed here as a rheumatological disorder with a similar pathogenic pathway to OA (see below for an explanation). Together, this was noted in eight individuals (F.215, F.232, F.265, F.311, F.334, F.336, F.344 and F.352).

Eburnation was noted in four individuals (F.311, F.334, F.336 and F.344) and is osteologically considered to be pathognomonic of OA (Roberts 2017; Waldron 2021, 77). Between them, these individuals had osteoarthritic changes affecting the shoulder girdle, neck, elbow, knees and toe. Demographically, two mature adults and two old middle adults were affected. Eburnation is defined as an area of smooth polished bone at diarthrodial regions and occurs subsequent to cartilage destruction (Findlay and Kuliwaba 2016). The implication is pain, stiffness and reduced movement in the affected joints (Hunter and Felson 2006).

Three individuals (F.106, F.232 and F.352) were noted for plaque form defects on the anterior side of the femoral necks, which are concordant with femoroacetabular cam impingements (FAI) (type B, after Hack et al. 2010). It affected two young middle adults and one old middle adult. FAI are a pathomechanical alteration of the hip (Leunig, Beaulé and Ganz 2009) with variable aetiology. It is characterised as a contour abnormality at the femoral head-neck junction and has been attributed to an acquired developmental deformity (Nötzli et al. 2002) such as supraphysiologic motion or high impact trauma (Leunig, Beaulé and Ganz 2009). Both may play a role in hip instability (Court-Brown et al. 2015, 1984) and can develop into osteoarthritis (Beck et al. 2005; Eijer and Hogervorst 2017; Zhang et al. 2015). Clinically, this type of cam impingement is more common in men (Chaudhry and Ayeni 2014). Differentially there was no salient indication of slipped upper femoral epiphyses, avascular necrosis, or previous fracture that would otherwise account for this impingement.

Individual F.344, an old middle adult, was noted for DISH (also known as Forestier's disease), which affected six thoracic vertebrae. Although DISH can manifest in the cervical, lumbar and extraspinal regions, the right side of the thoracic spine is the most affected, as seen in this individual. The protective effect of the pulsatile aorta on the left side of the thoracic vertebrae is thought to be the reason for the right sided incidence (Belanger and Rowe 2001; Waldron 2021, 130–31). DISH is currently understood as a condition whereby the anterior longitudinal ligament of the spine ossifies as well as the entheses of the vertebrae (Mader et al. 2017), presenting osteologically and radiologically as flowing enthesophytes and syndesmophytes. It is important to acknowledge that the aetiology of this condition is still unknown yet has variably been associated with OA and described as a noninflammatory musculoskeletal disease. It is traditionally differentiated from the osteophytic/spondylophytic form of OA, which is clinically associated with mechanical and age-related degeneration, initiated by local entheseal inflammation (Mader et al. 2017). However, this model is also being reconsidered as a metabolic disease (Katz, Agrawal and Velasquez 2010).

This typological differentiation is muddied on a molecular level. Biologically, osteophyte, enthesophyte and syndesmophyte formation are (theoretically) a response to the stimulation of mesenchymal cells and chondrocytes; this in turn promotes new bone growth (Pillai and Littlejohn 2014; Sudoł-Szopińska et al. 2015). There is growing evidence that the pathogenesis of all these ‘phytes’ are associated with the activity of the hormone/cytokine, leptin, which has proinflammatory properties (La Cava 2017). It is also a key regulator of chondrocyte metabolism and known to be active in DISH as well as OA (Dumond et al. 2003; Kortyna 2017; Min et al. 2021; Pillai and Littlejohn 2014). As such, there is a trend for these bone formations to variably co-exist with each other, which are conflated with metabolic/endocrine disorders such as diabetes mellitus (Kortyna 2017; Mageed et al. 2017) increased Body Mass Index (BMI) (Mays 2012; Pillai and Littlejohn 2014; Katzman et al. 2017) and cardiovascular disease (Oudkerk et al. 2019). That increased BMI is positively associated with DISH (Mader and Lavi 2009), infers that mechanical stress is a factor whereby extra load increases thoracic kyphosis.

These factors give cause for many investigators to believe that DISH is a systemic condition, where genetic, metabolic and inflammatory pathways are variably at play. Symptoms of DISH include back pain and stiffness. Clinically, limited motion of the thoracic spine is the most reported abnormality of DISH; activities such as dressing, standing from a chair and reaching can all be impaired (Katzman et al. 2017).

## Enthesophytes

Enthesophytes are abnormal bony expressions at tendon insertion sites, extending in the direction of ligament or tendon pull (Hardcastle et al. 2014). The pathogenesis of enthesophytes is varied, but can manifest in response to inflammatory, mechanical and/or molecular mechanisms involving mesenchymal cells (Sudoł-Szopińska et al. 2015). Depending on the anatomical location, these expressions can variably be attributed as musculoskeletal stress markers (Villotte et al. 2010), either as a result of sustained overuse or trauma. Enthesophytes were noted in fourteen individuals (F.146, F.195, F.198, F.216, F.217, F.232, F.265, F.302, F.309, F.311, F.333, F.334, F.336 and F.367) which affected the head, neck, back, upper arm, elbow, forearm, hands, pelvis, thigh, knee and ankle. Five mature adults, five old middle adults, two young middle adults and two broadly categorised adults were affected. Qualitative scoring was based on the system outlined by (Villotte 2013), whereby expressions were differentiated as minor and major as well as asymmetry. Enthesophytes were seen on the patellae of seven individuals (F.195, F.198, F.265, F.311, F.333, F.336 and F.367), which is clinically referred to as ‘patella tooth’. This likely relates to an expression of mechanical overuse. The patella is a sesamoid bone that receives the quadriceps tendon superiorly and the patellar tendon inferiorly, which attaches to the tibial tuberosity. F.198 and F.302 have associated enthesophytes over the tibial tuberosity and by possible aetiological relation, F.195 and F.198 have enthesophytes on the calcaneal tuberosities (which may relate to a resisted contraction of the gastrocnemius muscle at the achilles tendon). The tibial tuberosity expressions may have developed during adolescence, which may relate to a condition known as Osgood-Schlatter Disease (Circi, Atalay and Beyzadeoglu 2017). This condition is associated with repetitive loading activities such as running, jumping and kneeling, which strain the patellar tendon entheses via the contraction of the quadriceps muscle (Gholve et al. 2007; Smith and Varacallo 2018). Clinically, this is a self-limiting condition and usually resolves and becomes asymptomatic by adulthood. However, sustaining any one of the above activities may continue to perpetuate the enthesophyte expression, where habitual isometric contractions of the of the quadriceps muscle during activities such as kneeling may maintain these expressions.

F.217 and F.302 (an adult and old middle adult respectively) both had enthesophyte expressions along the medial edge of the femoral linea aspera whilst F.302 also had medially directed enthesophytes over the lesser trochanter. The former corresponds to the insertions of adductor magnus and adductor longus muscles, which flexes, adducts and medially rotates the thighs at the hips. The latter corresponds to the activity of the iliopsoas muscle, which flexes the trunk and thigh at the hip and externally rotates the thigh at the hip. These expressions may relate to the habitual isometric contraction of muscles during activities such as horse- back riding (Djukic et al. 2018) which is supported by historical accounts of friars using them as a means of transport (Miller 2012; see also Webb 1921, 28).

The expressions seen at the second cervical vertebrae in F.216 and F.302, both old middle adults, may relate to the alar ligament (rather than the apical ligament, which is considered rudimentary, with no significant function (Tubbs et al. 2011). The alar ligament has an important role in stabilising the craniocervical junction (CVJ) and functions to limit axial rotation. It has been shown that there is variation in the attachment to the odontoid process, where fibres transverse and pass superiorly and anteriorly across it (Sardi et al. 2017). The fibres can be orientated horizontally, upward or downward (Debernardi et al. 2011). This supplements the standard anatomical illustration of the ligament attaching to the lateral borders of the process. The enthesophyte formation may relate to a condition known as Crowned Dens Syndrome (CDS) in which the alar ligament intermittently calcifies or ossifies (Koyfman and Yaffe 2014). I thus reason that in combination with the rarer variation of alar attachment to the odontoid, this individual may have had symptoms associated with CDS, such as occipital pain and neck stiffness (Ledingham, Cappelen-Smith and Cordato 2018). If this enthesophyte denoted frontal insertion onto the surface of the dens, the fibres would most likely have an anterior orientation and thus lose their biomechanical advantage to restrain lateral and sagittal flexion (Sardi et al. 2017).

Individuals F.232 and F.311 had moderate unilateral enthesophyte expressions at the right and left head of the ulnar olecranon, respectively. although the pathogenic aetiology is not well understood at this anatomical location (Benjamin et al. 2006), a trend of incidence occurs in those who perform repetitive isometric and eccentric contractions of the biceps bacchii muscle; the former contraction for example, fixes the elbow during tasks of fine motor control such as writing, the later during activities such as throwing and heavy lifting (Alvi et al. 2014).

Individuals’ F.198 and F.232 had enthesophyte expression for the ligamentum flavum insertion site (occasionally known as para-articular processes) throughout the thoracic vertebrae. These bony expressions would have functionally narrowed the vertebral foramen, thus compressed the spinal cord. This stenosis is largely asymptomatic in clinical settings, but in the minority of cases may cause neurological deficits (Ehara et al. 1998).

The expression seen at the occipital protuberance of F.309 may have related to habitual loading of the nuchal ligament (which extends from the external occipital protuberance to the spinous process of C7 vertebra). Its function is to limit hyperflexion of the neck, though if this individual carried out repetitive activities that involved a head forward/down posture, this spur may be the resulting biological response (Shahar and Sayers 2016).

Expressions in the left hand of individual F.367 relate to the sheath attachments (specifically the A4 annular ligaments) that retain the ligament flexors (digitorum sublimis) on the intermediate phalanges. This can also be considered an enthesopathy (Cashmore and Zakrzewski 2013). Of the annular ligaments in the hand, the A4 are the most important in preventing a condition known as bowstringing when the proximal interphalangeal joint is flexed (Schweizer 2001). Whether a heritable or habitual trait, the expression may have enabled this individual to sustain a strong grasp or grip.

Individual F.232 had asymmetric clavicles, where the left exhibited a larger acromial end and deeper impression for the costoclavicular ligament. This ligament binds the medial end of the clavicle to the first rib and functions to limit excessive superior, anterior and/or posterior movement at the medial end of the clavicle. This mirrors movements at the lateral end of the clavicle, which could suggest activities that load and depress the shoulder.

The following two individuals were noted for apparent systemic biomechanical adaptations. Individual F.146, the only adult female in this assemblage, was singularly noted for rugosity and enthesophyte formations at the tuberosities of the humeri, interosseous borders of the forearms and right fingers. All these expressions probably relate to a habitual resistance of a downward force. When the upper limbs are fully adducted (i.e., resting at the side of the body) the deltoid muscle functions to maintain joint stability by resisting forces pulling down on the humerus (i.e., resisting dislocating forces). For example, along with the rotator cuff muscles, the deltoid would resist the inferior displacement of the humerus from the scapula when carrying heavy objects. The expressions in the forearm reflects forces acting on the interosseous membrane, specifically the more ligamentous structure called the distal oblique bundle. This functions to maintain distal radial ulnar joint (DRUJ) stability, particularly under load (Wright 2001). This suggests a pathomechanical correlation with the deltoid attachment expression in the humerus. The expressions on the manual proximal phalanges relate to the palmar and dorsal interossei muscles. These not only abduct and adduct the fingers at the metacarpophalangeal joint, but also prevent hyperextension and stabilise the joint (Panchal-Kildare and Malone 2013). This may relate to a habitual requirement to resist extension at this joint under load.

Individual F.334 was noted for bilateral enthesophytes over the anterior superior aspect of the iliac crest (ASIS), which has a known correlation with DISH (Benjamin et al. 2006; Mader et al. 2017; Slonimsky et al. 2016). The aetiology however is poorly understood and should not be considered as having the same pathogenic pathway (Castells Navarro and Buckberry 2020). However, biomechanically, the obliquus externus adominus inserts onto the ASIS and in this case may have (for example) compensated for a loss of sagittal and lateral flexion at the lower thoracic unit and acted to stabilise the spinal column. Other actions of this muscle include trunk rotation, compression of the abdominal viscera and expiration. To further support this, enthesophytes were noted over the lateral aspect of the left femoral greater trochanter, which correlates to the insertion for the gluteus medius muscle that abducts and internally rotates the thigh at the hip and stabilises the pelvis. The action of this muscle on the left side may have had a functional link to the biomechanical consequence of the DISH occurring on the right side of this individual.

## Inflammation and possible infection

Seven individuals (F.191, F.260, F.309, F.328, F.343, F.352, F.355) all have a pathology defined by demarked periosteal new bone and sclerosis, confined to an area around the parietal tubers and occipital planum but never extending inferior to the temporal lines. These individuals mostly stood out for the absence of other salient extracranial inflammation related pathology. Comprising 18% of the assemblage, this may correspond to the practice of tonsuring, where the hair was shaved or clipped from the crown of the head. That the delineated area of infection matches this area somewhat supports this hypothesis. Tonsuring was the first step of marking the separation of the individual from lay life (Cullum 2004, 53) and symbolised obedience to an ecclesiastical lifestyle (Miller 2012). There is an inferred level of severity, where F.328 F.343 and F.355 have periosteal reactions only, whereas F.191 F.260 F.309 and F352 have sclerotic bone and venous sulci involvement (Table S17).

The aetiology of this inflammation is difficult to ascribe, especially as this author cannot find any direct historical evidence to support a link between tonsure and scalp infection. One possibility would be a severe form of folliculitis (Inskip pers. comm. 2020): caused by a bacterial infection resulting, for example, from the use of an unclean blade (Karthikeyan 2009). Another possibility is a form of ringworm (Tinea Capitis), caused by dermatophytes (an aerobic fungi) that invade and infect the keratinised layers of skin and hair (Piggott and Friedlander 2012).

Other Trichophyton sources come from animals such as dogs (*Microsporum canis*) and horses (*Trichophyton equinum*), both having contagious potential. Horses were present at the friary although there is no evidence for dogs. Dermatophyte infections are clinically common everywhere in the world (Borman et al. 2007) and likely to have been common in medieval England, being known as Tineas during this period (Ajello 1974) and remedied using peony (Romaní and Romaní 2017). The severe form of ringworm is known as kerion celsi: an inflammatory disease presenting as a localised boggy, indurated mass, often with superimposed vesicles and pustules and can be associated with fever and purulent drainage. Differentially, these reactions may be related to haematologically disseminated skeletal tuberculosis (see below), particularly individual F.343, where hypervascularisation of the vertebrae were noted. ??repetition from main text

## Tuberculosis

Three individuals (F.312, F.315 and F.343) had salient indications of skeletal tuberculosis, which is usually secondary to pulmonary tuberculosis (Edeiken et al. 1963). Tw o adolescents and a young adult were affected. The most common/classic representation of skeletal tuberculosis is the cavitation and hypervascularisation of the vertebral bodies, particularly in the thoracic (as seen in these individuals) then less frequently in the lumbar and cervical region (Spekker et al. 2012; Weaver and Lifeso 1984). Systemic (also known as extrapulmonary/haematologically disseminated) manifestations of tuberculosis are generally not known to occur in the absence of pulmonary involvement (Morris et al. 2002). When it does, *Mycobacterium tuberculosis* usually affects the metaphysis of tubular bones due to their vascularised nature (Edeiken et al. 1963). The presentation is usually a cystic abscess at articulating joints and surfaces: i.e., the hip (as seen in individual F.312) and knee (Cruz and Starke 2007). Less frequently the shoulder (Ostrowska et al. 2012) and elbow (Sagoo, Lakdawala, and Subbu 2011) are affected, yet both areas are affected in individual F.312. Rarely but particularly in children does it affect the diaphysis of tubular bones (Tuli 2016, 190) but has been documented to occur in the forearm (Varunjikar, Jayan and Gadre 2014) and tibia (Chattopadhyay et al. 2009) (see F.230 as a differential diagnosis).

Other atypical extraspinal indications can take the form of sharply circumscribed round, oval and granular lytic lesions (Cruz and Starke 2007; Pálfi et al. 2012; Spekker et al. 2020) particularly in the ribs and skull. The periosteal reactions in the ribs of F.312 may be interpreted as a reaction to the infection.

## Osteitis

Individual F.146, a mature adult female, had notable bilateral bone formation around the tibiae and fibulae, particularly distoanteriorly, taking a swollen appearance. This possibly relates to an infection, aggravated by long periods of standing. Both osteitis and osteomyelitis are descriptive terms in clinical and palaeopathological literature to describe inflammatory conditions (Ortner and Putschar 1981, 28) that result from a bacterial infection from either an external or systemic source. Unless there is an advantage of computed tomography (CT) it can be difficult to distinguish between the two.

Osteitis is centripetal where infection manifests external to the bone whereas osteomyelitis is centrifugal, being a primary infection of the bone marrow. Mechanisms of osteitis centre around soft tissue trauma, whereas osteomyelitis results from infection via the vascular system or an open fracture, which goes on to affect the cortex and periosteum (Tiemann and Hofmann 2009). In both cases, bacterial microorganisms such as *staphylococcus aureus* are known to bind to bone (Ciampolini and Harding 2000) and become internalised/encapsulated by new bone formation. Together with the bacteria’s ability to form a biofilm, it can effectively resist the hosts immune response (Masters et al. 2019), thus the condition can become chronic.

There is no evidence for fractures in this individual, nor a particular focus for the inflammation. In support of a haematologically derived osteomyelitis, the osteological response appears greater towards the distal metaphyses, which are a more vascularised area of bone. However, the risk of this mechanism is much higher in children due to the greater density of vessels in this region during development (Hatzenbuehler and Pulling 2011).

Inferring osteitis is more conjectural. Soft tissue trauma is a likely route yet determining whether it was primary or secondary is difficult. That the infection is bilateral suggests an infection secondary to a systemic process. One such condition could have been chronic leg ulcers, which have a well-known link with bone infection (Agale 2013; Masters et al. 2019; Schattner, Dubin, I and Gelber 2016; Valencia et al. 2001). These ulcers are caused by venous/arterial insufficiency where ineffective/damaged valves cause blood to flow the wrong way; this results in high pressure in the vessels when standing, which damages the skin and leads to the ulceration (Valencia et al. 2001). Venous ulcers most commonly occur above the medial or lateral malleoli and arterial ulcers often affect the anterior surface of the tibia (Agale 2013), which correspond to the locations in this individual. Healing can be further complicated by other factors, such as age over 65 years, diabetes mellitus, obesity, nicotine and alcohol abuse (Tiemann and Hofmann 2009).

Differentially, this could be attributable to osteopetrosis (also known as marble bone disease), which is characterised by bone overgrowth, narrowed medullary cavities, sclerosis and, brittleness (Waldron 2021, 274–75). It is a rare congenital metabolic bone disease of unknown aetiology resulting from defective or absent bone resorbing cells called osteoclasts (Arumugam et al. 2015). Clinically, it is divided into a malignant, autosomal recessive form (affecting children through to young adults) and the less severe benign, autosomal dominant form. In the case of this individual, it would most likely be the latter form, which develops later in life. There is a trend for the proximal humerus and distal femur to be affected with an increased risk of fracture of the affected element (not evident here) (Bailey and Tapscott 2020). Osteomyelitis is a well-known complication of osteopetrosis, particularly of the maxillae and mandible (again, not evident here in these elements).

A further differential could be attributed to a treponemal disease (such as venereal syphilis) where the most commonly affected skeletal element is the tibia, followed by the frontal and parietal bones, the nasal–palatal region and sternum (and other long bones) (Aufderheide and Rodriguez-Martin 1998). However, the skull of this individual bore no indication of infection.

Bone infections are also a well-known precursor of squamous-cell carcinoma (Tavares et al. 2011) (i.e., skin cancer) though clinically the incidence is quite low with an estimated rate of between 0.2% and 1.7% of all cases (Bauer et al. 2007).

## Possible bacterial infection

Individual F.140, a young middle adult, was noted for bilateral striated periosteal new bone over the femorae and the interosseous surface of right tibia, together with bilateral lesions over the anterodistal aspects of the tibiae. This may relate to a treponemal infection (Walker et al. 2015), but without the skull, it is a tenuous diagnosis. Differentially, it could be attributed to Paget’s disease of bone.

## Endocrine disorders

### Cribra orbitalia

Eight individuals were noted for cribra orbitalia (F.106, F.232, F.310, F314, F.333, F.336, F.347 and F.352). Although this phenomenon has long been recognised in the palaeopathological literature, the cause of this is still poorly understood. Cribra Orbitalia (CO) has in the past been interpreted alongside porotic hyperostosis (PH) due to the similarity in its appearance (Walker et al. 2009) and frequency of association (Cole and Waldron 2019), thus thought to be an expression of an anaemic condition. However, there is growing divergence in the aetiology of CO and PH; this is particularly so in cases of CO that present without associated PH diploic expansion. CO could be attributed to, for example, a normal developmental variation, eye trauma or infection (Cole and Waldron 2019). Other than F.336, all these individuals had bilateral expression, which is more likely attributable to either an endocrine disorder (particularly of the pituitary), chronic renal failure, or a hereditary condition such as thalassemia (Kozłowski and Witas 2012; Rivera and Lahr 2017).

The following two individuals stood apart for multiple skeletal involvement, likely attributable to an endocrine disorder. Individual F.352**,** a young middle adult, had bilateral cribra orbitalia, ossified costochondral cartilage, bilateral plaque form defects on the femoral neck and bilateral medial torsion (anteversion) of the femora. Although ossified costochondral cartilage is considered an age-related change in mature adults (over 40 years of age), it is unusual in younger individuals and can often be associated with malignancy, autoimmune and endocrine disorders, particularly of the thyroid such as Graves’ Disease (Ontell et al. 1997; Rhomberg and Schuster 2014). There may be a pathogenic link with the ossified costochondral cartilage and the plaque form defects, which are associated with FAI (see above). FAI in turn can be correlated to slipped upper femoral epiphysis (SCFE) though there was no salient indication in this individual. However, if subtle, it is worth pointing out that SCFE has a known correlation with endocrinological diseases (Aprato et al. 2019; Meester and Pantel 2014). There is also a known pathomechanical association between femoral anteversion and an onset of FAI (Ejnisman et al. 2013; Philippon et al. 2011). Differentially, there is also a secondary association between hip malformations and thyroid malignancy/disorder (Ball 2009, 401; Zandi and Hozhabri 2014), which is also associated with premature costochondral ossification (Ontell et al. 1997).

Individual F.230 was noted for extraordinary large lesions in the skull, left arm, right collar bone, left leg and right femur (see catalogue for details). They are possibly attributable to osteitis fibrosa cystica, commonly defined by long standing end-stage hyperparathyroidism (Bandeira et al. 2014; George et al. 2010; Jayker et al. 2017; Minisola et al. 2018). Although the condition is clinically rare, the long bones are among the first elements to be affected (Gupta et al. 2001) along with the clavicles and skull (Misiorowski et al. 2017) as seen here. This is often due to a tumour on the parathyroid glands, which are usually responsible for normal calcium metabolism; overactivity elevates the level of parathyroid hormone, which in turn causes excessive resorption of bone (Standring et al. 2016, 94) and a high perveance of renal dysfunction (Nair et al. 2016). The latter is known to be associated with bone resorption of the auricular surface (Weissman 2009, 664), which is also seen in this individual. There is also a known association with diabetes mellitus (Gulcelik et al. 2009). With resulting elevation in calcium blood levels (hypercalcemia) kidney stones can also form (Tortora and Derrickson 2014, 655). Symptoms include bone pain (George et al. 2010) fatigue, weakness (Taniegra 2004) and personality changes (Tortora and Derrickson 2014, 665).

Differentially, Tuberculosis may be attributable, though disseminated skeletal tuberculosis without primary foci is rare (Varunjikar, Jayan and Gadre 2014). Gorham’s syndrome, also known as vanishing bone disease, is a distinct possibility, sharing many of the characteristics of osteitis fibrosa cystica in terms of range of bones affected and polyfocal osteolysis. It is a rare musculoskeletal disease (Tavakoli Darestani et al. 2013), which causes progressive osteolysis; however, its aetiology is unknown. The tapering of bone ends, as seen in the left ulna of this individual, is also a characteristic, resembling a ‘licked candy stick’ (Ahlmann, Ma and Tunru-dinh 2011).

### Neoplastic disease

Five male individuals had benign bone tumours (F.216, F.265, F.344, F.352 ad F.367). Demographically this is represented by one young middle adult, three old middle adults and one mature adult. The exact pathogenesis of subungual exostosis seen in Individual F.216, an old middle adult, is unclear; trauma, infection, tumour, hereditary abnormality, or activation of a cartilaginous cyst have all been suggested as possible aetiologies. Most consider it to be a reactive metaplasia resulting from microtrauma (DaCambra, Gupta and Ferri-De-Barros 2014). Type 2 osteoarthritic exostoses are seen later in life, between the fourth and sixth decades, corresponding with the age of this individual. The exostosis located at the distal, dorsal, and central aspect of the tuft of the distal phalanx results from excessive distal interphalangeal dorsiflexion and jamming of the big toe against the shoe (Lemont and Goss 2005, 35).

The osteochondroma, seen on the on the right tibial medio-proximal metaphysis of Individual F.265 (an old middle adult), is also a bony exostosis. As a solitary proliferation, it clinically constitutes around 35% of all benign bone tumours (de Souza and Bispo Júnior 2014). Osteochondromas are generally defined by abnormal endochondral ossification during skeletal maturation, presenting as a cartilaginous capped bone growth. They generally form in limb bones, the most common being the distal femur, proximal humerus, proximal tibia, and fibula (Raggio et al. 2018). However, the skull (Zanotti et al. 2018) vertebrae (Yakkanti et al. 2018) and scapula (Sajid et al. 2019) can also be affected.

Although there has been uncertainty in the literature as to whether osteochondromas are true neoplasms or a developmental lesion (Murphey et al. 2000; de Souza and Bispo Júnior 2014; Tiwari et al. 2017), recent genetic work supports that both solitary and multiple osteochondromas are true neoplasms derived from a mutation in a cartilaginous cell of the growth plate (Raggio et al. 2018). Multiple osteochondromas are usually defined as a hereditary variant (an autosomal dominant disease), differentiated by the presence of two or more osteochondromas (Sajid et al. 2019). The location of this osteochondroma possibly caused Pes Anserinus Syndrome, where clinically, irritation of the overlying bursa and/or tendons of the sartorius, gracilis and semitendinosus muscles can cause spontaneous medial knee pain on climbing or descending stairs. Other symptoms can include tenderness and occasional swelling over the inside aspect of the knee, weakness or feeling of giving way around the knee and a decrease in knee range of motion due to pain (Curtis, Huang and Smitaman 2019; Sakamoto and Matsuda 2017).

Individuals’ F.344 (radial head) F.352 (skull) and F.367 (left foot) had a solitary osteoma/hamartoma (see catalogue for details). Osteomata, osteoid osteomata, and hamartomata are all benign tumours, though as with exostoses and osteochondromata, there is debate as to whether they are differentially an inflammatory regenerating process due to an association with trauma (Eshed et al. 2002; El Fatayri et al. 2019). They all have a similar osteological presentation and can be hard to differentiate macroscopically. An osteoma (sometimes referred to as a ‘button osteoma’ or ‘button lesion’ in palaeopathological literature) is a slow-growing primary tumour, normally located on the ectocranial surface of the skull; it rarely presents on the inner table (Aufderheide and Rodriguez-Martin 1998). Multiple osteomata are not common, where such cases are associated with the autosomal disorder ‘Gardner syndrome’ (Giuffra et al. 2019). Osteomata are typically less than 10mm in diameter, and appear as well circumscribed ‘ivory like’ lamellar bone (Aufderheide and Rodriguez-Martin 1998). Osteoid osteomas are like button osteomata but are epidemiologically different; they are relatively common, occur post cranially (Cąkar et al. 2015), and variously described as being less than 20 or 15mm diameter. Hamartomas are characterised by a focal malformation of disorganised tissue (Aufderheide and Rodriguez-Martin 1998), and generally of the same dimension as an osteoid osteoma (though up to 60mm in soft tissues). They represent spontaneous growth disturbances within a circumscribed area which creates a local elevation, capped with lamellated bone (Eshed et al. 2002). To complicate the epidemiology of all three, Eshed et al. (Eshed et al. 2002) suggest that button osteomata are a misnomer. Based on histological differentiation, they suggest ‘button hamartoma’. However, in lieu of histology, Giuffra *et al.* (Giuffra et al. 2019) refer to these tumours as button lesions.

## Metabolic disorders

### Gout

There were five possible cases of gout (F.215, F.217, F.232, F.336 and F.367) affecting the pedal metatarsals and proximal and intermediate phalanges. Three mature adults, one old middle adult and one broadly categorised adult were affected. The condition was determined by porotic/erosive lesions with associated osteophytic and sclerotic development. Gout is often described as inflammatory arthritis and a metabolic disease (Thottam, Krasnokutsky and Pillinger 2017) where the metabolism of uric acid is either increased or its excretion through the kidney decreased (due to renal disease) (Waldron, T. 2021, 119). There is a predilection for gout to affect certain joints (particularly the metatarsophalangeal and interphalangeal joints) whereby uric crystals form in areas where hyaline cartilage has been compromised by osteoarthritis (Roddy and Doherty 2010). The condition is most prevalent in males over 40 years (Kozłowski and Witas 2012), which broadly fits with these individual’s age categories. Diet and alcohol consumption are well known associative factors in the epidemiology of gout; high consumption levels of beer, fructose sugars, fish and meat and low intake of vitamin C appear to have a correlation with gout; the consumption of purine rich vegetables and dairy products conversely have no association (Roddy and Doherty 2010). High blood pressure, a high body mass index and cardiovascular disease are also associated with gout (Thottam, Krasnokutsky and Pillinger 2017).

### Vitamin C deficiency (scurvy)

There were three possible cases of a scorbutic episode (scurvy) in individuals’ F.346, F.352 and F.355, affecting a juvenile and two young middle adults. This was indicted by the porotic and spiculated palatine process of F.352 and bilateral porotic hyperostosis over the supraorbital ridge and zygomata of F.355. The most salient indications were of F.346, with bilateral periosteal reactions over the zygomata (including the masseteric origin), the posterior aspect of the temporal squamae, the mandibular fossae, the mastoid processes, the occipital condyles, the occipital protuberance and anterior tables of the patellae. Most systemic pathologies that involve the periosteum largely affect the diaphysis of long bones (Rana, Wu and Eisenberg 2009). However, all these regions are confined to the skull, which makes them diagnostically concordant with prolonged episodes of ascorbic acid (Vitamin C) deficiency (Ortner and Ericksen 1997). Ascorbic acid is essential in the biosynthesis of collagen and bone cell (osteoid) formation and is only available through the diet. If this is perpetually compromised, blood vessels become fragile and susceptible to rupture, and periosteal membranes equally develop a propensity to tear and bleed (Klaus 2017). The pathological regions noted in these individuals are mostly subcutaneous, thus the vascular and periosteal structures in these regions were susceptible to minor traumata, bleeding and thus an inflammatory response. Differentially, the pathology of the palatine process may be due to masticatory trauma (Mays 2008).

## Developmental disorders

### Symphalangism

Distal symphalangism was noted in five individuals (F.198, F.216, F.310, F.336 and F.348); four were affected in the toes, and one in the hand (F.336). Two mature adults, one old middle adult, one young middle adult and one young adult were affected. This condition refers to interphalangeal ankylosis (union), involving the proximal or distal interphalangeal joints, although proximal involvement is more common (Chen 2017). Although all manual and pedal digits can be affected, it commonly appears in the 5th toe, followed by the 4th toe and the 5th finger of the hand (Barnes 2012). It is a rare genetic disorder caused by mutations of the nogin (NOG) gene (Mundlos 2016), is strongly heritable and is frequently accompanied by conductive hearing loss (Masuda et al. 2014). Differentially, distal symphalangism has been attributable to mild brachydactyly type B, but this is also accompanied by vertebral fusions (Mundlos 2016), which are absent in all these individuals. Osteoarthritis is also differentially attributable, secondary to trauma; however, there were no bridging osteophytes to suggest this.

### Spina bifida occulta

Spina bifida occulta was noted in five individuals (F.216, F.232, F.260, F.314, F.331). Three old middle adults and two young adults were affected. The condition is characterised by non-fusion of the sacral lamina, which exposes the neural tube. It is considered common and rarely has any consequences on function (Kumar and Tubbs 2011) but is linked to a genetic and nutritional deficiency (Anderson and Spain 2016; Cunningham et al. 2016). The site of the spinal defect is sometimes marked by a slight swelling, a dimple in the skin, or a tuft of hairs (Anderson and Spain 2016, 13).

### Craniosynostosis

Craniosynostosis was noted in individuals’ F.311 and F.367. The condition can be classified as either primary (genetic) or secondary (for example, to metabolic disorders) (Kabbani and Raghuveer 2004). Purposeful cranial deformation is also a known cause and can be differentiated by an increased presence of wormian bones (Sanchez-Lara et al. 2007) or an absence of abnormal sutural fusion (Nelson and Madimenos 2010). The presence of sagittal synostosis, as seen in these individuals, represents the most common type of non-syndromic single-suture fusion (David et al. 2009). Clinically, it is estimated to affect 1 in every 2000 live births worldwide, occurring more frequently in males than females (Al-Shaqsi et al. 2018). Studies have shown that there is a risk of raised intercranial pressure and a 35–50% risk of neurocognitive deficiencies such as reduced attention, planning, speech, behavioural and learning disabilities (Thiele-Nygaard, Foss-Skiftesvik and Juhler 2020).

### Transitional vertebrae

Individuals’ F.314 and F.336 both had Lumbosacral Transitional Vertebrae (LSTV). LSTV is the most common congenital anomaly of the lumbosacral spine and may manifest either as a sacral assimilation of the 5th lumbar vertebra (sacralisation) or separation of the 1st sacral vertebra into the lumbar spine (lumbarisation). F.314 was a lumbarisation of the most superior sacral segment (i.e., embryologically derived from S1) whilst F.336 was sacralised. The sacralised LSTV (type IIa after (Konin and Walz 2010) refers to the cranial shift of the Lumbar–Sacral (L-S) border forcing the last lumbar vertebra to become part of the sacrum (Barnes 2012, 65). The prevalence of LSTV is said to vary between 7% and 30% (Sekharappa et al. 2014). Although a causal link between LSTV and lower back pain still debated (Yokoyama et al. 2016) several studies have found an association, particularly with the type II variant (Apazidis et al. 2011). LSTV is known to affect sacral tilt by reducing lumbar lordosis (Benlidayi, Coskun and Basaran 2015; Lian, Levine and Cho, W. 2018). This has the biomechanical effect of shifting the nucleus pulposus posteriorly, thus reducing the angle between the anterior margins of the lumbar bodies (Mansfield and Neumann 2009, 202). This may account for the large spondylophytes seen on L3 and L4 where formation may have been a biological response to joint stabilisation.

Individual F.336 also had a supernumerary thoracolumbar transitional vertebra (TLTV). Likewise congenital, it forms in-utero from an extra pair of somites added to the vertebral column (Barnes 2012, 70). In this instance, the extra vertebral segment became a transitional vertebra between the borders of the thoracic and lumbar vertebra. The presence of a supernumeric thoracic vertebra is clinically rare (Doo et al. 2020). The transitional site from the kyphotic thoracic to the lordotic lumbar region is uniquely predisposed to stress caused by the transmission of energy through this region (Smith et al. 2010) and a known weak biomechanical structure (Kim, Dan and Shin 2015). The addition of an extra vertebrae in this region is likely to exacerbate and destabilise this region (Du Plessis 2018). Symptoms, if present are characterised by back pain and referred visceral and posterior iliac crest pain (Du Plessis, Greyling and Page 2018).

### Sternal foramen

Individual F.311 had a sternal foramen 5.31mm diameter. Although sternal foramina are not unusual, they are still considered rare (Crubézy 1992), yet largely asymptomatic (Turkay et al. 2017) affecting around 7% of Europeans (Janssens 1960). Clinically reported prevalence varies (Gossner 2013) reports between 4.5 and 6.7%. He cites (Yekeler et al. 2006) for the former, whose study comprised 1,000 consecutive patients (582 men and 418 women; age range, 20–92 years). Turkay *et al*. (Turkay et al. 2017) reported a similar incidence where 5.2% of 544 individuals ranging in age from 18 to 95 years had a sternal foramen. Sternal abnormalities have been associated with cardiac defects, either via displacement of the heart (Aktan and Savaş 1998) or vascular bundles making passage though the embryonic cartilaginous bundles of the sternum (Crubézy, 1992) . A more general common explanation is due to incomplete ossification of the cartilaginous bundles during development (McCormick 1981). Prevalence of the foramen between the sexes is ambiguous, where Janssens (Janssens 1960) suggests that it occurs more frequently in females, McCormick (McCormick 1981) and Singh & Pathak (Singh and Pathak 2013) suggests it occurs more frequently in males.

### Accessory transverse foramen

Individual F.311 had a single Accessory transverse foramen (ATF) on the left side of C5. These are formed when vestigial costal elements fuse to the vertebral body around the vertebral vascular bundle during development; they may be absent or present in duplicate or triplicate (Heary et al. 1996). Usually overlooked as a non-metric trait, the reported clinical incidence of ATF is variable; Taitz, Nathan and Arensburg found a double transverse foramen in 7% of all specimens (1978). More recent studies on smaller samples see significant variation in incidence between 1.6% (Murlimanju et al. 2011) and 20% (Singh, Anand and Singh 2019). Biomechanically, a double transverse foramen can be asymptomatic, but also has the potential to cause stenosis or occlusion of the vertebral artery with head rotation, causing further potential to restrict blood flow (ischemia) and a blood clot (Sangari et al. 2015). Symptoms of this can cause intermittent dizziness, headaches, vertigo, tinnitus and hearing loss (Dorobisz et al. 2019; Jadeja and Nalleballe 2018) and can be exacerbated by turning the head and can lead to stroke.

### Bilateral elongated styloid processes

Individual F.315 was noted for abnormal styloid processes of the temporal bones. This can result from a natural elongation of the styloid itself or from ossification of the stylohyoid ligament (Mann and Hunt 2005). The normal range of the styloid is 20–30mm and is considered elongated when greater than this (Moffat, Ramsden and Shaw 1977). Clinically known as Eagle’s syndrome when symptomatic, it can include cervical neck pain, throat pain, a foreign body sensation in throat, earache, headache, pain in the cervicofacial region, pain on swallowing and pain on changing head position (Müderris et al. 2014). although 50% of cases are asymptomatic (Mann and Hunt 2005). The exact cause of the elongated styloid process is not clear; local chronic irritations, endocrine disorders, mechanical stress or trauma during development could all result in calcification (Gokce, Sisman and Sipahioglu 2008).

### Spondylolysis, spondylolisthesis and a biomechanical adaptation

Individual F.260 was noted for a degenerative condition of the lower back. It is clinically known that most spondylolysis (pars fracture) defects occur in young children, with an increasing incidence until the age of 20, which fits in with the age bracket of this individual; the progression of vertebral slip (spondylolisthesis) is also relatively rare after adolescence (Ohmori et al. 1995). The spondylophytes seen in this individual are likely to be a biological response to intervertebral disc degeneration (IDD) between L4 and L5. IDD is known to be common among young adults (Takatalo et al. 2009) and can begin as early as the second decade of life (Zobel et al. 2012). A study of 538 participants from a Finnish population with a mean age of 19 suggests a genetic predisposition (Kelempisioti et al. 2011). Another study of 558 participants with a mean age of 21 determined a correlation of IDD with a higher Body Mass Index in males, yet no correlation with physical activity (Takatalo et al. 2013). Expressions for the psoas muscle attachment was noted on the right side of L2–L4 (Bogduk, Pearcy and Hadfield 1992). The psoas functions as a core stabiliser as well as flexion of the thigh/trunk, lateral rotation of the thigh and lateral flexion of the trunk. at maximum contraction, the psoas exerts large compression and shear forces on the lumbar segments. It is reasonable to suggest then, that the expressions seen on the right side of the bodies relate to a biomechanical compensation of the lumbosacral instability. Differentially, this was not an expression of ‘cupids bow’, which is a normal anatomical contour variant of the lumbar body rim (Weissman 2009).

## Oral pathologies of the inhumed individuals

Twenty-six individuals had their skull present, and twenty-nine individuals had recordable dentition.

### Ante-mortem tooth loss (ATML)

ATML was noted in middle and mature adults of six individuals (F.146, F.191, F.331, F.333, F.344, F.348, and F.352), defined by any stage of alveolar resorption. Although an age-related phenomenon, it is clinically associated with poor oral hygiene after advanced carious lesions, periodontal disease and trauma.

### Dental calculus

Of the 29 individuals with teeth present, nineteen were recorded with dental calculus which was graded following Brothwell (1981) (Table S18). Sixteen presented with lingual calculus, fifteen with labial calculus and eleven with buccal calculus. Although there is a logical expectation that calculus has an increased prevalence with age, there does not appear to be any meaningful corelation here. Dental calculus is a mineralised bacterial plaque that can form within a matter of days (Lieverse 1999). Its formation is dependent on a multitude of factors such as the consumption of alkaline foods and sugars, and individual variation such as their bacterial load genetic variation in the salivary content and the presence of disease (Akcali and Lang 2017). If the calculus is left to form, the implication is an increased susceptibility to oral disease, for example gingivitis (Akcali and Lang 2017) and periodontal disease (Timmerman and Van der Weijden 2006). In archaeological populations, the general inference (in lieu of biomolecular analysis) is that they did not practice regular oral hygiene. It is suggested that Medieval practices of oral hygiene were rudimentary, where palliative care was largely confined to non-invasive herbal remedies, designed to relieve pain and/or hasten the removal of a diseased tooth (Anderson 2004). A study on a medieval London population from four cemeteries concluded that adults with dental calculus had significantly lower survivorship and died at younger ages compared to individuals without dental calculus (Yaussy and Dewitte 2019).

### Carious lesions

Eight individuals had caries (F.146, F.230, F.311, F.331, F.336, F.344, F.348 and F.352). The most frequent location was interproximal (five individuals) followed by occlusal (four individuals), and cervical (two individuals). There was a near even distribution across the age brackets, with caries noted in one juvenile, four middle adults (two young, two old), and two mature adults.

Caries result from the localised destruction of dental enamel by the acidic by-products of bacterial fermentation of dietary carbohydrates (Selwitz, Ismail and Pitts 2007). Untreated caries in permanent teeth is the most prevalent modern clinical condition in humans, affecting about 35% of the world population (Kassebaum et al. 2015). The frequency of caries in archaeological populations have been used in previous studies as a proxy measure of carbohydrate consumption; the inference being that there was either a transition away from a protein dominant diet and/or an increase of cultivated crops added to the diet (Hillson 2008). It is worth noting that consuming milk at the same time as carbohydrate rich foods buffers the cariogenic effect on teeth. Properties in milk proteins are antibacterial (Bowen and Pearson 1993) and are capable of preventing demineralization of the enamel (Aimutis 2004).

### Periapical abscesses

Periapical abscesses were noted in four individuals (F.331, F.344, F.352 and F.191). Individual F.344 had multiple instances. Demographically, three middle adults (one young, two old) and a mature adult were affected. This pathology occurs secondary to dental caries, trauma (Robertson and Smith 2009), and possibly as a result of a poorly extracted tooth. It results from a persistent presence of infective material within the root and around the apex of a tooth. Tissue liquefaction and an accumulation of pus would have caused pain and tenderness of the tooth to pressure. Before the introduction of antibiotics, dental abscesses were a leading cause of mortality (Clarke 1999; Robertson and Smith 2009). All the abscesses identified were periapical and clinically are the most common form of dental infection (Siqueira and Rôças 2013).

### Periodontal disease

Two individuals were noted for moderate periodontal disease (F.191 and F.344). This condition is caused by a variety of pathogenic agents that are found in oral biofilms such as dental plaque (a precursor to calculus). This can result in inflammation and destruction of gum tissue (gingiva), the periodontal ligament, root cementum, and alveolar bone (Dewitte and Bekvalac 2011). The presentation in these cases were diffuse porotic alteration of the palatine process and alveolar margin.

### Buccal pits

Buccal pits were noted on individuals F.312, F.315 and F.343. Osteologically, buccal pits are a non-metric trait. Because they are an uncommon observation, they warrant further discussion. There is uncertainty in the literature as to what these traits relate to, either being a negative trait of a protostylid (i.e., an additional supernumerary cusp) or an otherwise independent expression of unknown aetiology (Richard-Scott et al. 2018). Also known as foramen caecum (Hillson 2002), a buccal pit marks the cervical termination of the mesio-buccal groove on mandibular molars (Pfeiffer 1979).

Protostylid expression is most commonly seen in the lower first molars (Richard-Scott et al. 2018). As observed here, buccal pits are routinely scored as the lowest degree of expression at grade 1, where the protostylid’s presence cannot be observed (Moreno, Reyes, and Moreno 2016). There is an apparent genetic predisposition to the formation of buccal pits (Alt, Loring Brace and Türp 1998) and is one of several non-metric dental traits that are used in the estimation of ancestry (Richard-Scott et al. 2018) and may also suggest a genetic relationship between individuals (Nirmala et al. 2013). However, because there is no positive enamel relief to suggest the amelogenesis (formation) of the supernumerary cusp, caution is advocated in attributing buccal pits to this trait (Katzenberg and Grauer 2019). Alternatively, the pit may be a developmental defect of the enamel and has been correlated with enamel hypoplasia in some populations (Schepartz 2010). In terms of oral health, buccal pits are a known caries-susceptible areas (Naaman, El-Housseiny, and Alamoudi 2017; Pfeiffer 1979).

### Enamel hypoplasia

Enamel hypoplasia was noted in seven individuals (F.190, F.309, F.310, F.334, F.336, F.343 and F.347), present in one juvenile, two adolescents, one young adult, two middle adults (one young, one old) and a mature adult. This dental pathology manifests when the individual is exposed to physiological stress in early life whilst tooth crowns are developing (Nelson 2018). There is generally a greater prevalence in men than women (Larsen 2015). The progenitor of this physiological stress is far from certain in the literature. These defects have in the past been tantalizingly attributed to the onset of weaning. The idea is that the child experiences a chronic nutritional deficiency as they move onto solid foods (Blakey, Leslie and Reidy 1994; Lanphear 1990). However, the epidemiology is more complex: other factors such as fever, disease, the mothers health and other environmental factors can all contribute (Katzenberg, Herring and Saunders 1996) so weaning cannot be the sole reason. Rather, it is a set of circumstances, of which weaning is one, that give the infant a poor growth environment (Larsen 2015), so resulting in these enamel defects.

## The disarticulated/comingled assemblage

Eleven features and seven layers contained disarticulated human bone. A right adult tibia fragment from [1534] likely relates to the cloister burials excavated from the footings of the related building constructed in 1908–09. This was some distance from the cemetery and chapter house group and is excluded from the following discussion.

### Minimum number based on element abundance (MNE) and age

Based on the most abundant non reproducible element (right temporal) there was a minimum number of four adult individuals. Within the sub-adult category, there was a minimum number of one. Although the most frequently occurring fragments came from the skull (n=151) no anatomical region of the skull exceeded a total of five non reproducible element counts (Table S19). Cranially, eleven features/layers contained seventeen left sided elements, eight features/layers contained eleven right sided elements, three features contained a single pair of elements (nasal, parietal and temporal) and nine features/layers contained nine un-sided elements (to include single axial elements such as the occipital and sphenoid bones). Postcranially, one feature had paired pelvic bones. There were eighteen left sided elements (from 18 features/layers) twenty-eight right sided elements (from 28 features/layers) and eleven un-sided elements (from eleven features/layers), which include four vertebrae (Table S20). Zonation information is presented in Table S21. Multiple individuals were found within F.106, F.343, F397 and contexts [1006] and [1391]. The additional individual from F.106 represented c.45% of a young middle adult. Due to the representative quantity of bone for this individual, it has been distinguished from the rest of the disarticulated bone in the catalogue. The additional individual from F.343 represents c.12% (the head and right arm) of a young adult male. F.397 contains a minimum number of three individuals (based on the number of left temporal bones) and is representative of a true charnel pit. The bone from contexts [1006] and [1391] contained two individuals, respectively. This sums to a minimum of seven individuals.

### Number of individuals based on MNE and feature count

If each feature/layer is considered a discrete unit of bone that represents an individual or individuals, there were twenty-three adults and two sub-adults (Table S22). In all, this confers a range of between five to twenty-five ‘individuals’. Most of these ‘individuals’ probably represent elements of partially truncated inhumations that are already included in the cemetery counts. Based on age/sex and stratigraphic location there are only two sets of disarticulated remains that are likely to represent additional individuals.

### Age

In most instances age was categorised within the adult (18+) category; this was further refined for F.106 [1014] and F.343 [1916] being a young middle adult and young adult, respectively. Bone from F.112 and [1006] was assessed within the sub-adult category.

### Sex

36% of the disarticulated assemblage was assessed within the male category; 59% were indeterminate. The individual assessed as a possible female from F.397 might reflect the morphological variance of the sexual characteristic that provided the estimate and may in fact be male.

### Pathology

The conditions summarised in Table S23 are broadly discussed in the above categories of the inhumed assemblage.

# Burial catalogue for the cemetery and chapter house

By Benjamin Neil & Craig Cessford, incorporating specialist information from Martin Allen (jettons), Esther Cameron (textiles), Andy Hall (copper alloy and iron buckles), Quita Mould (leather straps), Ian Riddler (bone and ivory buckles), Christiana L. Scheib (genetic sex and MtDNA) and Justin Wiles Hall (copper alloy and iron buckles)

## The cemetery

| **F.106, skeleton 1866**. Figure S8 | |
| --- | --- |
| Date of burial | 1302–49 |
| Interpretation of individual | Laity: child of benefactor? |
| Grave shape and dimensions (m) | Trapezoidal: 1.4+ by 088, 0.49 deep (originally *c*. 1.0 deep) |
| Burial position | South-southwest–north-northeast, extended, supine, skull rotated and flexed to look over left shoulder. The upper limbs were parallel to body, right side tight against the body, left under the left os coxae. The lower limbs were straight |
| Associated items | None |
| Age | Infant: 0–4 years |
| Morphological sex | Indeterminate. Initially identified as possibly male, based on Schutkowski (Schutkowski 1993). This method has a poor correlation in subsequent published studies and in this instance disagrees with the genetic sex |
| Genetic sex | XX: female |
| MtDNA | T2e1 |
| Stature (cm) | Too young to calculate |
| Percentage of skeleton present | *c*. 95 (effectively complete) |
| Preservation grade | 0: high |
| Fragmentation grade | 1: low |
| Other taphonomic alteration | None observed |
| Trauma | None observed |
| Pathology | Systemic stress indicator (bilateral cribra orbitalia) |
| Other skeletal observations | None |
| Dental pathology | None observed |

| *p* | *p* | *p* | *p* | *p* | *p* | *p* | *p* | *p* | *p* |
| --- | --- | --- | --- | --- | --- | --- | --- | --- | --- |
| A | B | C | D | E | F | G | H | I | J |
| T | S | R | Q | P | O | N | M | L | K |
| *p* | *p* | *p* | *p* | *p* | *p* | *p* | *p* | *p* | *p* |

| **F.106 (disarticulated), skeleton 1014** | |
| --- | --- |
| Date of burial | 1290–1329 |
| Interpretation of individual | Unknown |
| Grave shape and dimensions (m) | Unknown, as the grave was larger than required for the primary F.106 burial it is likely that this approximately replicates the earlier cut |
| Burial position | Entirely disarticulated in later grave F.106, with no *in situ* traces of earlier grave or skeleton. No evidence that the disarticulated bones were deliberately arranged |
| Associated items | None |
| Age | Young middle adult: 26–35 years |
| Morphological sex | Male |
| Genetic sex | Not sampled |
| MtDNA | Not sampled |
| Stature | 166.84 +/- 3.27 |
| Percentage of skeleton present | *c*. 45 |
| Preservation grade | 0: high |
| Fragmentation grade | 1: low |
| Other taphonomic alteration | None observed |
| Trauma | None observed |
| Pathology | Gout? Septic bursitis? (the left una olecranon has a stippled, ‘cloud’ like lobulations and course trabeculation located within the ulnar tuberosity, around the olecranon, extending laterally, bordered by the supinator crest)  Degeneration in the left hip (plaque form defect around the anterior side of the left femoral neck. Type B (after Hack et al. 2010) femoroacetabular cam impingement) |
| Other skeletal observations | None |
| Dental pathology | Mineralised dental plaque: heavy labial calculus over the mandibular teeth  Tooth loss: ante-mortem loss of (19), with resorbing alveolar bone |

| -- | -- | -- | -- | -- | -- | -- | -- | -- | -- | -- | -- | -- | -- | -- |
| --- | --- | --- | --- | --- | --- | --- | --- | --- | --- | --- | --- | --- | --- | --- |
| 1 | 3 | 4 | 5 | 6 | 7 | 8 | 9 | 10 | 11 | 12 | 13 | 14 | 15 | 16 |
| 32 | 30 | 29 | 28 | 27 | 26 | 25 | 24 | 23 | 22 | 21 | 20 | 19 | 18 | 17 |
| cl | / | cl | cl | cl | cl | cl | cl | cl | cl | cl | cl | x | p | p |

| **F.140, skeleton 1139**, Figure S9 | |
| --- | --- |
| Date of burial | 1302–49 |
| Interpretation of individual | Laity: adult benefactor? |
| Grave shape and dimensions (m) | Rectangular with rounded corners: 1.78 by 0.49, 0.25 deep (originally *c*. 0.5) |
| Burial position | South-southwest–north-northeast, extended, supine. Skull missing. The upper limbs were flexed at the elbow to bring the hands around the opposite sides of the lower thorax. The lower limbs were straight, drawn at the ankles |
| Associated items | None |
| Age | Young middle adult: 26–35 years |
| Morphological sex | Male |
| Genetic sex | XY: male |
| MtDNA | No data |
| Stature (cm) | 169.94 +/- 3.27 |
| Percentage of skeleton present | *c*. 85. Truncation at humeri and skull by construction in 1908–9 |
| Preservation grade | 0: high |
| Fragmentation grade | 1: low |
| Other taphonomic alteration | None observed |
| Trauma | None observed |
| Pathology | Probable infectious disorder (bilateral striated periosteal reaction over the femorae and the interosseous surface of right tibia with associated lesions over the anterodistal aspects of the left and right tibiae. Possible treponemal or osteitis infection) |
| Other skeletal observations | Activity marker (increased expression for the deltoid muscle attachment on the left clavicle over the right. The muscle is responsible for flexion and internal rotation of the arm at the shoulder) |
| Dental pathology | None observed |

| *p* | *p* | *p* | *p* | *p* | *p* | *p* | */* | *p* | *p* | *p* | *p* | *p* | *p* | *p* | *p* |
| --- | --- | --- | --- | --- | --- | --- | --- | --- | --- | --- | --- | --- | --- | --- | --- |
| 1 | 2 | 3 | 4 | 5 | 6 | 7 | 8 | 9 | 10 | 11 | 12 | 13 | 14 | 15 | 16 |
| 32 | 31 | 30 | 29 | 28 | 27 | 26 | 25 | 24 | 23 | 22 | 21 | 20 | 19 | 18 | 17 |
| *p* | *p* | *p* | *p* | *p* | *p* | */* | */* | */* | *p* | *p* | *p* | *--* | *--* | *--* | *--* |

| **F.195, skeleton 1355**, Figure S10 | |
| --- | --- |
| Date of burial | 1290–1349 |
| Interpretation of individual | Unknown |
| Grave shape and dimensions (m) | Rectangular with rounded corners: 0.9+ by 0.50, 0.64 deep (originally *c*. 0.7 deep) |
| Burial position | South-southwest–north-northeast, extended, supine. The lower limbs were straight, together at the ankles |
| Associated items | None |
| Age | Adult: 18+ years |
| Morphological sex | Indeterminate |
| Genetic sex | Not sampled |
| MtDNA | Not sampled |
| Stature (cm) | Not calculable |
| Percentage of skeleton present | *c*. 26. Bilateral truncation at proximal femur by construction in 1908–9 |
| Preservation grade | 0: high |
| Fragmentation grade | 1: low |
| Other taphonomic alteration | None observed |
| Trauma | None observed |
| Pathology | None observed |
| Other skeletal observations | Activity markers (enthesophyte formations on anterior table of the patellae and over the calcaneal tuberosities) |
| Dental pathology | Skull absent |

| **F.196, skeleton 1353**, Figure S11 | |
| --- | --- |
| Date of burial | 1302–49 |
| Interpretation of individual | Unknown |
| Grave shape and dimensions (m) | Rectangular with rounded corners: 1.6+ by 0.60, 0.65 deep (originally *c*. 0.7 deep) |
| Burial position | South-southwest–north-northeast, extended, supine. The lower limbs were straight, together at the ankles. |
| Associated items | None |
| Age | Adult: 18+ years |
| Morphological sex | Probably male |
| Genetic sex | Not sampled |
| MtDNA | Not sampled |
| Stature (cm) | 169.94 +/- 3.27 |
| Percentage of skeleton present | *c*. 37. Axial and upper appendicular elements missing. Truncated by construction of friary building |
| Preservation grade | 0: high |
| Fragmentation grade | 1: low |
| Other taphonomic alteration | None observed |
| Trauma | None observed |
| Pathology | Degenerative change in the hips |
| Bilateral porosity around the fovea capitis of the femorae | |
| Other skeletal observations | Bilateral hallux valgus (bilateral medial deviation of the 1st proximal phalanx of the feet) |
| Dental pathology | Skull absent |

| **F.198, skeleton 1364**, Figure S11 | |
| --- | --- |
| Date of burial | 1302–49 |
| Interpretation of individual | Laity: adult benefactor |
| Grave shape and dimensions (m) | Rectangular with rounded corners: 1.82+ by 0.52, 0.60 deep (originally *c*. 0.7 deep) |
| Burial position | South-southwest–north-northeast, extended, supine, upper limbs crossed over abdomen. The lower limbs were straight, drawn at the ankles |
| Associated items | None |
| Age | Mature adult: 46+ years |
| Morphological sex | Male |
| Genetic sex | Not sampled |
| MtDNA | Not sampled |
| Stature (cm) | 180.89 +/- 3.27 |
| Percentage of skeleton present | *c*. 85. Skull absent. Truncated by construction of friary building. |
| Preservation grade | 0: high |
| Fragmentation grade | 1: low |
| Other taphonomic alteration | None observed |
| Trauma | None observed |
| Pathology | Degenerative changes in the back, knees and ankles (marginal lipping between C6–C7; extensive spicule formation between T5–T6; new bone buttressing from T7–T12. Sharp marginal lipping around left and right femoral condyles and along anterior border of the left tibial talar facet. Related posterolateral lipping of both calcanei and tali. Schmorl’s nodes from inferior T5–superior T12 and superior L1–superior L3)  Age related change in the thorax (ossified costochondral cartilage between 1st ribs and manubrium)  Possible overuse activity, shin splint (periosteal reaction over the anteromedial surface of the left tibia. This has a varied and complex aetiology. However, as a unilateral, isolated pathology, it may relate to an inflammatory response to microtrauma. The location of the reaction is diagnostically concordant with medial tibial stress syndrome, where compressive cyclic loads cause oblique microcracks to develop along the planes of high shear stress (Franklyn and Oakes 2015). However, radiography is required to confirm this. Differentially, this is a non-specific periosteal inflammatory response to either infection and or trauma (Weston 2008) |
| Other skeletal observations | Activity markers (enthesophyte formations on anterior table of the patellae, the tuberosities of the tibiae and calcanei tuberosities)  Possible vertebral stenosis (enthesophyte expression for the ligamentum flavum insertion (para-articular processes) on T5, T7 and T8–T12 vertebrae)  Developmental disorder (symphalangism of a left pedal distal interphalangeal joint) |
| Dental pathology | Skull absent |

| **F.199, skeleton 1364**, Figure S12 | |
| --- | --- |
| Date of burial | 1290–1349 |
| Interpretation of individual | Unknown |
| Grave shape and dimensions (m) | Rectangular with rounded corners: 0.7+ by 0.48, 0.93 deep (originally *c*. 1.0 deep) |
| Burial position | South-southwest–north-northeast, extended, supine. The lower limbs were straight, drawn at the ankles. |
| Associated items | None |
| Age | Adult: 18+ years |
| Morphological sex | Indeterminate |
| Genetic sex | Not sampled |
| MtDNA | Not sampled |
| Stature (cm) | Not calculable |
| Percentage of skeleton present | *c*. 22. Bilateral truncation at distal femur by construction in 1908–9 |
| Preservation grade | 0: high |
| Fragmentation grade | 1: low |
| Other taphonomic alteration | None observed |
| Trauma | None observed |
| Pathology | None observed |
| Other skeletal observations | None |
| Dental pathology | Skull absent |

| **F.215, skeleton 1425**, Figure S13 | |
| --- | --- |
| Date of burial | 1290–1349 |
| Interpretation of individual | Unknown |
| Grave shape and dimensions (m) | Rectangular with rounded corners: 0.9+ by 0.45, 0.76 deep (originally *c*. 0.8 deep) |
| Burial position | South-southwest–north-northeast, extended, supine. The lower limbs were straight, with the feet resting dorsiflexed against the grave cut |
| Associated items | None |
| Age | Mature adult: 46+ years |
| Morphological sex | Male |
| Genetic sex | Not sampled |
| MtDNA | Not sampled |
| Stature (cm) | Not calculable |
| Percentage of skeleton present | *c*. 25. Bilateral truncation at mid-femur by construction in 1908–9 |
| Preservation grade | 0: high |
| Fragmentation grade | 1: low |
| Other taphonomic alteration | None observed |
| Trauma | None observed |
| Pathology | Gout (possible inflammatory arthritis of the right foot intermediate phalanx) |
| Other skeletal observations | None |
| Dental pathology | Skull absent |

| **F.216, skeleton 1428**, Figure S14 | |
| --- | --- |
| Date of burial | 1300–49 |
| Interpretation of individual | Adult friar |
| Grave shape and dimensions (m) | Rectangular with rounded corners: 1.90 by 0.71, 0.70 deep (originally *c*. 0.8 deep) |
| Burial position | South-southwest–north-northeast, extended, supine. The upper limbs were slightly abducted at the shoulder and flexed at the elbow to bring the left hand around the right side of the abdomen and the right hand over the left thorax. The lower limbs were straight, drawn at the ankles |
| Associated items | Copper alloy girdle buckle, type 1 [1430], located over the L5 vertebra and pointing to the right. Oval or D-shaped lipped frame with V-shaped notch for the pin and slightly offset opposing bar. The plate is formed from folded rectangular copper alloy sheet, with a rectangular slot for the pin. The plate is recessed for the frame. There is an oval aperture with groove on the reverse and obverse at the end of the slightly tapering plate. This has two small rivets at the back end, but there is also an additional, later, centrally placed crude rivet probably to affect a repair. This appears to have a plate normally associated with frames with forked spacers. 47x30mm, 13g, typologically fourteenth century. Leather strap 20mm wide, 3mm thick  Oyster shell, possibly deliberately pierced |
| Age | Old middle adult: 36–45 years |
| Morphological sex | Male |
| Genetic sex | Not sampled |
| MtDNA | Not sampled |
| Stature (cm) | 180.89 +/- 3.27 |
| Percentage of skeleton present | *c*. 85. Skull absent. Truncated by construction of friary building. |
| Preservation grade | 0: high |
| Fragmentation grade | 1: low |
| Other taphonomic alteration | Copper alloy staining over anterior surface of T12. This cannot relate to the girdle buckle, which is over L5. There is no obvious cause for the staining |
| Trauma | None observed |
| Pathology | Spina Bifida Occulta (incomplete fusion of S1 neural arch, hyperkyphotic S4–S5 (near 90°)  Benign bone tumours (distal phalanges of both feet have subungual exostosis) |
| Other skeletal observations | Normal developmental variation of the spine, aetiology unknown (lateral deviation away from midline of the thoracic spinous processes: T5,T6, T8,T9 towards left, T7 towards right. Although this is considered a normal developmental variation of the spine (Cramer and Darby 2014) it is worth noting that the muscles that attach to here are the ascending part of the trapezius muscle (draws scapula inferiomedially), latissimus dorsi (medial rotation, adduction and extension of the arm at the shoulder), the erector spinae muscles (extension and lateral flexion of the spine) and the transversospinalis muscles (extension, lateral flexion and rotation of the spine)  Possible activity related adaptation (small, superiorly pointing enthesophyte on the odontoid process of C2 (involving alar ligament)  Developmental disorder (bilateral symphalangism (ankylosis) of a single pedal distal interphalangeal joint: (possibly the small toes) |
| Dental pathology | Skull absent |

| **F.217, skeleton 1432**, Figure S15 | |
| --- | --- |
| Date of burial | 1290–1349 |
| Interpretation of individual | Unknown |
| Grave shape and dimensions (m) | Rectangular with rounded corners: 1.0+ by 0.55, 0.53 deep (originally *c*. 0.6 deep) |
| Burial position | South-southwest–north-northeast, extended, supine, the lower limbs were straight, drawn at the ankles. |
| Associated items | None |
| Age | Adult: 18+ |
| Morphological sex | Indeterminate |
| Genetic sex | Not sampled |
| MtDNA | Not sampled |
| Stature (cm) | Not calculable |
| Percentage of skeleton present | *c*. 25. Bilateral truncation at proximal femur by construction in 1908–9 |
| Preservation grade | 0: high |
| Fragmentation grade | 1: low |
| Other taphonomic alteration | None observed |
| Trauma | None observed |
| Pathology | Gout (exostosis, sharp lipping and cortical thinning over and around the distal articular surface of the right 1st metatarsal. Microporosity over right calcaneus and distal end of the first proximal phalanx) |
| Other skeletal observations | Activity marker (enthesophytes along medial edge of left linea aspera of femur corresponding to the insertions of adductor magnus and adductor longus (flexes, adducts and medially rotates the thigh at the hip)  Possible ancestry trait (notable anterior curvature of both femora) |
| Dental pathology | Skull absent |

| **F.232, skeleton 1482**, Figure S16 | |
| --- | --- |
| Date of burial | 1320–49 |
| Interpretation of individual | Laity: adult benefactor |
| Grave shape and dimensions (m) | Rectangular with rounded corners: 2.30 by 0.60, 0.70 deep (originally *c*. 1.2 deep) |
| Burial position | South-southwest–north-northeast, extended, supine, the skull was in neutral position. The upper limbs were slightly abducted at the shoulder and flexed at the elbow to bring the hands right over left over the sternum. The lower limbs were straight, drawn at the ankles |
| Associated items | None |
| Age | Younger old middle adult: earlier part of 36–45 years |
| Morphological sex | Male |
| Genetic sex | XY: male |
| MtDNA | No data |
| Stature (cm) | 167.32 +/- 3.27 |
| Percentage of skeleton present | *c*. 99 (effectively complete) |
| Preservation grade | 0: high |
| Fragmentation grade | 1: low |
| Other taphonomic alteration | None observed |
| Trauma | Tooth extraction (possible tooth extraction and associated trauma of (29): full alveolar resorption with a healed residual bone spur)  Fractured coccyx (abnormal sacrococcygeal angle likely relating to a healed flexion fracture of the coccyx) |
| Pathology | Possible anaemic disorder (bilateral Cribra Orbitalia, Porotic Hyperostosis over palatine process, glabella & occipital bone; bilateral macroporosity (1–1.5mm) inferior to frontal temporal lines)  Age related degeneration in the shoulders (bilateral porosity around inferior margins of glenoid fossa; minor lipping around inferior margin of right glenoid fossa with associated inferoposterior porosity of right humeral head margin)  Spina Bifida Occulta (non-fusion of S1 sacral laminae)  Degeneration in the hips (bilateral plaque form defects around the anterior side of the femoral heads and necks: Type B (after Hack et al. 2010) (femoroacetabular cam impingement)  Possible gout (bilateral extstoses over the first proximal foot phalanges) |
| Other skeletal observations | Possible vertebral stenosis (notable expression of the para-articular processes between T4–T10)  Activity markers at the right elbow and left collar bone (right elbow moderate superiorly pointing enthesophytes on right ulna olecranon. Asymmetric collar bones: the left clavicle exhibits a and larger acromial end and a deeper impression for the costoclavicular ligament) |
| Dental pathology | Tooth loss (ante-mortem tooth loss of (29))  Mineralised dental plaque (slight lingual and buccal dental calculus) |

| *ne* | *p* | *cl* | *p* | *p* | *cl* | *cl* | *cl* | *cl* | *cl* | *cl* | *cl* | *cl* | *cl* | *p* | *ne* |
| --- | --- | --- | --- | --- | --- | --- | --- | --- | --- | --- | --- | --- | --- | --- | --- |
| 1 | 2 | 3 | 4 | 5 | 6 | 7 | 8 | 9 | 10 | 11 | 12 | 13 | 14 | 15 | 16 |
| 32 | 31 | 30 | 29 | 28 | 27 | 26 | 25 | 24 | 23 | 22 | 21 | 20 | 19 | 18 | 17 |
| *s* | *p* | *p* | *x* | *p* | *s* | *cl* | *cl* | *cl* | *cl* | *cl* | *cl* | *cl* | *cl* | *cl* | *ne* |

| **F.237, skeleton 1516**, Figure S17 | |
| --- | --- |
| Date of burial | 1290–1360/1420 |
| Interpretation of individual | Unknown |
| Grave shape and dimensions (m) | Rectangular with rounded corners: 0.98 by 0.52, 0.78 deep (originally *c*. 0.9 deep) |
| Burial position | South-southwest–north-northeast, extended, supine. The lower limbs were straight, drawn at the ankles |
| Associated items | None |
| Age | Younger old middle adult: earlier part of 36–45 years |
| Morphological sex | Male |
| Genetic sex | Not sampled |
| MtDNA | Not sampled |
| Stature (cm) | 167.56 +/- 3.27 |
| Percentage of skeleton present | *c.* 35. Truncated at pelvis by construction in 1908–9 |
| Preservation grade | 0: high |
| Fragmentation grade | 1: low |
| Other taphonomic alteration | None observed |
| Trauma | None observed |
| Pathology | Avascular necrosis (osteonecrosis) of the right femoral head (sub-circular area of bone resorption exposing underlying trabecular structure superior to the fovea capitis. There are several risk factors to this disease all of which disrupt the blood supply to the femoral head: trauma, secondary conditions of hypercoagulation such as alcoholism, hematologic, metabolic (gout) diseases and other risk factors such as smoking can all be responsible (Malizos et al. 2007) plus others. That there is no evidence for fracture or gout, alcohol abuse may not be an unreasonable suggestion. Hip pain is typically the first symptom. This may lead to a dull ache or throbbing pain in the groin or buttock area) |
| Other skeletal observations | None |
| Dental pathology | Skull absent |

| **F.265, skeleton 1602**, Figure S18 | |
| --- | --- |
| Date of burial | 1320–49 |
| Interpretation of individual | Adult friar |
| Grave shape and dimensions (m) | Rectangular with rounded corners: 2.20 by 0.62, 0.53 deep (originally *c*. 0.8 deep) |
| Burial position | South-southwest–north-northeast, extended, supine, the skull was slightly rotated towards the right shoulder. The upper limbs were flexed at the elbow to bring the hands around the opposite sides of the thorax. The lower limbs were straight, drawn at the ankles |
| Associated items | Copper alloy girdle buckle, type 1 [1638] located over the pubic arch of the pelvis and pointing to the right. Oval, lipped frame. The pin and rectangular plate are intact. The frame has a V-shaped seat or notch for the pin on the outside edge with the opposing bar slightly offset to the back end of the frame. The plate is formed of a folded sheet with a rectangular slot for the pin and two rivets at the end of the plate. The pin is flanged with a transverse ridge. Little evidence of use wear. 38x24mm, 9g, typologically mid-fourteenth–mid fifteenth century. Leather strap 15–18mm wide, 3mm thick |
| Age | Younger old middle adult: earlier part of 36–45 years |
| Morphological sex | Male |
| Genetic sex | XY: male |
| MtDNA | H1at1 |
| Stature (cm) | 176.84 +/- 3.27 |
| Percentage of skeleton present | *c*. 98 (effectively complete) |
| Preservation grade | 0: high |
| Fragmentation grade | 1: low |
| Other taphonomic alteration | None observed |
| Trauma | None observed |
| Pathology | Mid foot arthritis (large periarticular osteophyte affecting the left naviculocuniform articulation) |
| Other skeletal observations | Activity marker (enthesophyte formations on anterior table of left patella)  Benign bone tumour (a solitary inferiorly pointed osteochondroma (spur) on the medio-proximal metaphysis of the right tibia. Possibly caused Pes Anserinus Syndrome) |
| Dental pathology | Mineralised dental plaque (slight lingual/buccal dental calculus) |

| p | p | p | p | p | p | p | p | p | p | p | p | p | p | p | p |
| --- | --- | --- | --- | --- | --- | --- | --- | --- | --- | --- | --- | --- | --- | --- | --- |
| 1 | 2 | 3 | 4 | 5 | 6 | 7 | 8 | 9 | 10 | 11 | 12 | 13 | 14 | 15 | 16 |
| 32 | 31 | 30 | 29 | 28 | 27 | 26 | 25 | 24 | 23 | 22 | 21 | 20 | 19 | 18 | 17 |
| cl | cl | cl | cl | p | p | p | p | p | p | cl | cl | p | p | cl | cl |

| **F.302, skeleton 1735**, Figure S19 | |
| --- | --- |
| Date of burial | 1300–1360/1420 |
| Interpretation of individual | Adult friar |
| Grave shape and dimensions (m) | Rectangular with rounded corners: 1.90 by 0.62, 0.83 deep (originally *c*. 0.9 deep) |
| Burial position | South-southwest–north-northeast, extended, supine, the skull was in neutral position. The upper limbs were slightly abducted at the shoulder and flexed at the elbow to bring the left hand around right side of the thorax and the right hand over right chest. The lower limbs were straight, drawn at the ankles |
| Associated items | Copper alloy girdle buckle, type 5 [1737] located centrally over the S1 of the sacrum and pointing to the right. Two fragments of an annular buckle, with partial pin attached. Heavily distorted and fragmentary. Diameter approximately 35mm, 7g. Found with a copper alloy rectangular mount of shallow pyramidal form, with a single integral rivet, attached to a double layer of leather strap. 11x12mm, 2g, typologically fourteenth–early fifteenth century. Leather strap 17mm wide, 2–3mm thick |
| Age | Older old middle adult: later part of 36–45 years. |
| Morphological sex | Probably male |
| Genetic sex | XY: male |
| MtDNA | H3 |
| Stature (cm) | 175.41 +/- 3.27 |
| Percentage of skeleton present | *c*. 98 (effectively complete) |
| Preservation grade | 0: high |
| Fragmentation grade | 1: low |
| Other taphonomic alteration | Copper alloy staining over anterior surface of sacrum, from girdle buckle |
| Trauma | None observed |
| Pathology | Age related degeneration in the middle of the back, right elbow and right foot (back: spondylophyte on right anterior side of T8 body. Right elbow: marginal lipping around the posterior margin of the right radial tuberosity. Right foot: periarticular osteophytes around the talus, calcaneus and navicular bones) |
| Other skeletal observations | Age related degeneration in the neck or ‘pseudogout’ (anteriorly directed enthesophyte on odontoid process of C2)  Activity markers (medially directed enthesophytes over lesser trochanter and linea aspera of left femur, corresponding to the insertions of adductor magnus and adductor longus (flexes, adducts and medially rotates the thigh at the hip). Inferiorly pointing enthesophytes over anterior table of left patella and associated superiorly pointing enthesophytes over the left tibial tuberosity. Asymmetric, not as evident on right side) |
| Dental pathology | Mineralised dental plaque (considerable labial/lingual/buccal dental calculus) |

| *p* | *p* | *p* | *cl* | *cl* | *cl* | *cl* | *p* | *cl* | *cl* | *cl* | *cl* | *cl* | *p* | *cl* | *cl* |
| --- | --- | --- | --- | --- | --- | --- | --- | --- | --- | --- | --- | --- | --- | --- | --- |
| 1 | 2 | 3 | 4 | 5 | 6 | 7 | 8 | 9 | 10 | 11 | 12 | 13 | 14 | 15 | 16 |
| 32 | 31 | 30 | 29 | 28 | 27 | 26 | 25 | 24 | 23 | 22 | 21 | 20 | 19 | 18 | 17 |
| *cl* | *p* | *cl* | *cl* | *cl* | *cl* | *cl* | *cl* | *cl* | *cl* | *p* | *cl* | *cl* | *cl* | *cl* | *cl* |

| **F.309, skeleton 1767 plus disarticulated 1766**, Figure S20 | |
| --- | --- |
| Elements present | This skeleton was truncated by later features, and it appears that disturbed elements [1766] were placed above the original skeleton [1767]. This probably took place when Post-Dissolution pit F.102 was dug  [1767]: articulated component comprising left shoulder girdle, left arm, left ribs (partial) and left os coxae  [1766]: disarticulated component comprising skull, right scapula, right arm, hands (partial), right ribs (partial), 7 cervical, 6 thoracic, 4 lumbar vertebrae, sacrum, right os coxae, both femora, both tibiae, both fibulae, feet (partial)  The absent elements are the right clavicle, manubrium, sternum and both patellae |
| Date of burial | 1290–1360 |
| Interpretation of individual | Unknown |
| Grave shape and dimensions (m) | Rectangular with rounded corners: 1.88 by 0.49, 0.98 deep (originally *c*. 1.1 deep) |
| Burial position | South-southwest–north-northeast, extended, supine, left arm crossed over the thorax |
| Associated items | None |
| Age | Young middle adult: 26–35 years |
| Morphological sex | Male |
| Genetic sex | Consistent with XY but not XX: male |
| MtDNA | V3a1 |
| Stature (cm) | 171.13 +/- 3.27 |
| Percentage of skeleton present | *c*. 70 including disarticulated. Truncated by later burial |
| Preservation grade | 0: high |
| Fragmentation grade | 1: low |
| Other taphonomic alteration | None observed |
| Trauma | None observed |
| Pathology | Probable infection of the scalp, secondary to severe ringworm (reactive porotic and sclerotic bone over the superoposterior aspect of the parietals and occipital planum. Sclerosis over right parietal is associated with multiple mediolateral sulci) |
| Other skeletal observations | Activity marker (large/elongated enthesophyte directed inferiorly from the occipital protuberance) |
| Dental pathology | Mineralised dental plaque (light labial dental calculus)  Trauma (occlusal chip to (9) and (10))  Systemic disorder (enamel hypoplasia: single furrow form defect on (6) (7) (9) (10) (11)) |

| p | p | ca | / | p | cl hy | cl hy | / | t hy | t hy | cl hy | cl | p | p | p | p |
| --- | --- | --- | --- | --- | --- | --- | --- | --- | --- | --- | --- | --- | --- | --- | --- |
| 1 | 2 | 3 | 4 | 5 | 6 | 7 | 8 | 9 | 10 | 11 | 12 | 13 | 14 | 15 | 16 |
| 32 | 31 | 30 | 29 | 28 | 27 | 26 | 25 | 24 | 23 | 22 | 21 | 20 | 19 | 18 | 17 |
| / | p | ca | / | / | / | / | / | / | / | p | / | p | p | p | / |

| **F.311, skeleton 1782**, Figure S21 | |
| --- | --- |
| Date of burial | 1290–1349 |
| Interpretation of individual | Adult friar |
| Grave shape and dimensions (m) | Rectangular with rounded corners: 2.09 by 0.58, 0.46 deep (originally *c*. 0.6 deep) |
| Burial position | South-southwest–north-northeast, extended, supine, the skull was in neutral position. The upper limbs were flexed at the elbows to bring the hands around the opposite sides of the abdomen. The lower limbs were straight, drawn at the ankles |
| Associated items | Iron girdle buckle, type 4 [1788] located over the upper wing of the left ilium of the os coxae and pointing to the right. Circular frame and pin in poor condition. 41mm diameter, 18g, typologically High/Late Medieval. Leather strap width unknown 3–4mm thick |
| Age | Mature adult: 46+ years. Degenerative characteristics suggest this may have been an old adult (60+ years) |
| Morphological sex | Male |
| Genetic sex | XY: male |
| MtDNA | X3 |
| Stature (cm) | 176.84 +/- 3.27 |
| Percentage of skeleton present | *c*. 98 (effectively complete) |
| Preservation grade | 0: high |
| Fragmentation grade | 1: low |
| Other taphonomic alteration | None observed |
| Trauma | Right rib fractures (non-united fracture of tenth or eleventh right rib; misaligned healed mid shaft fracture of 6th (?) right rib; overlapped healed mid shaft fracture of right 5th or 7th right rib) |
| Pathology | Osteoarthritis in the back, pectoral girdle, left elbow and right knee (Back: eburnation of the anterior surface of the odontoid process of C2. Ankylosis between left articular facets and left bodies of C2–C4; large spondylophytes on the left side of T8–T11 bodies; flowing spondylophytes (DISH) and body fusion between T11–T12, preserving intervertebral space. Pectoral girdle: bilateral marginal lipping of the glenoid fossae. Bilateral macroporosity of the acromioclavicular joints. Macroporosity and eburnation of the right sternoclavicular joint. Left elbow: eburnation within the left humeroradial joint. Marginal lipping around the left radial and ulna heads. Right knee: Eburnation over the patellar surface of right femoral lateral condyle and associated lateral facet of the right patella) |
| Other skeletal observations | Activity markers in the left elbow and left knee (Left elbow: eburnation within the left humeroradial joint. Marginal lipping around the left proximal radial and ulna heads. Left patellae: superiorly/inferiorly directed enthesophyte formations on anterior table of the left patellae)  Hole in the sternum (sternal foramen 5.31mm diameter)  Accessory transverse foramen (left side of C5) |
| Dental pathology | Mineralised dental plaque (slight/moderate labial/lingual/buccal dental calculus)  Tooth decay (interproximal caries (14)) |

| p | p | p | p | p | p | p | cl | / | p | cl | b r | cl | cl ca | cl | p |
| --- | --- | --- | --- | --- | --- | --- | --- | --- | --- | --- | --- | --- | --- | --- | --- |
| 1 | 2 | 3 | 4 | 5 | 6 | 7 | 8 | 9 | 10 | 11 | 12 | 13 | 14 | 15 | 16 |
| 32 | 31 | 30 | 29 | 28 | 27 | 26 | 25 | 24 | 23 | 22 | 21 | 20 | 19 | 18 | 17 |
| ne | p | p | p | cl | / | p | cl | p cl | cl | cl | p | p | p | cl | ne |

| **F.312, skeleton 1785 and disturbed material 1785 and 1972**, Figure S22 | |
| --- | --- |
| Elements present | While most of the skeleton [1785] was in its original position the cranium had been displaced by compaction of an earlier pit [1972] and other elements had been disturbed [1785]: articulated elements sternebrae, 6 thoracic vertebrae, 4 lumbar vertebrae, left and right humerus, left radius epiphyses (distal/proximal) right radial epiphysis, left and right ulna, hands (partial), both femora, left tibia, both fibulae  [1784]: disturbed elements comprising mandible, manubrium, 5 cervical vertebrae, 3 thoracic vertebrae, 2 left and 2 right rib head fragments, right distal radius, both clavicles, hands (partial), right tibia, feet (partial)  [1972]: disturbed elements of the cranium  Elements absent with no evidence for truncation: patellae, intermediate and distal phalanges of the feet |
| Date of burial | 1302–49 |
| Interpretation of individual | Laity: child of benefactor? |
| Grave shape and dimensions (m) | Rectangular with rounded corners: 2.00 by 0.60, 0.62 deep (originally *c*. 0.7 deep) |
| Burial position | South-southwest–north-northeast, extended, supine, the skull had moved away from the rest of the skeleton after death. The left arm was straight against the body to bring the hand around and under the left thigh. The right arm was slightly flexed at the elbow over the right hip. The lower limbs were straight, drawn at the knees |
| Associated items | None |
| Age | Adolescent: 13–18 years |
| Morphological sex | Possibly male |
| Genetic sex | XY: male |
| MtDNA | No data |
| Stature (cm) | Too young to calculate |
| Percentage of skeleton present | *c*. 98 (effectively complete) |
| Preservation grade | 1: high |
| Fragmentation grade | 1: low |
| Other taphonomic alteration | None observed |
| Trauma | Cut mark (located on the posterior surface of the right tibia, proximal to the soleal line) |
| Pathology | Probable bacterial infection (tuberculosis) affecting the upper limbs, ribs the lower half of back, hip and possibly the right shin (upper limbs and ribs: bilateral periosteal reaction of the humeral deltoid tuberosities and five sternal rib ends; tubercular osteomyelitis (cold abscesses) over right humeral supracondylar crest, the pronator ridge of left ulna and associated lateral surface of left radius. Lower back: hypervascularization of the thoracic vertebrae. Lumbar vertebrae less affected. Hip: bilateral cold abscesses over sacral tuberosities and the ventral surfaces of S3–S5; bilateral hypervascularisation/cortical discontinuity around the anteromedial aspect of the femoral necks. Right shin: periosteal reaction on the mid medial border of the right tibia) |
| Other skeletal observations | None |
| Dental pathology | Heritable trait (buccal pits on (31), (19), (18)) |

| p | p | p | p | p | p | p | p | p | p | a | p | p | p | p | p |
| --- | --- | --- | --- | --- | --- | --- | --- | --- | --- | --- | --- | --- | --- | --- | --- |
| 1 | 2 | 3 | 4 | 5 | 6 | 7 | 8 | 9 | 10 | 11 | 12 | 13 | 14 | 15 | 16 |
| 32 | 31 | 30 | 29 | 28 | 27 | 26 | 25 | 24 | 23 | 22 | 21 | 20 | 19 | 18 | 17 |
| / | p | p | p | p | / | p | p | p | p | / | / | p | p | p | / |

| **F.314, skeleton 1797**, Figure S23 | |
| --- | --- |
| Date of burial | 1320–49 |
| Interpretation of individual | Young adult friar, based on age at death possibly studying for advanced degree |
| Grave shape and dimensions (m) | Rectangular with rounded corners: 2.05 by 0.55, 0.80 deep (originally *c*. 0.9 deep) |
| Burial position | South-southwest–north-northeast, extended, supine, the skull was rotated slightly towards the right shoulder. The upper limbs were flexed to bring hands crossed over upper thorax (manubrium). The lower limbs were straight, drawn at the ankles |
| Associated items | Elephant ivory girdle buckle, type 1 [1803] located over the right side of S2 of the sacrum and pointing to the left. Skilfully produced near complete buckle with integral plate, made from elephant ivory with Schreger lines visible. Oval frame with a tongue groove and moulded lateral knops in front of the tongue rest. The integral buckle plate is rectangular and is bifurcated along most of its length, allowing a strap to be secured with two rectangular headed copper alloy rivets. The copper alloy buckle pin is secured on a shaft, also of copper alloy, which runs laterally through most of the buckle from one side, but does not emerge on the other side. The upper surface of the buckle is lightly embellished with spaced triangular notches set between three sets narrow parallel lines, providing a cable-like effect. Oval lipped frames occur in London contexts within ceramic phases 11–12, and predominantly in phase 11, of *c*. 1350–1400 (Egan and Pritchard 2002, 70, 74–5). Buckles made from elephant ivory form a much more exclusive commodity than those made of bone, with a specific provenance. Late Medieval buckles of this material are likely to have been made in France, and most likely in Paris, a centre of medieval elephant ivory production. From the late thirteenth century onwards, Paris and other workshops in France were major producers of objects of elephant ivory, whilst comparatively little was produced in this material elsewhere in northern Europe, or in England (Gaborit-Chopin 2003, 21–2; Stratford 1987, 108–9). In a French text of *c.* 1260 that describes Parisian workshops for ivory, as well as for the working of bone, horn and other materials, the patenostriers are listed as producing buttons and buckles, as well as rosary beads (Gaborit-Chopin 2003, 266–9; MacGregor 1991, 377). Accordingly, it seems likely that the ivory buckle was made in a French workshop, probably in Paris, before eventually finding its way to Cambridge |
| Age | Younger young adult: earlier part of 18–25 years |
| Morphological sex | Male |
| Genetic sex | XY: male |
| MtDNA | H1g1 |
| Stature (cm) | 179.46 +/- 3.27 |
| Percentage of skeleton present | *c*. 98 (effectively complete) |
| Preservation grade | 0: high |
| Fragmentation grade | 1: low |
| Other taphonomic alteration | None observed |
| Trauma | None observed |
| Pathology | Systemic stress indicator (bilateral cribra orbitalia)  Degeneration of the back (Schmorl's nodes between T5–T11. Lumbosacral Transitional Vertebrae (LSTV). Lumbarization of the sacrum: S1 and S2 bodies are non-fused; S1 has a defined spinous process with complete sagittal cleft (spina bifida occulta) |
| Other skeletal observations | Developmental anomaly/possible trauma (ankylosis between T3–T4 neural arches, bodies are unaffected with no associated pathology or morphological variation. Likely congenital |
| Geometric morphometrics | Greatest left-directional asymmetry (13%) of any individual studied, which may explain the atypical buckle direction. For greater detail see Cessford *et al*. forthcoming |
|  |  |
| Dental pathology | Mineralised dental plaque (slight lingual dental calculus) |

| p | p | p | p | p | p | p | p | p | cl | p | p | p | p | p | p |
| --- | --- | --- | --- | --- | --- | --- | --- | --- | --- | --- | --- | --- | --- | --- | --- |
| 1 | 2 | 3 | 4 | 5 | 6 | 7 | 8 | 9 | 10 | 11 | 12 | 13 | 14 | 15 | 16 |
| 32 | 31 | 30 | 29 | 28 | 27 | 26 | 25 | 24 | 23 | 22 | 21 | 20 | 19 | 18 | 17 |
| p | p | p | p | p | cl | cl | cl | cl | cl | cl | p | p | p | p | p |

| **F.315, skeleton 1800**, Figure S24 | |
| --- | --- |
| Date of burial | 1302–49 |
| Interpretation of individual | Probably laity: adult benefactor? |
| Grave shape and dimensions (m) | Rectangular with rounded corners: 2.03 by 0.61, 0.72 deep (originally *c*. 0.8 deep) |
| Burial position | South-southwest–north-northeast, extended, supine, the skull was in neutral position. The left arm was straight against the body to bring the hand next to the left hip. The right arm was straight, truncated at the elbow. The left leg was straight. The right os coxae and lower limb were truncated |
| Associated items | None |
| Age | Young adult: 18–25 years |
| Morphological sex | Male |
| Genetic sex | Consistent with XY but not XX: male |
| MtDNA | W |
| Stature (cm) | 168.51 +/- 3.27 |
| Percentage of skeleton present | *c*. 73. Truncated by later burial |
| Preservation grade | 1: high |
| Fragmentation grade | 1: low |
| Other taphonomic alteration | None observed |
| Trauma | None observed |
| Pathology | Developmental disorder (bilateral elongated styloid processes)  Probable bacterial infection (tuberculosis) (hyperascularization between T9–T11 on left side of bodies)  Osteochondritis dessicans (characterised by a small hamartoma within the proximal facet of the 1st proximal phalanx and an associated round 7.06mm cortical defect exposing trabecular bone in the distal head of the 1st metatarsal. The exact pathophysiology and aetiology of this condition is still unclear though always develops within an articulation. In this individuals case, there is a known association with hallux valgus (Wells 1974). Clinically, it is regarded as an acquired lesion of subchondral bone where ischemia seems to play a role. It is characterised by degrees of osseous resorption, collapse, and sequestrum formation. Thus, causes can range from overuse, trauma and inflammation to genetics and vascular abnormalities (Magee, Zachazewski and Quillen 2009) |
| Other skeletal observations | None |
| Dental pathology | Mineralised dental plaque (slight labial dental calculus)  Heritable trait (bilateral buccal pits on (18) and (31)) |

| p | p | p | p | p | cl | cl | cl | cl | cl | / | p | p | p | p | / |
| --- | --- | --- | --- | --- | --- | --- | --- | --- | --- | --- | --- | --- | --- | --- | --- |
| 1 | 2 | 3 | 4 | 5 | 6 | 7 | 8 | 9 | 10 | 11 | 12 | 13 | 14 | 15 | 16 |
| 32 | 31 | 30 | 29 | 28 | 27 | 26 | 25 | 24 | 23 | 22 | 21 | 20 | 19 | 18 | 17 |
| a | p | p | p | cl | cl | cl | cl | cl | cl | cl | p | p | p | p | a |

| **F.328, skeleton 1824**, Figure S25 | |
| --- | --- |
| Elements present | Relatively shallow burial with extensive generalised later truncation, particularly to lower limbs |
| Date of burial | 1349–1360/1420 |
| Interpretation of individual | Probably laity: child of benefactor? |
| Grave shape and dimensions (m) | Rectangular with rounded corners: 0.90+ by 0.70, 0.38 deep (originally *c*. 0.5 deep) |
| Burial position | South-southwest–north-northeast, extended, supine, the skull was rotated to look over the left shoulder. The upper limbs were abducted and flexed at the elbows to bring the hands over lower abdomen |
| Associated items | None |
| Age | Adolescent: 13–18 years |
| Morphological sex | Possibly male |
| Genetic sex | XY: male |
| MtDNA | H4a1a4b |
| Stature (cm) | 170.24 +/- 4.05 |
| Percentage of skeleton present | *c*. 53. Truncated by construction of friary building |
| Preservation grade | 0: high |
| Fragmentation grade | 1: low |
| Other taphonomic alteration | Truncated at pelvis |
| Trauma | None observed |
| Pathology | Probable infection of the scalp |
| Periosteal reactions confined to the parietal bones | |
| Other skeletal observations | None |
| Dental pathology | None observed |

| -- | -- | -- | -- | -- | -- | -- | -- | -- | -- | -- | -- | -- | -- | -- | -- |
| --- | --- | --- | --- | --- | --- | --- | --- | --- | --- | --- | --- | --- | --- | --- | --- |
| 1 | 2 | 3 | 4 | 5 | 6 | 7 | 8 | 9 | 10 | 11 | 12 | 13 | 14 | 15 | 16 |
| 32 | 31 | 30 | 29 | 28 | 27 | 26 | 25 | 24 | 23 | 22 | 21 | 20 | 19 | 18 | 17 |
| -- | -- | -- | -- | -- | -- | -- | -- | -- | -- | -- | -- | -- | l | e | ne |

| **F.331, skeleton 1873**, Figure S26 | |
| --- | --- |
| Date of burial | 1320–49 |
| Interpretation of individual | Adult friar |
| Grave shape and dimensions (m) | Trapezoidal: 1.18+ by 0.80, 0.73 deep (originally *c*. 0.8 deep) |
| Burial position | South-southwest–north-northeast, extended, supine, the skull was in forward flexion and rotated to look over the right shoulder. The upper limbs were flexed at the elbows and crossed at the forearm to bring left hand over the sacrum and right hand over the left ilium |
| Associated items | Copper alloy girdle buckle, type 3 [1882], located to the right of the L5 vertebra and pointing to the right. Oval or D-shaped, the frame thickens towards the middle and has a rectangular notch or recess for the pin. The opposing bar is slightly offset. The pin is intact and appears to have fused tightly within the frame notch. Similar buckles from London are dated to the fourteenth century (Egan and Pritchard 2002, 70). This buckle and pin display very little use wear. 23x31mm, 9g. A crescent shaped copper alloy mount was found in association with the buckle, attached to the strap by two integral round flatheaded rivets. 19.2x12.3mm, 2g. The lower strap at the mount has a straight end with cropped corners indicating it to be a terminal. The upper strap, closest to the mount, also appears to have slightly tapering sides, the end is now slightly broken but had been straight, which would suggest a second terminal, rather than a join to extend the length of the strap. Width 16mm, 3mm thick, bovine. At the buckle pin the strap is 7mm wide and 3mm thick |
| Age | Old middle adult: 36–45 years |
| Morphological sex | Male |
| Genetic sex | XY: male |
| MtDNA | H23 |
| Stature (cm) | 177.33 +/- 4.05 |
| Percentage of skeleton present | *c*. 63. Truncated by construction of friary building. |
| Preservation grade | 1: high |
| Fragmentation grade | 1: low |
| Other taphonomic alteration | Truncated at the hip |
| Trauma | None observed |
| Pathology | Developmental disorder of the cranium (craniosynostosis with slight ectocranial keel and expression of the sagittal sulcus crests, increasing in intensity towards the cruciform eminence)  Age related degeneration of the right shoulder, right elbow and back (slight marginal lipping around right radial tuberosity, slight modification of the right acromioclavicular articulation and minor spondylophytes on anterior borders of T3–T10 bodies. Schmorl's nodes from T6–T12, L1, L2 and L4)  Spina Bifida Occulta (unfused S1 lamina, left process is inferior to the right. There is associated sacral asymmetry with slight left wedging around the sagittal plane) |
| Other skeletal observations | None |
| Dental pathology | Mineralised dental plaque (slight labial/lingual/buccal dental calculus)  Tooth loss (ante-mortem tooth loss of (17) and (32))  Tooth decay and infection (large occlusal caries (3.33mm diameter) involving (3) and large periapical abscess on lingual and labial surfaces of the alveolar process) |

| p | p | ab ca | p | p | p | p | p | p | p | p | p | p | p | -- | -- |
| --- | --- | --- | --- | --- | --- | --- | --- | --- | --- | --- | --- | --- | --- | --- | --- |
| 1 | 2 | 3 | 4 | 5 | 6 | 7 | 8 | 9 | 10 | 11 | 12 | 13 | 14 | 15 | 16 |
| 32 | 31 | 30 | 29 | 28 | 27 | 26 | 25 | 24 | 23 | 22 | 21 | 20 | 19 | 18 | 17 |
| x | p | p | p | p | cl | cl | / | / | cl | cl | cl | p | cl | p | x |

| **F.332, skeleton 1880**, Figure S27 | |
| --- | --- |
| Date of burial | 1349–1360/1420 |
| Interpretation of individual | Young adult friar, based on age at death possibly studying for advanced degree |
| Grave shape and dimensions (m) | Rectangular with rounded corners: 1.74 by 0.56, 0.46 deep (originally *c*. 0.6 deep) |
| Burial position | South-southwest–north-northeast, extended, supine, the cranium was absent. The left arm was flexed at the elbow to bring the forearm and hand across the lower thorax. The right arm was missing. Both lower limbs were in anatomical position at the femoroacetabular and tibiofemoral joints. Although drawn at the ankles, the lower limbs were rotationally displaced inferior to mid femorae fractures; thus, right side was presented posteromedially and the left was presented posterolaterally |
| Associated items | Copper alloy girdle buckle, type 3 [1879], located over the L4 vertebra and pointing upwards. Oval or D-shaped. The frame thickens towards the middle and has a deep rectangular notch or recess for the pin. Slightly offset and narrowed opposing bar. The pin has moulded, ridged section to the upper surface towards the base. Traces of leather present around the pin. Similar to buckle with F.331. 22x29mm, 10g, typologically fourteenth century. Leather strap 14mm wide, 2–4mm thick |
| Age | Young adult: 18–25 years |
| Morphological sex | Probably male |
| Genetic sex | Not sampled |
| MtDNA | Not sampled |
| Stature (cm) | 160.00 +/- 4.05 |
| Percentage of skeleton present | *c*. 79. Truncated by later pit. |
| Preservation grade | 1: high |
| Fragmentation grade | 1: low |
| Other taphonomic alteration | Cortical flaking and delamination  Copper alloy staining anterior surface of L4, from girdle buckle |
| Trauma | Neck fractures (C6 and T1 both have incomplete body and lamina fractures)  Possible healed collar bone fracture or overuse injury (the lateral (acromial) end of the left clavicle has a contour anomaly within a 20mm area, largely confined to the inferior surface of the bone. This could relate to a chronic overuse injury or a healed fracture (type I after (Holder, Kolla and Lehto 2017)  3). Bilateral leg fractures: bilateral mid shaft fractures to both femurs (see discussion for description) |
| Pathology | None observed |
| Other skeletal observations | None |
| Dental pathology | Skull not present |

| p | p | p | p | l | / | l | l | -- | -- | -- | -- | -- | -- | -- | -- |
| --- | --- | --- | --- | --- | --- | --- | --- | --- | --- | --- | --- | --- | --- | --- | --- |
| 1 | 2 | 3 | 4 | 5 | 6 | 7 | 8 | 9 | 10 | 11 | 12 | 13 | 14 | 15 | 16 |
| 32 | 31 | 30 | 29 | 28 | 27 | 26 | 25 | 24 | 23 | 22 | 21 | 20 | 19 | 18 | 17 |
| -- | -- | -- | -- | -- | b | b | / | b | p | p | p | p | p | p | p |

| **F.333, skeleton 1884**, Figures S28–S29 | |
| --- | --- |
| Date of burial | 1290–1349 |
| Interpretation of individual | Adult friar |
| Grave shape and dimensions (m) | Rectangular with rounded corners: 2.10 by 0.60, 0.74 deep (originally *c*. 0.8 deep) |
| Burial position | South-southwest–north-northeast, extended, supine, the skull was in neutral position. The upper limbs were flexed to bring hands over lower abdomen. The lower limbs were extended |
| Associated items | Iron girdle buckle, type 4 [1899], located on the S1 part of the sacrum and pointing to the right. In poor condition, heavily corroded. Possibly oval or D- shaped frame. 38x33mm, 10g, typologically High/Late Medieval |
| Age | Young middle adult: 26–35 years |
| Morphological sex | Male |
| Genetic sex | XY: male |
| MtDNA | I3 |
| Stature (cm) | 167.56 +/- 3.27 |
| Percentage of skeleton present | *c.* 89. Truncated by construction of friary building |
| Preservation grade | 1: high |
| Fragmentation grade | 1: low |
| Other taphonomic alteration | Occasional dark-grey mottling over vertebrae and temporals |
| Trauma | None observed |
| Pathology | Systemic stress indicator (minor bilateral cribra orbitalia, with greater expression in right. Cribra cranii over supraorbital ridge)  Age related degeneration in the back (minor spondylophytes on anterior borders of T3–T11 and L2–L5 bodies greatest expression between T5–T6) |
| Other skeletal observations | Overuse activity of right knee (enthesophyte formations on anterior table of right patella)  Sprain of the right ankle (minor enthesophytes on the medial epiphysis of the right fibula. This probably relates to a sprain involving the anterior tibiofibular ligament (ATFL). The ATFL is a weak, flat ligament that originates on the lateral malleolus of the fibula and extends anteromedially to the lateral side of the neck of the talus. It can be easily injured when the ankle is inverted (or ‘rolled’) with excessive force) |
| Dental pathology | Tooth loss (ante-mortem tooth loss of (2)) |

| p | x | p | p | p | p | p | p | p | p | p | p | p | p | p | p |
| --- | --- | --- | --- | --- | --- | --- | --- | --- | --- | --- | --- | --- | --- | --- | --- |
| 1 | 2 | 3 | 4 | 5 | 6 | 7 | 8 | 9 | 10 | 11 | 12 | 13 | 14 | 15 | 16 |
| 32 | 31 | 30 | 29 | 28 | 27 | 26 | 25 | 24 | 23 | 22 | 21 | 20 | 19 | 18 | 17 |
| p | p | p | p | p | p | p | p | p | p | p | p | p | p | p | p |

| **F.334, skeleton 1887**, Figures S30 | |
| --- | --- |
| Date of burial | 1320–49 |
| Interpretation of individual | Adult friar |
| Grave shape and dimensions (m) | Rectangular with rounded corners: 1.54+ by 0.60, 0.67 deep (originally *c*. 0.8 deep) |
| Burial position | South-southwest–north-northeast, extended, supine, the skull was in neutral position. The right arm was slightly abducted. Both upper limbs were flexed at the elbow to bring the right hand over the left forearm and the right hand around the right side of the abdomen. The lower limbs were extended |
| Associated items | Copper alloy girdle buckle, type 1 [1888], located over the L5 vertebra and pointing to the right. Oval or D-shaped frame, with pin and rectangular plate intact. The simple frame has a V-shaped seat or notch for the pin on the outside edge with the opposing bar slightly offset. The plate is formed from a folded sheet with a rectangular slot for the pin. There are a pair of short V-shaped notches on the front from the pin slot towards the back end of the plate. The strap is attached with two round rivets positioned towards the end of the plate. The pin has a small flange. 43x19mm, 10g, typologically mid-fourteenth–mid fifteenth century. Leather strap 15–20mm wide, 3mm thick |
| Age | Old middle adult: 36–45 years. Some degenerative characteristics suggest this may have been an old adult (60+ years) |
| Morphological sex | Male |
| Genetic sex | XY: male |
| MtDNA | H23 |
| Stature (cm) | 169.7 +/- 3.27 |
| Percentage of skeleton present | *c*. 80. Truncated by construction of friary building. |
| Preservation grade | 0: high |
| Fragmentation grade | 1: low |
| Other taphonomic alteration | Localised purple staining over right radius. No identifiable cause  Copper alloy staining over the anterior bodies of L5 and sacrum S1, from girdle buckle |
| Trauma | None observed |
| Pathology | Age related degeneration in the right shoulder girdle (macroporosity of the right acromioclavicular and sternoclavicular joints)  Osteoarthritis in the middle of the back and left knee (DISH on the right side from T6–T11 and bilateral eburnation over the lateral condyle (patellar lip) with associated eburnation on the patella) |
| Other skeletal observations | Age related ossification (ossification of laryngeal and first costochondral cartilage)  Possible overuse/activity markers related to DISH (bilateral enthesophytes over the anterior superior aspect of the iliac crest (ASIS). Enthesophyte formation over the lateral aspect of the left greater trochanter) |
| Geometric morphometrics | Markedly symmetrical humeri, possibly associated with being a ‘scholar’ during formative teenage years |
| Dental pathology | Mineralised dental plaque (slight–moderate labial/lingual/buccal dental calculus)  Systemic disorder (well defined linear enamel hypoplasia from (6)–(11) and significantly on (22)) |

| cl | cl | cl | cl | cl | cl hy | cl hy | cl hy | cl hy | cl hy | cl hy | p | p | p | p | cl |
| --- | --- | --- | --- | --- | --- | --- | --- | --- | --- | --- | --- | --- | --- | --- | --- |
| 1 | 2 | 3 | 4 | 5 | 6 | 7 | 8 | 9 | 10 | 11 | 12 | 13 | 14 | 15 | 16 |
| 32 | 31 | 30 | 29 | 28 | 27 | 26 | 25 | 24 | 23 | 22 | 21 | 20 | 19 | 18 | 17 |
| p | p | p | p | p | p | cl | cl | cl | p | cl hy | p cl | p | p | p | p |

| **F.336, skeleton 1894**, Figure S31 | |
| --- | --- |
| Date of burial | 1300–49 |
| Interpretation of individual | Adult friar |
| Grave shape and dimensions (m) | Rectangular with rounded corners: 2.10 by 0.55, 0.70 deep (originally *c*. 0.8 deep) |
| Burial position | South-southwest–north-northeast, extended, supine, the skull was in neutral position. The upper limbs were flexed at the elbow to bring the right hand over the upper left forearm and the left hand above the right hip. The lower limbs were straight, drawn at the ankles |
| Associated items | Copper alloy girdle buckle, type 1 [1898], located on the left side of the L4 vertebra and pointing to the right. Oval or D-shaped frame, the pin and short rectangular plate are intact with leather adhering to the plate. The frame is lipped on the outside edge with a V-shaped notch for the pin. The opposing bar is slightly offset. The plate is formed from a rectangular sheet of copper alloy with a rectangular notch for the pin. The upper surface of the plate may have traces of tinning or another coating. The pin has a flanged transverse ridge. There are two rivet holes towards the back of the plate to secure the leather strap within. 31x21mm, 8g, typologically fourteenth century. Leather strap 12–14 mm wide, 4mm thick. Textile present |
| Age | Mature adult: 45+ years. Degenerative characteristics suggest this may have been an old adult (60+ years). |
| Morphological sex | Male |
| Genetic sex | XY: male |
| MtDNA | U5a1a1 |
| Stature (cm) | 171.13 +/- 3.27 |
| Percentage of skeleton present | *c*. 99 (effectively complete) |
| Preservation grade | 1: high |
| Fragmentation grade | 1: low |
| Other taphonomic alteration | Copper alloy staining over the left side of T5 and T6 bodies, from girdle buckle  Copper alloy staining over the anterior border of the left radius and left lunate. Probably from girdle buckle  Copper alloy staining over a manubrium fragment. No identifiable cause, possibly a thin pendant that did not survive  Copper alloy staining over the right superior nuchal line. No identifiable cause, possibly a pin that did not survive |
| Trauma | Healed cranial trauma (two small healed depression fractures on the outer table of the frontal bone; largest is 29mm medial of left temporal line, 65mm superior of the supraorbital margin; sclerotic bone healing is seen within the depression; the smaller depressed fracture is 32mm medial of right temporal line, 43mm superior of right supraorbital margin. Fracture outlines are curvilinear/crescent shaped, directed from above) |
| Pathology | Possible trauma to the right eye or symptom of anaemic disorder (cribra orbitalia in right orbit. Because this is unilateral, the pathogenesis may relate to an inflammation of the lacrimal gland (Cole and Waldron 2019) which is situated in the upper lateral region of the eye orbits)  Age related degeneration in left elbow, left wrist, shoulder girdle and back (left elbow: marginal lipping around the proximal head of the left ulna. Left wrist: 4macro/microporosity over the pisiform facet of left triquetral. Shoulder girdle: bilateral macroporosity involving the acromioclavicular and sternoclavicular joints. Back: spondylophytes between C2–T1: bodies of C5–C6 are macroporotic; degenerative changes between the left inferior and superior C6–C7 facets, respectively. Minor spondylophytes between bodies of T2–T12. Large spondylophytes on anterior body of L3 and L4. Schmorl's nodes between T3–L2)  Osteoarthritis in the neck and right toe (neck: eburnation between right inferior and superior articular facet of C2–C3, respectively. Right toe: eburnation over the distal head of the right proximal foot phalanx)  Developmental disorder (symphalangism of a left manual distal interphalangeal joint)  Gout on the left and right big toes (gout on the medial and plantar sides of the left and right hallux heads) |
| Other skeletal observations | Remnant metopic suture  Supernumerary thoracic vertebra (thoracolumbar transitional vertebra (TLTV)  Lumbosacral Transitional Vertebra (sacralised fifth lumbar vertebra) (Lumbosacral Transitional Vertebrae (LSTV) with a right side diarthrodial joint articulating with the sacral ala. (see discussion)  Possible activity/overuse marker (enthesophyte formations on anterior table of right patella) |
| Dental pathology | Trauma (9) has an occlusal chip with worn edge)  Systemic disorder (enamel hypoplasia on maxillary incisors (6) and (11))  Tooth decay (interproximal caries between (30) (31) and (4) (5). Occlusal caries on (1))  Mineralised dental plaque (slight–moderate labial/lingual/buccal dental calculus)  Tooth loss: ante-mortem tooth loss of (16)) |

| ca | p | ca | ca | p | hy | / | p | t | / | hy | / | p | p | p | x |
| --- | --- | --- | --- | --- | --- | --- | --- | --- | --- | --- | --- | --- | --- | --- | --- |
| 1 | 2 | 3 | 4 | 5 | 6 | 7 | 8 | 9 | 10 | 11 | 12 | 13 | 14 | 15 | 16 |
| 32 | 31 | 30 | 29 | 28 | 27 | 26 | 25 | 24 | 23 | 22 | 21 | 20 | 19 | 18 | 17 |
| p | ca | ca | cl | p | cl | / | / | / | cl | cl | cl | cl | p | p | p |

| **F.343, skeleton 1918**, Figure S32 | |
| --- | --- |
| Date of burial | 1310–49 |
| Interpretation of individual | Young friar, based on age at death possibly studying for first degree. |
| Grave shape and dimensions (m) | Rectangular with rounded corners: 2.16 by 0.42, 0.55 deep (originally *c*. 0.6 deep) |
| Burial position | South-southwest–north-northeast, extended, supine, the skull was in neutral position. The upper limbs were flexed at the elbow to bring right hand over the right chest and left hand over the sternum. The lower limbs were straight, drawn at the ankles |
| Associated items | Iron girdle buckle, type unknown [1953], location uncertain due to later disturbance. Frame in poor and fragmentary condition, heavily corroded. D-shaped form, with possible pin attached. Typologically High/Late Medieval. 35 by 46mm, 21g, Typologically High/Late Medieval |
| Age | Adolescent: 13–18 years |
| Morphological sex | Male |
| Genetic sex | XY: male |
| MtDNA | No data |
| Stature (cm) | 178.03 +/- 3.27 |
| Percentage of skeleton present | *c*. 95 (effectively complete) |
| Preservation grade | 2: medium |
| Fragmentation grade | 1: low |
| Other taphonomic alteration | None observed |
| Trauma | None |
| Pathology | Probable infection of the scalp (porotic periosteal reaction over the calotte of the skull. Confined to the parietal bones, (superior to the superior temporal lines) and occipital planum)  Tuberculosis? (hyperascularization of the anterior surface of C6/C7 bodies) |
| Other skeletal observations | None |
| Dental pathology | Mineralised dental plaque: slight labial and lingual dental calculus  Heritable trait? (bilateral buccal pits on (18) and (31)  Systemic disorder (enamel hypoplasia on (3), (8), (11), (19)–(21) and (26)–(30)) |

| p | p | p | p | p | p | p | p | p | p | p | p | p | p | p | p |
| --- | --- | --- | --- | --- | --- | --- | --- | --- | --- | --- | --- | --- | --- | --- | --- |
| 1 | 2 | 3 | 4 | 5 | 6 | 7 | 8 | 9 | 10 | 11 | 12 | 13 | 14 | 15 | 16 |
| 32 | 31 | 30 | 29 | 28 | 27 | 26 | 25 | 24 | 23 | 22 | 21 | 20 | 19 | 18 | 17 |
| p | p | p | p | p | p | cl | cl | cl | p | p | p | p | p | p | p |

| **F.343 (disarticulated), skeleton 1916**, Figure S32 | |
| --- | --- |
| Date of burial | 1290–1329 |
| Interpretation of individual | Unknown |
| Grave shape and dimensions (m) | Unknown |
| Burial position | Entirely disarticulated in later grave F.343, with no *in situ* traces of earlier grave or skeleton. Redeposited bones mainly over thorax, with part of crania and two long bones apparently deliberately arranged in grave to west of skull of later burial |
| Associated items | None |
| Age | Young adult: 18–25 years |
| Morphological sex | Male |
| Genetic sex | Not sampled |
| MtDNA | Not sampled |
| Stature |  |
| Percentage of skeleton present | *c*. 12. See table S21 for element representation |
| Preservation grade | 1: high |
| Fragmentation grade | 1: low |
| Other taphonomic alteration | None observed |
| Trauma | None observed |
| Pathology | Slight porosity over the supraorbital ridge |
| Other skeletal observations | None |
| Dental pathology | Unequal wear: heavy wear over the buccal margin of (30) and (31) |

| -- | -- | -- | -- | -- | -- | -- | -- | -- | b | p | p | p | p | p |
| --- | --- | --- | --- | --- | --- | --- | --- | --- | --- | --- | --- | --- | --- | --- |
| 1 | 3 | 4 | 5 | 6 | 7 | 8 | 9 | 10 | 11 | 12 | 13 | 14 | 15 | 16 |
| 32 | 30 | 29 | 28 | 27 | 26 | 25 | 24 | 23 | 22 | 21 | 20 | 19 | 18 | 17 |
| p | p | p | -- | -- | -- | -- | -- | -- | -- | -- | -- | -- | -- | -- |

| **F.344, skeleton 1932**, Figure S33 | |
| --- | --- |
| Date of burial | 1320–49 |
| Interpretation of individual | Adult friar |
| Grave shape and dimensions (m) | Rectangular with rounded corners: 1.80 by 0.55, 0.78 deep (originally *c*. 0.9 deep) |
| Burial position | South-southwest–north-northeast, extended, supine, the skull was in neutral position. The upper limbs were slightly flexed to bring right hand over the top of the right leg, and the left hand over the pubis. The lower limbs were straight, drawn at the ankles |
| Associated items | Copper alloy girdle buckle, type 7 [1933], located over the L5 vertebra and pointing to the right. D-shaped buckle of unusually narrow form. The frame has a rectangular notch or recess for the pin. The pin has a slight transverse ridge. An unusual buckle possibly not used with a girdle, although its position within the grave contradicts this. A similar buckle made of iron is recorded from York (Ottaway and Rogers 2002, 2892). 30x20mm, 5g, typologically mid-fourteenth–fifteenth century. Leather strap width unknown, 3–4mm thick. Textile present |
| Age | Old Middle Adult: 36–45 years |
| Morphological sex | Male |
| Genetic sex | XY: male |
| MtDNA | No data |
| Stature (cm) | 171.84 +/- 3.27 |
| Percentage of skeleton present | *c*. 95. Truncated by construction of friary building. |
| Preservation grade | 1: high |
| Fragmentation grade | 1: low |
| Other taphonomic alteration | None observed |
| Trauma | Possible trauma in left hip (a supra-acetabular cyst on the left os coxae) |
| Pathology | Age related changes in the chest, neck and lower back (ossified costochondral cartilage between left 1st rib and manubrium. Macroporotic and spiculated C5–C7 endplates; minor spondylophytes over thoracic and lumbar bodies. Large spondylophytes on the right side of T1–T2 bodies and T11–T12 bodies, located near the kyphotic and lordotic transitions)  Osteoarthritis in the right elbow (eburnation of the right radiocapitellar joint)  Osteoma/harmatoma on the lateral side of the right radial proximal epiphysis (irregular oval shape, max dimension: 6.76mm) |
| Other skeletal observations | None |
| Geometric morphometrics | Markedly symmetrical humeri, possibly associated with being a ‘scholar’ during formative teenage years |
| Dental pathology | Chipped upper left molar (disto-occlusal trauma of (17) (metacone))  Activity related trauma to lower right premolar (V-shaped labial-lingual occlusal groove on (28) (habitual activity: thread notch?))  Tooth loss (ante-mortem tooth loss of (2) and (15))  Oral infection (periapical abscesses involving (3) and (19); the latter with periostitis localised around the alveolar margin)  Caries (occlusal caries on (3) and (19), interproximal caries on (13)) |

| p | x | ca pa | p | p | p | p | p | p | p | p | p | ca | p | x | p |
| --- | --- | --- | --- | --- | --- | --- | --- | --- | --- | --- | --- | --- | --- | --- | --- |
| 1 | 2 | 3 | 4 | 5 | 6 | 7 | 8 | 9 | 10 | 11 | 12 | 13 | 14 | 15 | 16 |
| 32 | 31 | 30 | 29 | 28 | 27 | 26 | 25 | 24 | 23 | 22 | 21 | 20 | 19 | 18 | 17 |
| p | p | p | p | p t | p | p | p | p | p | p | p | p | ca pa | p | t |

| **F.346, skeleton 1935**, Figure S34 | |
| --- | --- |
| Elements present | Relatively shallow burial, with extensive generalised later truncation |
| Date of burial | 1290–1349 |
| Interpretation of individual | Unknown |
| Grave shape and dimensions (m) | Rectangular with rounded corners: 1.4+ by 0.56, 0.45 deep (originally *c*. 0.6 deep) |
| Burial position | South-southwest–north-northeast, extended, supine. The lower limbs were drawn at the ankles |
| Associated items | None |
| Age | Juvenile: 5–12 years |
| Morphological sex | Indeterminate |
| Genetic sex | Not sampled |
| MtDNA | Not sampled |
| Stature (cm) | Not calculable |
| Percentage of skeleton present | *c*. 14. Truncated at knees, generalised truncation due to shallow burial. |
| Preservation grade | 1: high |
| Fragmentation grade | 1: low |
| Other taphonomic alteration | None observed |
| Trauma | None observed |
| Pathology | None observed |
| Other skeletal observations | None |
| Dental pathology | Skull absent |

| **F.347, skeleton 1945**, Figure S35 | |
| --- | --- |
| Date of burial | 1300–49 |
| Interpretation of individual | Novice or young local friar |
| Grave shape and dimensions (m) | Rectangular with rounded corners: 1.86 by 0.58, 0.52 deep (originally *c*. 0.6 deep) |
| Burial position | South-southwest–north-northeast, extended, supine, the skull was slightly laterally flexed and rotated to look over the left shoulder. The upper limbs were slightly abducted and flexed at the elbow to bring crossed hands over the lower thorax. the lower limbs were drawn at the knees and ankles |
| Associated items | Animal bone girdle buckle, type 1 [1944], located centrally over the S1 of the sacrum and pointing to the right. Similar but slightly different design than the elephant ivory example from F.314. Oval frame including a tongue rest, but lacks any lateral knops. The thin copper alloy pin was secured on a lateral shaft of iron, which no longer survives. This was inserted laterally through one side of the buckle, as with the ivory example. The integral plate has lightly curved edges and the upper surface is decorated with a lateral groove and two raised mouldings, with bands of triple incised lateral lines set just below the pin and along the inner edge of the plate. The plate is bifurcated and was secured to a strap with three small copper alloy rivets  Late Medieval bone buckles are known from France in particular (Chazottes and Thuaudet 2014). They occur in several forms, both with and without integral plates. Of the latter type, those with long rectangular plates are common (Chazottes and Thuaudet 2014, 189–90, fig. 3). Buckles with shorter integral plates, closer in form to the pair from Cambridge, have been found at Goltho, Douai and York, and there are also several unprovenanced examples (Chazottes and Thuaudet 2014 fig. 5). One of the Goltho buckles is unstratified, whilst the other, which forms a close parallel for the Cambridge bone buckle, was found on cobbles lying beside a building abandoned in the late fourteenth or early fifteenth century (Beresford 1975, 26, 77). The Douai bone buckle now lacks its frame, and its iron spindle passes through both edges of the plate, but its simple decoration is similar to the Cambridge bone buckle; it came from a context of the second half of the fourteenth century (Chaoui-Derieux 2010, 67, fig. 9). The York buckle has no precise provenance (Waterman 1959 fig 19.7). The distribution of these bone buckles, with finds from Goltho and York, suggests that they may well have been made in England, quite possibly in northern England. An example from Wharram Percy (Andrews and Milne 1979, 128, fig 70.31; MacGregor, A. 1985, 105; Riddler 2012, 197) is probably of an earlier tenth–twelfth century type, despite being found in a Late Medieval context |
| Age | Adolescent: 13–18 years |
| Morphological sex | Possibly male |
| Genetic sex | XY: male |
| MtDNA | U5a2c4 |
| Stature (cm) | Too young to calculate |
| Percentage of skeleton present | *c*. 99 (effectively complete) |
| Preservation grade | 2: medium |
| Fragmentation grade | 1: low |
| Other taphonomic alteration | None observed |
| Trauma | None observed |
| Pathology | Systemic condition (very slight bilateral cribra orbitalia. Localised, mild bilateral periosteal reactions over the zygomata, including the masseteric origin, the posterior aspect of the temporal squamae, the mandibular fossae, the mastoid processes, the occipital condyles, the occipital protuberance and anterior tables of the patellae) |
| Other skeletal observations | None |
| Dental pathology | Mineralised dental plaque (slight labial dental calculus)  Systemic stress indicator (enamel hypoplasia on (8) and (9)) |

| p | p | p | p | p | p | p | hy cl | hy | p | p | p | p | p | p | p |
| --- | --- | --- | --- | --- | --- | --- | --- | --- | --- | --- | --- | --- | --- | --- | --- |
| 1 | 2 | 3 | 4 | 5 | 6 | 7 | 8 | 9 | 10 | 11 | 12 | 13 | 14 | 15 | 16 |
| 32 | 31 | 30 | 29 | 28 | 27 | 26 | 25 | 24 | 23 | 22 | 21 | 20 | 19 | 18 | 17 |
| p | p | p | p | p | p | p | cl | cl | cl | p | p | p | p | p | p |

| **F.348, skeleton 1948**, Figure S36 | |
| --- | --- |
| Date of burial | 1349–1360/1420 |
| Interpretation of individual | Adult friar |
| Grave shape and dimensions (m) | Rectangular with rounded corners: 2.25 by 0.60, 0.54 deep (originally *c*. 0.7 deep) |
| Burial position | South-southwest–north-northeast, extended, supine, the skull was in neutral position. The upper limbs were abducted and flexed at the elbows to bring the hands over the thorax. The lower limbs were straight, drawn at the knees and ankles |
| Associated items | Iron girdle buckle, type 1 [1949], located centrally over the S2 of the sacrum and pointing to the right. Heavily corroded with plate and pin. Oval or D-shaped frame with rectangular plate. Leather strap attached by two rivets. 41 by 29mm, 16g. Typologically fourteenth–fifteenth century. Leather strap 28mm wide, 4mm thick. Textile present, probably relatively coarse animal fibre |
| Age | Young middle adult: 26–35 years |
| Morphological sex | Male |
| Genetic sex | XY: male |
| MtDNA | V3c |
| Stature (cm) | 177.32 +/- 3.27 |
| Percentage of skeleton present | *c*. 98 (effectively complete) |
| Preservation grade | 1: high |
| Fragmentation grade | 1: low |
| Other taphonomic alteration | None observed |
| Trauma | None observed |
| Pathology | Systemic stress indicators (slight porotic hyperostosis over superior aspect of the calotte. Mild periosteal reaction over anterior surface of right femur)  Age related degeneration in the back (Schmorl's nodes: T4, T5, T7–T9 and T11)  Developmental disorder (symphalangism (ankylosis) of the right pedal first distal interphalangeal joint (DIP) |
| Other skeletal observations | None |
| Dental pathology | Mineralised dental plaque (moderate to considerable lingual/labial/buccal dental calculus)  Tooth loss (ante-mortem tooth loss of (19) and (30). Complete alveolar resorption for (18) and partial for (30))  Tooth decay and infection (mesial caries on (13), with slight periapical infection)  Tooth trauma (dental trauma on (17) distolingually: edges are worn) |

| p | p | p | p | p | cl | cl | cl | p | cl | p | cl | cl ca pa | p | p | p |
| --- | --- | --- | --- | --- | --- | --- | --- | --- | --- | --- | --- | --- | --- | --- | --- |
| 1 | 2 | 3 | 4 | 5 | 6 | 7 | 8 | 9 | 10 | 11 | 12 | 13 | 14 | 15 | 16 |
| 32 | 31 | 30 | 29 | 28 | 27 | 26 | 25 | 24 | 23 | 22 | 21 | 20 | 19 | 18 | 17 |
| cl | cl | x | cl | cl | cl | cl | cl | cl | cl | cl | p cl | cl | x | cl | cl t |

| **F.352, disarticulated bone groups 1965 1966 1967 1968 1964**, Figure S37 | |
| --- | --- |
| Elements present | Entirely disarticulated groups of bone found in grave shaped cut with no *in situ* skeleton  [1965]: skull, rib, vertebra, right tibia  [1966]: left humerus, left scapula, rib, vertebrae, left femur  [1967]: rib, vertebrae, left tibia  [1968]: ribs, vertebrae, ulna, right os coxae, right femur  Absent elements: both clavicles, right scapula, right humerus, left radius and ulna, manubrium and sternum, 3 cervical, 6 thoracic and 3 lumbar vertebrae, right os coxae, sacrum, both patellae, both fibulae and both feet |
| Date of burial | 1349–1360/1420 |
| Interpretation of individual | Young adult friar, based on age at death possibly studying for advanced degree |
| Grave shape and dimensions (m) | Later cut containing bones rectangular with rounded corners: 1.2+ by 0.78, 0.59 deep (originally *c*. 0.7 deep) |
| Burial position | Disturbed, originally presumably aligned south-southwest–north-northeast |
| Associated items | Copper alloy girdle buckle, type 1 [1970], original position and orientation unknown. Oval or D-shaped frame. The pin and rectangular plate are intact. The frame is lipped on the outside edge with a V-shaped seat or notch for the pin. It appears slightly misshapen. The opposing bar is offset. The plate is formed from folded rectangular sheet copper alloy with rectangular notch for pin. The buckle plate is also recessed for the frame. The end of the plate has a centrally positioned aperture of circular form with angled grove pointing towards the pin on both the upper and lower surfaces. The back end of the plate is also slightly concave in shape with two protruding rivets fixing a leather strap fragment within the plate. No visible decoration to the upper surface of the plate. The pin has distinct flanges. The plate matches a type used on buckles with composited rigid plates, however, this buckle frame lacks the integral forked spacer that normally accompanies this type of buckle (Egan and Pritchard 2002, 79). 47x22mm, 12g, this appears to be an interesting hybrid typologically dating to the later fourteenth century. Leather strap 16mm wide, 3–4mm thick |
| Age | Young middle adult: 26–35 years |
| Morphological sex | Probably male |
| Genetic sex | XY: male |
| MtDNA | H2a5 |
| Stature (cm) | 167.08 +/- 3.27 |
| Percentage of skeleton present | *c*. 53. Disturbed and then truncated by construction of friary building |
| Preservation grade | 1: high |
| Fragmentation grade | 1: low |
| Other taphonomic alteration | Copper alloy staining over back of skull (left mid lambdoid). From girdle buckle after skeleton was disturbed and redeposited  Copper alloy staining over proximoanterior surface of right femur. From girdle buckle after skeleton was disturbed and redeposited |
| Trauma | None observed |
| Pathology | Systemic endocrine disorder (bilateral cribra orbitalia)  Probable scalp infection (periosteal reaction over the superior aspect of the calotte. Depression (4.82mm diameter) on the outer table of the right parietal located 37.17mm from bregma; involved by the periosteal reaction, which may suggest lysis rather than trauma. Osteoma/harmatoma (7.13mm diameter) located 32mm anterior of the parietal foramina on the sagittal suture)  Abnormal bone porosity over the hard palate (porotic and spiculated palatine process likely relates to an inflammatory response)  Age related change? (ossified costochondral cartilage)  Degeneration of the hip (plaque form defect around the right femoral head. Type B (after Hack et al. 2010 femoroacetabular cam impingement)  Inward pointing lower limbs (bilateral medial torsion (anteversion) of the femora) |
| Other skeletal observations | None |
| Dental pathology | Ante-mortem tooth loss (14)  Mineralised dental plaque (moderate to considerable asymmetric formation of lingual/labial/buccal dental calculus confined to the right side)  Asymmetric wear/oral parafunctionality (significant asymmetric occlusal wear affecting the right dental arcade: polished and faceted (suggests habitual activity and/or strong preference for right sided mastication)  Tooth decay and Infection (occlusal caries and large periapical buccal and lingual abscess affecting (3) |

| p | cl | cl ca pa | cl | cl | cl | p | p | p | p | p | p | p | x | p | p |
| --- | --- | --- | --- | --- | --- | --- | --- | --- | --- | --- | --- | --- | --- | --- | --- |
| 1 | 2 | 3 | 4 | 5 | 6 | 7 | 8 | 9 | 10 | 11 | 12 | 13 | 14 | 15 | 16 |
| 32 | 31 | 30 | 29 | 28 | 27 | 26 | 25 | 24 | 23 | 22 | 21 | 20 | 19 | 18 | 17 |
| cl | cl | cl | cl | cl | cl | cl | cl | p | p | p | p | p | p | p | p |

| **F.355, skeleton 1975**, Figure S38 | |
| --- | --- |
| Date of burial | 1349 |
| Interpretation of individual | Laity: benefactor |
| Grave shape and dimensions (m) | Rectangular with rounded corners: 1.1+ by 0.56, 0.83 deep (originally *c*. 0.9 deep) |
| Burial position | South-southwest–north-northeast, extended, supine, the skull was rotated to look over the left shoulder. The right arm was flexed at the elbow and wrist to bring the hand over the abdomen. The left arm was slightly flexed to bring the hand over the left hip |
| Associated items | None |
| Age | Young middle adult: 26–35 years |
| Morphological sex | Male |
| Genetic sex | XY: male |
| MtDNA | K2a6 |
| Pathogen aDNA | *Yersinia pestis* |
| Stature (cm) | 173.98 +/- 3.27 |
| Percentage of skeleton present | *c.* 63. Truncated at the pelvis by construction of friary building |
| Preservation grade | 1: high |
| Fragmentation grade | 1: low |
| Other taphonomic alteration | Black staining over right triquetral, hamate and capitate (wrist). No identifiable cause |
| Trauma | None observed |
| Pathology | Abnormal bone porosity over forehead and cheek bones (porotic hyperostosis over the supraorbital ridge, extending bilaterally over the zygomatic processes)  Probable infection of the scalp (possible (healed) periosteal reaction characterised by mild cribra and sclerosis over the calotte and bilateral mediolateral sulci over parietals, *c.* 25mm anterior of the parietal foramina)  Age related degeneration in the back (Schmorl's nodes involving five thoracic vertebrae) |
| Other skeletal observations | None |
| Dental pathology | Mineralised dental plaque (slight–moderate labial/lingual/buccal dental calculus) |

| ne | p | cl | p | p | p | p | p | p | p | cl | cl | cl | p | p | ne |
| --- | --- | --- | --- | --- | --- | --- | --- | --- | --- | --- | --- | --- | --- | --- | --- |
| 1 | 2 | 3 | 4 | 5 | 6 | 7 | 8 | 9 | 10 | 11 | 12 | 13 | 14 | 15 | 16 |
| 32 | 31 | 30 | 29 | 28 | 27 | 26 | 25 | 24 | 23 | 22 | 21 | 20 | 19 | 18 | 17 |
| p | p | p | p | p | p | cl | cl | cl | cl | p | p | p | p | p | ne |

| **F.367, skeleton 2011**, Figure S39 | |
| --- | --- |
| Date of burial | 1300–49 |
| Interpretation of individual | Adult friar |
| Grave shape and dimensions (m) | Rectangular with rounded corners: 2.00 by 0.50, 0.50 deep (originally *c*. 0.7 deep) |
| Burial position | South-southwest–north-northeast, extended, supine, the skull was in possible neutral rotation (facial bones truncated). The upper limbs were flexed at the elbow to bring the hands over the respective ischial spines and pubis. The lower limbs were straight, drawn at the ankles |
| Associated items | Copper alloy girdle buckle, type 6 [2010], located between the sacrum and the pelvic pubic arch and pointing to the right. Rectangular frame and rectangular sheet copper alloy plate intact. The frame has a thick outside edge with two filed grooves towards the top and bottom and three grooves towards the middle, the central one acting as a seat or guide for the pin. The sides of the frame are very thin and slightly convex in form. The pin is intact and of simple form. The plate is decorated to the upper surface with three parallel grooves towards the back edge and there is the suggestion of parallel diagonal lines to the reverse. The plate is attached to a leather strap with two rivets towards the rear of the plate. Similar to examples for both York (Ottaway and Rogers 2002, 2839), and London (Egan and Pritchard 2002, 96). Little use wear. Frame 20x21mm, plate 25x18mm, 14g, typologically fourteenth century. Leather strap 17–19mm wide, 3–5mm thick, bovine |
| Age | Mature adult: 45+ |
| Morphological sex | Probably male |
| Genetic sex | XY: male |
| MtDNA | U5b |
| Stature (cm) | 173.98 +/- 3.27 |
| Percentage of skeleton present | *c*. 95 (effectively complete) |
| Preservation grade | 1: high |
| Fragmentation grade | 1: low |
| Other taphonomic alteration | Copper alloy staining around right second metacarpophalangeal joint, from girdle buckle |
| Trauma | None observed |
| Pathology | Developmental disorder of the skull (craniosynostosis characterised by obliteration and flattening of the obelionic suture and bossing of the occipital planum)  Age related degeneration in the shoulder girdles, neck, lower back (minor bilateral lipping of the glenohumeral joints; macroporosity inferior to the posterior margin of the left humeral head and between the right humeral head and the greater tubercle. Macroporosity of the right sternoclavicular and left acromioclavicular joints. Macroporosity and spiculation of C5 inferior endplate, C6 endplates and C7 superior endplate, T11 inferior and T12 superior; moderate on all endplates from L1–L5. Minor spondylophytes on right anterior bodies of L2/L3. Schmorl's nodes from T7–T12 and L1–L3.  Bridging osteophyte of the right anterosuperior sacroiliac joint)  Trauma? (anterosuperior periosteal reaction towards the sternal end of the right clavicle. This may relate to mechanical irritation of the area, such as a strap)  Gout in the left foot (osteoma/harmatoma on the medial border of the left MT3 5.94mm diameter, 28.38mm from distal end. Mild sclerotic reaction over the plantar margin of the left MT4. A proximal osteophyte on a left pedal intermediate phalanx) |
| Other skeletal observations | Activity expression in the hands and knees (hands: noted bilateral expression of the flexor digitorum sublimis insertion onto the manual intermediate phalanges. Knees: bilateral superiorly pointed enthesophyte formations on the anterior table of the patellae) |
| Dental pathology | None observed |

| -- | -- | -- | -- | -- | -- | -- | -- | -- | -- | -- | -- | -- | -- | -- | -- |
| --- | --- | --- | --- | --- | --- | --- | --- | --- | --- | --- | --- | --- | --- | --- | --- |
| 1 | 2 | 3 | 4 | 5 | 6 | 7 | 8 | 9 | 10 | 11 | 12 | 13 | 14 | 15 | 16 |
| 32 | 31 | 30 | 29 | 28 | 27 | 26 | 25 | 24 | 23 | 22 | 21 | 20 | 19 | 18 | 17 |
| -- | -- | -- | -- | -- | -- | / | / | / | / | / | / | / | p | p | p |

## The chapter house

| **F.146, skeleton 1164**, Figures S41–S42 | |
| --- | --- |
| Date of burial | 1500–38 |
| Interpretation of individual | Laity: adult benefactor |
| Grave shape and dimensions (m) | Rectangular with rounded corners: 2.02 by 0.45, 1.30 deep (originally *c*. 1.4 deep) |
| Burial position | South-southwest–north-northeast, extended, supine, the skull was in neutral position. The upper limbs were straight against the body and pronated to bring the hand next to the hips. The lower limbs were straight, drawn at the ankles |
| Associated items | Copper alloy Nuremberg jetton [1524]; probably deliberately placed next to leg and related to possible infection. Anonymous Rose/Orb type (large orb) (*c*. 1500−1580s) (cf. Mitchiner 1988, 377−81, nos. 1190−1226), 24mm diameter, chipped, 1.15g |
| Age | Mature adult: 46+ years. Degenerative characteristics suggest this may have been an old adult (60+ years) |
| Morphological sex | Female |
| Genetic sex | XX: female |
| MtDNA | H1ak |
| Stature (cm) | 157.84 +/- 3.72 |
| Percentage of skeleton present | *c*. 98 (effectively complete) |
| Preservation grade | 0: high |
| Fragmentation grade | 1: low |
| Other taphonomic alteration | Copper alloy staining over the distomedial aspect of the right tibia |
| Trauma | None observed |
| Pathology | Age related degeneration in the shoulders and lower back (shoulders: bilateral marginal lipping around the glenoid cavities. Lower back: intervertebral endplate macroporosity between C6 and C7. Minor Schmorl's nodes from T4 to L3 with apparent associated micro-fracturing of the bodies inferiorly)  Possible bilateral bacterial infection of the lower legs (inflammation of the left and right tibia and fibula, characterised by notable bone formation affecting the diaphyseal and distal metaphyseal surfaces. The condition generally worsens distally. The affected elements take a swollen appearance characterised by vascularised sclerotic bone) |
| Other skeletal observations | Activity related expression in the shoulders, forearms and right hand (shoulders: rugosity and enthesophyte formations around the deltoid tuberosities of the humeri. Forearms: rugosity/remodelling of the interosseous borders of the distal ulnae/radii. Right hand: Expression/hypertrophy of the palmar and dorsal interossei muscle attachments on the manual proximal phalanges) |
| Dental pathology | Mineralised dental plaque (slight-moderate labial/lingual/buccal of dental calculus; (sage green colouration)  Tooth decay (interproximal caries on (13), buccal carries in (18) and (17))  Tooth loss (ante-mortem tooth loss of (15), (16) and (19)) |

| p | p | p | p | p | p | p | p | p | p | p | p | ca | p | x | x |
| --- | --- | --- | --- | --- | --- | --- | --- | --- | --- | --- | --- | --- | --- | --- | --- |
| 1 | 2 | 3 | 4 | 5 | 6 | 7 | 8 | 9 | 10 | 11 | 12 | 13 | 14 | 15 | 16 |
| 32 | 31 | 30 | 29 | 28 | 27 | 26 | 25 | 24 | 23 | 22 | 21 | 20 | 19 | 18 | 17 |
| p | p | p | p | p | p | l | l | l | l | p | p | p | x | ca | ca |

| **F.189, no skeleton**, Figure S43 | |
| --- | --- |
| Date of burial | 1360/1420–1425/75 |
| Interpretation of individual | Unknown, but probably revered member of the Austin friars |
| Grave shape and dimensions (m) | Later ‘translation’ cut rectangular with rounded corners: 2.02 by 0.70, 0.93 deep (originally *c*. 1.0 deep) |
| Discussion | Grave-shaped cut with no skeletal remains, probably related to later ‘translation’ of bones |
| Burial position | Presumably south-southwest–north-northeast, extended, supine |
| Associated items | None |
| Percentage of skeleton present | 0 |

| **F.190, skeleton 1458**, Figure S44 | |
| --- | --- |
| Date of burial | 1425/75–1538 |
| Interpretation of individual | Laity: child of benefactor |
| Grave shape and dimensions (m) | Rectangular with rounded corners: 1.66 by 0.95, 1.12 deep (originally *c*. 1.2 deep) |
| Burial position | South-southwest–north-northeast, extended, supine, the skull was flexed forward and rotated to look over the right shoulder. The upper limbs were straight against the body and pronated to bring the hand next to the hips. The lower limbs were straight, drawn at the ankles |
| Associated items | None |
| Age | Juvenile: 5–12 years |
| Morphological sex | Too young to determine |
| Genetic sex | Consistent with XY but not XX: male |
| MtDNA | V7a |
| Pathogen aDNA | *Yersinia pestis* |
| Stature (cm) | Too young to calculate |
| Percentage of skeleton present | *c*. 99 (effectively complete) |
| Preservation grade | 1: high |
| Fragmentation grade | 1: low |
| Other taphonomic alteration | None observed |
| Trauma | None observed |
| Pathology | None observed |
| Other skeletal observations | None |
| Dental pathology | Mineralised dental plaque (slight labial and lingual calculus on (24) and (25))  Systemic stress indicator (enamel hypoplasia on (24), (22) and (21)) |

| ne | p | / | p | p | p | p | p | p | p | p | p | p | p | p | ne |
| --- | --- | --- | --- | --- | --- | --- | --- | --- | --- | --- | --- | --- | --- | --- | --- |
| 1 | 2 | 3 | 4 | 5 | 6 | 7 | 8 | 9 | 10 | 11 | 12 | 13 | 14 | 15 | 16 |
| 32 | 31 | 30 | 29 | 28 | 27 | 26 | 25 | 24 | 23 | 22 | 21 | 20 | 19 | 18 | 17 |
| ne | p | p | p | / | p | / | cl | cl hy | p | hy | hy | p | p | p | ne |

| **F.191, skeleton 1460**, Figure S45 | |
| --- | --- |
| Date of burial | 1425/75–1538 |
| Interpretation of individual | Adult friar |
| Grave shape and dimensions (m) | Rectangular with rounded corners: 1.90 by 0.72, 1.08 deep (originally *c*. 1.2 deep) |
| Burial position | South-southwest–north-northeast, extended, supine, the skull was in a neutral position. The right arm was slightly abducted. Both upper limbs were flexed at the elbow to bring the left hand around the right side of the abdomen and the right hand over the left os coxae. The lower limbs were straight, drawn at the ankles |
| Associated items | Copper alloy girdle buckle, type 2 [1465] located on the pelvis on the upper wing of left ilium and pointing right. Double oval frame buckle, with the frame thickening to middle on either side. The central bar is slightly narrower and projects slightly above and below the frame. There are filing marks present along the outside edge of the frame. There appears not to be a seat or notch for the pin. The pin is missing; however, the trapezoidal sheet plate is present, there are two rivets towards the back end with leather. Typologically late fourteenth–fifteenth century. Leather strap 30mm wide, 4mm thick |
| Age | Mature adult: 46+ years |
| Morphological sex | Male |
| Genetic sex | XY: male |
| MtDNA | T1a1 |
| Stature (cm) | 178.74 +/- 3.27 |
| Percentage of skeleton present | *c*. 99 (effectively complete) |
| Preservation grade | 1: high |
| Fragmentation grade | 1: low |
| Other taphonomic alteration | None observed |
| Trauma | Diaphyseal fracture of the left forearm (healing fracture (transverse/minimally oblique/comminuted) of the left ulna. The ipsilateral radius was unaffected) |
| Pathology | Probable infection of the scalp (calotte exhibits diffuse sclerotic and nodular bone reactions confined to the posterosuperior aspect of both parietal bones)  Age related changes in the throat and chest, back and right hip (chest: extensive ossification of laryngeal and costochondral cartilage. Back: degeneration just superior to the cervicothoracic and thoracolumbar transitions. Moderate spondylophytes around the intervertebral margins of C4 & C5. Small intervertebral Schmorl’s nodes between T8 & T9; anterior displacement of the T11 superior nucleus pulposus. Vertical and posterior nucleus pulposus displacement between T11–T12, of which were in the process of ankylosing. Right hip: degenerative asymmetry of the sacroiliac joint; the right has a greater degree of modification) |
| Other skeletal observations | Activity related expression? (bilateral expression of the pectoralis major rugosities on the clavicles. This suggests a habitual resistance to extension of the upper limbs at the shoulder (i.e., flexion that may involve activities such as lifting) |
| Dental pathology | Infection (periapical abscess of (6), with indications of diffuse periodontal disease involving the upper right dental arch)  Tooth loss (extensive ante-mortem tooth loss of (9)–(20), (27) and (29)–(32), with alveolar resorption) |

| p | p | p | p | p | pa | p | p | x | x | x | x | x | x | x | x |
| --- | --- | --- | --- | --- | --- | --- | --- | --- | --- | --- | --- | --- | --- | --- | --- |
| 1 | 2 | 3 | 4 | 5 | 6 | 7 | 8 | 9 | 10 | 11 | 12 | 13 | 14 | 15 | 16 |
| 32 | 31 | 30 | 29 | 28 | 27 | 26 | 25 | 24 | 23 | 22 | 21 | 20 | 19 | 18 | 17 |
| x | x | x | x | / | x | / | / | / | / | / | / | x | x | x | x |

| **F.230, skeleton 1482**, Figures S46–S47 | |
| --- | --- |
| Date of burial | 1475–1538 |
| Interpretation of individual | Novice friar |
| Grave shape and dimensions (m) | Rectangular with rounded corners: 1.95 by 0.64, 1.12 deep (originally *c*. 1.2 deep) |
| Burial position | South-southwest–north-northeast, extended, supine. The skull was rotated to look over the left shoulder. The right arm was adducted at the shoulder and flexed at the elbow to bring the hand pronated over the left hand next to the left os coxae. The left arm was straight. The lower limbs were straight, drawn at the ankles |
| Associated items | Copper alloy girdle buckle, type 2 [1507], located over the upper wing of the left ilium of the pelvis and pointing right. Small double oval frame with decorative grooves on both sides of the frame and intact pin. 28x24mm, 7g, typologically fourteenth–early fifteenth century. Leather strap 16mm wide, 3mm thick |
| Age | Juvenile: 5–12 years |
| Morphological sex | Too young to determine |
| Genetic sex | XY: male |
| MtDNA | T2b |
| Pathogen aDNA | *Yersinia pestis* |
| Stature (cm) | Too young to calculate |
| Percentage of skeleton present | *c*. 97 (effectively complete) |
| Preservation grade | 2: medium |
| Fragmentation grade | 1: low |
| Other taphonomic alteration | None observed |
| Trauma | None observed |
| Pathology | Endocrine disorder (sub-circular osteolytic lesions on the frontal and parietal bones, located around the parietal foramina. Large, sharply defined, smooth walled, scalloped edge osteolytic lesions present on the left humerus, left clavicle left radius and ulna. Only the diaphysis remains of the latter two elements. The lesions in the left arm and left clavicle immediately penetrate the medullary cavities with no evidence of an osteogenic response (such as a periosteal reaction). Osteolytic lesions are present on the proximal, anteromedial half of the left femur, around the greater trochanter of the right femur the lateral surfaces of the tibiae and the medial surface of the right fibula. The auricular surface of the left os coxae is characterised by subchondral erosion and spiculated new bone) |
| Other skeletal observations | None |
| Dental pathology | Mineralised dental plaque (slight lingual and labial dental calculus)  Tooth decay (caries along the gingival margin of (M)) |

| p | p | p | p | p | p | p | p | p | p |
| --- | --- | --- | --- | --- | --- | --- | --- | --- | --- |
| A | B | C | D | E | F | G | H | I | J |
| T | S | R | Q | P | O | N | M | L | K |
| p | p | p | p | cl | cl | cl | p | p | ca cl |

| **F.260, skeleton 1863**, Figure S48 | |
| --- | --- |
| Date of burial | 1360/1420–1425/75 |
| Interpretation of individual | Young friar, based on age at death possibly studying for first degree |
| Grave shape and dimensions (m) | Rectangular with rounded corners: 1.90 by 0.72, 0.84 deep (originally *c*. 1.1 deep) |
| Burial position | South-southwest–north-northeast, extended, supine. The skull was in forward and lateral flexion towards the left shoulder, resting against the wall of the grave cut. The upper limbs were straight against the body to bring the hands under the respective sides of the ischium. The lower limbs were straight |
| Associated items | Copper alloy girdle buckle, type unknown [1864], located over the L5 vertebrae and pointing to the right. Disturbed, length of heavily corroded copper alloy pin, 1g. Leather strap present |
| Age | Young adult: 18–25 years |
| Morphological sex | Probably male |
| Genetic sex | Consistent with XY but not XX: male |
| MtDNA | U5a1a1 |
| Stature (cm) | 171.17 +/- 4.05 |
| Percentage of skeleton present | *c*. 88 |
| Preservation grade | 2: medium |
| Fragmentation grade | 1: low |
| Other taphonomic alteration | Anteroposterior compression of the skull causing deformation and green bone fracturing.  Black mottling/staining over the lower appendicular elements, particularly around the knees |
| Trauma | None observed |
| Pathology | Possible scalp infection (periosteal reaction over superior aspect of calotte with marked venous sulci over the outer table of the right parietal bone)  Lower back instability (spondylophytes around the anterior margins of L4 and L5. Bilateral spondylolysis of L5, grade 1 anterolisthesis (Niggemann et al. 2012) and left posterolateral wedging of the body)  Spina bifida occulta (complete sacral midline cleft) |
| Other skeletal observations | Biomechanical compensation for lower back instability (L2–L4 have a lipped, sub-semi-circular cortical extension from the endplate rims. This is a unilateral expression occurring on the right side of the bodies, located inferiorly on L2, superiorly and inferiorly on L3 and superiorly on L4)  Possible marker for a habitual squatting posture (anterior medial squatting facet on the left tibia) |
| Dental pathology | None observed |

| e | p | p | p | p | p | / | p | p | p | p | p | p | p | p | p |
| --- | --- | --- | --- | --- | --- | --- | --- | --- | --- | --- | --- | --- | --- | --- | --- |
| 1 | 2 | 3 | 4 | 5 | 6 | 7 | 8 | 9 | 10 | 11 | 12 | 13 | 14 | 15 | 16 |
| 32 | 31 | 30 | 29 | 28 | 27 | 26 | 25 | 24 | 23 | 22 | 21 | 20 | 19 | 18 | 17 |
| p | p | p | p | p | / | p | p | p | p | p | p | p | p | p | e |

| **F.310, skeleton 1771**, Figure S49 | |
| --- | --- |
| Date of burial | 1425/75–1538 |
| Interpretation of individual | Young friar, based on age at death possibly studying for first degree |
| Grave shape and dimensions (m) | Trapezoidal: 1.90 by 0.66–0.60, 1.10 deep (originally *c*. 1.2 deep) |
| Burial position | South-southwest–north-northeast, extended, supine, the skull was in neutral position. The upper limbs were flexed at the elbows to bring the left hand around the side of the right abdomen and the right hand over the right pubis. The lower limbs were straight |
| Associated items | Copper alloy girdle buckle, type 2 [1787], located to the right of the L3 vertebra and pointing to the right. Small double oval frame with slightly asymmetrical loops. The upper portion of one of the loops shows signs of wear. Traces what appears to be an iron pin remain. Similar buckles from London have been suggested to be shoe buckles (Egan and Pritchard 2002, 86), however, the position within the grave indicates it was a girdle buckle. 25x19mm, 3g, typologically fourteenth–early fifteenth century. Leather strap 9mm wide, 2mm thick |
| Age | Young adult: 18–25 years |
| Morphological sex | Possibly female? |
| Genetic sex | Consistent with XY but not XX: male |
| MtDNA | W3a1a2 |
| Pathogen aDNA | *Yersinia pestis* |
| Stature (cm) | 171.17 +/- 4.05 |
| Percentage of skeleton present | *c*. 98 (effectively complete) |
| Preservation grade | 2: medium |
| Fragmentation grade | 1: low |
| Other taphonomic alteration | None observed |
| Trauma | None observed |
| Pathology | Systemic stress indicator (slight bilateral cribra orbitalia)  Possible sinusitis (periosteal reactions over posterior portions of the vomer)  Degeneration of the back (marginal lipping between T8-T9. Schmorl's nodes between T8–L1. Marginal lipping around left femoral caput)  Possible developmental disorders (bilateral medially bowing tibial curvature. Symphalangism of a distal interphalangeal joint (possible little toe) |
| Other skeletal observations | None |
| Dental pathology | Mineralised dental plaque (slight lingual dental calculus)  Systemic stress indicator (enamel hypoplasia on (5), (8) and (9)) |

| p | p | / | p | hy | p | p | hy | hy | p | / | p | p | p | p | p |
| --- | --- | --- | --- | --- | --- | --- | --- | --- | --- | --- | --- | --- | --- | --- | --- |
| 1 | 2 | 3 | 4 | 5 | 6 | 7 | 8 | 9 | 10 | 11 | 12 | 13 | 14 | 15 | 16 |
| 32 | 31 | 30 | 29 | 28 | 27 | 26 | 25 | 24 | 23 | 22 | 21 | 20 | 19 | 18 | 17 |
| / | p | cl | p | p | cl | l | l | l | l | cl | p | p | cl | / | p |

# Burial catalogue for the church and cloister

By Sarah A. Inskip

There is a considerable collection of human skeletal remains recovered in the nineteenth–twentieth centuries that may relate to the Cambridge Austin friars that are held in the Duckworth Collection. A lack of precise information means that in some cases it is impossible to determine if human skeletal remains relate to the friary or the nearby parish cemetery of St Bene’t’s. Only material that can be certainly linked to the friary has been included in this work. Although a wide range of complete and partial skeletons and disarticulated remains were recovered and studied (Duckworth and Pocock 1910), only cranial elements could be located in the Duckworth Collection. Although it is possible to link certain skeletons to the friary church (Table S24) and cloister (Table S25), there does not appear to be any means to link them to particular skeletons whose locations were recorded in 1908–9. The human skeletal remains were recorded using the methodologies of the After the Plague project (Robb in prep.), sex and age information has been adjusted to make these comparable to the skeletons recorded by Neil from the cemetery and chapter house (see above). Given the limitations of this material only basic information is presented. Additional material will be presented in After the Plague publications, including palaeopathological information recorded by Jenna Dittmar (Robb in prep.). The remains are predominantly adult, although one juvenile is present, and male or probably male, although four female or probably female individuals are represented.

**Table S1.** Basic information for radiocarbon determinations on skeletons from the Cambridge Austin friars. All determinations are on human rib bone. HPD: highest posterior density.

| SUERC lab. no. | Burial | δ13C | δ15N | C14 age (BP) | 68% HPD | 95% HPD | Comment |
| --- | --- | --- | --- | --- | --- | --- | --- |
| 78903 | 343 | -18.2 | 14.5 | 732±26 | 1264–1284 | 1226–1295 | Cemetery sequence, earliest/first burial |
| 78900 | 332 | -18.5 | 13.4 | 688±22 | 1277–1298 | 1271–1385 | Cemetery sequence, middle/second burial |
| 78901 | 328 | -18.5 | 14.0 | 635±26 | 1295–1388 | 1286–1397 | Cemetery sequence, latest/third burial |
| 78902 | 230 | -19.3 | 11.0 | 404±26 | 1445–1485 | 1437–1619 | Chapter house burial |

**Table S2.** 95.4 per cent HPD ranges for unadjusted, adjusted and modelled results for radiocarbon determinations from the Cambridge Austin friars.

| Burial or phasing | Unadjusted | Marine reservoir adjusted | Marine reservoir and bone turnover adjusted | Bayesian modelling |
| --- | --- | --- | --- | --- |
| 343 | 1226–1294 | 1299–1407 | 1303–1411 | 1318–1416 |
| 332 | 1272–1384 | 1309–1421 | 1315–1428 | 1348–1425 |
| 328 | 1286–1396 | 1325–1447 | 1329–1450 | 1393–1445 |
| End of cemetery | – | – | – | 1395–1503 |
| Start of chapter house | – | – | – | 1431–1533 |
| 230 | 1437–1618 | 1488–1643 | 1490–1645 | 1476–1536 |

**Table S3.** Sex estimation categories.

| Term | Read as | Meaning |
| --- | --- | --- |
| Female | Female | Analyst has full confidence in the determination of sex for the remains |
| Male | Male |  |
| (female) | Probably Female | Analyst does not have full confidence in the determination, but feels the remains are probably the stated sex |
| (male) | Probably Male |  |
| Female? | Possibly female | Analyst does not have confidence in the determination, but feels the available evidence hints at the stated sex |
| Male? | Possibly male |  |
| Indet. | Sex indeterminate | The remains have been analysed, but lacks sufficient diagnostic morphology for determination |

**Table S4.** Age estimation categories.

|  | Sub-adult |  |  |  | Adult |  |  |  |  |
| --- | --- | --- | --- | --- | --- | --- | --- | --- | --- |
| Category | Neonate | Infant (I) | Juvenile (J) | Adolescent (ADOL) | Adult (A) | Young Adult (YA) | Young Middle Adult (YMA) | Old Middle Adult (OMA) | Mature Adult (MA) |
| Abbreviation |  | I | J | ADOL | A | YA | YMA | OMA | MA |
| Age (years) | <6months | 0–4 | 5–12 | 13–18 | 18+ | 18–25 | 26–35 | 36–45 | 46+ |

**Table S5 S23.** Dental recording notations.

| p | l | x | b | / | r | e | ne |
| --- | --- | --- | --- | --- | --- | --- | --- |
| Present in bone | Loose | Lost ante-mortem | Broken post-mortem | Lost post-mortem | Root only | Erupting | Not erupted |
| cl | ca | sc | pa | pd | hy | t | -- |
| Calculus | Caries | Sclerosed | Periapical abscess | Periodontal disease | Hypoplasia | Trauma | Bone not present |

**Table S6.** Skeletal fragmentation categories.

| Attribute | Score | Definition |
| --- | --- | --- |
| Low | 1 | At least one of an axial, upper and lower appendicular elements is complete |
| Medium | 2 | Excluding sullegic and trephic processes, all axial and appendicular elements are broken due to some taphonomic agency but at least one bone from each major anatomical zone can be refitted |
| High | 3 | None of the elements can be completely refitted. Fragment sizes mostly range between 10–200mm with diagenetic processes impacting on bone survival |
| Very high | 4 | Fragment sizes mostly range between 10–50mm. significant diagenetic activity is a likely factor for poor preservation |

**Table S7.** Skeletal preservation categories.

| Attribute | Score | Definition |
| --- | --- | --- |
| High | 0 | Surface morphology clearly visible with fresh appearance to bone and no modifications |
|  | 1 | Slight and patchy surface erosion |
| Medium | 2 | More extensive surface erosion than grade 1 with deeper surface penetration |
|  | 3 | Most of bone surface affected by some degree of erosion; general morphology maintained but detail of parts of surface masked by erosive action. |
| Low | 4 | All of bone surface affected by erosive action; general profile maintained and depth of modification not uniform across whole surface. |
|  | 5 | Heavy erosion across whole surface, completely masking normal surface morphology, with some modification |
| Very low | 5+ | As grade 5, but with extensive penetrating erosion resulting in modification of profile |

**Table S8.** Head position of skeletons in Cambridge Austin friars cemetery and chapter house.

| Head position | Cemetery | Chapter house | Total |
| --- | --- | --- | --- |
| Neutral | 11 | 3 | 14 |
| Rotated left | 4 | 2 | 6 |
| Rotated right | 3 | 1 | 4 |
| Unknown | 14 | – | 14 |
| **Total** | **32** | **6** | **38** |

**Table S9.** Arm position of skeletons in Cambridge Austin friars cemetery and chapter house.

| Arm position | Cemetery | Chapter house | Total |
| --- | --- | --- | --- |
| Straight | 3 | 4 | 7 |
| Over thorax | 10 | – | 10 |
| Over abdomen/hips | 10 | 2 | 10 |
| Over thighs | 1 | – | 1 |
| Unknown | 8 | – | 8 |
| **Total** | **32** | **6** | **38** |

**Table S10.** Leg position of skeletons in Cambridge Austin friars cemetery and chapter house.

| Leg position | Cemetery | Chapter house | Total |
| --- | --- | --- | --- |
| Drawn together | 22 | 5 | 27 |
| Straight | 4 | 1 | 5 |
| Unknown | 6 | – | 6 |
| **Total** | **32** | **6** | **38** |

**Table S11.** Staining on skeletons from the Cambridge Austin friars cemetery and chapter house.

| Burial | Location | Colour | Agent | Cause |
| --- | --- | --- | --- | --- |
| 146 | Medial side of right tibia | Green | Copper alloy | Jetton |
| 216 | T12 vertebrae | Green | Copper alloy | Unknown |
| 260 | Lower limbs, particularly knees | Black, mottled | Manganese | Unknown |
| 302 | Sacrum | Green | Copper alloy | Girdle buckle |
| 332 | L4 vertebra | Green | Copper alloy | Girdle buckle |
| 334 | L5/sacrum, right radius | Green, purple | Copper alloy, Permanganate | Copper alloy from girdle buckle |
| 336 | T5 vertebra, manubrium, head (r), wrist (r) | Green | Copper alloy | Girdle buckle, plus unknown items? |
| 352 | Back of head, top of right thigh | Green | Copper alloy | Girdle buckle, including after redeposition |
| 355 | Around right wrist | Black | Iron, Manganese | Unknown |
| 367 | Around right 2nd MCP joint | Green | Copper alloy | Girdle buckle |

**Table S12.** Truncation of articulated individuals from the Cambridge Austin friars cemetery. No truncation was apparent at the chapter house.

| Plane of truncation | Burial(s) | Location of truncation | Missing |
| --- | --- | --- | --- |
| Axial | 140, 312 | Thoracic vertebrae | Proximally (at T3) |
|  | 195, 199, 215, 217 | Thighs | Proximally (various) |
|  | 237, 328, 331, 355 | Hips | Proximally (1) and distally (3) |
|  | 333, 334, 346 | Knees | Proximally (1) and distally (2) |
| Right parasagittal | 309 | Skull, vertebrae, pelvis, Lower limbs | Laterally (right) |
| Right parasagittal | 315 | Forearm, pelvis, leg | Laterally (right) |
| Coronal | 328, 367 | Skull | Anteriorly |
| Unknown | 332 | Skull | Mixed |

**Table S13.** Male stature in the Cambridge Austin friars cemetery by age group in cm.

| Age | No. | Average | Geomean (central tendency) | Median | Range |
| --- | --- | --- | --- | --- | --- |
| Adolescent | 2 | 174.135 | 174.09 | 174.135 | 170.24–178.03 |
| Young Adult | 3 | 169.32 | 169.14 | 168.51 | 160.00–179.46 |
| Middle Adult | 13 | 173.34 | 173.28 | 173.98 | 167.08–180.89 |
| Mature Adult | 5 | 174.08 | 174.02 | 173.98 | 167.56–180.89 |
| **All** | **23** | **172.72** | **172.62** | **173.98** | **160.00–180.89** |

**Table S14.** Stature in the Cambridge Austin friars chapter house by age and sex in cm.

| Sex | Young Adult | Mature Adult |
| --- | --- | --- |
| Male | 171.17 | 178.74 |
| Female | 175.38 | 157.84 |

**Table S15.** Summary of trauma in the Cambridge Austin friars cemetery and chapter house.

| Burial | Skeletal element | Type |
| --- | --- | --- |
| 191 | Ulna | Low energy trauma |
| 232 | Coccyx | Blunt force trauma |
| 311 | Ribs | Blunt force trauma |
| 312 | Tibia | Cut mark (perimortem?) |
| 332 | Neck and thighs | High energy trauma |
| 336 | Head | Blunt force trauma |
| 344 | Hip | Hyperflexion/hyperextension |

**Table S16.** Number of individuals from the Cambridge Austin friars cemetery and chapter house affected by degeneration, by anatomical region.

| Throat | Chest | Shoulder | Elbow | Wrist | Back | Hip | Knee | Ankle | Foot |
| --- | --- | --- | --- | --- | --- | --- | --- | --- | --- |
| 1 | 1 | 6 | 3 | 1 | 12 | 2 | 1 | 1 | 1 |

**Table S17**. Inflammation of the skull of individuals from the Cambridge Austin friars cemetery and chapter house.

|  | 191 | 260 | 309 | 328 | 343 | 352 | 355 |
| --- | --- | --- | --- | --- | --- | --- | --- |
| Severity | High | High | High | Low | Low | High | Low |
| Age | MA | YA | YMA | ADOL | ADOL | YMA | YMA |

**Table S18.** Dental calculus of individuals at the Cambridge Austin friars cemetery and chapter house.

| Feature | Age | Accumulation | Location |
| --- | --- | --- | --- |
| 146 | MA | Slight | Labial, lingual buccal |
| 190 | J | Slight | Labial, lingual |
| 230 | J | Slight | Labial, lingual |
| 232 | OMA | Slight | Lingual, buccal |
| 265 | OMA | Slight | Lingual, buccal |
| 302 | OMA | Considerable | Labial, lingual buccal |
| 309 | YMA | Slight | Labial |
| 310 | YA | Slight | Lingual |
| 311 | MA | Moderate | Labial, lingual buccal |
| 314 | YA | Slight | Lingual |
| 315 | YA | Slight | Labial |
| 331 | OMA | Slight | Labial, lingual buccal |
| 334 | OMA | Moderate | Labial, lingual buccal |
| 336 | MA | Moderate | Labial, lingual buccal |
| 343 | ADOL | Slight | Labial, lingual |
| 347 | ADOL | Slight | Labial |
| 348 | YMA | Considerable | Labial, lingual buccal |
| 352 | YMA | Considerable | Labial, lingual buccal |
| 355 | YMA | Slight | Labial, lingual buccal |

**Table S19.** Disarticulated cranial elements from the Cambridge Austin friars cemetery.

| Side (element, age) | No. of features | No. of contexts | MNE no. | Fragment no. |
| --- | --- | --- | --- | --- |
| PAIRED BONES | 3 | 3 | 3 | 45 |
| Nasal (young adult) | 1 | 1 | 1 | 1 |
| Parietal (adult) | 1 | 1 | 1 | 39 |
| Temporal (sub-adult) | 1 | 1 | 1 | 5 |
| LEFT SIDED ELEMENTS | 11 | 11 | 17 | 32 |
| Frontal (total) | 2 | 2 | 3 | 11 |
| Frontal (adult) | 1 | 1 | 2 | 10 |
| Frontal (young adult) | 1 | 1 | 1 | 1 |
| Mandible (adult) | 1 | 1 | 3 | 3 |
| Maxilla (total) | 2 | 2 | 2 | 2 |
| Maxilla (adult) | 1 | 1 | 1 | 1 |
| Maxilla (young adult) | 1 | 1 | 1 | 1 |
| Parietal (total) | 3 | 3 | 3 | 5 |
| Parietal (adult) | 2 | 2 | 2 | 2 |
| Parietal (young adult) | 1 | 1 | 1 | 3 |
| Temporal (adult) | 1 | 1 | 3 | 8 |
| Zygomatic (adult) | 1 | 1 | 2 | 2 |
| Zygomaticomaxilla (young adult | 1 | 1 | 1 | 1 |
| RIGHT SIDED ELEMENTS | 8 | 8 | 11 | 28 |
| Frontal (total) | 2 | 2 | 3 | 12 |
| Frontal (adult) | 1 | 1 | 2 | 11 |
| Frontal (young adult) | 1 | 1 | 1 | 1 |
| Mandible (total) | 2 | 2 | 2 | 7 |
| Mandible (adult) | 1 | 1 | 1 | 4 |
| Mandible (young adult) | 1 | 1 | 1 | 3 |
| Maxilla (adult) | 1 | 1 | 1 | 1 |
| Parietal (young adult) | 1 | 1 | 1 | 1 |
| Temporal (total) | 2 | 2 | 4 | 7 |
| Temporal (adult) | 1 | 1 | 3 | 6 |
| Temporal (young adult) | 1 | 1 | 1 | 1 |
| Single axial elements | 3 | 3 | 4 | 35 |
| Occipital (total) | 2 | 2 | 3 | 17 |
| Occipital (adult) | 1 | 1 | 2 | 16 |
| Occipital (young adult) | 1 | 1 | 1 | 1 |
| Sphenoid (adult) | 1 | 1 | 1 | 18 |
| UN-SIDED ELEMENTS | 6 | 6 | 6 | 11 |
| Calvaruim (adult) | 1 | 1 | 1 | 4 |
| Frontal (sub-adult) | 1 | 1 | 1 | 1 |
| Occipital (total) | 2 | 2 | 2 | 3 |
| Occipital (adult) | 1 | 1 | 1 | 1 |
| Occipital (sub-adult) | 1 | 1 | 1 | 2 |
| Sphenoid (total) | 2 | 2 | 2 | 3 |
| Sphenoid (sub-adult) | 1 | 1 | 1 | 1 |
| Sphenoid (young adult) | 1 | 1 | 1 | 2 |
| **Overall total** | **31** | **31** | **41** | **151** |

**Table S20.** Disarticulated post-cranial skeletal elements from the Cambridge Austin friars cemetery.

| Side (element, age) | F.no. | Context no. | MNE no. | Fragments no. |
| --- | --- | --- | --- | --- |
| PAIRED BONES | 1 | 1 | 1 | 2 |
| Os Coxae (adult) | 1 | 1 | 1 | 2 |
| LEFT SIDED ELEMENTS | 18 | 18 | 18 | 40 |
| 1st M/C (adult) | 1 | 1 | 1 | 1 |
| 2nd M/T (adult) | 1 | 1 | 1 | 1 |
| 3rd M/C (adult) | 1 | 1 | 1 | 1 |
| 3rd M/T (adult) | 1 | 1 | 1 | 1 |
| 5th M/C (adult) | 1 | 1 | 1 | 1 |
| Calcaneus (adult) | 1 | 1 | 1 | 1 |
| Femur (total) | 2 | 2 | 2 | 3 |
| Femur (adult) | 1 | 1 | 1 | 1 |
| Femur (sub-adult) | 1 | 1 | 1 | 2 |
| Humerus (adult) | 2 | 2 | 2 | 17 |
| Os Coxae (adult) | 1 | 1 | 1 | 3 |
| PPH (adult) | 1 | 1 | 1 | 1 |
| Radius (adult) | 1 | 1 | 1 | 1 |
| Rib (adult) | 1 | 1 | 1 | 2 |
| Ribs (adult) | 1 | 1 | 1 | 4 |
| Talus (sub-adult) | 1 | 1 | 1 | 1 |
| Tibia (total) | 2 | 2 | 2 | 2 |
| Tibia (adult) | 1 | 1 | 1 | 1 |
| Tibia (sub-adult) | 1 | 1 | 1 | 1 |
| Axial elements | 5 | 5 | 5 | 6 |
| Lumbar vert. (total) | 4 | 4 | 4 | 5 |
| Lumbar vert. (adult) | 3 | 3 | 3 | 3 |
| Lumbar vert. (sub-adult) | 1 | 1 | 1 | 2 |
| Thoracic vert. (adult) | 1 | 1 | 1 | 1 |
| RIGHT SIDED ELEMENTS | 28 | 28 | 28 | 32 |
| 1st PPH (adult) | 1 | 1 | 1 | 1 |
| 2nd M/T (sub-adult) | 1 | 1 | 1 | 1 |
| 3rd M/C (adult) | 1 | 1 | 1 | 1 |
| 3rd M/T (sub-adult) | 1 | 1 | 1 | 1 |
| 4th M/C (adult) | 1 | 1 | 1 | 1 |
| Clavicle (total) | 2 | 2 | 2 | 2 |
| Clavicle (sub-adult) | 1 | 1 | 1 | 1 |
| Clavicle (young adult) | 1 | 1 | 1 | 1 |
| Femur (total) | 3 | 3 | 3 | 3 |
| Femur (adult) | 2 | 2 | 2 | 2 |
| Femur (sub-adult) | 1 | 1 | 1 | 1 |
| Humerus (total) | 3 | 3 | 3 | 4 |
| Humerus (adult) | 2 | 2 | 2 | 2 |
| Humerus (young adult) | 1 | 1 | 1 | 2 |
| Ilium (adult) | 2 | 2 | 2 | 3 |
| Radius (total) | 3 | 3 | 3 | 3 |
| Radius (adult) | 2 | 2 | 2 | 2 |
| Radius (young adult) | 1 | 1 | 1 | 1 |
| Rib (adult) | 1 | 1 | 1 | 1 |
| Ribs (adult) | 1 | 1 | 1 | 3 |
| Sacrum – ala (adult) | 1 | 1 | 1 | 1 |
| Talus (sub-adult) | 1 | 1 | 1 | 1 |
| Tibia (total) | 2 | 2 | 2 | 2 |
| Tibia (adult) | 1 | 1 | 1 | 1 |
| Tibia (sub-adult) | 1 | 1 | 1 | 1 |
| Ulna (total) | 4 | 4 | 4 | 4 |
| Ulna (adult) | 3 | 3 | 3 | 3 |
| Ulna (young adult) | 1 | 1 | 1 | 1 |
| UN-SIDED ELEMENTS | 6 | 6 | 6 | 12 |
| Femoral caput (adult) | 1 | 1 | 1 | 1 |
| Radius (adult) | 1 | 1 | 1 | 1 |
| Rib (adult) | 1 | 1 | 1 | 1 |
| Ribs (adult) | 1 | 1 | 1 | 5 |
| Scapula (adult) | 1 | 1 | 1 | 1 |
| Thoracic vert. (adult) | 1 | 1 | 1 | 3 |
| **Overall total** | **58** | **57** | **58** | **92** |

**Table S21.** Zonation of disarticulated elements from the Cambridge Austin friars cemetery.

| Feature | Context | Element | Zone |
| --- | --- | --- | --- |
| 103 | 1010 | Femur | 10 |
| 103 | 1010 | Radius | 1,2,5 |
| 103 | 1010 | Ilium | 10 |
| 103 | 1029 | Lumbar Vert | 1 |
| 103 | 1029 | Lumbar Vert | 1 |
| 103 | 1029 | Calcaneus | Complete |
| 110 | 1036 | 4th M/C | 1,3 |
| 110 | 1036 | Parietal | 4 (posterior frag) |
| 112 | 1040 | Frontal | N/A (fragment) |
| 112 | 1040 | Occipital | 5 |
| 112 | 1040 | Sphenoid | N/A |
| 112 | 1040 | Temporal | 6,7 |
| 112 | 1040 | Lumbar Vert | 1,2,3 |
| 112 | 1040 | Femur | 5,3,2,6 |
| 112 | 1040 | Femur | 9,10,11 |
| 112 | 1040 | Tibia | 7 |
| 112 | 1040 | Tibia | 7 |
| 112 | 1040 | Talus | Complete |
| 112 | 1040 | Talus | Complete |
| 112 | 1040 | 2nd M/T | Complete |
| 112 | 1040 | 3rd M/T | Complete |
| 113 | 1042 | 2nd M/T | 3 partial 1,2 |
| 113 | 1042 | 3rd M/T | 3 partial 1,2 |
| 183 | 1392 | Os Coxae | 7,10,12,4,6,1,2,3 |
| 183 | 1392 | 1st M/C | Complete |
| 183 | 1392 | 3rd M/C | Complete |
| 183 | 1392 | PPH | Complete |
| 183 | 1392 | Thoracic Vert | 1,2,3 |
| 206 | 1394 | Humerus | 2,11,10,9,8,7 |
| 206 | 1394 | Rib | 1,2 |
| 206 | 1394 | Rib | 1 |
| 206 | 1394 | Rib | 3 |
| 206 | 1394 | Scapula | 7 |
| 206 | 1394 | Thoracic Vert | 1 |
| 309 |  | 3rd M/C | Complete |
| 318 | 1846 | Sacrum - Ala | 2 |
| 343 | 1916 | Frontal | complete |
| 343 | 1916 | Frontal | complete |
| 343 | 1916 | Occipital | 5 (planum) |
| 343 | 1916 | Sphenoid | n/a |
| 343 | 1916 | Mandible | 3,4,5,6 |
| 343 | 1916 | Parietal | complete refit |
| 343 | 1916 | Parietal | complete |
| 343 | 1916 | Temporal | 7 |
| 343 | 1916 | Maxilla | 12 |
| 343 | 1916 | Zygomaticomaxilla | 11,13 |
| 343 | 1916 | Nasal | 14,15 |
| 343 | 1916 | Humerus | complete refit |
| 343 | 1916 | Radius | 5,6,7,8 |
| 343 | 1916 | Ulna | A,B,C,D,E,F,G |
| 343 | 1916 | Clavicle | 1,2,3 |
| 397 | 1004 | Frontal | 2 |
| 397 | 1004 | Frontal | 1 |
| 397 | 1004 | Occipital | 5 |
| 397 | 1004 | Sphenoid | N/A |
| 397 | 1004 | Mandible | 1,6 |
| 397 | 1004 | Mandible | 1,4,5,6 |
| 397 | 1004 | Parietal | 3,4 |
| 397 | 1004 | Temporal | 6 |
| 397 | 1004 | Temporal | 7 |
| 397 | 1004 | Maxilla | 12 |
| 397 | 1004 | Maxilla | 13 |
| 397 | 1004 | Zygomatic | 10 |
| 397 | 1004 | Humerus | 2,10,7,8,3,5,6,4 |
| 397 | 1004 | Femur | 1,2,3,4,5 |
| Layer | 1006 | Clavicle | 2,3, ½ 1 |
| Layer | 1006 | Tibia | 1,3,7 |
| Layer | 1026 | 1st PPH | Complete |
| Layer | 1215 | Humerus | Complete |
| Layer | 1215 | Ulna | A,B,C,D, ½ E |
| Layer | 1215 | Ilium | 12 |
| Layer | 1215 | Femur | 6 |
| Layer | 1215 | Femoral Caput | 1 |
| Layer | 1215 | 5th M/C | Complete |
| Layer | 1534 | Tibia | 7,8,9,10 |
| Layer | 1391 | Humerus | 3,4,5,6, ½ 7, ½ 8 |
| Layer | 1391 | Radius | 1,2,5 |
| Layer | 1391 | Radius | 3,4,9,10,8,6,7,5 |
| Layer | 1391 | Ulna | A,B,C,D,1/2 E |
| Layer | 1391 | Ulna | E,F,G,H |
| Layer | 1391 | Ribs | 1,2 |
| Layer | 1391 | Ribs | 1,2 |
| Layer | 1391 | Ribs | 3 |
| Layer | 1391 | Lumbar vert. | Complete |
| Layer | 1802 | Radius | ½ 1, 5,6,7,8, ½ 9, 1/2, 10 |
| Layer | 1802 | Os Coxae | 2,4,12 |
| Layer | 2046 | Parietal | 4 |
| Layer | 2046 | Occipital | 5 |
| Layer | 2046 | Calvaruim | Indet. |

**Table S22.** Number of individuals from disarticulated/comingled remains from the Cambridge Austin friars cemetery, based on additional individuals within single features and contexts by age and sex.

| Sex/age | Sub-adult | A | YA | YMA | Total |
| --- | --- | --- | --- | --- | --- |
| Male | – | 3 | – | 1 | 4 |
| Probable male | 1 | 2 | 1 | – | 4 |
| Female | – | – | – | – | – |
| Possible female | – | 1 | – | – | 1 |
| Indeterminate | 1 | 15 | – | – | 16 |
| **Total** | **2** | **21** | **1** | **1** | **25** |

**Table S23.** Summary of selected conditions and information from disarticulated/comingled remains from the Cambridge Austin friars cemetery.

| Feature/context | Condition | Category | Notes |
| --- | --- | --- | --- |
| 103 | Schmorl’s nodes | Degenerative | Lumbar vertebra |
| 106 | Dental calculus | Dental pathology | (20) to (29) |
|  | Tooth loss | Dental pathology | (19) |
|  | Gout? Septic bursitis? | Bacterial infection | Left elbow |
|  | Cam impingement | Degenerative | left hip |
| 343 | Unusual buccal wear | Dental pathology | (30) to (32) |
|  |  | Stature estimation | 166.54±4.05cm |
| 397 | Cribra orbitalia | Endocrine/traumatic | Left orbit |
|  | Slight porotic hyperostosis | Metabolic | Parietal bones |
|  | Periodontal disease | Dental pathology | Left maxilla |
| [1215] |  | Stature estimation | 177.02±4.05cm |

**Table S24.** Cranium from Cambridge Austin friars church in the Duckworth Collection

| Catalogue | Location | Sex | Age |
| --- | --- | --- | --- |
| 0779A | Mortlake house, Benet Street | Male | Adult: 18+ |

**Table S25.** Crania from the Cambridge Austin friars cloister garth/walk in the Duckworth Collection. The numbering of some of the crania relates to the publication by Duckworth and Pocock (Duckworth and Pocock 1910).

| Catalogue | Location/information on bag | Sex | Age |
| --- | --- | --- | --- |
| 5331 | Examination Hall | Male | Adult: 18+ |
| 5401 | Examination Hall foundation | Male | Adult: 18+ |
| 5402 | Examination Hall foundation | Male | Adult: 18+ |
| 5403 | Examination Hall foundation | Probably male | Adult: 18+ |
| 5404 | Examination Hall foundation | Male | Adult: 18+ |
| 5405 | Examination Hall foundation | Male | Adult: 18+ |
| 5406 | Examination Hall foundation | (male) | Adult: 18+ |
| 5407 | Examination Hall foundation | Male | Adult: 18+ |
| 5408 | Examination Hall foundation | Male | Adult: 18+ |
| 5409 | Examination Hall foundation | Male | Adult: 18+ |
| 5616 | New Lecture Room | Male | Adult: 18+ |
| 5618 | New Lecture Room | Probably male | Adult: 18+ |
| 5629 | Examination Hall | Male | Adult: 18+ |
| ? | New Lecture rooms | Indet | Adult: 18+ |
| ? | New Lecture rooms | Indet. | Juvenile: 5–12 |
| EU.1.00.281 | Examination Hall | Male | Adult: 18+ |
| EU.1.00.282 | Examination Hall | Male | Adult: 18+ |
| EU.1.00.287 | New Lecture Room | Male | Adult: 18+ |
| EU1.1.1 | BS I, A, 20 VII? | Probably male | Adult: 18+ |
| EU1.1.10 | BS10 | Male | Mature adult: 46+, degenerative changes suggest could be 60+ |
| EU1.1.11 | NO11 Examination Hall, 1908 | Male | Adult: 18+ |
| EU1.1.12 | BS12 | Male | Adult: 18+ |
| EU1.1.13 | BS13 | Probably male | Adult: 18+ |
| EU1.1.14 | BS14 | Male | Adult: 18+ |
| EU1.1.15 | 15 | Male | Mature adult: 46+, degenerative changes suggest could be 60+ |
| EU1.1.16 | 16 | Male | Adult: 18+ |
| EU1.1.17 | BS17 | Male | Adult: 18+ |
| EU1.1.18 | 18 | Male | Mature adult: 46+, degenerative changes suggest could be 60+ |
| EU1.1.19 | BS19 Female | Probably female | Adult: 18+ |
| EU1.1.2 | BS 2; B, 21VII | Male | Adult: 18+ |
| EU1.1.20 | BS20 Female | Probably female | Adult: 18+ |
| EU1.1.21 | 21 Female | Probably female | Adult: 18+ |
| EU1.1.22 | 22 Female | Female | Mature adult: 46+, degenerative changes suggest could be 60+ |
| EU1.1.24 | 24 | Probably male | Adult: 18+ |
| EU1.1.26 | BS26 | Probably male | Adult: 18+ |
| EU1.1.27 | BS27 | Male | Adult: 18+ |
| EU1.1.29 | BS29 | Probably male | Adult: 18+ |
| EU1.1.3? | BS 3 | Male | Mature adult: 46+, degenerative changes suggest could be 60+ |
| EU1.1.30 | BS30 | Male | Adult: 18+ |
| EU1.1.32 | BS32 | Male | Adult: 18+ |
| EU1.1.33A | BS18 | Probably male | Adult: 18+ |
| EU1.1.4 | NO4 Examination Hall: 1908, PC 1.00.283 | Male | Adult: 18+ |
| EU1.1.5 | BS 5 | Probably male | Mature adult: 46+, degenerative changes suggest could be 60+ |
| EU1.1.6 | BS 6 | Male | Adult: 18+ |
| EU1.1.7 | NO7 Examination Hall, 1908 | Male | Adult: 18+ |
| EU1.1.8 | (6) New no. 8 | Male | Adult: 18+ |
| EU1.1.9 | BS9 | Male | Adult: 18+ |

**Figure S1.** Bar chart of head positions of skeletons in the Cambridge Austin friars cemetery and chapter house.

**Figure S2.** Bar chart of arm positions of skeletons in the Cambridge Austin friars cemetery and chapter house.

**Figure S3.** Bar chart of leg positions of skeletons in the Cambridge Austin friars cemetery and chapter house.

**Figure S4.** Bar chart of osteological sex attributions of skeletons in the Cambridge Austin friars cemetery and chapter house. This does not include aDNA information (see Figure 12).

**Figure S5.** Bar chart of osteological age group attributions of skeletons in the Cambridge Austin friars cemetery and chapter house (see Figure 12).

**Figure S6.** Pie chart of ratio of degeneration according to regions affected, of skeletons in the Cambridge Austin friars cemetery and chapter house.

**Figure S7.** Plan of Cambridge Austin friars cemetery, with photographs of individual skeletons overlaid (see Figure 5).

**Figure S8.** Photograph of burial F.106, skeleton [1866].

**Figure S9.** Photograph of tryncated burail F.140, skeleton [1139].

**Figure S10.** Photograph of truncated burial F.195, skeleton [1355].

**Figure S11.** Photograph of truncated burials F.196, skeleton [1353] (right), and F.198, skeleton [1364] (left). Image also shows pier foundation F.183 from later cloister, which truncated these skeletons.

**Figure S12.** Photograph of truncated burial F.199, skeleton [1367].

**Figure S13.** Photograph of truncated burial F.215, skeleton [1425].

**Figure S14.** Photograph of truncated burial F.216, skeleton [1428], plus detail photograph of *in situ* copper alloy girdle buckle type 1 [1430], photographs of buckle and x-ray of buckle.

**Figure S15.** Photograph of truncated burial F.217, skeleton [1432].

**Figure S16.** Photograph of burial F.232, skeleton [1482].

**Figure S17.** Photograph of truncated burial F.237, skeleton [1516].

**Figure S18.** Photograph of burial F.265, skeleton [1602], plus detail photograph of *in situ* copper alloy girdle buckle type 1 [1638] and photographs and x-ray of buckle and leather strap.

**Figure S19.** Photographs of charnel in grave F.302 and burial F.302, skeleton [1735], with some charnel remaining, plus detail photograph of *in situ* copper alloy girdle buckle type 5 [1737] and photograph of buckle.

**Figure S20.** Photograph of truncated burial F.309, skeleton [1767], plus photographs of cranium with high level of inflammation possibly related to tonsuring.

**Figure S21.** Photograph of burial F.311, skeleton [1782], plus detail photograph of *in situ* iron girdle buckle type 4 [1788] and photographs of buckle.

**Figure S22.** Photograph of burial F.312, skeleton [1785], plus inset photograph of cranium [1972] which had slumped and become detached as the fill of an earlier pit compacted.

**Figure S23.** Photograph of burial F.314, skeleton [1797], plus detail photograph of *in situ* elephant ivory girdle buckle type 1 [1803] and photographs of buckle.

**Figure S24.** Photograph of truncated burial F.315, skeleton [1800].

**Figure S25.** Photograph of truncated burial F.328, skeleton [1824], plus photograph of cranium with low level of inflammation possibly related to tonsuring. Surviving skeleton disturbed due to shallow burial.

**Figure S26.** Photograph of truncated burial F.331, skeleton [1873], plus detail photograph of *in situ* copper alloy girdle buckle type 3 [1882] and photographs and x-ray of buckle.

**Figure S27.** Photograph of truncated burial F.332, skeleton [1879] with broken and displaced femurs, plus detail photograph of *in situ* copper alloy girdle buckle type 3 [1882] and photographs of buckle.

**Figure S28.** Plan of burial F.332, skeleton [1879], with X-rays (with colours inverted) and detailed photographs of perimortem bilateral femoral fractures and photographs of C6 vertebrae with incomplete perimortem fracture and left clavicle with healed fracture.

**Figure S29.** Photograph of truncated burial F.333, skeleton [1884], plus detail photograph of *in situ* iron girdle buckle, type 4 [1899] and photographs of buckle. Image also shows wall foundation F.340 from later cloister, which truncated this skeleton.

**Figure S30.** Photograph of truncated burial F.334, skeleton [1887], plus detail photograph of *in situ* copper alloy girdle buckle, type 1 [1888] and photographs of buckle. Image also shows wall foundation F.340 from later cloister, which truncated this skeleton.

**Figure S31.** Photograph of burial F.336, skeleton [1894], plus detail photograph of *in situ* copper alloy girdle buckle type 1 [1898] and photographs. X-ray of cranium with evidence for blunt force trauma and photograph of metatarsal. medial side of the left hallux head with evidence for gout.

**Figure S32.** Photographs of charnel in grave F.343 and burial F.343, skeleton [1918], plus photograph of cranium with low level of inflammation possibly related to tonsuring.

**Figure S33.** Photograph of slightly truncated burial F.344, skeleton [1932], plus detail photograph of *in situ* copper alloy girdle buckle type 7 [1933] and photographs of buckle.

**Figure S34.** Photograph of truncated burial F.346, skeleton [1935]. Surviving skeleton disturbed due to shallow burial.

**Figure S35.** Photograph of burial F.347, skeleton [1945], plus detail photograph of *in situ* animal bone girdle buckle type 1 [1944] and photographs of buckle.

**Figure S36.** Photograph of burial F.348, skeleton [1948], plus detail photograph of *in situ* iron girdle buckle type 1 [1949] and photographs of buckle.

**Figure S37.** Photograph of disturbed burial F.352, disarticulated bone groups [1965], [1966], [1967 and [1968], plus detail photograph of *in situ* copper alloy girdle buckle type 1 [1970] and photographs and x-ray of buckle and leather strap. Plus, photograph of cranium with high level of inflammation possibly related to tonsuring.

**Figure S38.** Photograph of truncated burial F.355, skeleton [1975], plus photograph of cranium with low level of inflammation possibly related to tonsuring.

**Figure S39.** Photograph of burial F.367, skeleton [2011], plus detail photograph of *in situ* copper alloy girdle buckle, type 6 [2010] and photographs of buckle and leather strap.

**Figure S40.** Plan of Cambridge Austin friars chapter house with photographs of individual skeletons (see Figure 8).

**Figure S41.** Photograph of burial F.146, skeleton [1164], plus photographs of copper alloy jetton [1524] found beside skeleton and silver coin [1163] from general grave fill. Light coloured floor makeup visible in upper section of grave cut. Note that the photograph of the skeleton is a combination of two images, as the skeleon has to be excavated in two stages.

**Figure S42.** Plan of burial F.146, skeleton [1164], with photographs of detail of right and left humerii, detail of right ulna, detail of left radius, whole right tibia and fibula, whole left tibia and fibula and detail of left fibula. These show notable bone formation, probably linked to an infection.

**Figure S43.** Photograph of empty grave shaped feature F.189, probably related to ‘translation’ of skeleton from earlier burial. Light coloured floor makeup visible, plus foundation for chapter house bench (upper left) and burial F.190, skeleton [1458] (lower right).

**Figure S44.** Photograph of burial F.190, skeleton [1458]. Light coloured floor makeup visible, plus part of empty grave shaped feature F.189 (upper left).

**Figure S45.** Photograph of burial F.191, skeleton [1460], plus detail photograph of *in situ* copper alloy girdle buckle type 2 [1465], photographs and x-ray of buckle. Photographs of cranium with high level of inflammation possibly related to tonsuring plus x-ray and detail photograph of healing fracture of the left ulna. Light coloured floor makeup visible.

**Figure S46.** Photograph of burial F.230, skeleton [1482], plus detail photograph of *in situ* copper alloy girdle buckle type 2 [1507] and photographs and x-ray of buckle.

**Figure S47.** Plan of burial F.230, skeleton [1482], with photographs of right clavicle (A), right humerus (B), right radius and ulna (C–D), detail of left femur (E), left tibia and fibula (F–G) and detail of right tibia (H). These have extraordinarily large lesions probably linked to osteitis fibrosa cystica, although other possible causes exist.

**Figure S48.** Photograph of burial F.260, skeleton [1863].

**Figure S49.** Photograph of burial F.310, skeleton [1771], plus detail photograph of *in situ* copper alloy girdle buckle type 2 [1787] plus photographs of buckle and leather strap.

# References

Agale, S.V. 2013. ‘Chronic Leg Ulcers: Epidemiology, Aetiopathogenesis, and Management’. *Ulcers* 2013: 1–9. https://doi.org/10.1155/2013/413604.

Ahlmann, E., Ma, Y., and Tunru-dinh, V. 2011. ‘A Rare Case Report of Extensive Polyostotic Gorham’s Disappearing Bone Disease Involving the Upper Extremity’. *Case Reports in Orthopedics* 2011: 1–6. https://doi: 10.1155/2011/486756.

Aimutis, W. 2004. ‘Bioactive Properties of Milk Proteins with Particular Focus on Anticariogenesis’. *Journal of Nutrition* 134 (4): 989s–995. <https://doi.org/10.1128/JB.188.5.1691>.

Ajello, L. 1974. “Natural History of the Dermatophytes and Related Fungi.” *Mycopathologia et Mycologia applicata* 53: 93–110.

Akcali, A, and N Lang. 2017. ‘Dental Calculus: The Calciﬁed Biofilm and Its Role in Disease Development’. *Periodontolog*y 2000: 1–8. https://doi.org/10.1111/prd.12151.

Aktan, Z. and Savaş, R. 1998. ‘Anatomic and HRCT Demonstration of Midline Sternal Foramina’. *Turkish Journal of Medical Sciences* 28: 511–514.

AlQahtani, S, M Hector, and H Liversidge. 2010. ‘Brief Communication: The London Atlas of Human Tooth Development and Eruption’. *American Journal of Physical Anthropology* 142 (3): 481–90. https://doi.org/10.1002/ajpa.21258.

Al-Shaqsi, S, E Zellner, J Ching, C Forrest, and J Phillips. 2018. ‘The Natural History of Cranial Morphology in Sagittal Craniosynostosis’. *Journal of Craniofacial Surgery* 29 (4): 852–855. https://doi.org/10.1097/SCS.0000000000004298.

Alt, Kurt W., C. Loring Brace, and Jens C. Türp. 1998. The History of Dental Anthropology, Edited by K Alt, F Rosing, and M Teschler-Nicola. *Dental Anthropology. Fundamentals, Limits, and Prospects*. 15–39. Wein: Springer. https://doi.org/10.1007/978-3-7091-7496-8_3.

Alvi, H, D Kalainov, D Biswas, A Soneru, and M Cohen. 2014. ‘Surgical Management of Symptomatic Olecranon Traction Spurs.’ *Orthopaedic Journal of Sports Medicine* 2 (7): 1–5. https://doi.org/10.1177/2325967114542775.

Anavian, J, S Guthrie, and P Cole. 2009. ‘Surgical Management of Multiple Painful Rib Nonunions in Patient With a History of Severe Shoulder Girdle Trauma: A Case Report and Literature Review’. *Journal of Orthopaedic Trauma* 23 (8): 600–604. https://doi.org/10.1097/BOT.0b013e3181a15e90.

Anderson, E, and B Spain. 2016. *The Child with Spina Bifida.* 2nd ed. Oxford: Routledge. https://doi.org/10.1017/CBO9781107415324.004.

Anderson, T. 2004. ‘Dental Treatment in Medieval England’. *British Dental Journal* 197: 291–292. https://doi.org/10.1038/sj.bdj.4811726.

Andrews, D.D. and Milne, G. 1979. *Wharram: A Study of Settlement on the Yorkshire Wolds. Volume I. Domestic Settlement, 1: Areas 10 and 6*. Society for Medieval Archaeology Monograph 8. London: Maney.

Apazidis, A., Ricart, P., Diefenbach, C., and Spivak, J. 2011. ‘The Prevalence of Transitional Vertebrae in the Lumbar Spine’. *The Spine Journal* 11 (9): 858–862. https://doi: 10.1016/j.spinee.2011.08.005.

Aprato, A, A Conti, F Bertolo, and A Massè. 2019. ‘Slipped Capital Femoral Epiphysis: Current Management Strategies’. *Orthopedic Research and Reviews* 11: 47–54. https://doi.org/10.2147/ORR.S166735.

Arden, N., Blanco, F., Bruyère, O., Cooper, A., Guermazi, A., Hayashi, D., Hunter, D., et al. 2018. *Atlas of Osteoarthritis*. 2nd ed. London: Springer Healthcare.

Arumugam, E, M Harinathbabu, R Thillaigovindan, and G Prabhu. 2015. ‘Marble Bone Disease: A Rare Bone Disorder’. *Cureus* 7 (10): 1–11. https://doi.org/10.7759/cureus.339.

Aufderheide, A, and C Rodriguez-Martin. 1998. *The Cambridge Encyclopedia of Human Paleopathology*. Cambridge: Cambridge University Press.

Bailey, J, and D Tapscott. 2020. ‘Osteopetrosis’. *StatPearls* [Internet]. https://www.ncbi.nlm.nih.gov/books/NBK557529/

Ball, D. 2009. ‘Medullary Thyroid Cancer’. In *Clinical Management of Thyroid Disease,* edited by Wondisford, F. and Radovick, S., 399–405. Philadelphia: Saunders, Elsevier.

Bandeira, F., Cusano, N., Silva, B., Cassibba, S., Almeida, C., Machado, V., and Bilezikian, J. 2014. ‘Bone Disease in Primary Hyperparathyroidism’. *Arquivos Brasileiros de Endocrinologia & Metabologia* 58 (5): 553–561. https://doi:10.1590/0004-2730000003381

Barnes, E. 2012. ‘Developmental Disorders in the Skeleton’. In *A Companion to Paleopathology*, edited by A Grauer, 380–400. Chichester: Wiley-Blackwell.

Bauer, T., David, T., Rimareix, F., and Lortat-Jacob, A. 2007. ‘Marjolin’s Ulcer in Chronic Osteomyelitis: Seven Cases and a Review of the Literature’. *Revue de Chirurgie Orthopedique et Reparatrice de l’Appareil Moteur* 93 (1): 63–71. https://doi: 10.1016/s0035-1040(07)90205-6.

Beck, M., Kalhor, M., Leunig, M., and Ganz, R. 2005. ‘Hip Morphology Influences the Pattern of Damage to the Acetabular Cartilage. Femoroacetabular Impingement as a Cause of Early Osteoarthritis of the Hip’. *Journal of Bone and Joint Surgery* Series B 87 (7): 1012–1018. https://doi: 10.1302/0301-620X.87B7.15203.

Belanger, T. and Rowe, D. 2001. ‘Diffuse Idiopathic Skeletal Hyperostosis: Musculoskeletal Manifestations’. *Journal of the American Academy of Orthopaedic Surgeons* 9 (2): 258–267. https://doi: 10.5435/00124635-200107000-00006

Benjamin, M., Ralphs, J., Bydder, G., Best, T., and Milz, S. 2006. ‘Where Tendons and Ligaments Meet Bone- Attachment Sites (“Entheses”) in Relation to Exercise and/or Mechanical Load’. *Journal of Anatomy* 208 (4): 471–490. https://doi: 10.1111/j.1469-7580.2006.00540.x

Benlidayi, I., Coskun, N., and Basaran, S. 2015. ‘Does Lumbosacral Transitional Vertebra Have Any Influence on Sacral Tilt?’ Spine 40 (22): 1176–1179. https://doi: 10.1097/BRS.0000000000001117

Beresford, G. 1975. *The Medieval Clay-Land Village: Excavations at Goltho and Barton Blount.* Society for Medieval Archaeology Monograph 6. London: Society for Medieval Archaeology.

Blakey, M, T Leslie, and J Reidy. 1994. ‘Frequency and Chronological Distribution of Dental Enamel Hypoplasia in Enslaved African Americans: A Test of the Weaning Hypothesis’. *American Journal of Physical Anthropology* 95 (4): 371–383. https://doi: 10.1002/ajpa.1330950402.

Blom, A.A., Inskip, S.A., Baetesen, W.A., and Hoogland, M.L.P. 2018. ‘Testing the Sternal Clavicle Ageing Method on a Post-Medieval Dutch Skeletal Collection’. *Archaeometry* 60 (6): 1391–1402. https://doi.org/10.1111/arcm.12402.

Bogduk, N, M Pearcy, and G Hadfield. 1992. ‘Mechanics and Biomechanics of Psoas Major’. *Clinical Biomechanics* 7 (2): 109–119. https://doi: 10.1016/0268-0033(92)90024-X.

Borman, A., Campbell, C., Fraser, M. and Johnson, E. 2007. ‘Analysis of the Dermatophyte Species Isolated in the British Isles Between 1980 and 2005 and Review of Worldwide Dermatophyte Trends Over the Last Three Decades’. *Medical Mycology* 45: 131–141.

Bowen, W, and S Pearson. 1993. ‘Effect of Milk on Cariogenesis’. *Caries Research* 27: 461–66. https://doi.org/10.1159/000261581.

Brasel, K, C Guse, P Layde, and J Weigelt. 2006. ‘Rib Fractures: Relationship with Pneumonia and Mortality’. *Critical Care Medicine* 34 (6): 1642–1646. https://doi.org/10.1097/01.CCM.0000217926.40975.4B.

Bronk Ramsey C. 2009. ‘Bayesian Analysis of Radiocarbon Dates’. *Radiocarbon* 51 (1): 337–360. https://doi.org/10.1017/S0033822200033865.

Bronk Ramsey C. and Lee, S. 2013. ‘Recent and Planned Developments of the Program OxCal’. *Radiocarbon* 55 (2–3): 720–730. https://doi.org/10.1017/S0033822200057878.

Brooks, S, and J Suchey. 1990. ‘Skeletal Age Determination Based on the Os Pubis: A Comparison of the Acsádi-Nemeskéri and Suchey-Brooks Methods’. *Human Evolution* 5 (3): 227–238. https://doi.org/10.1007/BF02437238.

Brothwell, D. 1981. *Digging up Bones*. 3rd ed. Ithaca, New York: Cornell University Press.

Buckberry, J, and A Chamberlain. 2002. ‘Age Estimation from the Auricular Surface of the Ilium: A Revised Method’. *American Journal of Physical Anthropology* 119 (3): 231–239. https://doi.org/10.1002/ajpa.10130.

Bugaev, N, J Breeze, M Alhazmi, H Anbari, S Arabian, S Holewinski, and R Rabinovici. 2016. ‘Magnitude of Rib Fracture Displacement Predics Opiod Requirements’. *Journal of Trauma and Acute Care Surgery* 81 (4): 699–704. https://doi.org/10.1016/j.physbeh.2017.03.040.

Buikstra, J, D Ubelaker, and D Aftandilian, eds. 1994. *Standards for Data Collection from Human Skeletal Remains. Proceedings of a Seminar at The Field Museum of Natural History. Arkansas Archaeological Survey Report*. Fayetteville: Arkansas Archaeological Survey.

Cai, X, S Yan, and G Giddins. 2013. ‘A Systematic Review of the Non-Operative Treatment of Nightstick Fractures of the Ulna’. *Bone and Joint Journal* 95 B (7): 952–959. https://doi.org/10.1302/0301-620X.95B7.31669.

Cąkar, M, C Esenyel, M Seyran, A Tekin, M Adaş, M Bayraktar, and Ü Coşkun. 2015. ‘Osteoid Osteoma Treated with Radiofrequency Ablation’. *Advances in Orthopedics* 2015, 1–5. https://doi.org/10.1155/2015/807274.

Calce, S. 2012. ‘A New Method to Estimate Adult Age-at-Death Using the Acetabulum.’ *American Journal of Physical Anthropology* 148 (1): 11–23. https://doi.org/10.1002/ajpa.22026.

Capes, W.W. 1912. *The Register of Thomas de Charlton, Bishop of Hereford AD1327−1344*. Canterbury and York Society 9. Hereford.

Cashmore, L. and Zakrzewski, S. 2013. ‘Assessment of Musculoskeletal Stress Marker Development in the Hand’. *International Journal of Osteoarchaeology* 23 (3): 334–347. https://doi.org/10.1002/oa.1254.

Castells Navarro, L. and Buckberry, J. 2020. ‘Back to the Beginning: Identifying Lesions of Diffuse Idiopathic Skeletal Hyperostosis Prior to Vertebral Ankylosis’. *International Journal of Paleopathology* 28: 59–68. https://doi: 10.1016/j.ijpp.2019.12.004

Cava, Antonio La. 2017. ‘Leptin in Inflammation and Autoimmunity’. *Cytokine* 98: 51–58. https://doi.org/10.1016/j.cyto.2016.10.011.

Cawley, D, F Power, and M Murphy. 2016. ‘Cerebrospinal Fluid Leak After Coccyx Fracture’. *Spine Journal* 16 (11): e735–736. https://doi.org/10.1016/j.spinee.2016.03.055.

Cessford, C., A. Hall, B. Mulder, B. Neil, I. Riddler and J. Wiles. forthcoming. “Buried with Their Buckles On: Clothed Burial at the Augustinian Friary, Cambridge, and Corporate and Individual Identity.” *Medieval Archaeology* 66 (1).

Cessford, C. and Alexander, C. in prep. ‘The Radiocarbon Dating Program and Bayesian Analysis’. In *After the Plague: Health and History in Medieval Cambridge*, by Robb, J.E. Cambridge: McDonald Institute monograph.

Chaoui-Derieux, D. 2010. ‘Socio-Economic and Cultural Implications in Medieval Society: The Unpublished Collections of the Region of Douai (France)’. In *Ancient and Modern Bone Artefacts from America to Russia : Cultural, Technological and Functional Signatures*, edited by Legrand-Pineau, A., Sidéra, I., Buc, N., David, E., and Scheinsohn, V., 65–70. British Archaeological Reports International Series 2136. Oxford: Archaeopress.

Chattopadhyay, P., Bandyopadhyay, A., Ghosh, S., and Kundu, A. 2009. ‘Primary Diaphyseal Tuberculosis of the Tibia’. *Singapore Medical Journal* 50 (6): 226–228.

Chaudhry, H. and Ayeni, O. 2014. ‘The Etiology of Femoroacetabular Impingement: What We Know and What We Don’t’. *Sports Health* 6 (2): 157–161. https://doi: 10.1177/1941738114521576.

Chazottes, M-A. and Thuaudet, O., 2014. ‘L’utilisation Des Matières Dures d’origine Animale Dans La Production d’accessoires de La Ceinture à La Fin Du Moyen Âge : Quelques Exemples Provençaux’. *Archéologie Du Midi Médiéval* 32: 183–198.

Chen, H. 2017. *Atlas of Genetic Diagnosis and Counseling*. 3rd ed. Springer Science+Business Media. https://doi.org/10.1007/978-1-4614-1037-9.

Ciampolini, J. and Harding, K. 2000. ‘Pathophysiology of Chronic Bacterial Osteomyelitis. Why Do Antibiotics Fail So Often?’ *Postgraduate Medical Journal* 76 (898): 479–483. https://doi: 10.1136/pmj.76.898.479.

Circi, E., Atalay, Y., and Beyzadeoglu, T. 2017. ‘Treatment of Osgood–Schlatter Disease: Review of the Literature’. *Musculoskeletal Surgery* 101 (3): 195–200. . https://doi: 10.1007/s12306-017-0479-7.

Clarke, H. 1999. ‘Toothaches and Death’. *Journal of the History of Dentistry* 47 (1): 11–13.

Cole, G, and T Waldron. 2019. ‘Cribra Orbitalia: Dissecting an Ill-Defined Phenomenon’. *International Journal of Osteoarchaeology* 29 (4): 613–621. https://doi.org/10.1002/oa.2757.

Court-Brown, C, J Heckman, M McQueen, W Ricci, P Tornetta, and M McKee, eds. 2015. *Rockwood and Green’s Fractures in Adults*. 8th ed. London: Wolters Kluwer.

Cramer, G, and S Darby. 2014. C*linical Anatomy of the Spine, Spinal Cord, and ANS*. 3rd ed. Elsevier Mosby.

Crubézy, E. 1992. ‘Sternal Foramina: Problems Arising from the Study of a Family’. *International Journal of Anthropology* 7 (4): 1–7. https://doi.org/10.1007/BF02447864.

Cruz, A. and Starke, J. 2007. ‘Clinical Manifestations of Tuberculosis in Children’. *Paediatric Respiratory Reviews* 8 (2), 107–117. https://doi.org/10.1016/j.prrv.2007.04.008.

Cullum, P. 2004. ‘Boy/Man into Clerk/Priest: The Making of the Late Medieval Clergy’. In *Rites of Passage. Cultures of Transition in the Fourteenth Century*, edited by McDonald, N. and Ormrod, W., 51–65. York: York Medieval Press.

Cunningham, C, L Scheuer, S Black, H Liversidge, and A Christie. 2016a. Developmental Juvenile Osteology. 2nd ed. London: Academic Press.

Curtis, B., Huang, B., and Smitaman, E. 2019. ‘Pes Anserinus: Anatomy and Pathology of Native and Harvested Tendons’. *American Journal of Roentgenology* 213 (5): 1107–1116. https://doi: 10.2214/AJR.19.21315.

DaCambra, M., Gupta, S., and Ferri-De-Barros, F. 2014. ‘Subungual Exostosis of the Toes: A Systematic Review’. *Clinical Orthopaedics and Related Research* 472 (4): 1251–1259. . https://doi: 10.1007/s11999-013-3345-4

Daniell, C. 1997. *Death and Burial in Medieval England 1066-1550*. London: Routledge.

Dash, C. 2019. ‘Cervicothoracic Junction Fractures Management: An Overview of Literature’. *Indian Journal of Neurotrauma* 16 (1): 27–32. https://doi.org/10.1055/s-0039-1700621.

David, L, S Glazier, J Pyle, J Thompson, and L Argenta. 2009. ‘Classification System for Sagittal Craniosynostosis’. *Journal of Craniofacial Surgery* 20 (2): 279–282. https://doi.org/10.1097/SCS.0b013e3181945ab0.

de Meijer, A. 1955. ‘John Capgrave, O.E.S.A.’ *Augustiniana* 5: 400–440.

Debernardi, A., D’Aliberti, G., Talamonti, G., Villa, F., and Collice, M. 2011. The Craniovertebral Junction Area and the Role of the Ligaments and Membranes Neurosurgery 68 (2): 291–301. https://doi: 10.1227/NEU.0b013e3182011262.

Dewitte, S, and J Bekvalac. 2011. ‘The Association between Periodontal Disease and Periosteal Lesions in the St. Mary Graces Cemetery, London, England A.D. 1350–1538’. *American Journal of Physical Anthropology* 146 (4): 609–618. https://doi.org/10.1002/ajpa.21622.

Djukic, K, N Miladinovic-Radmilovic, M Draskovic, and M Djuric. 2018. ‘Morphological Appearance of Muscle Attachment Sites on Lower Limbs: Horse Riders Versus Agricultural Population’. *International Journal of Osteoarchaeology* 28 (6): 656–668. https://doi.org/10.1002/oa.2680.

Doo, A., Lee, J., Yeo, G., Lee, K., Kim, Y., Mun, J., Han, Y., and Son, J. 2020. ‘The Prevalence and Clinical Significance of Transitional Vertebrae: A Radiologic Investigation Using Whole Spine Spiral Three-Dimensional Computed Tomographic Images’*. Anesthesia and Pain Medicine* 15 (1): 103–110. https://doi: 10.17085/apm.2020.15.1.103.

Dorobisz, K., Dorobisz, T., Janczak, D., and Krecicki, T. 2019. ‘The Evaluation of the Sense of Hearing in Patients with Carotid Artery Stenosis within the Extracranial Segments’. *Acta Neurologica Belgica* 119 (3): 385–392. https://doi: 10.1007/s13760-018-01058-3.

Du Plessis, A., Greyling, L., and Page, B. 2018. ‘Differentiation and Classification of Thoracolumbar Transitional Vertebrae’. *Journal of Anatomy* 232 (5): 850–856. https://doi.org/10.1111/joa.12781.

Du Plessis, A.M. 2018. ‘A Supernumeric Thoracic Vertebra Associated with Neural Tube Defects’. *Clinical and Experimental Anatomy* 1 (1894): 31–39.

Duckworth, W.L.H. and Pocock, W.I. 1910. ‘On the Human Bones Found on the Site of the Augustinian Friary, Bene’t Street, Cambridge’. *Proceedings of the Cambridge Antiquarian Society* 22: 53–75.

Dumond, H, N Presle, B Terlain, D Mainard, D Loeuille, P Netter, and P Pottie. 2003. ‘Evidence for a Key Role of Leptin in Osteoarthritis’. *Arthritis and Rheumatism* 48 (11): 3118–3129. https://doi.org/10.1002/art.11303.

Dunbar, E., Cook, G.T., Naysmith, P., Tripney, B.G., and Xu, S. 2016. ‘AMS 14C Dating at the Scottish Universities Environmental Research Centre (SUERC) Radiocarbon Dating Laboratory’. *Radiocarbon* 58 (1): 9–23. https://doi.org/10.1017/RDC.2015.2.

Dupras, T., and J Schultz. 2014. ‘Taphonomic Bone Staining and Color Changes in Forensic Contexts’. In *Manual of Forensic Taphonomy*, edited by J Pokines and S Symes, 215–340. London: CRC Press.

Dymond, I. 1984. ‘The Treatment of Isolated Fractures of the Distal Ulna’. *Journal of Bone and Joint Surgery* Series B 66 (3): 408–410. https://doi.org/10.1302/0301-620x.66b3.6725352.

Edeiken, J., DePalma, A., Moskowitz, H., and Smythe, V. 1963. ‘“Cystic” Tuberculosis of Bone’. *Clinical Orthopaedics and Related Research* 28: 163–168.

Edwards, J, and W Hunt. 2018. ‘Non-Union’. In *Rib Fracture Management. A Practical Manual*, edited by M de Moya and J Mayberry, 123–134. Elsevier.

Egan G. and Pritchard F. 2002. *Dress Accessories c. 1150–c. 1450. Medieval Finds from Excavations in London*. Woodbridge: Boydell & Brewer.

Ehara, S, T Shimamura, R Nakamura, and K Yamazaki. 1998. ‘Paravertebral Ligamentous Ossification: DISH, OPLL and OLF’. *European Journal of Radiology* 27 (3): 196–205. https://doi.org/10.1016/S0720-048X(97)00164-2.

Eijer, H. and Hogervorst, T. 2017. ‘Femoroacetabular Impingement Causes Osteoarthritis of the Hip by Migration and Micro-Instability of the Femoral Head’. *Medical Hypotheses* 104: 93–96.

Ejnisman, L., Philippon, M., Lertwanich, P., Pennock, A., Herzog, M., Briggs, K., and Ho, C. 2013. ‘Relationship Between Femoral Aanteversion and Findings in Hips with Femoroacetabular Impingement’. *Orthopedics* 36 (3): 293–300. https://doi: 10.3928/01477447-20130222-17.

Emden, A.B. 1963. *A Biographical Register of the University of Cambridge to 1500.* Cambridge: Cambridge University Press, 1963.

Eshed, V, B Latimer, C Greenwald, L Jellema, B Rothschild, S Wish-Baratz, and I Hershkovitz. 2002. ‘Button Osteoma: Its Etiology and Pathophysiology’. *American Journal of Physical Anthropology* 118 (3): 217–230. https://doi.org/10.1002/ajpa.10087.

Falys, C, and D Prangle. 2015. ‘Estimating Age of Mature Adults from the Degeneration of the Sternal End of the Clavicle’. *American Journal of Physical Anthropology* 156 (2): 203–214. https://doi.org/10.1002/ajpa.22639.

Falys, C. and Prangle, D. 2015. ‘Estimating Age of Mature Adults from the Degeneration of the Sternal End of the Clavicle’. *American Journal of Physical Anthropology* 156 (2): 203–214. https://doi: 10.1002/ajpa.22639

Faraday, M.A. 1991. Ludlow 1085−1660: *A Social, Economic and Political History.* Chichester: Phillimore.

Fatayri, B El, A Djebara, A Fourdrain, Y Bulaid, and M Sanguina. 2019. ‘Resection of a Rare Metacarpal Distal Condyle Osteoid Osteoma’. *Case Reports in Orthopedics* 2019: 1–6. https://doi.org/10.1155/2019/4542862.

Findlay, D, and J Kuliwaba. 2016. ‘Bone-Cartilage Crosstalk: A Conversation for Understanding Osteoarthritis’. *Bone Research* 4: 1–12. https://doi.org/10.1038/boneres.2016.28.

Foye, P, J Shah, and D Sinha. 2017. ‘Coccyx Fracture and Dislocation’. In *Musculoskeletal Sports and Spine Disorders. A Comprehensive Guide*, edited by S Kahn and R Xu, 161–163. New York: Springer.

France, D. 1998. ‘Observational and Metric Analysis of Sex in the Skeleton’. In *Forensic Osteology: Advances in the Identification of Human Remains*, edited by KJ Reichs, 2nd ed., 163–86. Springfield: Charles C Thomas Publisher.

Franklyn, M, and B Oakes. 2015. ‘Aetiology and Mechanisms of Injury in Medial Tibial Stress Syndrome: Current and Future Developments’. *World Journal of Orthopaedics* 6 (8): 577–589. https://doi.org/10.5312/wjo.v6.i8.577.

Fredeman, J. 1979. ‘The Life of John Capgrave, O.E.S.A. (1393–1464)’. *Augustiniana* 29: 197–237.

Gaborit-Chopin, D. 2003. *Ivoires Médiévaux Ve–XVe Siècle, Musée Du Louvre, Département Des Objets d’Art, Paris*. Paris: Éditions de la Réunion des Musées Nationaux.

Gabriel, B, and J Zierath. 2017. ‘The Limits of Exercise Physiology: From Performance to Health’. *Cell Metabolism* 25 (5): 1000–1011. https://doi.org/10.1016/j.cmet.2017.04.018.

Gayk, S. 2010. ‘John Capgrave’s Material Memorials’. In *Image, Text, and Religious Reform in Fifteenth-Century England*, edited by Gayk, S., 123–54. Cambridge: Cambridge University Press.

George, J., Acharya, S., Bandgar, T., Menon, P., and Shah, N. 2010. ‘Primary Hyperparathyroidism in Children and Adolescents’. *Indian Journal of Pediatrics* 77: 175–178.

Gholve, P., Scher, D., Khakharia, S., Widmann, R., and Green, D. 2007. ‘Osgood Schlatter Syndrome’. *Current Opinion in Pediatrics* 19 (1): 44–50. https://doi: 10.1097/MOP.0b013e328013dbea.

Giuffra, V, S Minozzi, G Riccomi, A Naccarato, M Castagna, R Lencioni, S Chericoni, V Mongelli, and C Felici. 2019. ‘Multiple Osteomata from Medieval Tuscany, Italy (ca. 10th-12th AD)’. *International Journal of Paleopathology* 25: 56–61. https://doi.org/10.1016/j.ijpp.2019.04.003.

Gokce, C., Sisman, Y., and Sipahioglu, M. 2008. ‘Styloid Process Elongation or Eagle’s Syndrome: Is There Any Role for Ectopic Calcification?’ *European Journal of Dentistry* 2 (3): 224–228.

Goldring, S, and M Goldring. 2016. ‘Changes in the Osteochondral Unit During Osteoarthritis: Structure, Function and Cartilage Bone Crosstalk’. *Nature Reviews Rheumatology* 12 (11): 632–644. https://doi.org/10.1038/nrrheum.2016.148.

Gossner, J. 2013. ‘Relationship of Sternal Foramina to Vital Structures of the Chest: A Computed Tomographic Study’. *Anatomy Research International* 2013: 1–4. https://doi.org/10.1155/2013/780193.

Groh, M, and J Herrera. 2009. ‘A Comprehensive Review of Hip Labral Tears’. *Current Reviews in Musculoskeletal Medicine* 2 (2): 105–17. https://doi.org/10.1007/s12178-009-9052-9.

Gulcelik, N., Bozkurt, F., Tezel, G., Kaynaroglu, V., and Erbas, T. 2009. ‘Normal Parathyroid Hormone Levels in a Diabetic Patient with Parathyroid Adenoma’. *Endocrine* 35 (2): 147–50. https://doi: 10.1007/s12020-008-9135-1.

Gupta, A., Horattas, M., Moattari, A., and Shorten, S. 2001. ‘Disseminated Brown Tumors from Hyperparathyroidism Masquerading as Metastatic Cancer: A Complication of Parathyroid Carcinoma’. *The American Surgeon* 67 (10): 951–955.

Hack, K, G Di Primio, K Rakhra, and P Beaulé. 2010. ‘Prevalence of Cam-Type Femoroacetabular Impingement Morphology in Asymptomatic Volunteers’. *The Journal of Bone and Joint Surgery-American Volume* 92 (14): 2436–2444. https://doi.org/10.2106/JBJS.J.01280.

Hardcastle, S, P Dieppe, C Gregson, N Arden, T Spector, D. Hart, M Edwards, et al. 2014. ‘Osteophytes, Enthesophytes, and High Bone Mass: A Bone-Forming Triad with Potential Relevance in Osteoarthritis’. *Arthritis and Rheumatology* 66 (9): 2429–2439. https://doi.org/10.1002/art.38729.

Harper-Bill, C. 1991. *The Cartulary of the Augustinian Friars of Clare*. Woodbridge: Boydell.

Hatzenbuehler, J. and Pulling, T. 2011. ‘Diagnosis and Management of Osteomyelitis’. *American Family Physician* 84 (9): 1027–1033.

Heary, R., Albert, T., Ludwig, S., Vaccaro, A., Wolansky, L., Leddy, T., and Schmidt, R. 1996. ‘Surgical Anatomy of the Vertebral Arteries’. Spine 21 (18): 2074–80. https://doi: 10.1097/00007632-199609150-00004.

Hillson, S. 2002. *Dental Anthropology*. 3rd ed. Cambridge: Cambridge University Press. https://doi.org/10.1017/CBO9781107415324.004.

Hillson, S. 2008. ‘Dental Pathology’. In *Biological Anthropology of the Human Skeleton*, edited by M. Katzenberg and S Saunders, 2nd ed. 301–340. Hoboken: Wiley-Liss.

Holder, J, S Kolla, and S Lehto. 2017. ‘Clavicle Fractures: Allman and Neer Classification’. *Journal of Advances in Radiology and Medical Imaging* 2 (1): 1–11. https://doi.org/10.15744/2456-5504.2.102.

Hunter, D, and D Felson. 2006. ‘Osteoarthritis’. *British Medical Journal* 332 (42): 639–642. https://doi: 10.1136/bmj.332.7542.639.

Ikeda, T, G Awaya, S Suzuki, Y Okada, and H Tada. 1988. ‘Torn Acetabular Labrum in Young Patients. Arthroscopic Diagnosis and Management’. *The Journal of Bone and Joint Surgery* 70 (1): 13–16. https://doi.org/10.1302/0301-620x.70b1.3339044.

Jadeja, N. and Nalleballe, K. 2018. ‘Pearls & Oy-Sters: Bow Hunter Syndrome: A Rare Cause of Posterior Circulation Stroke. Do Not Look The Other Way’. *Neurology* 91 (7): 329–331. https://doi: 10.1212/WNL.0000000000006009.

Janssens, P. 1960. ‘Le Squelette Néolithique d’Avennes Sa Perforation Sternale’. *Societe Royale Belge D’Anthropologie et de Prehistoire* 71: 43–46.

Jayker, S., Prakash, C., Arkeswara, M., Ansari, M., Hashim, S., and Parthasarathi Achappa, P. 2017. ‘Osteitis Fibrosa Cystica of Tibia as Initial Manifestation of Primary Hyperparathyroidism’. *Journal of Case Reports* 7 (1): 1–4. http://doi: 10.17659/01.2017.0001

Jong, T, and P De Jong. 1989. ‘Ulnar-Shaft Fracture Needs No Treatment: A Pilot Study of 10 Cases’. *Acta Orthopaedica* 60 (3): 263–64. https://doi.org/10.3109/17453678909149273.

Judd, M. 2008. ‘The Parry Problem’. *Journal of Archaeological Science* 35 (6): 1658–1666. https://doi.org/10.1016/j.jas.2007.11.005.

Kabbani, H, and T Raghuveer. 2004. ‘Craniosynostosis’. American Family Physician 69 (12): 2863–70. https://doi.org/10.1097/01.ccn.0000457312.34253.a5.

Karthikeyan, K. 2009. ‘Tonsuring: Myths and Facts’. *International Journal of Tricholog*y 1 (1): 33–34. https://doi: 10.4103/0974-7753.51927

Kassebaum, N, E Bernabé, M Dahiya, B Bhandari, C Murray, and W Marcenes. 2015. ‘Global Burden of Untreated Caries: A Systematic Review and Metaregression’. *Journal of Dental Research* 94 (5): 650–658. https://doi.org/10.1177/0022034515573272.

Katz, J, S Agrawal, and M Velasquez. 2010. ‘Getting to the Heart of the Matter: Osteoarthritis Takes Its Place as Part of the Metabolic Syndrome’. *Current Opinion in Rh eumatology* 22 (5): 512–519. https://doi10.1097/.org/BOR.0b013e32833bfb4b.

Katzenberg, M, and A Grauer, eds. 2019. *Biological Anthropology of the Human Skeleton.* 3rd ed. Hoboken: Wiley-Blackwell.

Katzenberg, M, D Herring, and S Saunders. 1996. ‘Weaning and Infant Mortality: Evaluating the Skeletal Evidence’. *Yearbook of Physical Anthropology* 39: 177–99.

Katzman, W., Huang, M., Kritz-Silverstein, D., Barrett-Connor, E., and Kado, D. 2017. ‘Diffuse Idiopathic Skeletal Hyperostosis (DISH) and Impaired Physical Function: The Rancho Bernardo Study’. *Journal of the American Geriatrics Society* 65 (7): 1476–1481. https://doi: 10.1111/jgs.14810

Kawahara, H, H Baba, M Wada, M Azuchi, M Ando, and S Imura. 1997. ‘Multiple Rib Fractures Associated with Severe Coughing - A Case Report’. *International Orthopaedics* 21 (4): 279–281. https://doi: 10.1007/s002640050168

Kelempisioti, A., Eskola, P., Okuloff, A., Karjalainen, U., Takatalo, J., Daavittila, I., Niinimäki, J., et al. 2011. ‘Genetic Susceptibility of Intervertebral Disc Degeneration Among Young Finnish Adults’. *BMC Medical Genetics* 12: 2–9. https://doi: 10.1186/1471-2350-12-153

Kelleher, R. 2018. ‘Pilgrims, Pennies and the Ploughzone, Folded Coins in Medieval Britain’. In *Divina Moneta: Coins in Religion and Ritual*., edited by Burström, N.M. and Ingvardson, G.T., 68−86. Abingdon: Routledge.

Kim, B., Dan, J., and Shin, D. 2015. ‘Treatment of Thoracolumbar Fracture’. *Asian Spine Journal* 9 (1): 133–146. https://doi: 10.4184/asj.2015.9.1.133

Kim, Saeyoung, and Seungwon Jang. 2018. ‘Radicular Pain Caused by Schmörl’s Node: A Case Report’. *Brazilian Journal of Anesthesiology* 68 (3): 322–324. https://doi.org/10.1016/j.bjan.2017.07.007.

Klaus, H. 2017. ‘Paleopathological Rigor and Differential Diagnosis: Case Studies Involving Terminology, Description, and Diagnostic Frameworks for Scurvy in Skeletal Remains’. *International Journal of Paleopathology* 19: 96–110. https://doi.org/10.1016/j.ijpp.2015.10.002.

Knox, R. and Leslie, S. 1923. *The Miracles of King Henry VI: Being an Account and Translation of Twenty-Three Miracles Taken from the Manuscript in the British Museum (Royal 13 c. Viii).* Cambridge: Cambridge University Press.

Knüsel, C, and A Outram. 2004. ‘Fragmentation: The Zonation Method Applied to Fragmented Human Remains from Archaeological and Forensic Contexts’. *Environmental Archaeology* 9 (1): 85–97. https://doi.org/10.1179/env.2004.9.1.85.

Konin, G, and D Walz. 2010. ‘Lumbosacral Transitional Vertebrae: Classification, Imaging Findings and Clinical Relevance’. *American Journal of Neuroradiology* 31 (10): 1778–1786. https://doi.org/10.3174/ajnr.A2036.

Kortyna, R. 2017. ‘Diffuse Idiopathic Skeletal Hyperostosis: A Review’. *Journal of Orthopaedics for Physician Assistants* 5 (4): 1–6. https://doi 10.2106/JBJS.JOPA.17.00009

Koyfman, A. and Yaffe, D. 2014. ‘Crowned Dens Syndrome: A Case Report, Neuroradiology’. *Neuroradiology Journal* 27 (4): 495–497. https://doi: 10.15274/NRJ-2014-10056.

Kozłowski, T, and H Witas. 2012. ‘Metabolic and Endocrine Diseases’. In *A Companion to Paleopathology*, 401–419. New York: Wiley-Blackwell.

Kraan, P van der, and W van den Berg. 2007. ‘Osteophytes: Relevance and Biology’. *Osteoarthritis and Cartilage* 15 (3): 237–44. https://doi.org/10.1016/j.joca.2006.11.006.

Kumar, A. and Tubbs, R. 2011. ‘Spina Bifida: A Diagnostic Dilemma in Paleopathology’. *Clinical Anatomy* 24 (1): 19–33. https://doi: 10.1002/ca.21058

Lane, M, N Nahm, and H Vallier. 2015. ‘Morbidity and Mortality of Bilateral Femur Fractures’. *Orthopedics* 38 (7): e588-592. https://doi.org/10.3928/01477447-20150701-56.

Lanphear, K. 1990. ‘Frequency and Distribution of Enamel Hypoplasias in a Historic Skeletal Sample’. *American Journal of Physical Anthropology* 81 (1): 35–43. https://doi: 10.1002/ajpa.1330810106.

Larsen, C. 2015. *Bioarchaeology: Interpreting Behavior from the Human Skeleton*. 2nd ed. Cambridge: Cambridge University Press. https://doi.org/10.1002/(SICI)1520-6300(1999)11:3<417::AID-AJHB17>3.0.CO;2-L.

Ledingham, D., Cappelen-Smith, C., and Cordato, D. 2018. ‘Crowned Dens Syndrome’. *Practical Neurology* 18 (1): 57–59. http://dx.doi.org/10.1136/practneurol-2017-001793

Lee, C, and K Porter. 2005. ‘Prehospital Management of Lower Limb Fractures’. *Emergency Medicine Journal* 22 (9): 660–63. https://doi.org/10.1136/emj.2005.024489.

Lee, J, Y Gil, K Shin, J Kim, S Joo, K Koh, and W Song. 2016. ‘An Anatomical and Morphometric Study of the Coccyx Using Three-Dimensional Reconstruction’. *Anatomical Record* 299 (3): 307–12. https://doi.org/10.1002/ar.23300.

Lemont, H. and Goss, L. 2005. ‘Subungual Exostosis, Nail Disease and Radiologic Considerations’. In *Nails. Diagnosis, Therapy, Surgery*, edited by Scher, R. and Daniel, C., 31–36. London: Elsevier Saunders.

Leunig, M., Beaulé, P., and Ganz, R. 2009. ‘The Concept of Femoroacetabular Impingement: Current Status and Future Perspectives’. *Clinical Orthopaedics and Related Research* 467 (3): 616–622. https://doi: 10.1007/s11999-008-0646-0

Lian, J., Levine, N., and Cho, W. 2018. ‘A Review of Lumbosacral Transitional Vertebrae and Associated Vertebral Numeration’. *European Spine Journal* 27 (5): 995–1004. https://doi: 10.1007/s00586-018-5554-8.

Lieverse, A. 1999. ‘Diet and the Aetiology of Dental Calculus’. *International Journal of Osteoarchaeology* 9 (4): 219–32. https://doi.org/10.1002/(SICI)1099-1212(199907/08)9:4<219::AID-OA475>3.0.CO;2-V.

Lovell, N. 1997. ‘Trauma Analysis in Paleopathology’. *American Journal of Physical Anthropology* 40: 139–170. https://doi.org/10.1002/(SICI)1096-8644(1997)25+<139::AID-AJPA6>3.0.CO;2-%23.

Lundstrom, K. 2020. *Nutrition and Disease: Prevention and Therapy*. Cambridge: Cambridge Scholars Publishing.

MacGregor, A. 1985. *Bone, Antler, Ivory and Horn. The Technology of Skeletal Materials since the Roman Period*. London: Croom Helm.

MacGregor, A.. 1991. ‘Antler, Bone and Horn’. In *English Medieval Industries*, edited by Blair, J. and Ramsay, N., 355–78. London: Hambledon Press.

Mackay, D, L Wood, and A Rangan. 2000. ‘The Treatment of Isolated Ulnar Fractures in Adults: A Systematic Review’. *Injury* 31 (8): 565–570. https://doi.org/10.1016/S0020-1383(00)00051-6.

Mader, R, and I Lavi. 2009. ‘Diabetes Mellitus and Hypertension As Risk Factors for Early Diffuse Idiopathic Skeletal Hyperostosis (DISH)’. *Osteoarthritis and Cartilage* 17 (6): 825–828. https://doi.org/10.1016/j.joca.2008.12.004.

Mader, R, J Verlaan, I Eshed, B Jacome, P Puttini, F Atzeni, D Buskila, et al. 2017. ‘Diffuse Idiopathic Skeletal Hyperostosis (DISH): Where We Are Now and Where To Go Next’. *RMD Open* 3 (1): 1–6. https://doi.org/10.1136/rmdopen-2017-000472.

Magee, D, J Zachazewski, and W Quillen. 2009. *Pathology and Intervention in Musculoskeletal Rehabilitation.* Missouri: Saunders, Elsevier.

Magee, T, and G Hinson. 2000. ‘Association of Paralabral Cysts with Acetabular Disorders’. *American Journal of Roentgenology* 174 (5): 1381–1384. . https://doi: 10.2214/ajr.174.5.1741381.

Mageed, S., Al-Agam, A., Arafa, U., and Ahmed, A. 2017. ‘Musculoskeletal Disorders in Type 2 Diabetes Mellitus’, *Sohag Medical Journal* 21 (3): 255–260. https://doi: 10.21608/SMJ.2017.39739

Maigne, J, L Doursounian, and F Jacquot. 2020. ‘Classification of Fractures of the Coccyx From a Series of 104 Patients’. *European Spine Journal* 2020 (10): 2534–2542. https://doi.org/10.1007/s00586-019-06188-7.

Malizos, K, A Karantanas, S Varitimidis, Z Dailiana, K Bargiotas, and T Maris. 2007. ‘Osteonecrosis of the Femoral Head: Etiology, Imaging and Treatment’. *European Journal of Radiology* 63 (1): 16–28. https://doi.org/10.1016/j.ejrad.2007.03.019.

Mann, R. and Hunt, D. 2005. *Photographic Regional Atlas of Bone Disease. A Guide to Pathologic and Normal Variation in the Human Skeleton*. 3rd ed. Springfield: Charles C Thomas.

Mansfield, P. and Neumann, D. 2009. *Essentials of Kinesiology for the Physical Therapist*. 2nd ed. St. Louis: Mosby Elsevier.

Marasco, S, G Lee, R Summerhayes, M Fitzgerald, and M Bailey. 2015. ‘Quality of Life After Major Trauma with Multiple Rib Fractures’. *Injury* 46 (1): 61–65. https://doi.org/10.1016/j.injury.2014.06.014.

Masters, Elysia A., Ryan P. Trombetta, Karen L. de Mesy Bentley, Brendan F. Boyce, Ann Lindley Gill, Steven R. Gill, Kohei Nishitani, et al. 2019. ‘Evolving Concepts in Bone Infection: Redefining “Biofilm”, “Acute vs. Chronic Osteomyelitis”, “the Immune Proteome” and “Local Antibiotic Therapy”’. *Bone Research* 7 (1): 20. https://doi.org/10.1038/s41413-019-0061-z.

Masuda, S, K Namba, H Mutai, S Usui, Y Miyanaga, H Kaneko, and T Matsunaga. 2014. ‘A Mutation in the Heparin-Binding Site of Noggin as a Novel Mechanism of Proximal Symphalangism and Conductive Hearing Loss’. *Biochemical and Biophysical Research Communications* 447 (3): 496–502. https://doi.org/10.1016/j.bbrc.2014.04.015.

Mays, S. 2005. ‘Supra-Acetabular Cysts in a Medieval Skeletal Population’. *International Journal of Osteoarchaeology* 15 (4): 233–246. https://doi.org/10.1002/oa.778.

Mays, S. 2008. ‘A Likely Case of Scurvy from Early Bronze Age Britain’. *International Journal of Osteoarchaeology* 18: 178–187. https://doi.org/10.1002/oa.

Mays, S. 2012. ‘The Relationship Between Paleopathology and the Clinical Sciences’. In *A Companion to Paleopathology*, edited by Grauer, A., 285–309. Chichester: Wiley-Blackwell.

Mays, S. 2016. ‘Estimation of Stature in Archaeological Human Skeletal Remains from Britain’. *American Journal of Physical Anthropology* 161 (4): 646–655. https://doi.org/10.1002/ajpa.23068.

McCormick, W. 1981. ‘Sternal Foramena in Man’. *The American Journal of Forensic Medicine and Pathology* 2 (3): 249–252. https://doi: 10.1097/00000433-198109000-00011.

McCoy, J. 2012. ‘Wheels and Wycliffites: The Role of Sacred Images in Capgrave’s The Life of Saint Katherine’. *Fifteenth-Century Studies* 37: 97–112.

Mckinley, J. 2004. ‘Compiling a Skeletal Inventory: Disarticulated and Co-Mingled Remains’. In *Guidelines to the Standards for Recording Human Remains*. IFA Paper No. 7, edited by M Brickley and J McKinley, 14–17. BABAO, IFA.

Meester, L, and J Pantel. 2014. ‘Slipped Capital Femoral Epiphysis in Adults: Case Report and Review of Literature’. *Reumatismo* 68 (1): 40–47. https://doi.org/10.4081/jlimnol.2014.831.

Miller, A. 2012. ‘To “Frock” a Cleric: The Gendered Implications of Mutilating Ecclesiastical Vestments in Medieval England’. *Gender and History* 24 (2): 271–291. https://doi.org/10.1111/j.1468-0424.2012.01682.x.

Min, S, T Shi, X Han, D Chen, Z Xu, D Shi, and H Teng. 2021. ‘Serum Levels of Leptin, Osteopontin, and Sclerostin in Patients With and Without Knee Osteoarthritis’. *Clinical Rheumatology* 40: 287–294. https://doi.org/10.1007/s10067-020-05150-z.

Minisola, S., Gianotti, L., Bhadada, S., and Silverberg, S. 2018. ‘Classical Complications of Primary Hyperparathyroidism, Best Practice & Research’. *Clinical Endocrinology & Metabolism* 32 (6): 791–803. https://doi: 10.1016/j.beem.2018.09.001

Misiorowski, W., Czajka-Oraniec, I., Kochman, M., Zgliczyński, W., and Bilezikian, J. 2017. ‘Osteitis Fibrosa Cystica—A Forgotten Radiological Feature of Primary Hyperparathyroidism’. *Endocrine* 58 (2): 380–385. https://doi: 10.1007/s12020-017-1414-2.

Mitchiner, M. 1988. *Jetons, Medalets and Tokens. Vol. 1. The Medieval Period and Nuremberg*. London: BA Seaby.

Moffat, D., Ramsden, R., and Shaw, H. 1977. ‘The Styloid Process Syndrome: Aetiological Factors and Surgical Management’. *The Journal of Laryngology and Otology* 91 (4): 279–294. https://doi: 10.1017/s0022215100083699.

Moreno, S, M Reyes, and F Moreno. 2016. ‘Cusp Expression of Protostylid in Deciduous and Permanent Molars’. *Journal of Forensic Dental Sciences* 8 (3): 155–163. https://doi.org/10.4103/0975-1475.195108.

Morris, B., Varma, R., Garg, A., Awasthi, M., and Maheshwari, M. 2002. ‘Multifocal Musculoskeletal Tuberculosis in Children: Appearances on Computed Tomography’. *Skeletal Radiology* 31 (1): 1–8. <https://doi>: 10.1007/s00256-001-0439-y

Müderris, T., Bercin, S., Sevil, E., Beton, S., and Kiris, M. 2014. ‘Surgical Management of Elongated Styloid Process: Intraoral or Transcervical?’. *European Archives of Oto-Rhino-Laryngology* 271 (6): 1709–1713. https://doi: 10.1007/s00405-013-2664-0.

Mundlos, S. 2016. ‘NOG and Proximal Symphalangism (SYM1), Multiple Synostosis (SYN1), Tarsal- Carpal Coalition, and Isolated Stapes Ankylosis’. In *Epstein’s Inborn Errors of Development. The Molecular Basis of Clinical Disorders Of Morphogenesis,* edited by R Erickson and A Wynshaw-Boris, 381–383. Oxford: Oxford University Press. https://doi: 10.1093/med/9780199934522.003.0047

Murlimanju, B., Prabhu, L., Shilpa, K., Rai, R., Dhananjaya, K., and Jiji, P. 2011. ‘Accessory Transverse Foramina in the Cervical Spine: Incidence, Embryological Basis, Morphology and Surgical Importance’. *Turkish Neurosurgery* 21 (3): 384–387. https://doi: 10.5137/1019-5149.JTN.4047-10.0.

Murphey, M, J Choi, M Kransdorf, D Flemming, and F Gannon. 2000. ‘Imaging of Osteochondroma: Variants and Complications with Radiologic-Pathologic Correlation’. *Radiographics* 20 (5): 1407–1434. https://doi: 10.1148/radiographics.20.5.g00se171407

Musonza, T, and S Todd. 2018. ‘Medical Management of Rib Fractures’. In Rib Fracture Management. A Practical Manual, edited by M de Moya and J Mayberry, 55–67. Amsterdam: Elsevier. https://doi: 10.1007/978-3-319-91644-6_5

Naaman, R, A El-Housseiny, and N Alamoudi. 2017. ‘The Use of Pit and Fissure Sealants—A Literature Review’. *Dentistry Journal* 5 (4): 34. https://doi.org/10.3390/dj5040034.

Nair, C.G., M. Babu, P. Jacob, R. Menon, J. Mathew, and Unnikrishnan. 2016. ‘Renal Dysfunction in Primary Hyperparathyroidism; Effect of Parathyroidectomy: A Retrospective Cohort Study’. *International Journal of Surgery* 36: 383–387. https://doi.org/10.1016/j.ijsu.2016.11.009.

Nathan, S, B Fisher, and C Roberts. 2010. ‘Coccydynia: A Review of Pathoanatomy, Aetiology, Treatment and Outcome’. *Journal of Bone and Joint Surgery* Series B 92 B (12): 1622–1627. https://doi.org/10.1302/0301-620X.92B12.25486.

Nelson, G, and F Madimenos. 2010. ‘Obelionic Cranial Deformation in the Puebloan Southwest’. *American Journal of Physical Anthropology* 143 (3): 465–472. https://doi.org/10.1002/ajpa.21353.

Nelson, J. 2018. ‘An Examination of the Differential Susceptibility Pattern of the Dentition to Linear Enamel Hypoplasia’. *Compass* 2 (1): 54–69. https://doi.org/10.29173/comp49.

Niggemann, P, J Kuchta, D Grosskurth, H Beyer, J Hoeffer, and K Delank. 2012. ‘Spondylolysis and Isthmic Spondylolisthesis: Impact of Vertebral Hypoplasia on the Use of the Meyerding Classification’. *British Journal of Radiology* 85 (1012): 358–62. https://doi.org/10.1259/bjr/60355971.

Nirmala, S, K Gaddam, P Vimaladevi, and S Nuvvula. 2013. ‘Protostylid: A Case Series’. *Contemporary Clinical Dentistry* 4 (3): 349–352. https://doi.org/10.4103/0976-237x.118338.

Norén, A, N Lynnerup, A Czarnetzki, and M Graw. 2005. ‘Lateral Angle: A Method for Sexing Using the Petrous Bone’. *American Journal of Physical Anthropology* 128 (2): 318–323. https://doi.org/10.1002/ajpa.20245.

Noronha, A, J Modamio, Y Jarosz, E Guerard, N Sompairac, G Preciat, A Daníelsdóttir, et al. 2019. ‘The Virtual Metabolic Human Database: Integrating Human and Gut Microbiome Metabolism with Nutrition and Disease’. *Nucleic Acids Research* 47 (D1): D614–624. https://doi.org/10.1093/nar/gky992.

Nötzli, H., Wyss, T., Stoecklin, C., Schmid, M., Treiber, K., and Hodler, J. 2002. ‘The Contour of the Femoral Head-Neck Junction as a Predictor for the Risk of Anterior Impingement’*. The Journal of Bone and Joint Surgery* 84: 556–60. https://doi: 10.1302/0301-620x.84b4.12014.

Ohmori, K., Ishida, Y., Takatsu, T., Inoue, H., and Suzuki, K. 1995. ‘Vertebral Slip in Lumbar Spondylolysis and Spondylolisthesis. Long-Term Follow-Up of 22 Adult Patients’. *Journal of Bone and Joint Surgery* Series B 77: 771–73.

Ombregt, L. 2013. ‘Ageing of the Lumbar Spine’. In *A System of Orthopaedic Medicine*, 3rd ed., 437–446. London: Churchill Livingstone.

Ontell, F., Moore, E., Shepard, J., and Shelton, D. 1997. ‘The Costal Cartilages in Health and Disease’. *Radiographics* 17 (3): 571–577. https://doi: 10.1148/radiographics.17.3.9153697.

Ortner, D, and M Ericksen. 1997. ‘Bone Changes in the Human Skull Probably Resulting from Scurvy in Infancy and Childhood’. *International Journal of Osteoarchaeology* 7 (3): 212–220. https://doi.org/10.1002/(SICI)1099-1212(199705)7:3<212::AID-OA346>3.0.CO;2-5.

Ortner, D. and Putschar, W. 1981. *Identification of Pathological Conditions in Human Skeletal Remains*. Washington: Smithsonian Institution Press.

Ostrowska, M., Gietka, J., Nesteruk, T., Piliszek, A., and Walecki, J. 2012. ‘Shoulder Joint Tuberculosis’. *Polish Journal of Radiology* 77 (4): 55–59. https://doi: 10.12659/pjr.883630

Ottaway, P. and Rogers, N. 2002. *Craft, Industry and Everyday Life: Finds from Medieval York*. Archaeology of York 17/15. York: Council for British Archaeology.

Oudkerk, S., Mohamed Hoesein, F., Mali, W., Öner, F., Verlaan, J., de Jong, P., Kinney, G., et al. 2019. ‘Subjects with Diffuse Idiopathic Skeletal Hyperostosis Have an Increased Burden of Coronary Artery Disease: An Evaluation in the COPDGene Cohort’. A*therosclerosi*s 287(May): 24–29. https://doi: 10.1016/j.atherosclerosis.2019.05.030.

Pálfi, G., Bereczki, Z., Ortner, D., and Dutour, O. 2012. ‘Juvenile Cases of Skeletal Tuberculosis from the Terry Anatomical Collection (Smithsonian Institution, Washington, D.C., USA)’. *Acta Biologica Szegediensis* 56 (1): 1–12.

Panchal-Kildare, S. and Malone, K. 2013. ‘Skeletal Anatomy of the Hand’. *Hand Clinics* 29 (4): 459–471. https://doi: 10.1016/j.hcl.2013.08.001.

Papadakos, P, and M Gestring, eds. 2015. *Encyclopedia of Trauma Care.* Springer. https://doi.org/10.1007/978-3-642-36200-2.

Pfeiffer, S. 1979. ‘The Relationship of Buccal Pits to Caries Formation and Tooth Loss’. *American Journal of Physical Anthropology* 50 (1): 35–37. https://doi.org/10.1002/ajpa.1330500106.

Philippon, M., Ejnisman, L., Pennock, A., Ho, C., Herzog, M., Lertwanich, P., and Briggs, K. 2011. ‘Does Femoral Anteversion Play a Role in the Pathomechanics and Subsequent Surgical Treatment of Femoroacetabular Impingement?’ *Arthroscopy: The Journal of Arthroscopic & Related Surgery* 27 (5): e53. https://doi.org/10.1016/j.arthro.2011.03.048.

Piggott, C. and Friedlander, S. 2012. ‘Dermatophytes and Other Superficial Fungi’. In *Principles and Practice of Pediatric Infectious Diseases*, 4th ed., 1246–1250. London: Elsevier Saunders.

Pillai, S, and G Littlejohn. 2014. ‘Metabolic Factors in Diffuse Idiopathic Skeletal Hyperostosis – A Review of Clinical Data’. *The Open Rheumatology Journal* 8 (1): 116–128. https://doi.org/10.2174/1874312901408010116.

Raggio, B, S Ficenec, T Flowers, C Lawlor, and K Rodriguez. 2018. ‘Osteochondroma of the Hyoid: First Pediatric Case and Literature Review’. *Clinical Pediatrics* 57 (3): 307–310. https://doi.org/10.1177/0009922817722012.

Rana, R, J Wu, and R Eisenberg. 2009. ‘Periosteal Reaction’. *American Journal of Roentgenology* 193 (4): 259–272. https://doi.org/10.2214/AJR.09.3300.

Reber, S, and T Simmons. 2015. ‘Interpreting Injury Mechanisms of Blunt Force Trauma from Butterfly Fracture Formation’. *Journal of Forensic Sciences* 60 (6): 1401–1411. https://doi.org/10.1111/1556-4029.12797.

Reimer, P.J., Bard, E., Bayliss, A., Beck, J., Blackwell, P.G., Bronk Ramsey, C., Grootes, P.M., et al. 2013. ‘IntCal13 and Marine13 Radiocarbon Age Calibration Curves 0–50,000 Years Cal BP’. *Radiocarbon* 55 (4): 1869–1887. https://doi.org/10.2458/azu_js_rc.55.16947.

Rhomberg, W. and Schuster, A. 2014. ‘Premature Calcifications of Costal Cartilages: A New Perspective’. *Radiology Research and Practice* 2014: 1–5. https://doi: 10.1155/2014/523405.

Richard-Scott, G., C Turner II, G Townsend, and M Martinón-Torres. 2018. *The Anthropology of Modern Human Teeth*. 2nd ed. Cambridge: Cambridge University Press. https://doi.org/10.1017/cbo9781316529843.

Riddler, I.D. 2012. ‘The Late Saxon Material Culture’. In *A History of Wharram Percy and Its Neighbours, Wharram. A Study of Settlement on the Yorkshire Wolds XIII*, by Wrathmell, S., 196–203. York University Archaeological Publications 15. York: York University.

Rivera, F, and M Lahr. 2017. ‘New Evidence Suggesting a Dissociated Etiology for Cribra Orbitalia and Porotic Hyperostosis’. *American Journal of Physical Anthropology* 164 (1), 1–21. https://doi.org/10.1002/ajpa.23258.

Robb, J.E., ed. in prep. *After the Plague: Health and History in Medieval Cambridge.* Cambridge: McDonald Institute for Archaeological Research.

Roberge, R, M Morgenstern, and H Osborn. 1984. ‘Cough Fracture of the Ribs’. *American Journal of Emergency Medicine* 2 (6): 513–517. https://doi: 10.1016/0735-6757(84)90077-9.

Roberts, C. 2017. ‘Guidance on Recording Palaeopathology (Abnormal Variation)’. In *Updated Guidelines to the Standards for Recording Human Remains*, edited by P Mitchell and M Brickley, 44–47. Chartered Institute for Archaeologists.

Robertson, D., and A. J. Smith. 2009. ‘The Microbiology of the Acute Dental Abscess’. *Journal of Medical Microbiology* 58 (2): 155–162. https://doi.org/10.1099/jmm.0.003517-0.

Roddy, E, and M Doherty. 2010. ‘Epidemiology of Gout’. *Arthritis Research & Therapy* 12 (6): 1–11. https://doi: 10.1186/ar3199.

Rogers, T. 1999. ‘A Visual Method of Determining the Sex of Skeletal Remains Using the Distal Humerus’. *Journal of Forensic Sciences* 44 (1): 57–60. https://doi.org/10.1520/jfs14411j.

Romaní, J. & Romaní, M., 2017. ‘Causes and Cures of Skin Diseases in the Work of Hildegard of Bingen’. *Actas Dermo-Sifiliográficas (English Edition)*, 108(6), 538–543.

Roskell, J.S., Clark, L., and Rawcliffe, C., eds. 1993. *The House of Commons, 1386–1421.* Stroud: Alan Sutton for the History of Parliament Trust.

Roth, F. 1966. *The English Austin Friars 1249–1538*. New York: Augustinian Historical Institute.

Rothschild, B. 1997. ‘Porosity: A Curiosity Without Diagnostic Significance’. *American Journal of Physical Anthropology* 104 (4): 529–533. https://doi: 10.1002/(SICI)1096-8644(199712)104:4<529::AID-AJPA7>3.0.CO;2-M.

Rowbotham, S, S Blau, and J Hislop-Jambrich. 2017. ‘Recording Skeletal Completeness: A Standardised Approach’. *Forensic Science International* 275: 117–123. https://doi.org/10.1016/j.forsciint.2017.02.036.

Sagoo, R., Lakdawala, A., and Subbu, R. 2011. ‘Tuberculosis of the Elbow Joint’. *Journal of the Royal Society of Medicine: Short Reports* 2 (3): 17. https://doi: 10.1258/shorts.2011.010130.

Sajid, S, A Yousaf, U Nabi, A Shahbaz, and U Amin. 2019. ‘Sarcomatous Transformation of Recurrent Scapular Osteochondroma in a Patient with the Hereditary Multiple Osteochondromas: A Case Report and Literature Review’. *Cureus* 11 (12): e6308. https://doi.org/10.7759/cureus.6308.

Sakamoto, A. and Matsuda, S. 2017. ‘Pes Anserinus Syndrome Caused by Osteochondroma in Paediatrics: A Case Series Study’. *The Open Orthopaedics Journal* 11 (1): 397–403. https://doi: 10.2174/1874325001711010397.

Salvatore, M., Henschke, C., Yip, R., Kaur, S., Li, K., Padilla, M., and Yankelevitz, D. 2017. ‘Osteophyte Induced Lung Fibrosis Prevalence and Osteophyte Qualities Predicting Disease’. *Clinical Imaging* 44: 1–4. https://doi: 10.1016/j.clinimag.2017.02.008.

Sanchez-Lara, P, J Graham Jr, A Hing, J Lee, and M Cunningham. 2007. ‘The Morphogenesis of Wormian Bones: A Study of Craniosynostosis and Purposeful Cranial Deformation’. American Journal of Medical Genetics Part A 143A: 3243–3251. https://doi.org/10.1002/ajmg.a.

Sangari, S., Dossous, P., Heineman, T., and Mtui, E. 2015. ‘Dimensions and Anatomical Variants of the Foramen Transversarium of Typical Cervical Vertebrae’. *Anatomy Research International* 2015: 1–5. . https://doi: 10.1155/2015/391823.

Sardi, Juan P., Joe Iwanaga, Cameron Schmidt, Tarush Rustagi, Jens R. Chapman, Rod J. Oskouian, and R. Shane Tubbs. 2017. ‘Anatomy of the Alar Ligament: Part II: Variations of Its Attachment onto the Dens’. *World Neurosurgery* 107: 1007–1011. https://doi.org/10.1016/j.wneu.2017.07.186.

Sauder, D, and G Athwal. 2007. ‘Management of Isolated Ulnar Shaft Fractures’. *Hand Clinics* 23 (2): 179–184. https://doi.org/10.1016/j.hcl.2007.01.004.

Saukko, P, and B Knight. 2016. *Knight’s Forensic Pathology*. 4th ed. Boca Raton: CRC Press.

Schattner, A., Dubin, I., and Gelber, M. 2016. ‘A New Diagnostic Clue to Osteomyelitis in Chronic Leg Ulcers’. *American Journal of Medicine* 129 (5): 538–539. https://doi: 10.1016/j.amjmed.2015.10.020.

Schepartz, L. 2010. ‘Bioarchaeology of Apollonia: Tumuli 9, 10, 11 and Appendixes 1, 2, and 3’. In *The Complex of Tumuli 9, 10 and 11 in the Necropolis of Apollonia (Albania) Volume I*, edited by M Grazia Amore, 48–77. Oxford: British Archaeological Reports International Series 2059. https://doi.org/10.30861/9781407305509.

Schutkowski, H. 1993. ‘Sex Determination of Infant and Juvenile Skeletons: I. Morphognostic Features’. *American Journal of Physical Anthropology* 90 (2): 199–205. https://doi.org/10.1002/ajpa.1330900206.

Schweizer, A. 2001. ‘Biomechanical Properties of the Crimp Grip Position in Rock Climbers’. *Journal of Biomechanics* 34 (2): 217–223. https://doi: 10.1016/s0021-9290(00)00184-6.

Sekharappa, V., Amritanand, R., Krishnan, V., and David, K. 2014. ‘Lumbosacral Transition Vertebra: Prevalence and Its Significance’. *Asian Spine Journal* 8 (1): 51–58. https://doi: 10.4184/asj.2014.8.1.51.

Selwitz, R, A Ismail, and N Pitts. 2007. ‘Dental Caries’. *The Lancet* 369: 51–59. https://doi: 10.1016/S0140-6736(07)60031-2.

Shahar, D. and Sayers, M. 2016. ‘A Morphological Adaptation? The Prevalence of Enlarged External Occipital Protuberance in Young Adults’. *Journal of Anatomy* 229 (2): 286–291. https://doi: 10.1111/joa.12466.

Sharma, R, and S Sharma. 2020. ‘Physiology, Blood Volume’. StatPearls [Internet]. https://www.ncbi.nlm.nih.gov/books/NBK526077/

Singh, A., Anand, C., and Singh, S. 2019. ‘A Study of Anatomical Variations in Transverse Foramen of Cervical Vertebrae for Morphological and Clinical Importance’. *International Journal of Contemporary Medical Research* 6 (6): 9–11. https://DOI:10.21276/ijcmr.2019.6.6.19

Singh, J. and Pathak, R. 2013. ‘Sex and Age Related Non-Metric Variation of the Human Sternum in a Northwest Indian Postmortem Sample: A Pilot Study’. *Forensic Science International* 228 (1–3): 181.e1-181.e12. https://doi: 10.1016/j.forsciint.2013.02.002.

Siqueira, José F., and Isabela N. Rôças. 2013. ‘Microbiology and Treatment of Acute Apical Abscesses’. *Clinical Microbiology Reviews* 26 (2): 255–273. https://doi.org/10.1128/CMR.00082-12.

Skubic, J, B Okafor, and D Nehra. 2018. ‘Pathophysiology of Rib Fractures and Lung Contusion’. In *Rib Fracture Management*. A Practical Manual, edited by M de Moya and J Mayberry. Elsevier.

Slonimsky, E., Leibushor, N., Aharoni, D., Lidar, M., and Eshed, I. 2016. ‘Pelvic Enthesopathy on CT Is Significantly More Prevalent in Patients with Diffuse Idiopathic Skeletal Hyperostosis (DISH) Compared with Matched Control Patients’. *Clinical Rheumatology* 35 (7): 1823–1827. https://doi: 10.1007/s10067-015-3151-3.

Smith, D., Nania, A., Hirani, N., Ritchie, G., Jones, M., and Murchison, J. 2017. ‘Osteophyte Related Fibrosis: An Under-Appreciated but Common Finding on High-Resolution Computed Tomography (HRCT)’. *Clinical Radiology* 72: S3. https://doi.org/10.1016/j.crad.2017.06.048.

Smith, H., Anderson, D., Vaccaro, A., Albert, T., Hilibrand, A., Harrop, J., and Ratliff, J. 2010. ‘Anatomy, Biomechanics, and Classification of Thoracolumbar Injuries’. *Seminars in Spine Surgery* 22 (1): 2–7. https://doi.org/10.1053/j.semss.2009.10.001.

Smith, J. and Varacallo, M. 2018. ‘Osgood Schlatter Disease’. StatPearls [Internet]. https://www.ncbi.nlm.nih.gov/books/NBK441995/

Souza, A de, and R Bispo Júnior. 2014. ‘Osteochondroma: Ignore or Investigate?’ *Revista Brasileira de Ortopedia* 49 (6): 555–564. https://doi.org/10.1016/j.rboe.2013.10.002.

Spekker, O., Hunt, D., Paja, L., Molnár, E., Pálfi, G., and Schultz, M. 2020. ‘Tracking Down the White Plague: The Skeletal Evidence of Tuberculous Meningitis in the Robert J. Terry Anatomical Skeletal Collection’. *PLoS ONE* 15 (3): 1–17. https://doi.org/10.1371/journal.pone.0230418.

Spekker, O., Pálfi, G., Kozocsay, G., Pósa, A., Bereczki, Z., and Molnár, E. 2012. ‘New Cases of Probable Skeletal Tuberculosis from the Neolithic Period in Hungary: A Morphological Study’. *Acta Biologica Szegediensis* 56 (2): 115–123.

Standring, S., Anand, N., Birch, R., Collins, P., Crossman, A., Gleeson, M., Jawaheer, G., et al. 2016. *Gray’s Anatomy. The Anatomical Basis of Clinical Practice*. 41st ed. Amsterdam: Elsevier.

Stratford, N. 1987. ‘Gothic Ivory Carving in England’. In *Age of Chivalry. Art in Plantagenet England 1200–1400*, edited by Alexander, J. and Binski, P., 107–13. London.

Streubel, P, and R Pesántez. 2015. ‘Diaphyseal Fractures of the Radius and Ulna’. In *Rockwood and Green’s Fractures in Adults*, edited by C Court-Brown, J Heckman, M McQueen, W Ricci, P Tornetta, and M McKee, 8th ed., 1121–1177. London: Wolters Kluwer.

Sudoł-Szopińska, I, B Kwiatkowska, M Prochorec-Sobieszek, and W Maśliński. 2015. ‘Enthesopathies and Enthesitis. Part 1. Etiopathogenesis’. *Journal of Ultrasonography* 15 (60): 72–84. https://doi.org/10.15557/jou.2015.0006.

Szabo, R, and M Skinner. 1990. ‘Isolated Ulnar Shaft Fractures: Retrospective Study of 46 Cases’. *Acta Orthopaedica* 61 (4): 350–352. https://doi.org/10.3109/17453679008993534.

Taitz, C., Nathan, H., and Arensburg, B. 1978. ‘Anatomical Observations of the Foramina Transversaria’. *Journal of Neurology, Neurosurgery, and Psychiatry* 41 (2): 170–176. https://doi: 10.1136/jnnp.41.2.170.

Takatalo, J.., Karppinen, J., Niinimäki, J., Taimela, S., Näyhä, S., Järvelin, M., Kyllönen, E., and Tervonen, O. 2009. ‘Prevalence of Degenerative Imaging Findings in Lumbar Magnetic Resonance Imaging Among Young Adults’. *Spine* 34 (16): 1716–1721. https://doi: 10.1097/BRS.0b013e3181ac5fec.

Takatalo, J., Karppinen, J., Taimela, S., Niinimäki, J., Laitinen, J., Blanco Sequeiros, R., Paananen, M., et al. 2013. ‘Body Mass Index Is Associated with Lumbar Disc Degeneration in Young Finnish Males: Subsample of Northern Finland Birth Cohort Study 1986’. *BMC Musculoskeletal Disorder*s 14 (87): 1–10. https://doi: 10.1186/1471-2474-14-87.

Taniegra, E. 2004. ‘Hyperparathyroidism’. *American Family Physician* 69 (2): 333–339.

Tavakoli Darestani, R., Sharifzadeh, A., Lemraski, M., and Zanganeh, R. 2013. ‘A Rare Case of Gorham’s Disease: Primary Ulnar Involvement with Secondary Spread to the Radius and Elbow’. *Trauma Monthly* 18 (1): 41–45. https://doi: 10.5812/traumamon.9905

Tavares, E., Dores, J., Ferreira, L., Martinho, G., and Vera-Cruz, F. 2011. ‘Marjolin’s Ulcer Associated with Ulceration and Chronic Osteomyelitis’. *Anais Brasileiros de Dermatologia* 86 (2): 366–369. https://doi: 10.1590/s0365-05962011000200026.

Thiele-Nygaard, A, J Foss-Skiftesvik, and M Juhler. 2020. ‘Intracranial Pressure, Brain Morphology and Cognitive Outcome in Children with Sagittal Craniosynostosis’. *Child’s Nervous System* 36 (4): 689–695. https://doi.org/10.1007/s00381-020-04502-z.

Thottam, Gabrielle E., Svetlana Krasnokutsky, and Michael H. Pillinger. 2017. ‘Gout and Metabolic Syndrome: A Tangled Web’. *Current Rheumatology Reports* 19 (10): 1–8. https://doi.org/10.1007/s11926-017-0688-y.

Tiemann, A. and Hofmann, G. 2009. ‘Principles of the Therapy of Bone Infections in Adult Extremities: Are There Any New Developments?’. *Strategies in Trauma and Limb Reconstruction* 4 (2): 57–64. https://doi: 10.1007/s11751-009-0059-y.

Timmerman, M, and G Van der Weijden. 2006. ‘Risk Factors for Periodontitis’. *International Journal of Dental Hygiene* 4 (1): 2–7. https://doi.org/10.1016/j.immuni.2010.12.017.

Tiwari, V, V Sampath Kumar, R Poudel, A Kumar, and S Khan. 2017. ‘Pes Anserinus Bursitis Due to Tibial Spurs in Children’. *Cureus* 9 (7): 5–10. https://doi: 10.7759/cureus.1427

Tortora, G, and B Derrickson. 2014. *Principles of Anatomy & Physiology*. 14th ed. John Wiley & Sons Ltd.

Trotter, M. 1970. ‘Estimation of Stature from Intact Long Limb Bones’. In *Personal Identification in Mass Disasters. Report of a Seminar Held in Washington, D.C., 9-11 December 1968, by Arrangement between the Support Services of the Department of the Army and the Smithsonian Institution*., edited by T Stewart, 71–83. Washington: National Museum of Natural History.

Tubbs, R., Hallock, J., Radcliff, V., Naftel, R., Mortazavi, M., Shoja, M., Loukas, M., and Cohen-Gadol, A. 2011. ‘Ligaments of the Craniocervical Junction’. *Journal of Neurosurgery: Spine* 14 (6): 697–709. <https://doi>: 10.3171/2011.1.SPINE10612.

Tuli, S. 2016. *Tuberculosis of the Skeletal System*. 5th ed. London: Jayppe Brothers Medical Pubishers.

Turkay, R, E Inci, S Ors, M Nalbant, and I Gurses. 2017. ‘Frequency of Sternal Variations in Living Individuals’. *Surgical and Radiologic Anatomy* 39 (11): 1273–78. https://doi.org/10.1007/s00276-017-1854-7.

Türker, F. 2019. ‘Hemorrhagic Shock [Online First]’. In *Clinical Management of Shock - The Science and Art of Physiological Restoration*. IntechOpen. https://doi.org/0.5772/intechopen.82358.

Tzikas, S., Triantafyllou, K., Papadopoulos, C., and Vassilikos, V. 2016. ‘A Case of a Paracardial Osteophyte Causing Atrial Compression’. *Case Reports in Medicine* 2016: 3–5. https://doi: 10.1155/2016/4325830.

Valencia, I., Falabella, A., Kirsner, R., and Eaglstein, W. 2001. ‘Chronic Venous Insufficiency and Venous Leg Ulceration’. *Journal of the American Academy of Dermatology* 44 (3): 401–424. https://doi: 10.1067/mjd.2001.111633.

Varunjikar, M., Jayan, B., and Gadre, N. 2014. ‘A Rare Presentation of Tuberculosis Affecting Both Bones of Forearm’. *International Journal of Medical and Applied Sciences* 3 (1): 195–201.

Venn, J. and Venn, J.A. 1922a*. Alumni Cantabrigienses Part I from the Earliest Times to, Vol. I. Abbas–Cutts.* Cambridge: Cambridge University Press.

Venn, J. and Venn, J.A. 1922b*. Alumni Cantabrigienses Part I from the Earliest Times to, Vol. II. Dabbs–Juxton.* Cambridge: Cambridge University Press.

Venn, J. and Venn, J.A. 1924*. Alumni Cantabrigienses Part I from the Earliest Times to, Vol. III. Kaile – Ryves.* Cambridge: Cambridge University Press.

Venn, J. and Venn, J.A. 1927*. Alumni Cantabrigienses Part I from the Earliest Times to, Vol. IV. Saal – Zuinglius.* Cambridge: Cambridge University Press.

Villotte, S. 2013. *Practical Protocol for Scoring the Appearance of Some Fibrocartilaginous Entheses on the Human Skeleton*. https://www.academia.edu/1427191/Practical_protocol_for_scoring_the_appearance_of_some_fibrocartilaginous_entheses_on_the_human_skeleton

Villotte, S, D Castex, V Couallier, O Dutour, C Knüsel, and D Henry-Gambier. 2010. ‘Enthesopathies as Occupational Stress Markers: Evidence from the Upper Limb’. *American Journal of Physical Anthropology* 142 (2): 224–234. https://doi: 10.1002/ajpa.21217.

Waldron, T. 2021. *Palaeopathology*. 2nd ed. Cambridge: Cambridge University Press.

Walker, D, N Powers, B Connell, and R Redfern. 2015. ‘Evidence of Skeletal Treponematosis from the Medieval Burial Ground of St. Mary Spital, London, and Implications for the Origins of the Disease in Europe’. *American Journal of Physical Anthropology* 156 (1): 90–101. https://doi.org/10.1002/ajpa.22630.

Walker, P, R Bathurst, R Richman, T Gjerdrum, and V Andrushko. 2009. ‘The Causes of Porotic Hyperostosis and Cribra Orbitalia: A Reappraisal of the Iron-Deficiency-Anemia Hypothesis’. *American Journal of Physical Anthropology* 139 (2): 109–25. https://doi: 10.1002/ajpa.21031.

Waterman, D.M. 1959. ‘Late Saxon, Viking and Early Medieval Finds from York’. *Archaeologia* 97: 59–105.

Weaver, P. and Lifeso, R. 1984. ‘The Radiological Diagnosis of Tuberculosis of the Adult Spine’. *Skeletal Radiology* 12 (3): 178–186. https://doi: 10.1007/BF00361084.

Webb, E. 1921. *The Records of St. Bartholomew’s Priory and St. Bartholomew the Great, West Smithfield: Volume 1*. Oxford: Oxford University Press.

Weissman, B. 2009. *Imaging of Arthritis and Metabolic Bone Disease*. Philadelphia: Mosby Elsevier.

Wells, Clavin. 1974. ‘Osteochondritis Dissecans in Ancient British Skeletal Material’. *Medical History* 18 (4): 365–369. https://doi.org/10.1017/S0025727300019815.

Wertheimer, A, A Olaussen, S Perera, S Liew, and B Mitra. 2018. ‘Fractures of the Femur and Blood Transfusions’. *Injury* 49 (4): 846–851. https://doi.org/10.1016/j.injury.2018.03.007.

Weston, Darlene A. 2008. ‘Investigating the Specificity of Periosteal Reactions in Pathology Museum Specimens’. *American Journal of Physical Anthropology* 137 (1): 48–59. https://doi.org/10.1002/ajpa.20839.

Williams, F, N Manek, P Sambrook, T Spector, and A MacGregor. 2007. ‘Schmorl’s Nodes: Common, Highly Heritable, and Related to Lumbar Disc Disease’. *Arthritis Care and Research* 57 (5): 855–860. https://doi.org/10.1002/art.22789.

Winburn, A. and Stock, M. 2019. ‘Reconsidering Osteoarthritis as a Skeletal Indicator of Age at Death’. *American Journal of Physical Anthropology* 170 (3): 459–473. https://doi: 10.1002/ajpa.23914.

Winstead, K.A. 2007. *John Capgrave’s Fifteenth Century*. Philadelphia: University of Philadelphia Press.

Wood, J.W., Milner, G.R. Harpending, H.C. Weiss, K.M. Cohen, M.N. Eisenberg, L.E. Hutchinson, D.L. et al. 1992. ‘The Osteological Paradox: Problems of Inferring Prehistoric Health from Skeletal Samples [and Comments and Reply]’. *Current Anthropology* 33 (4): 343–70.

Wright, T. 2001. ‘Interosseous Membrane of the Forearm’. *Journal of the American Society for Surgery of the Hand* 1 (2): 123–134. https://doi.org/10.1053/jssh.2001.23907.

Yakkanti, R, I Onyekwelu, L Carreon, and J Dimar. 2018. ‘Solitary Osteochondroma of the Spine: A Case Series: Review of Solitary Osteochondroma With Myelopathic Symptoms’. *Global Spine Journal* 8 (4): 323–339. https://doi.org/10.1177/2192568217701096.

Yaussy, S, and S Dewitte. 2019. ‘Calculus and Survivorship in Medieval London: The Association Between Dental Disease and a Demographic Measure of General Health’. *American Journal of Physical Anthropology* 168: 552–565. https://doi.org/10.1002/ajpa.23772.

Yekeler, E, M Tunaci, A Tunaci, M Dursun, and G Acunas. 2006. ‘Frequency of Sternal Variations and Anomalies Evaluated by MDCT’. *American Journal of Roentgenology* 186 (4): 956–960. https://doi.org/10.2214/AJR.04.1779.

Yokoyama, K., Kawanishi, M., Yamada, M., Tanaka, H., Ito, Y., Kawabata, S., and Kuroiwa, T. 2016. ‘Spinopelvic Alignment and Sagittal Balance of Asymptomatic Adults With 6 Lumbar Vertebrae’. *European Spine Journal* 25 (11): 3583–3588. https://doi: 10.1007/s00586-015-4284-4.

Yukata, K. 2015. ‘Cystic Lesion Around The Hip Joint’. *World Journal of Orthopedics* 6 (9): 688. https://doi.org/10.5312/wjo.v6.i9.688.

Zampetti, S, V Mariotti, N Radi, and M Belcastro. 2016. ‘Variation of Skeletal Degenerative Joint Disease Features in an Identified Italian Modern Skeletal Collection’. *American Journal of Physical Anthropology* 160 (4): 683–693. https://doi.org/10.1002/ajpa.22998.

Zandi, B. and Hozhabri, H. 2014. ‘Correlation between Femoral Neck Anteversion in Patients with Osteoarthritis of the Hip and Normal Controls’. *Patient Safety & Quality Improvement Journal* 3 (2): 206–210. https://doi.org/10.22038/PSJ.2015.4173.

Zanotti, M, I Melamed, V Diomin, E Walter, L Baraf, M Frenkel, and I Shelef. 2018. ‘A Multidisciplinary Approach for the Treatment of Young Patients With Suprasellar Osteochondroma’. *Child’s Nervous System* 34 (3): 559–563. https://doi.org/10.1007/s00381-017-3619-3.

Zhang, C., Li, L., Forster, B., Kopec, J., Ratzlaff, C., Halai, L., Cibere, J., and Esdaile, J. 2015. ‘Femoroacetabular Impingement and Osteoarthritis of the Hip’. *Canadian Family Physician* 61 (12): 1055–1060.

Zobel, B., Vadalà, G., Del Vescovo, R., Battisti, S., Martina, F., Stellato, L., Leoncini, E., Borthakur, A., and Denaro, V. 2012. ‘T1 ρ Magnetic Resonance Imaging Quantification of Early Lumbar Intervertebral Disc Degeneration in Healthy Young Adults’. *Spine* 37 (14): 1224–1230. https://doi.org/10.1097/BRS.0b013e31824b2450.
