# Supplementary figures and images for: The people of the Cambridge Austin friars
Source: Archaeol J (Lond). 2022 Sep 26;179(2):383–444. doi: 10.1080/00665983.2022.2090675 (PMC9580237; doi:10.1080/00665983.2022.2090675)

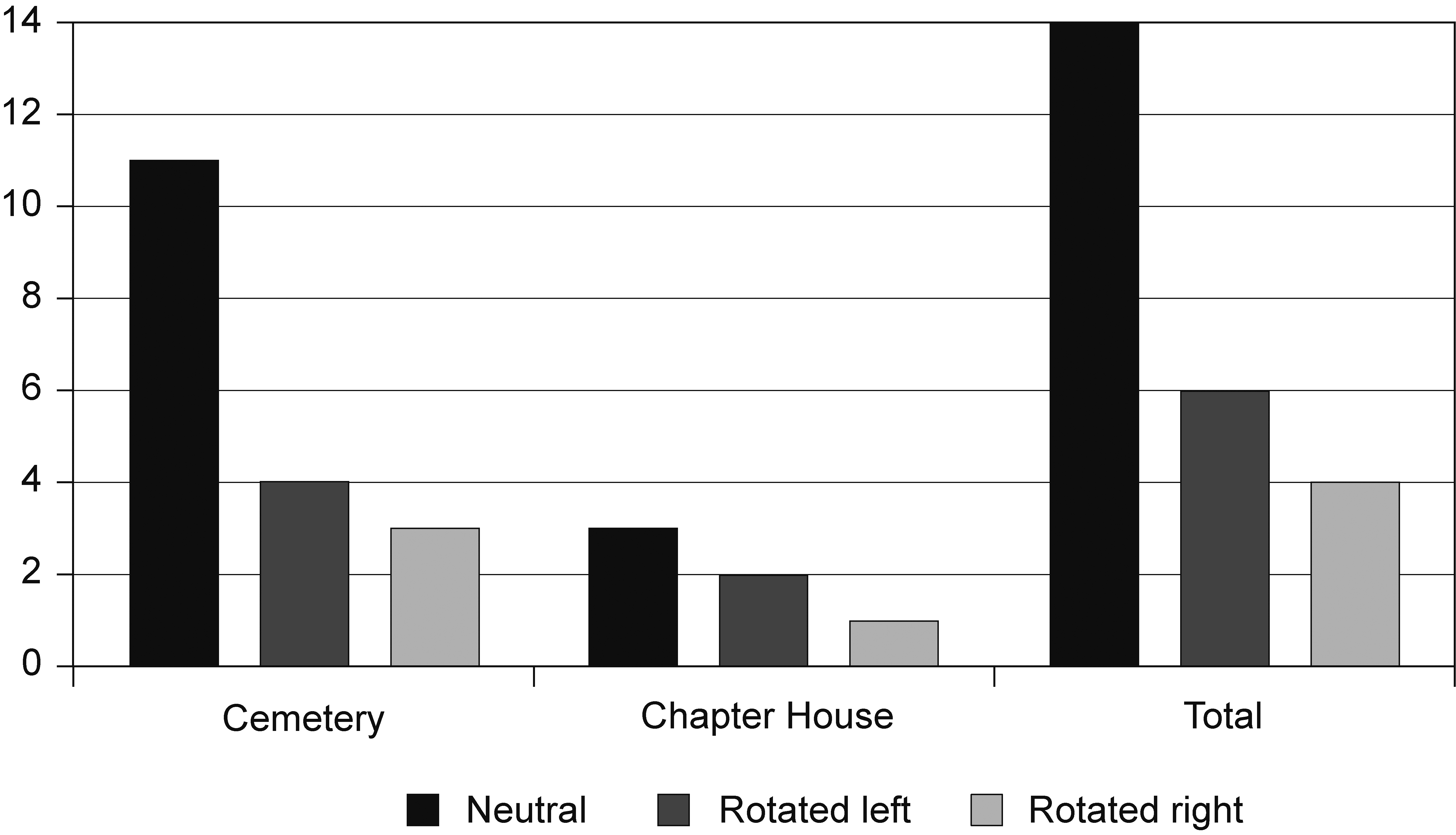

Supplement: Supplemental Material [file RAIJ_A_2090675_SM0285.zip › Supplementary text and figures/Figure_S1 Head positions graph.tif]

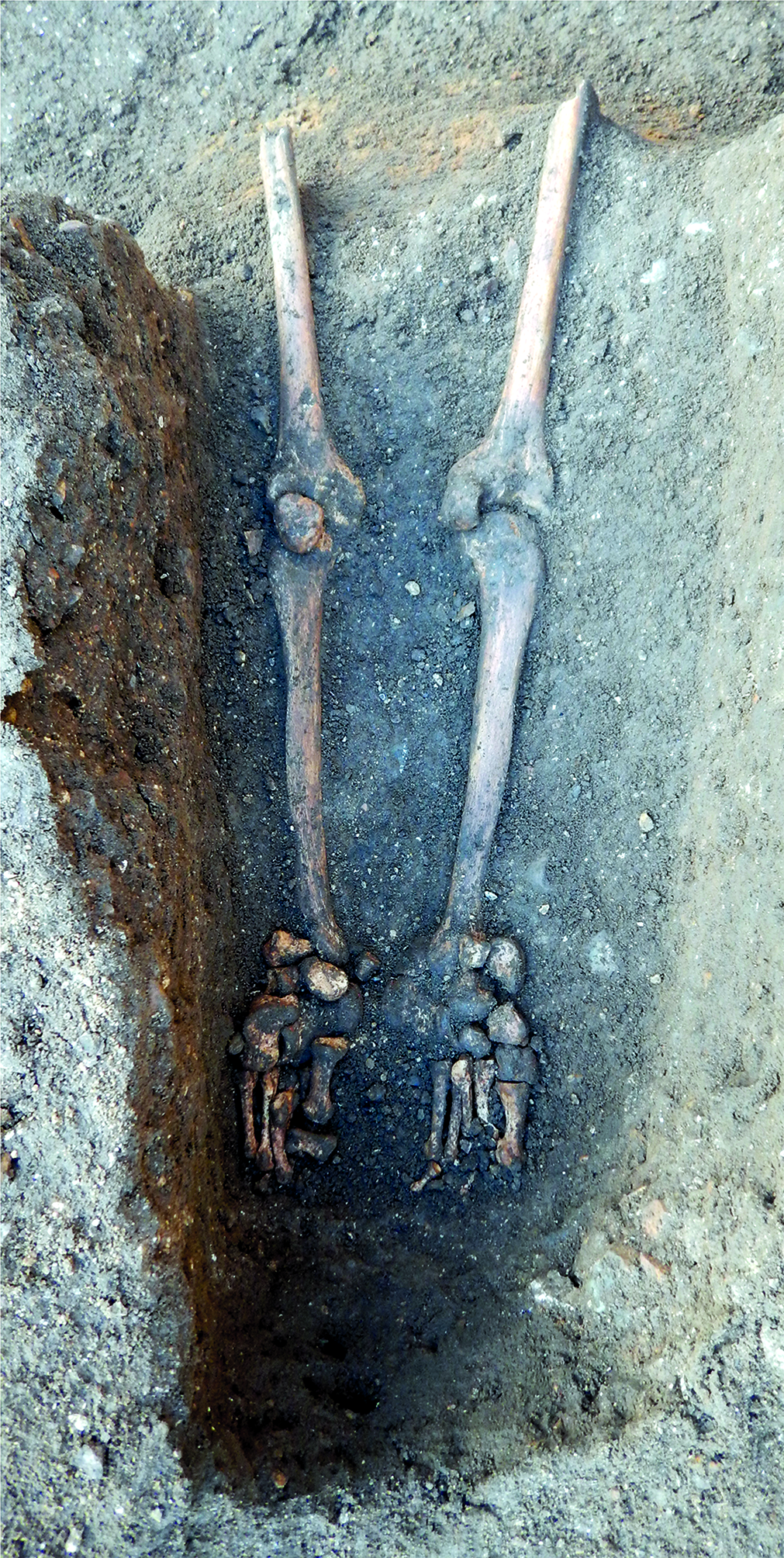

Supplement: Supplemental Material [file RAIJ_A_2090675_SM0285.zip › Supplementary text and figures/Figure_S10 F195.tif]

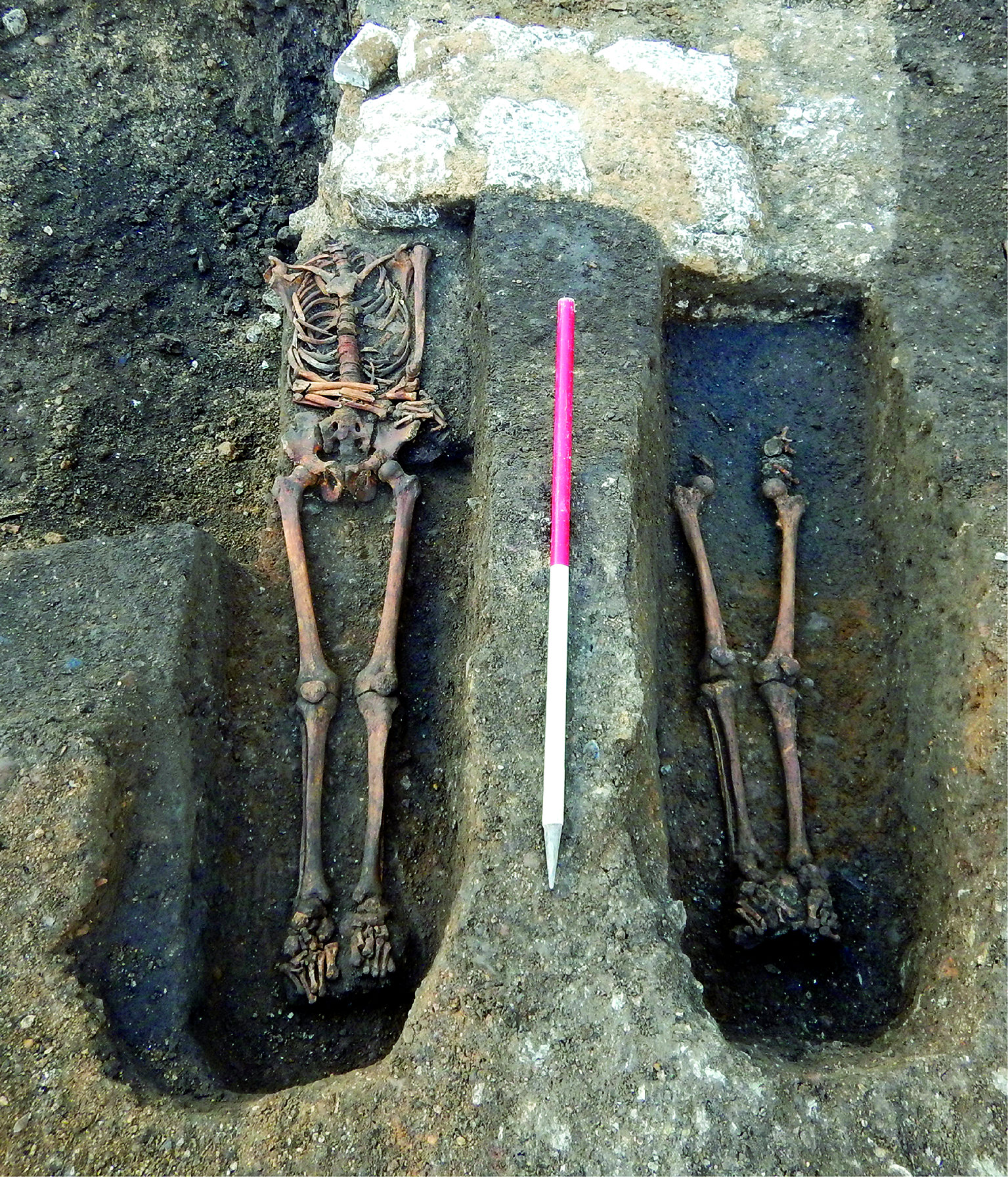

Supplement: Supplemental Material [file RAIJ_A_2090675_SM0285.zip › Supplementary text and figures/Figure_S11 F196 and F198.tif]

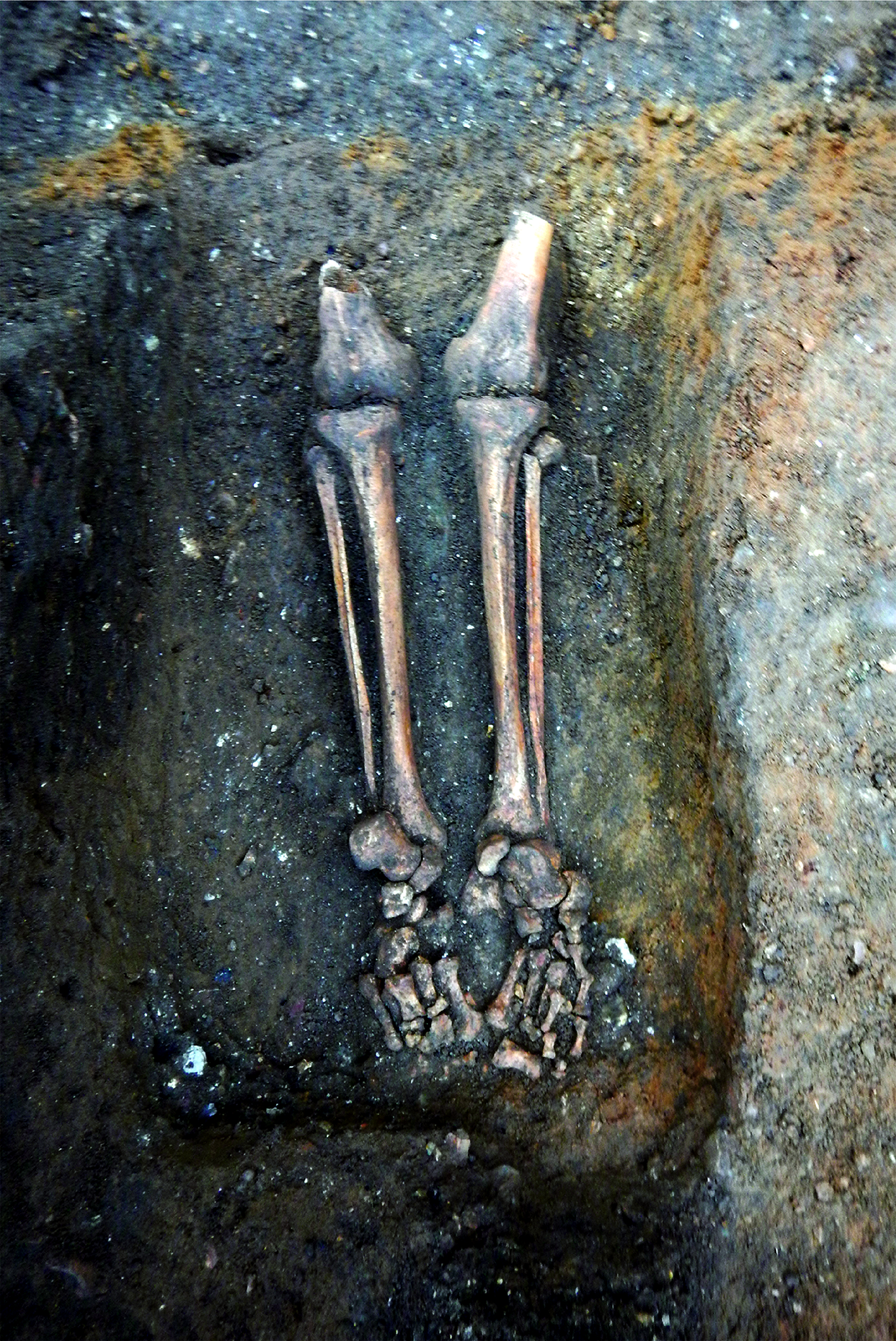

Supplement: Supplemental Material [file RAIJ_A_2090675_SM0285.zip › Supplementary text and figures/Figure_S12 F199.tif]

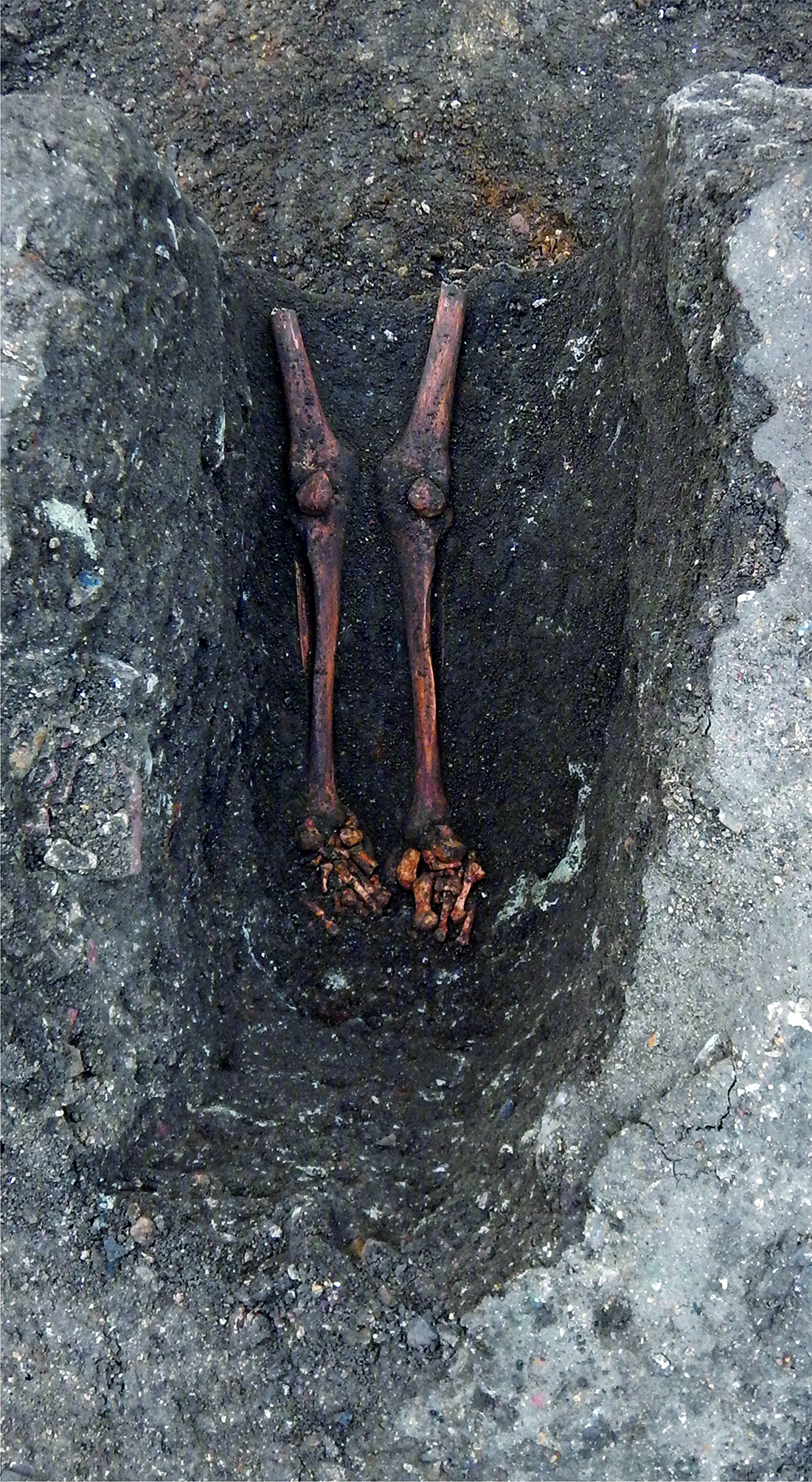

Supplement: Supplemental Material [file RAIJ_A_2090675_SM0285.zip › Supplementary text and figures/Figure_S13 F215.tif]

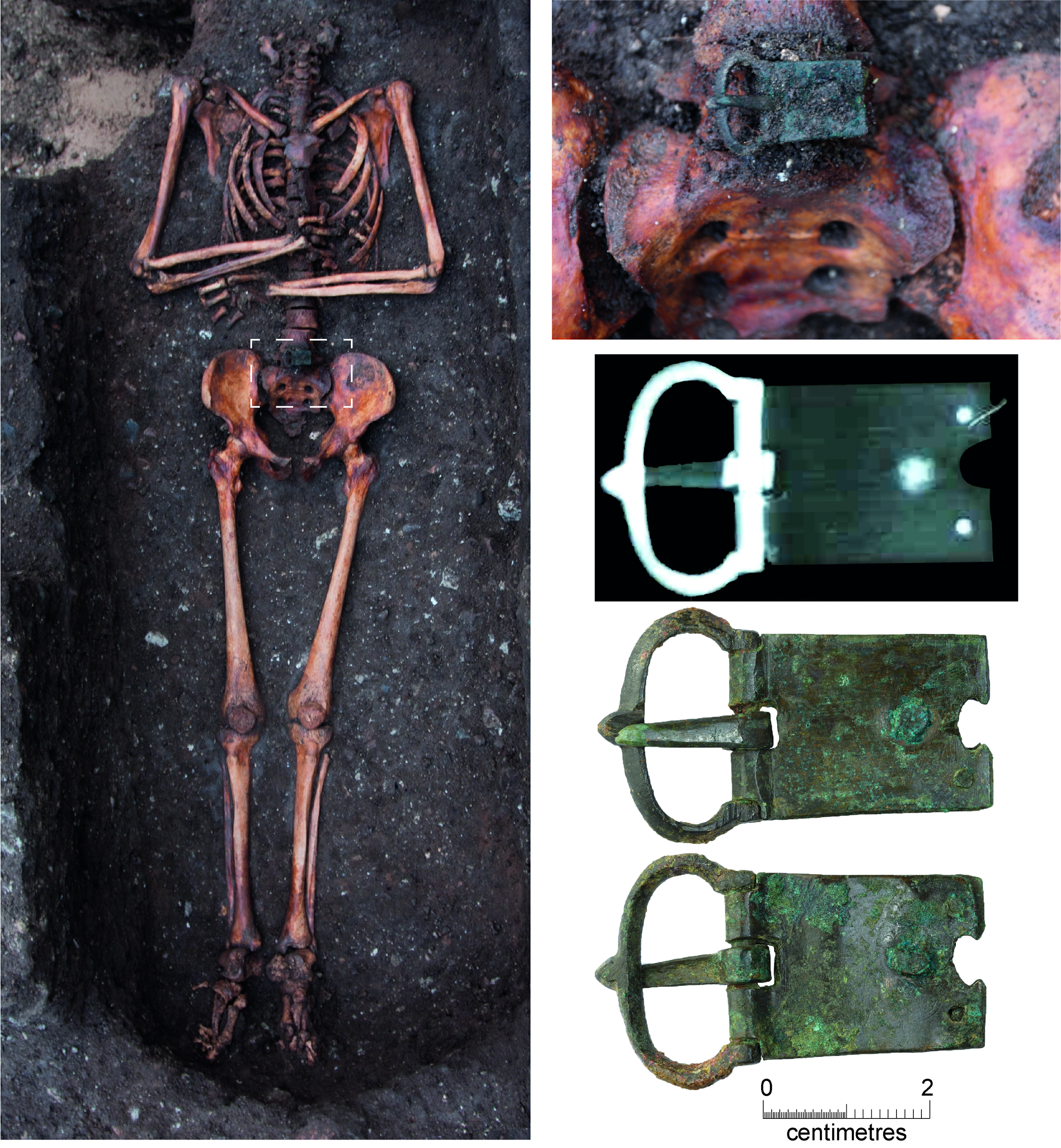

Supplement: Supplemental Material [file RAIJ_A_2090675_SM0285.zip › Supplementary text and figures/Figure_S14 F216.tif]

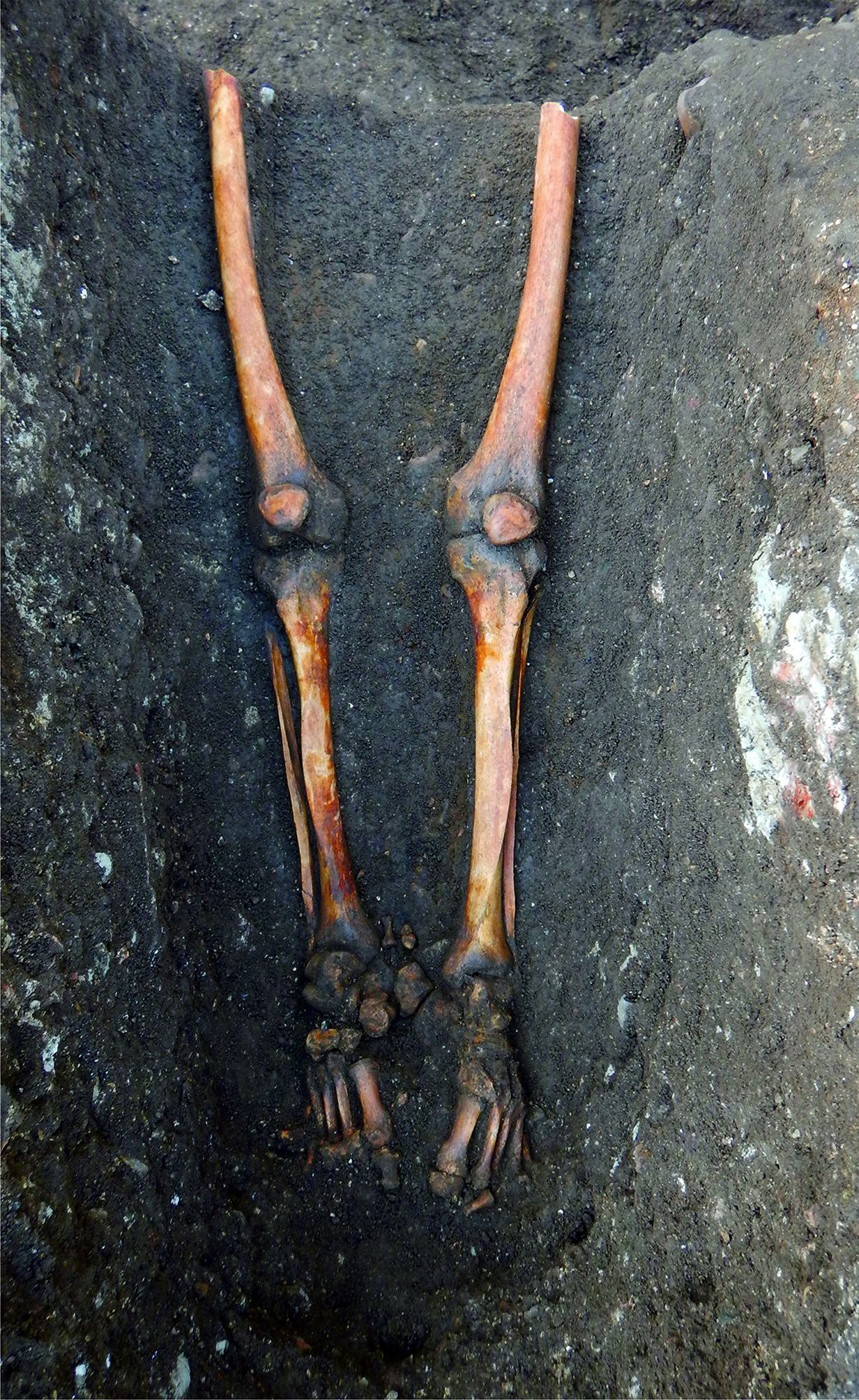

Supplement: Supplemental Material [file RAIJ_A_2090675_SM0285.zip › Supplementary text and figures/Figure_S15 F217.tif]

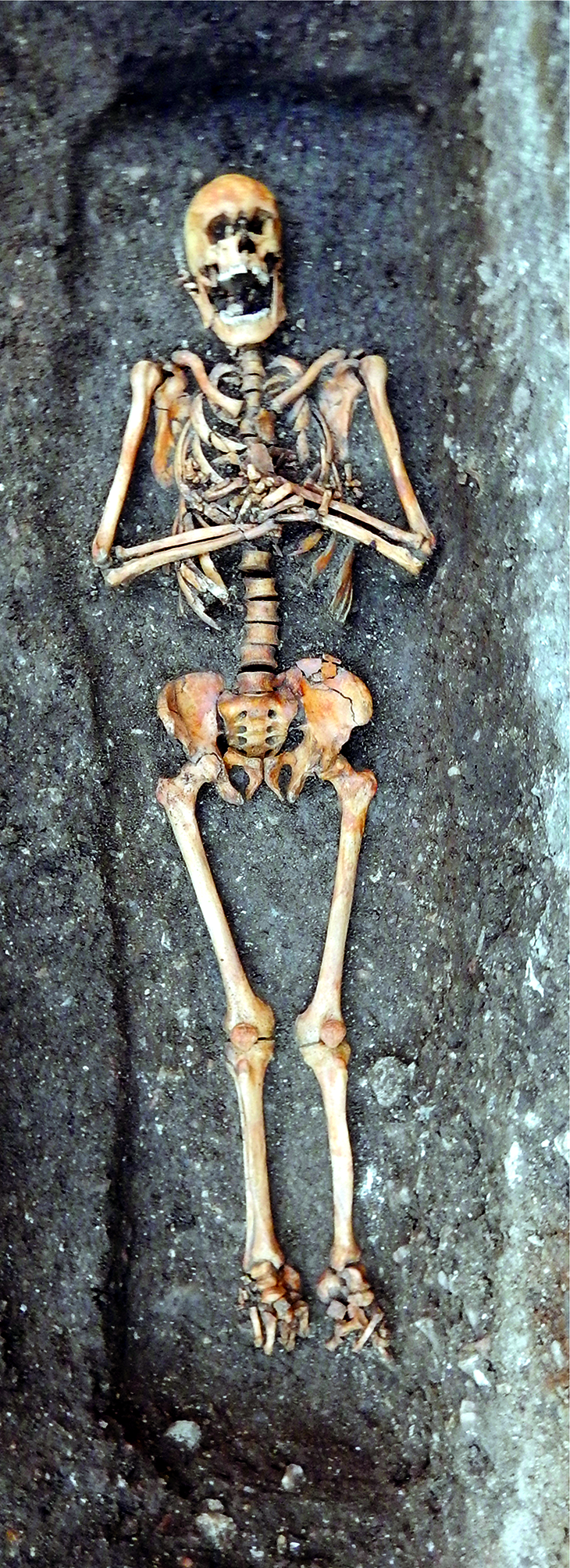

Supplement: Supplemental Material [file RAIJ_A_2090675_SM0285.zip › Supplementary text and figures/Figure_S16 F232.tif]

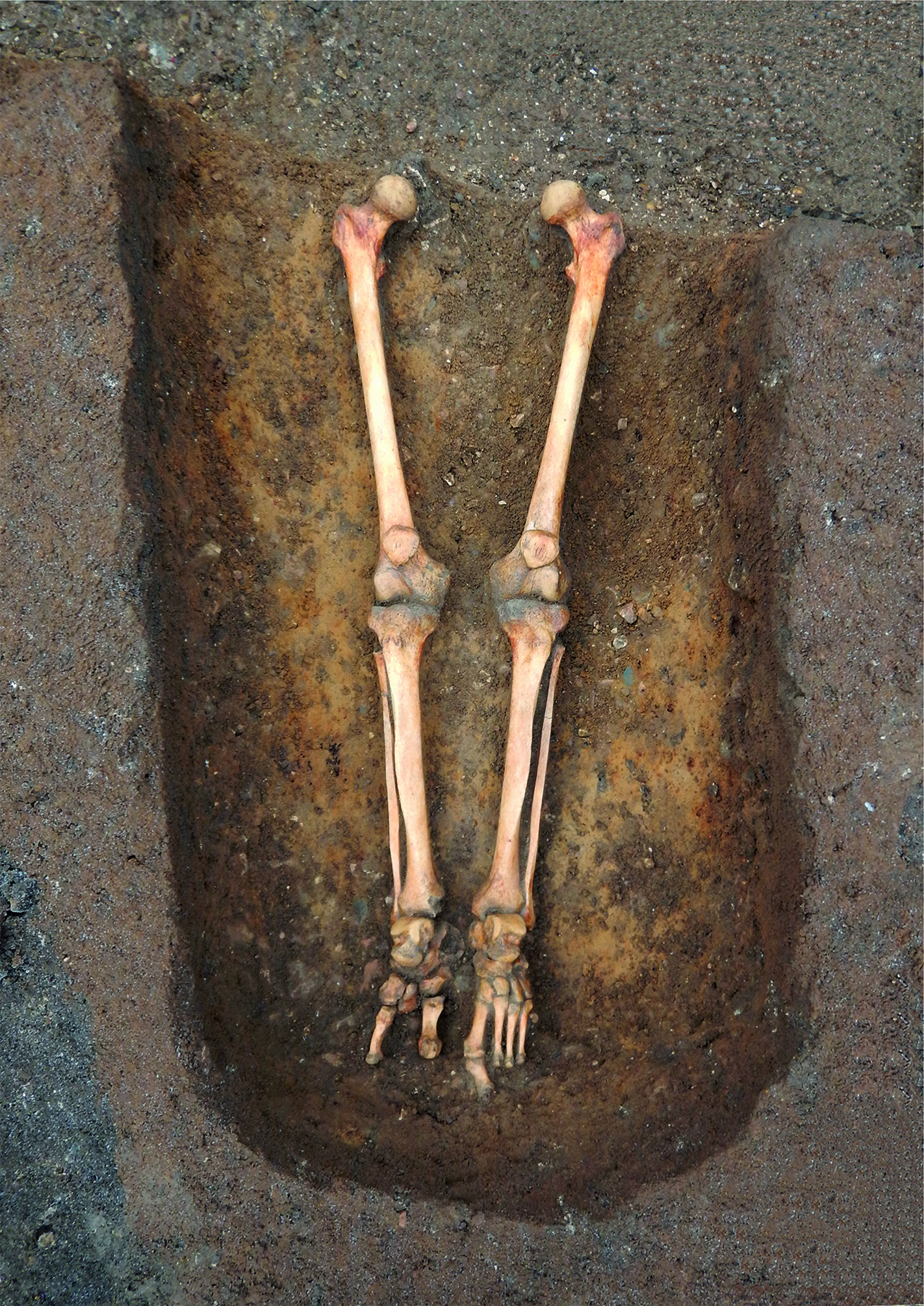

Supplement: Supplemental Material [file RAIJ_A_2090675_SM0285.zip › Supplementary text and figures/Figure_S17 F237.tif]

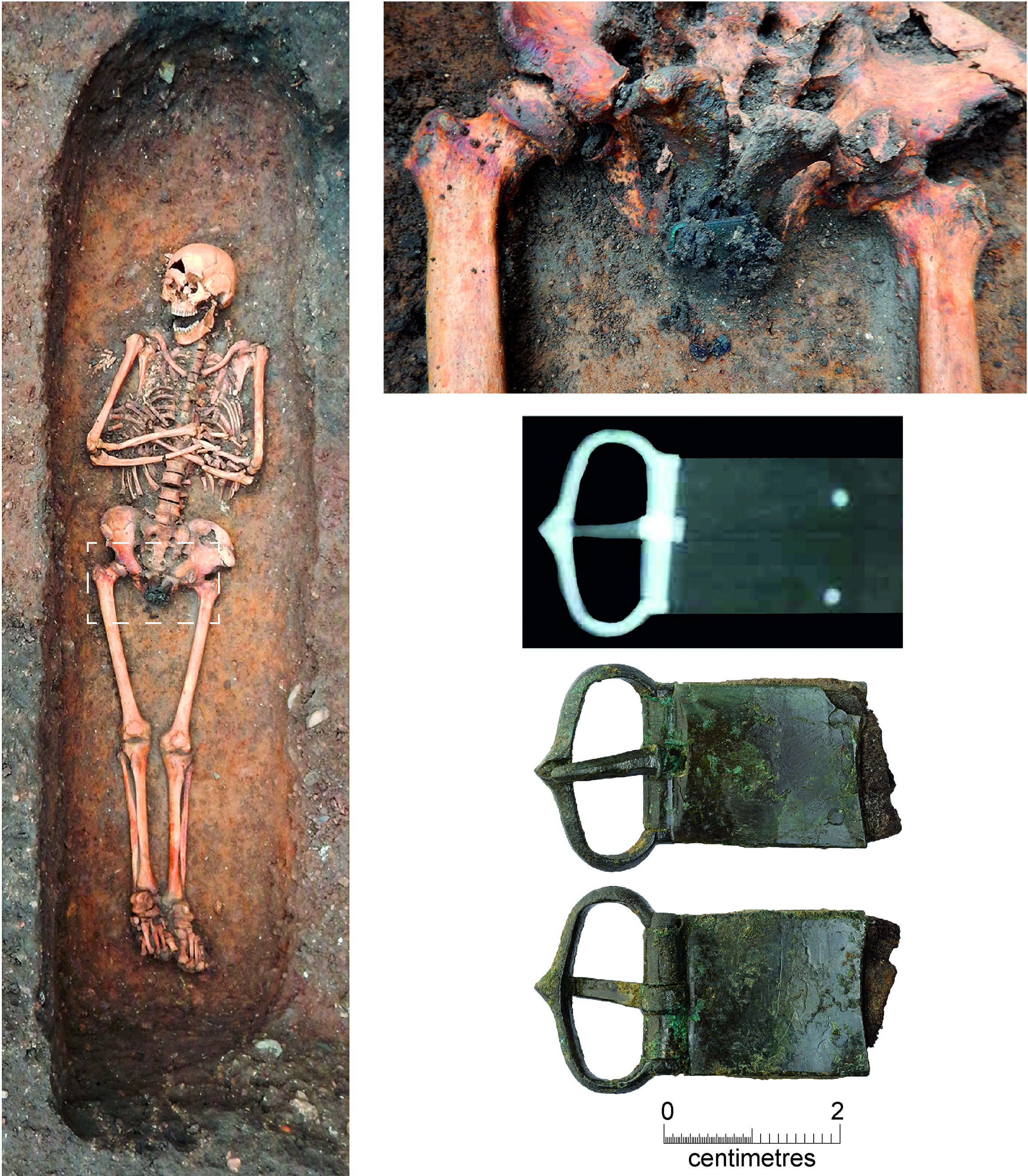

Supplement: Supplemental Material [file RAIJ_A_2090675_SM0285.zip › Supplementary text and figures/Figure_S18 F265.tif]

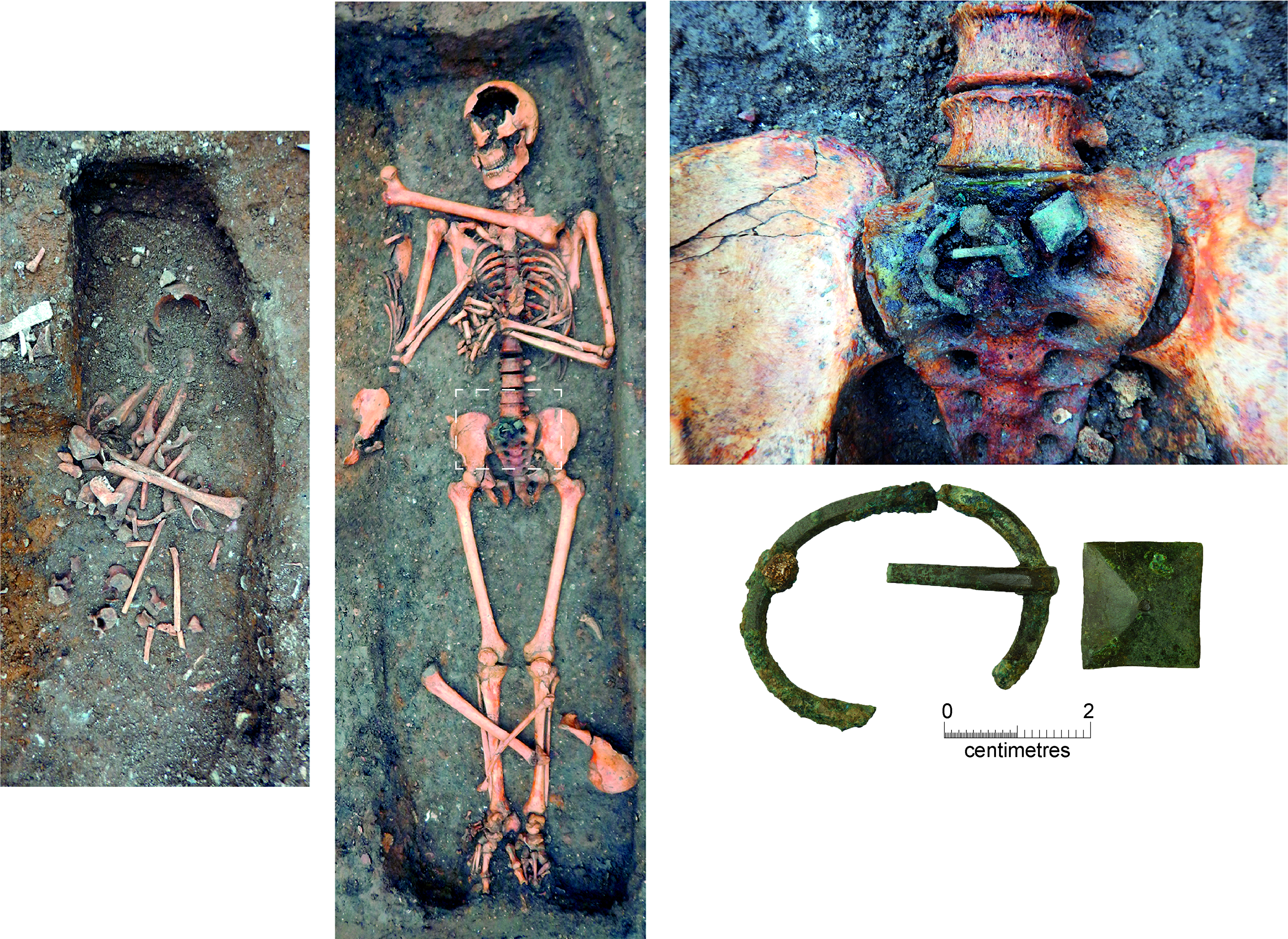

Supplement: Supplemental Material [file RAIJ_A_2090675_SM0285.zip › Supplementary text and figures/Figure_S19 F302.tif]

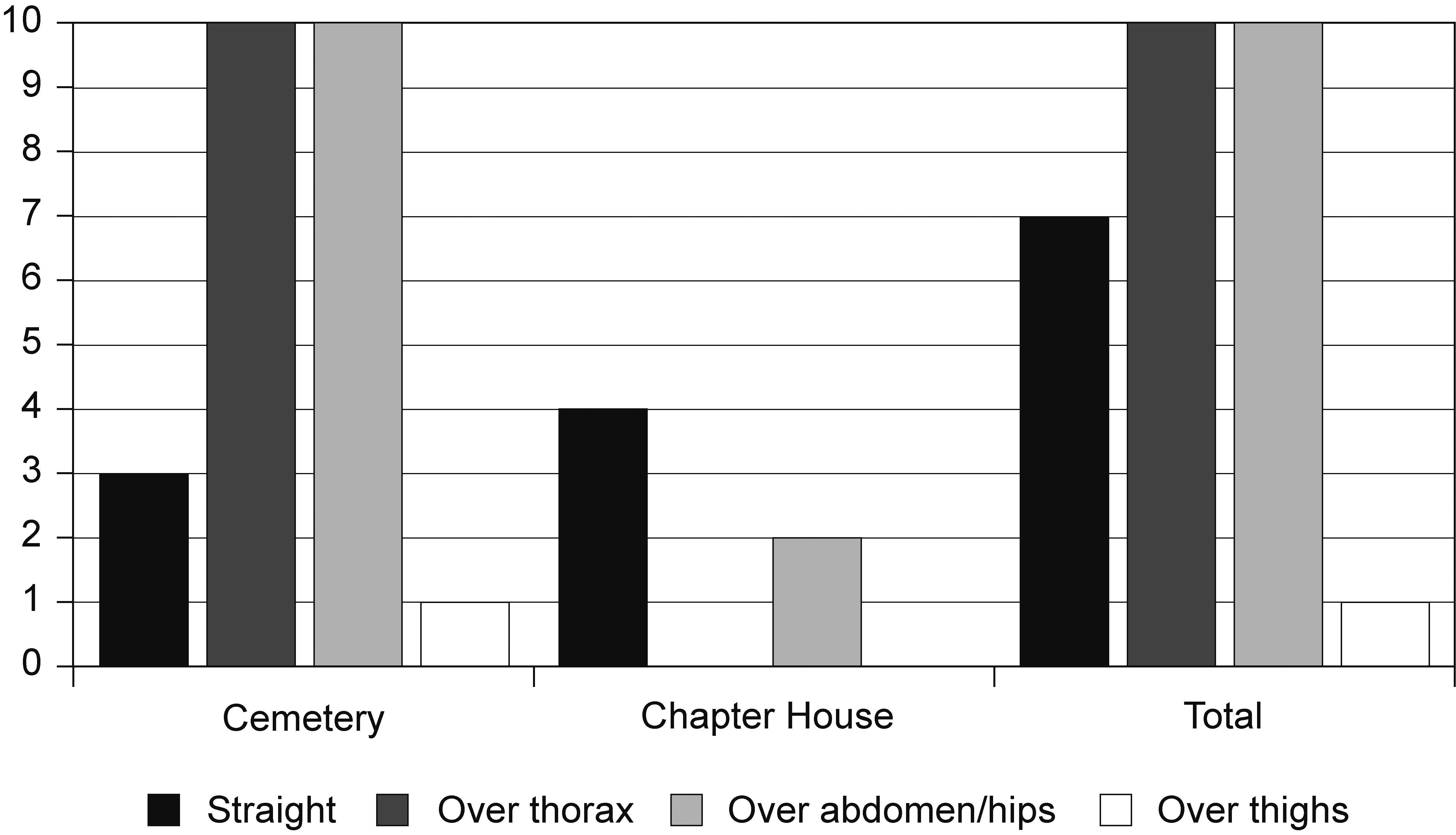

Supplement: Supplemental Material [file RAIJ_A_2090675_SM0285.zip › Supplementary text and figures/Figure_S2 Arm position graph.tif]

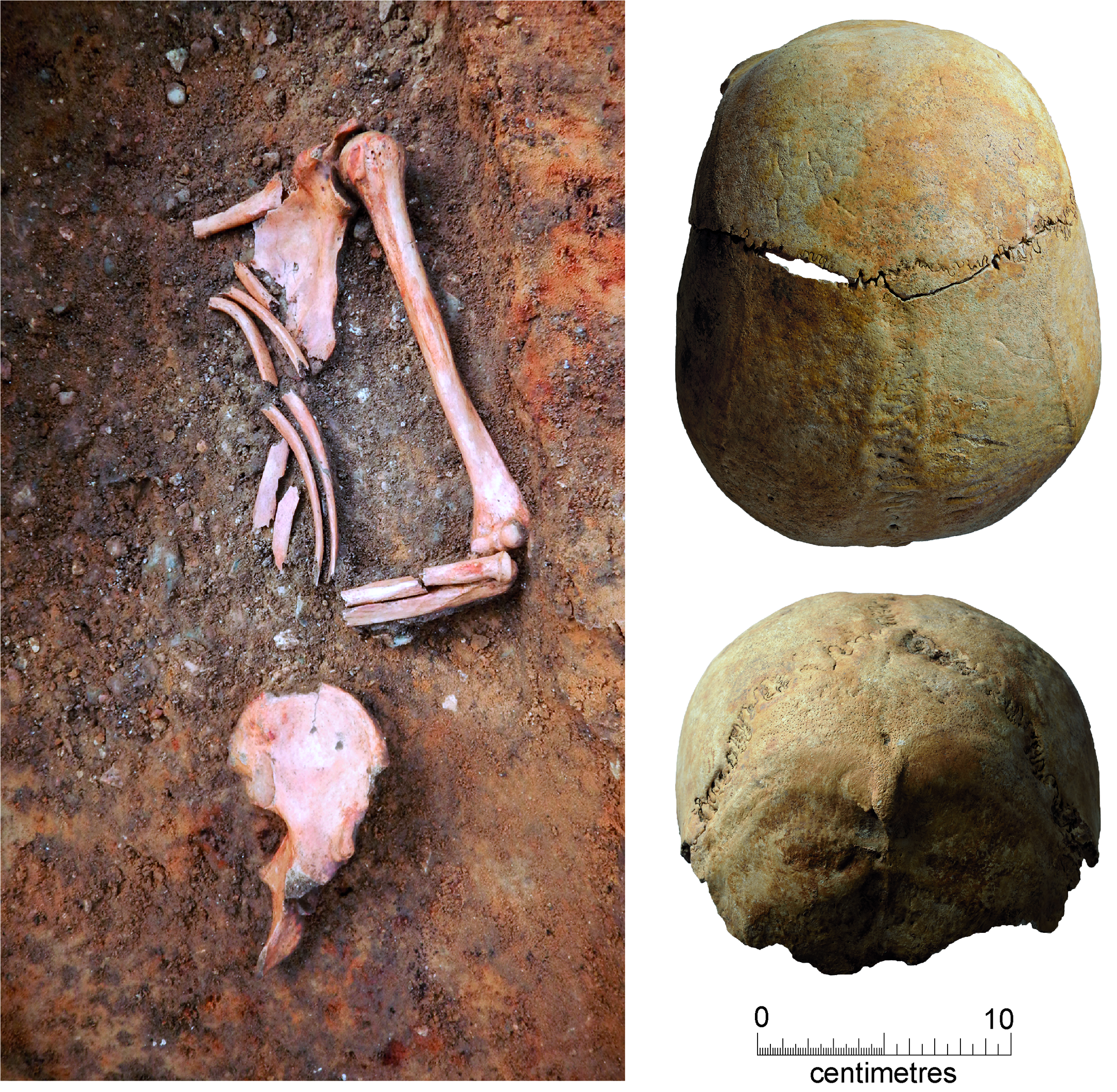

Supplement: Supplemental Material [file RAIJ_A_2090675_SM0285.zip › Supplementary text and figures/Figure_S20 F309.tif]

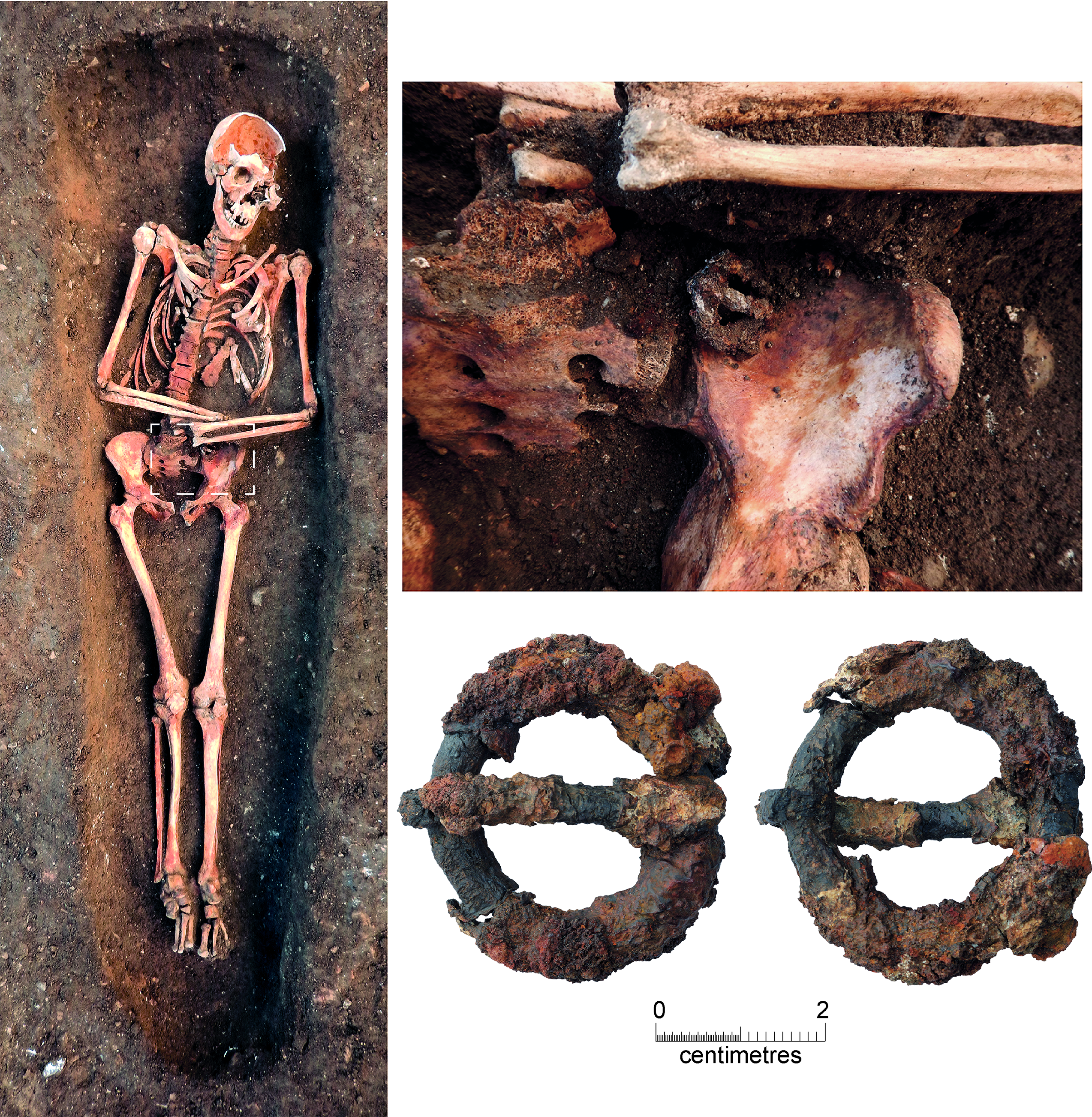

Supplement: Supplemental Material [file RAIJ_A_2090675_SM0285.zip › Supplementary text and figures/Figure_S21 F311.tif]

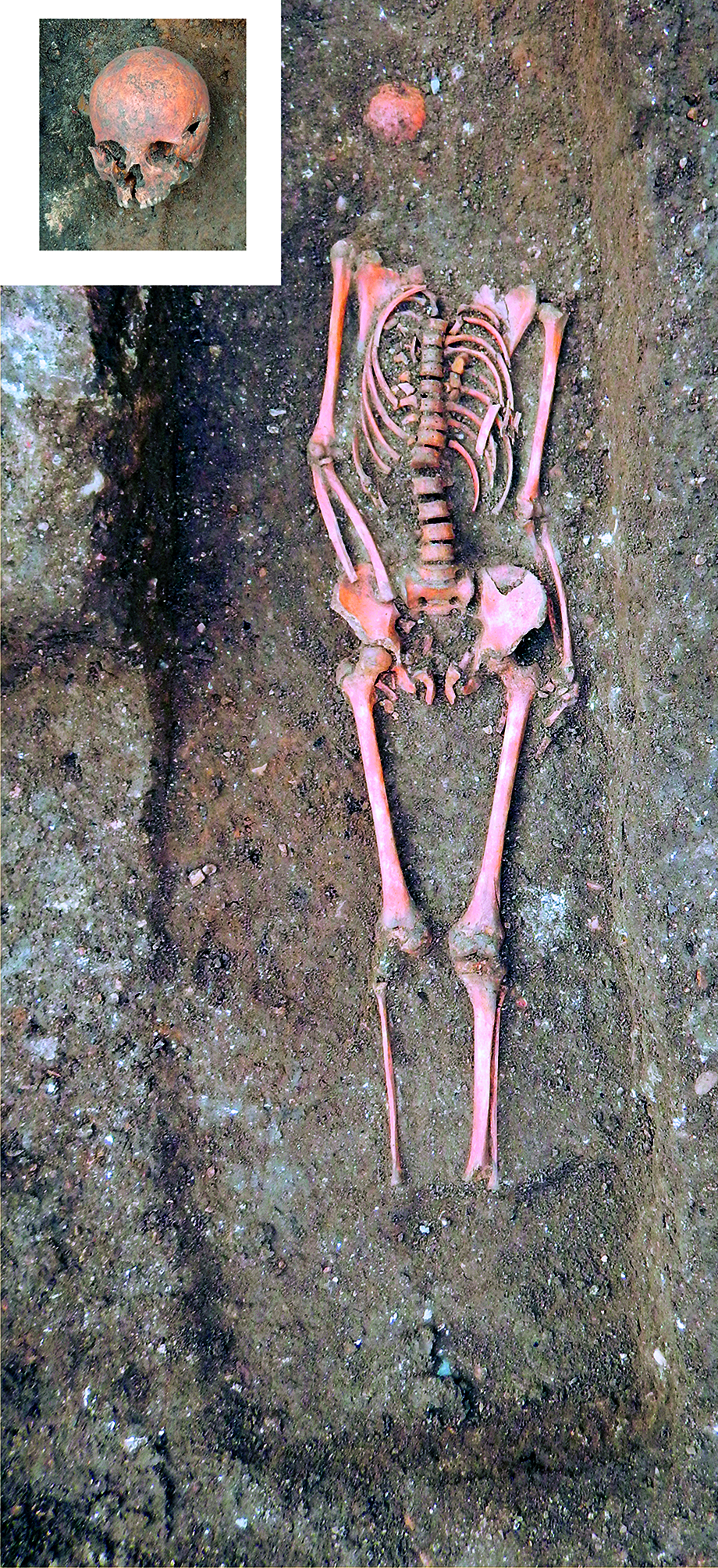

Supplement: Supplemental Material [file RAIJ_A_2090675_SM0285.zip › Supplementary text and figures/Figure_S22 F312.tif]

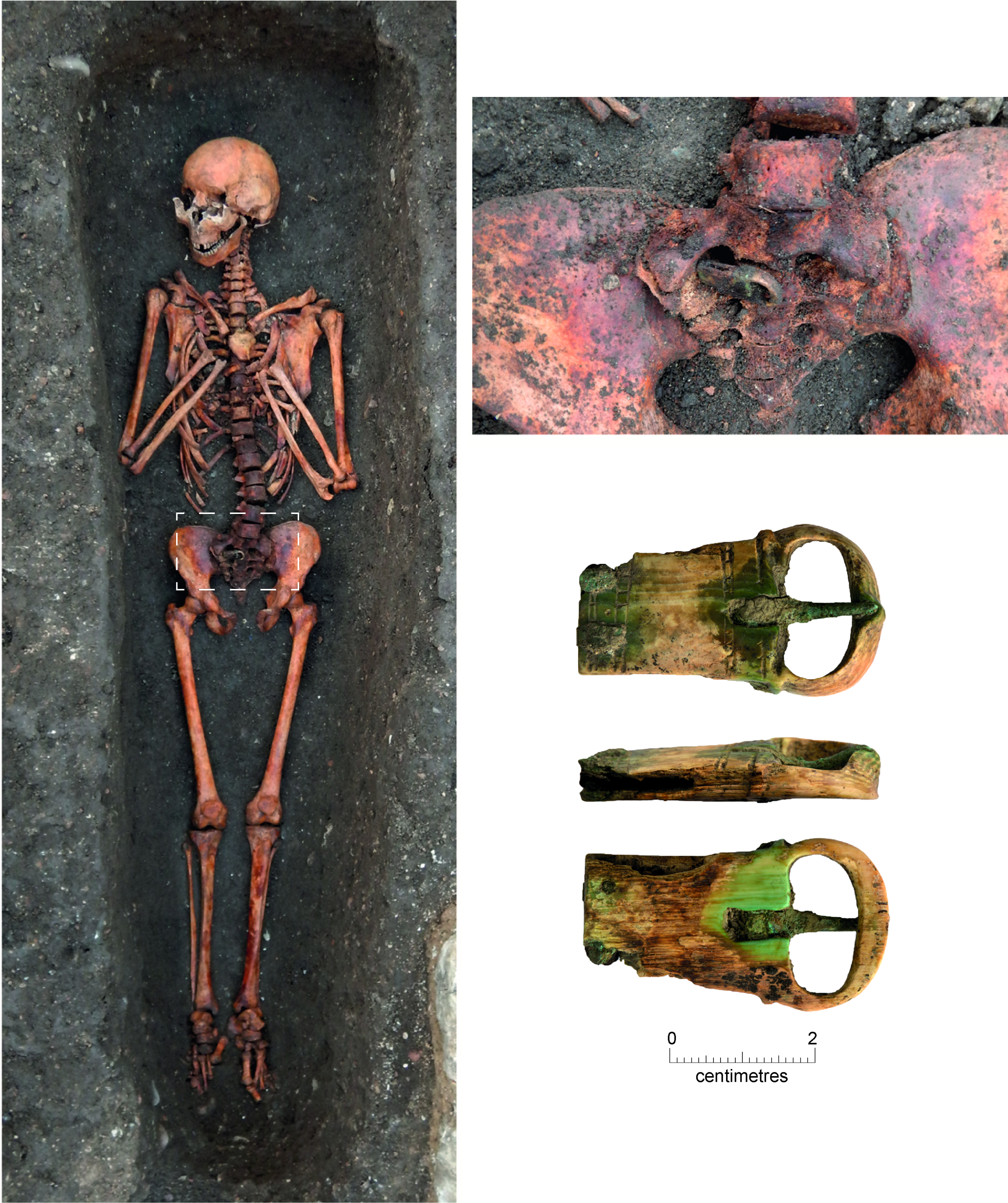

Supplement: Supplemental Material [file RAIJ_A_2090675_SM0285.zip › Supplementary text and figures/Figure_S23 F314.tif]

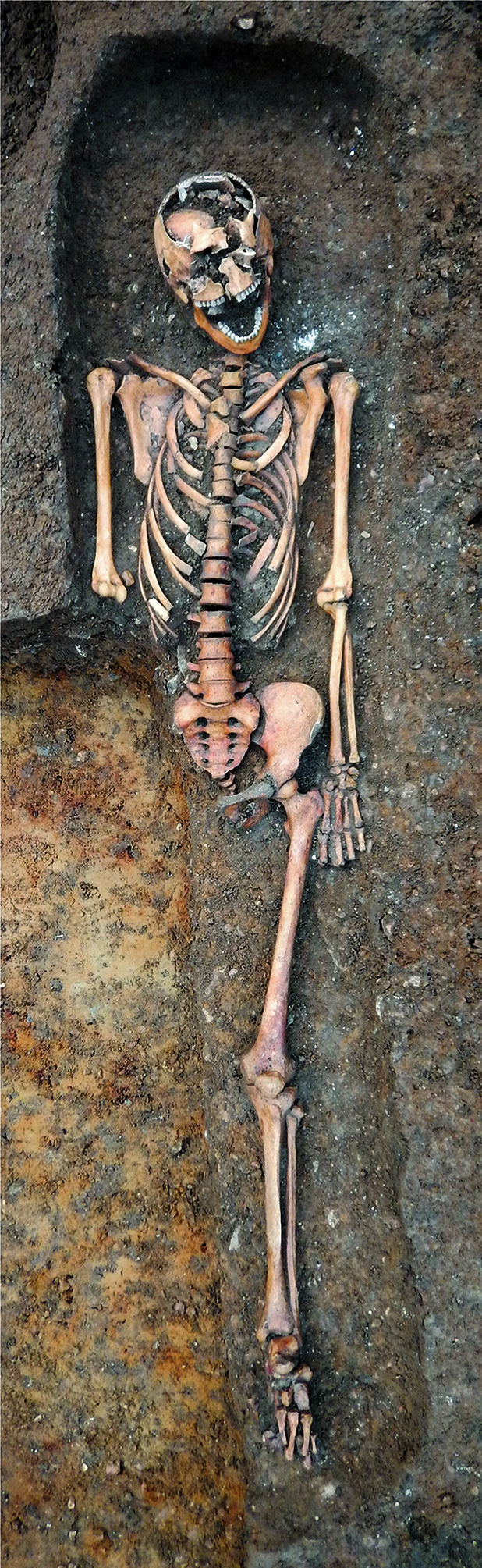

Supplement: Supplemental Material [file RAIJ_A_2090675_SM0285.zip › Supplementary text and figures/Figure_S24 F315.tif]

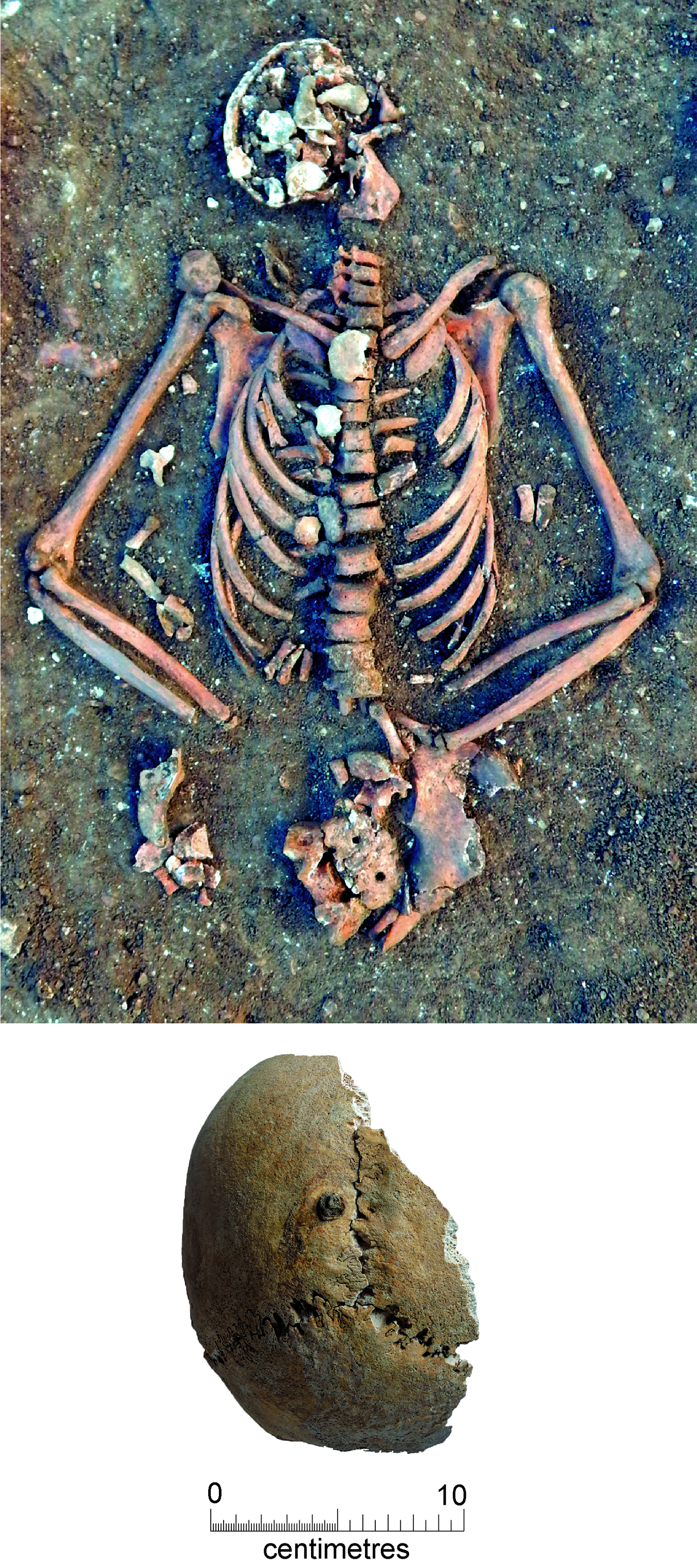

Supplement: Supplemental Material [file RAIJ_A_2090675_SM0285.zip › Supplementary text and figures/Figure_S25 F328.tif]

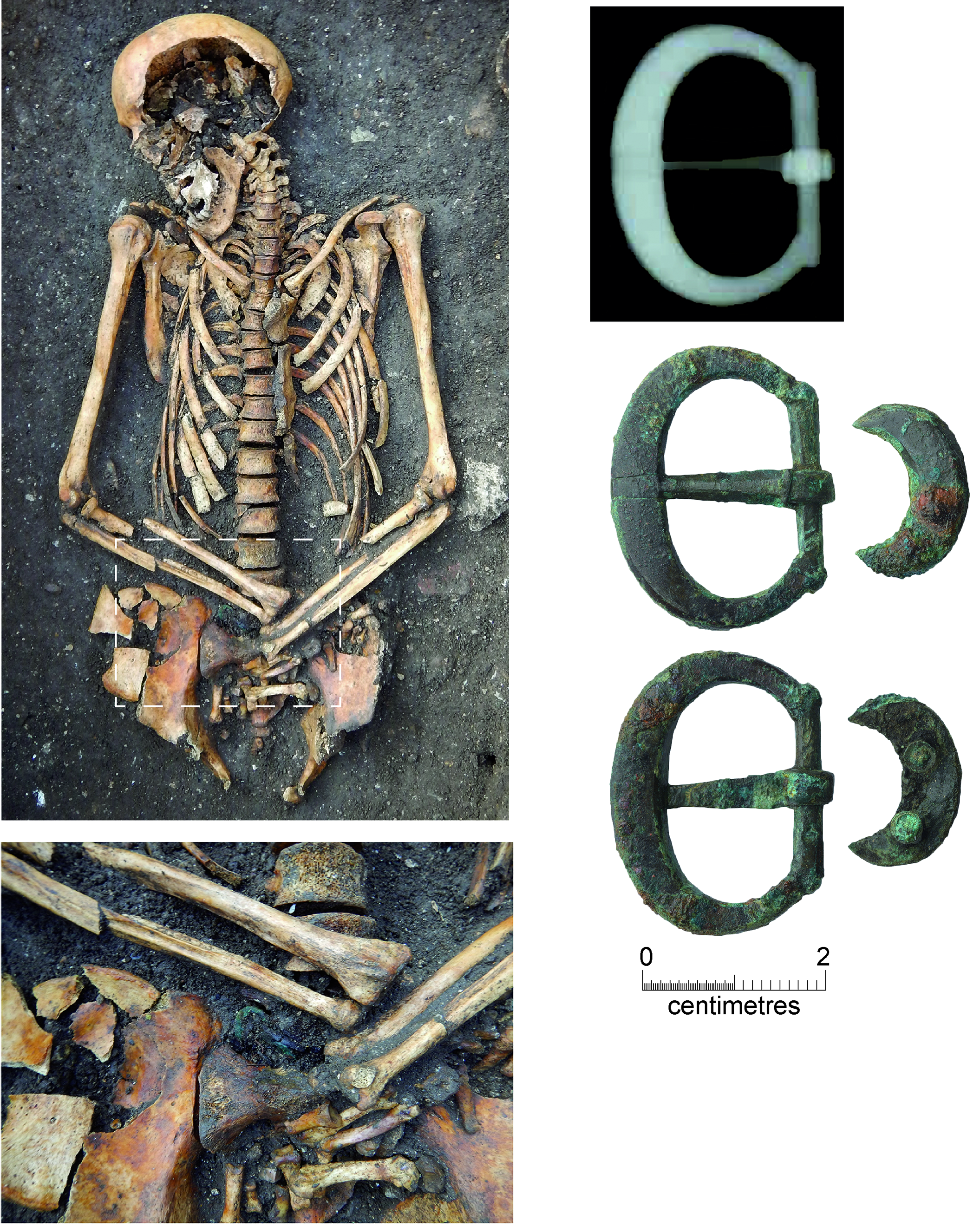

Supplement: Supplemental Material [file RAIJ_A_2090675_SM0285.zip › Supplementary text and figures/Figure_S26 F331.tif]

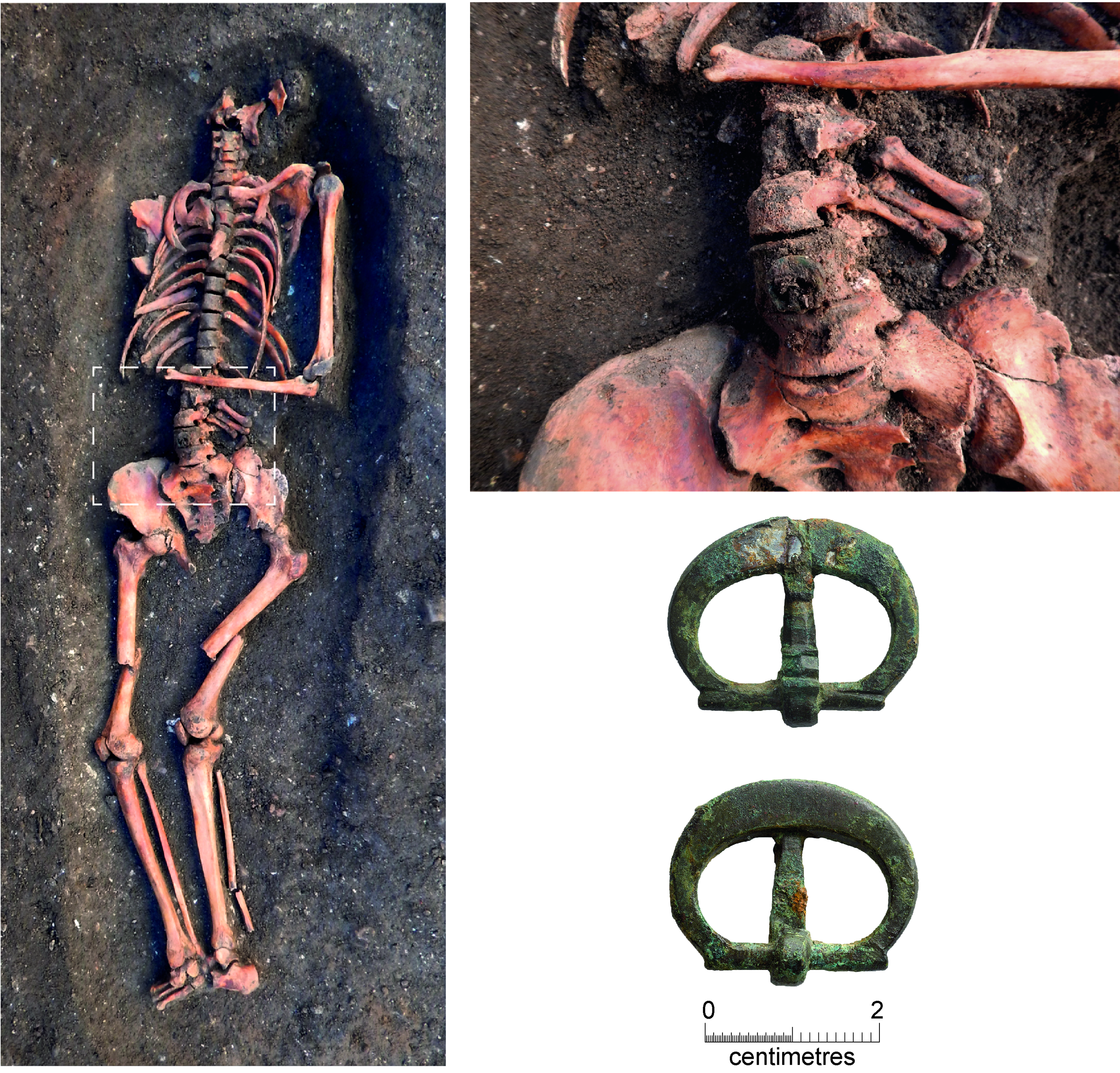

Supplement: Supplemental Material [file RAIJ_A_2090675_SM0285.zip › Supplementary text and figures/Figure_S27 F332.tif]

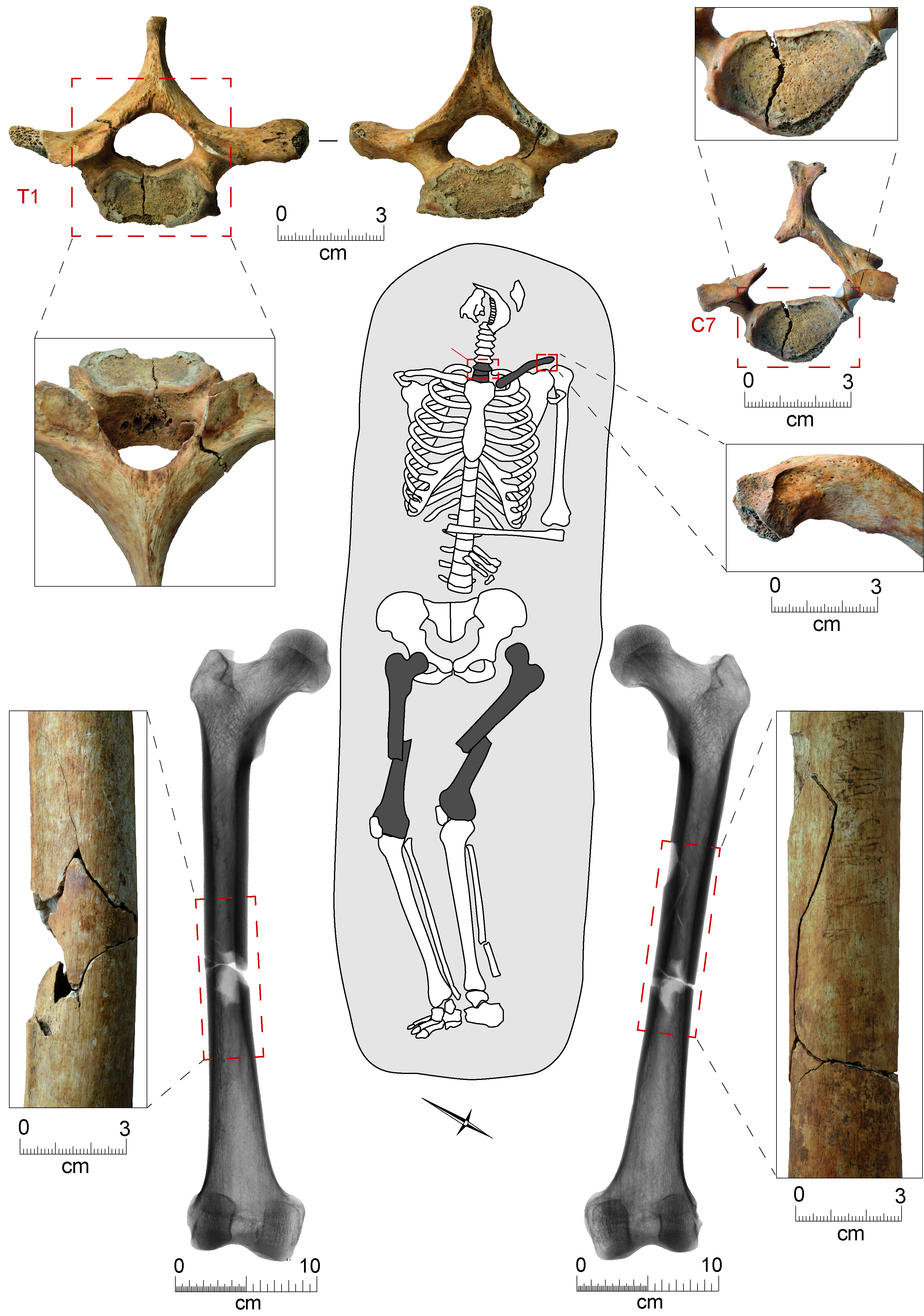

Supplement: Supplemental Material [file RAIJ_A_2090675_SM0285.zip › Supplementary text and figures/Figure_S28 F.332 mod.tif]

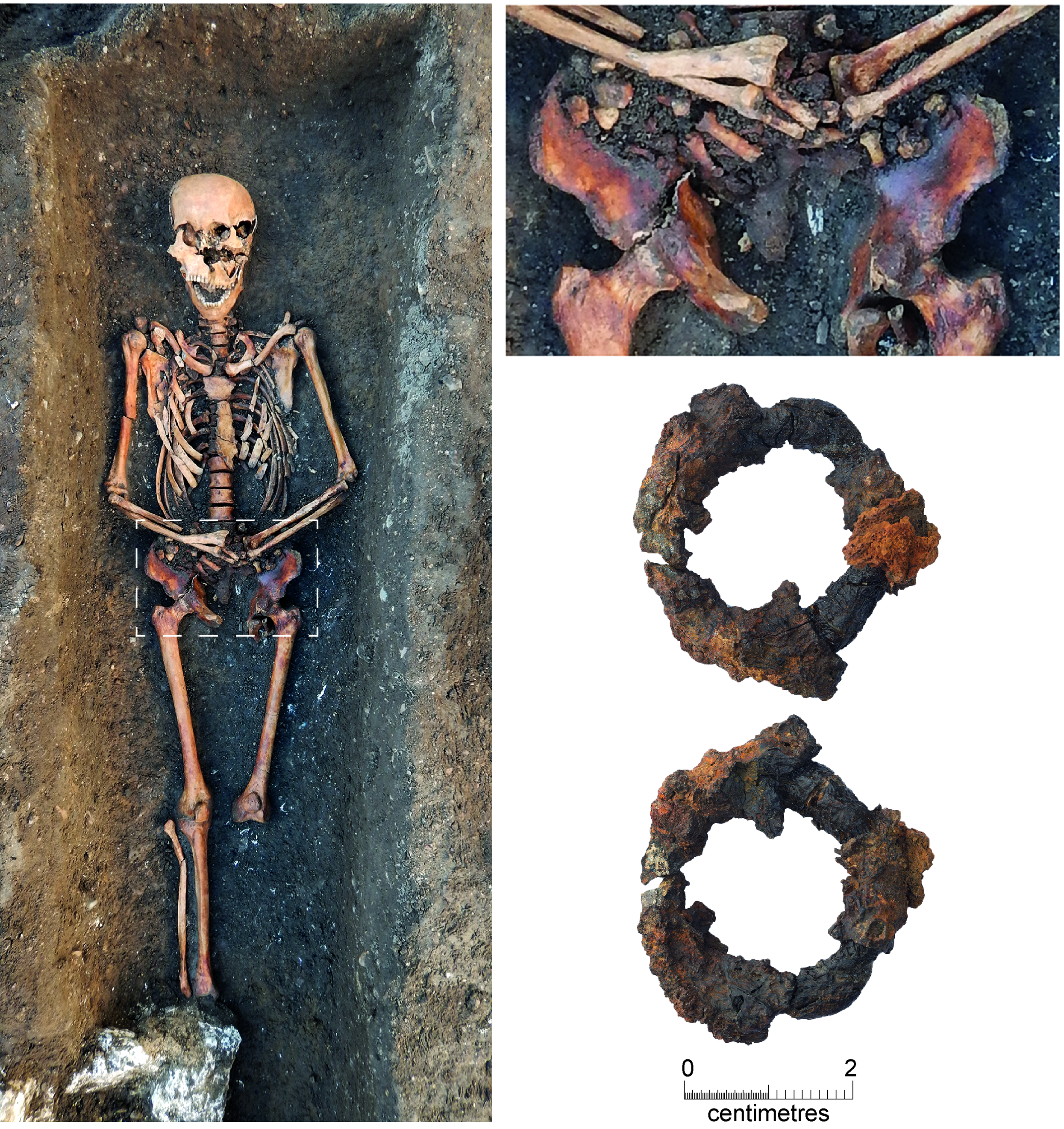

Supplement: Supplemental Material [file RAIJ_A_2090675_SM0285.zip › Supplementary text and figures/Figure_S29 F333.tif]

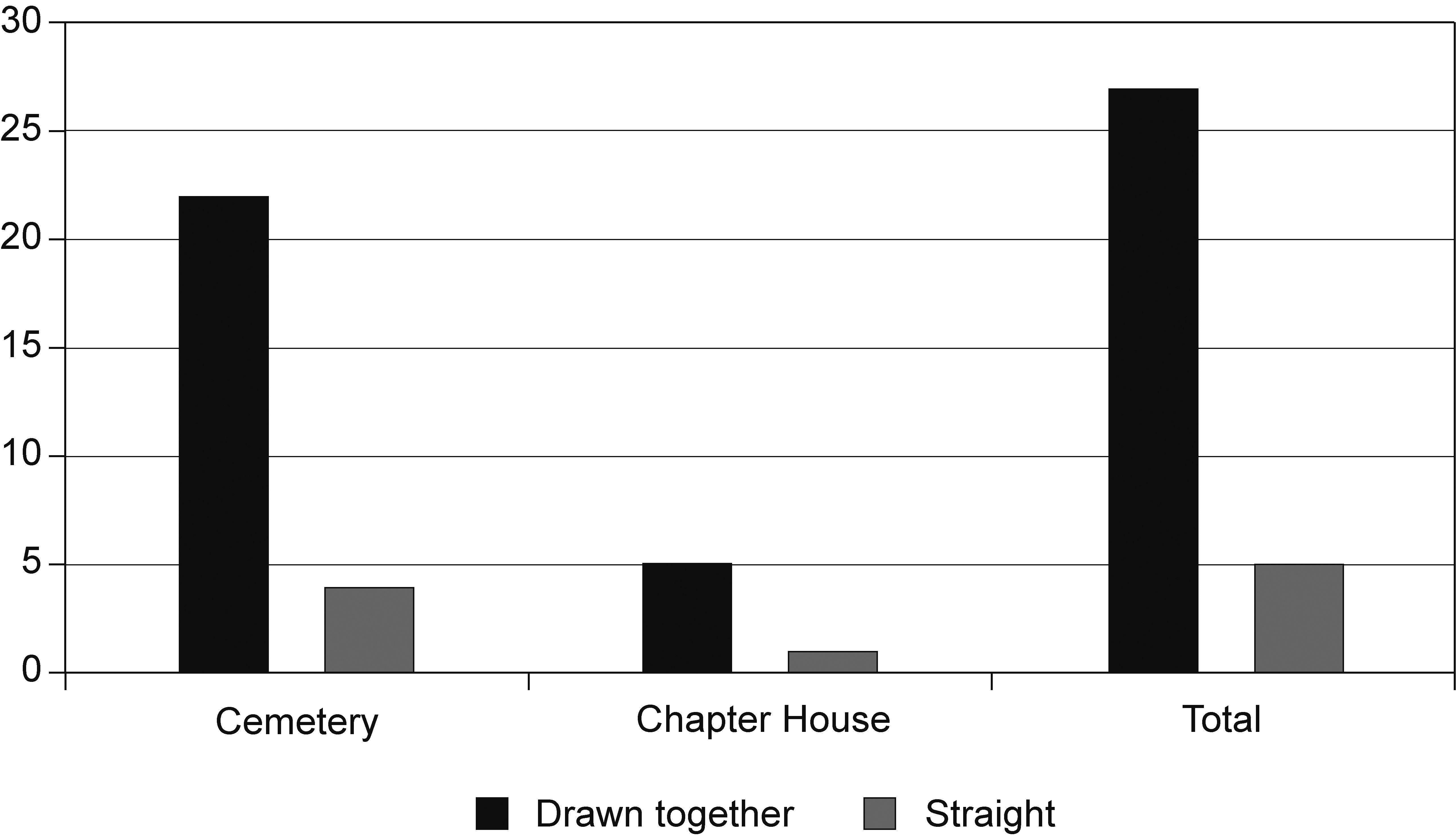

Supplement: Supplemental Material [file RAIJ_A_2090675_SM0285.zip › Supplementary text and figures/Figure_S3 Leg position graph.tif]

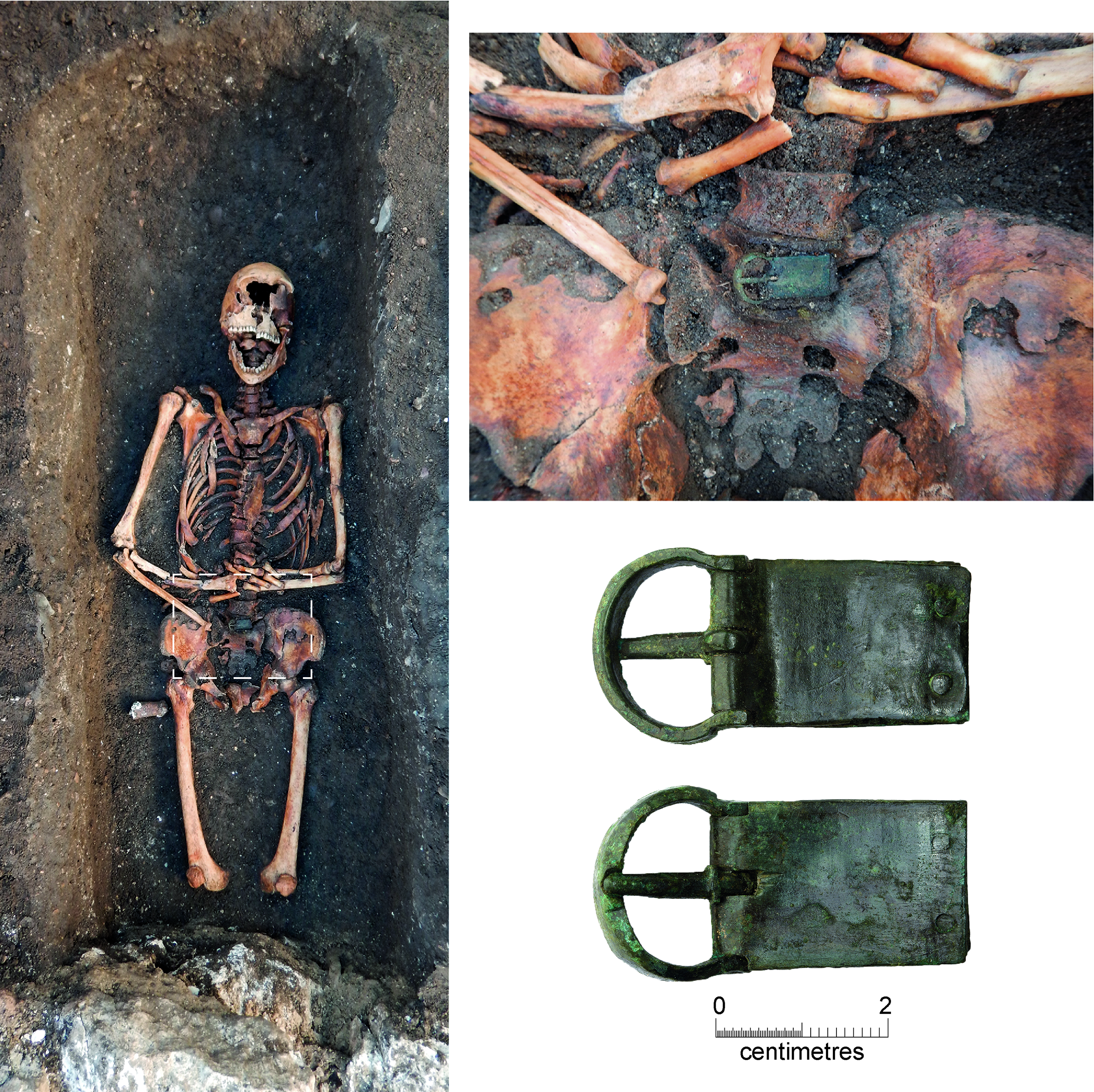

Supplement: Supplemental Material [file RAIJ_A_2090675_SM0285.zip › Supplementary text and figures/Figure_S30 F334.tif]

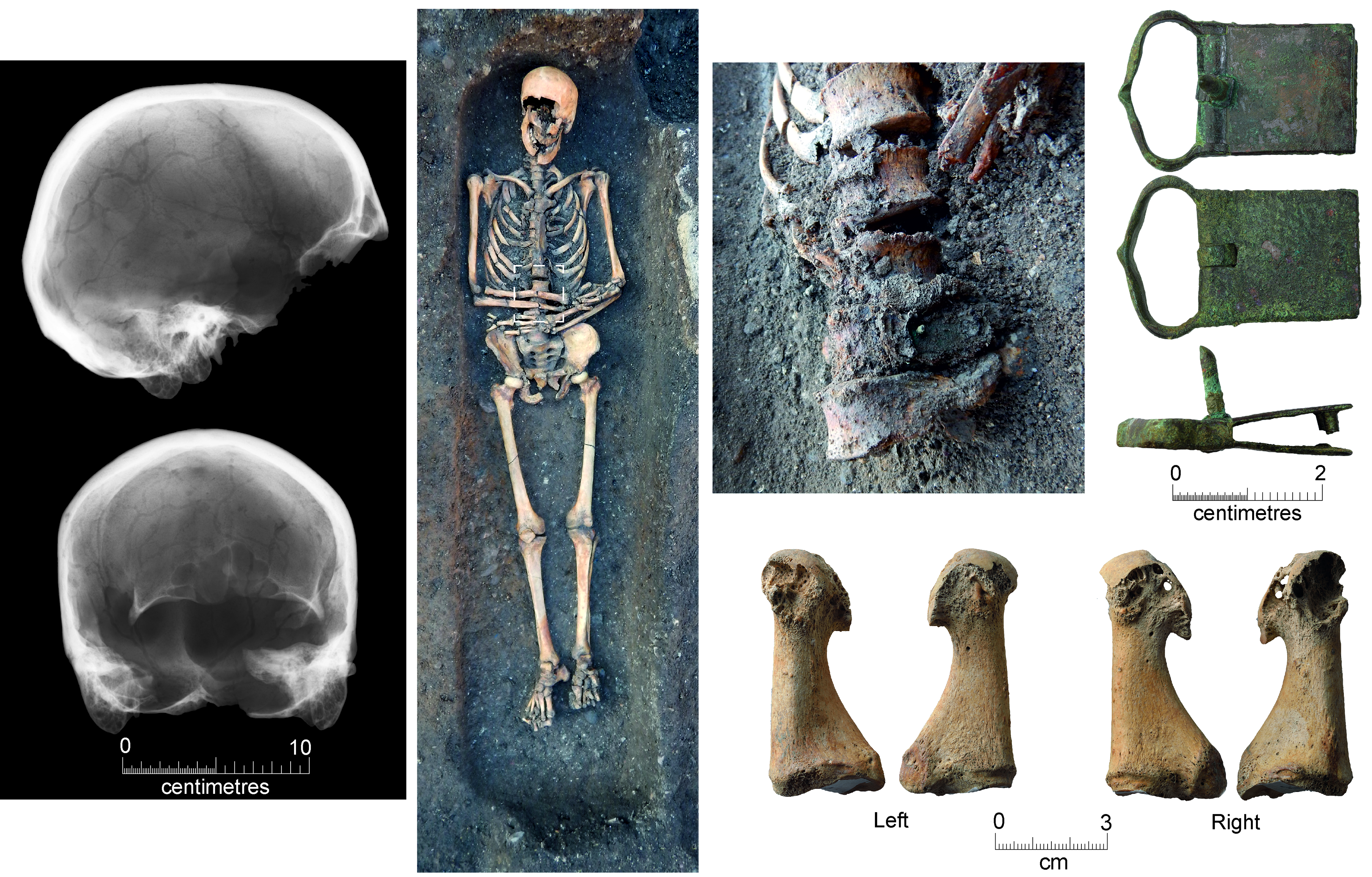

Supplement: Supplemental Material [file RAIJ_A_2090675_SM0285.zip › Supplementary text and figures/Figure_S31 F336.tif]

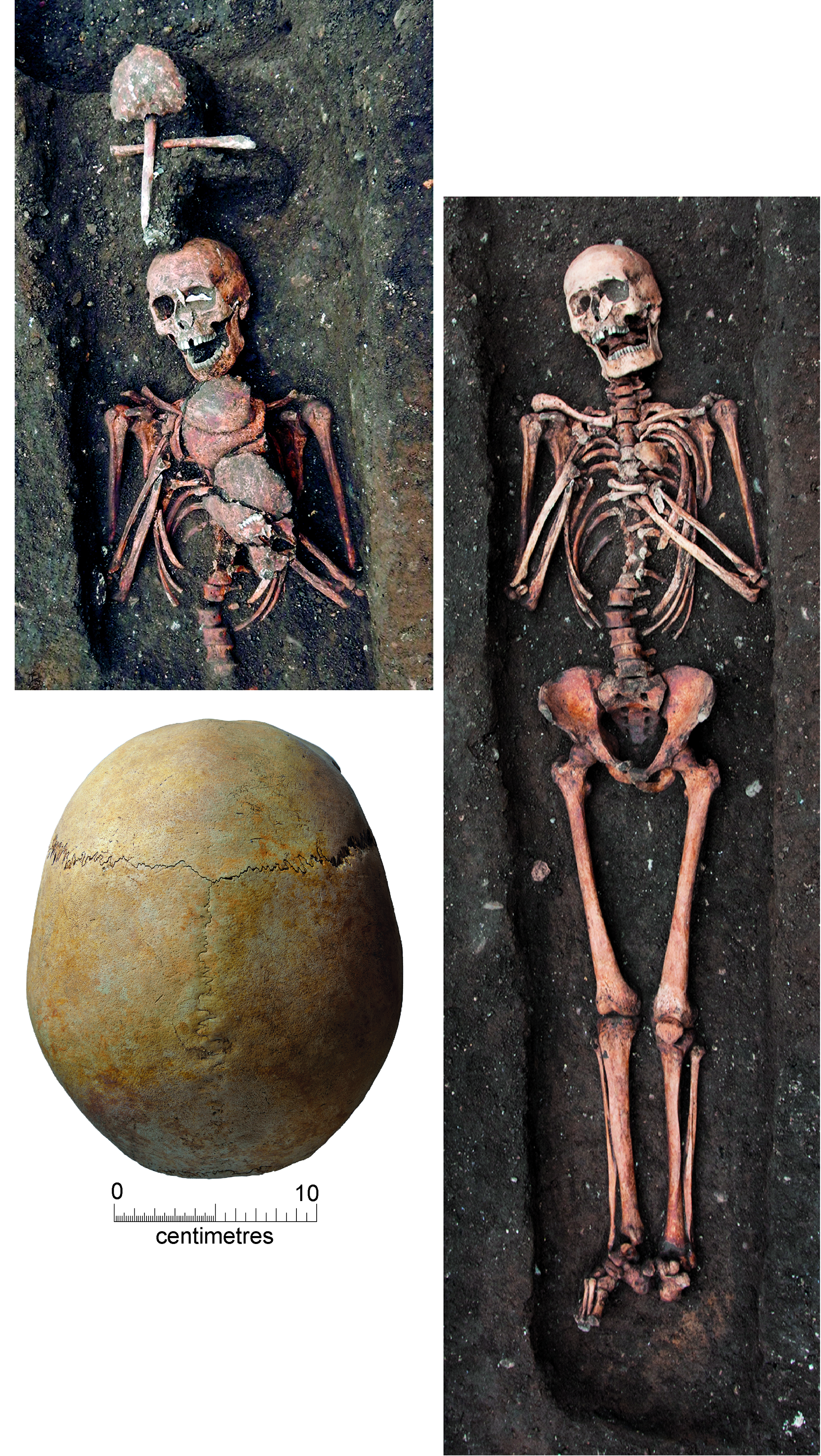

Supplement: Supplemental Material [file RAIJ_A_2090675_SM0285.zip › Supplementary text and figures/Figure_S32 F343.tif]

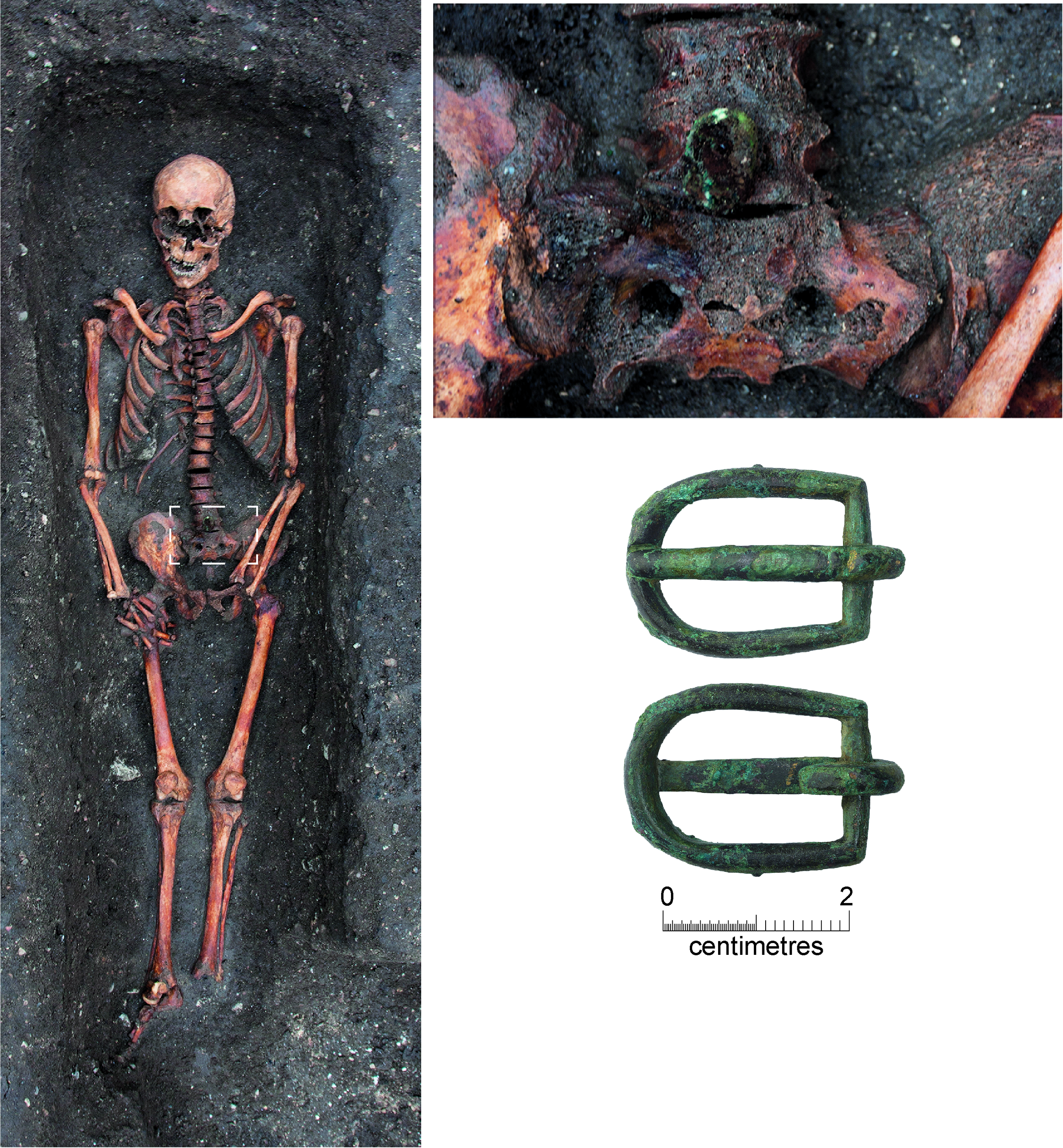

Supplement: Supplemental Material [file RAIJ_A_2090675_SM0285.zip › Supplementary text and figures/Figure_S33 F344.tif]

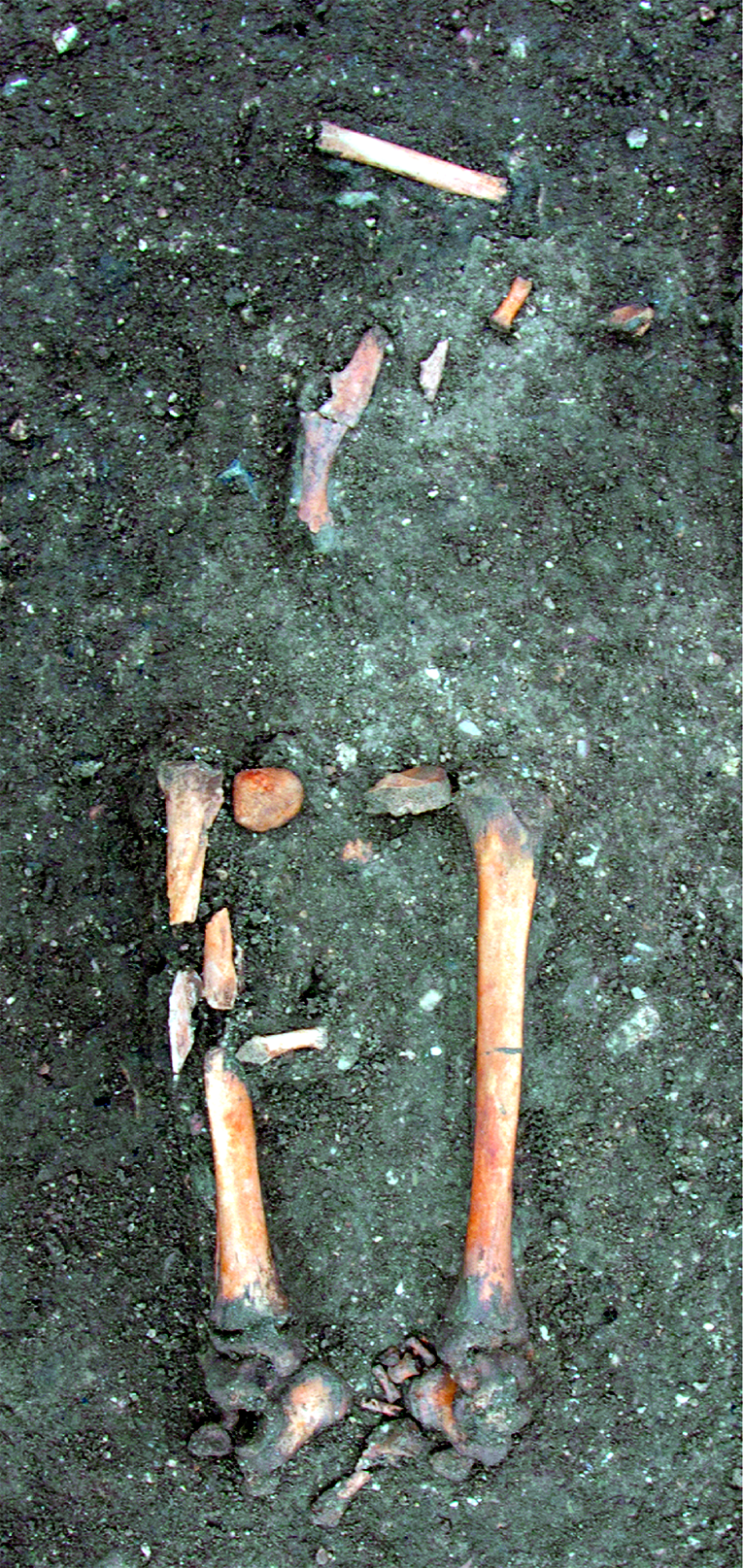

Supplement: Supplemental Material [file RAIJ_A_2090675_SM0285.zip › Supplementary text and figures/Figure_S34 F346.tif]

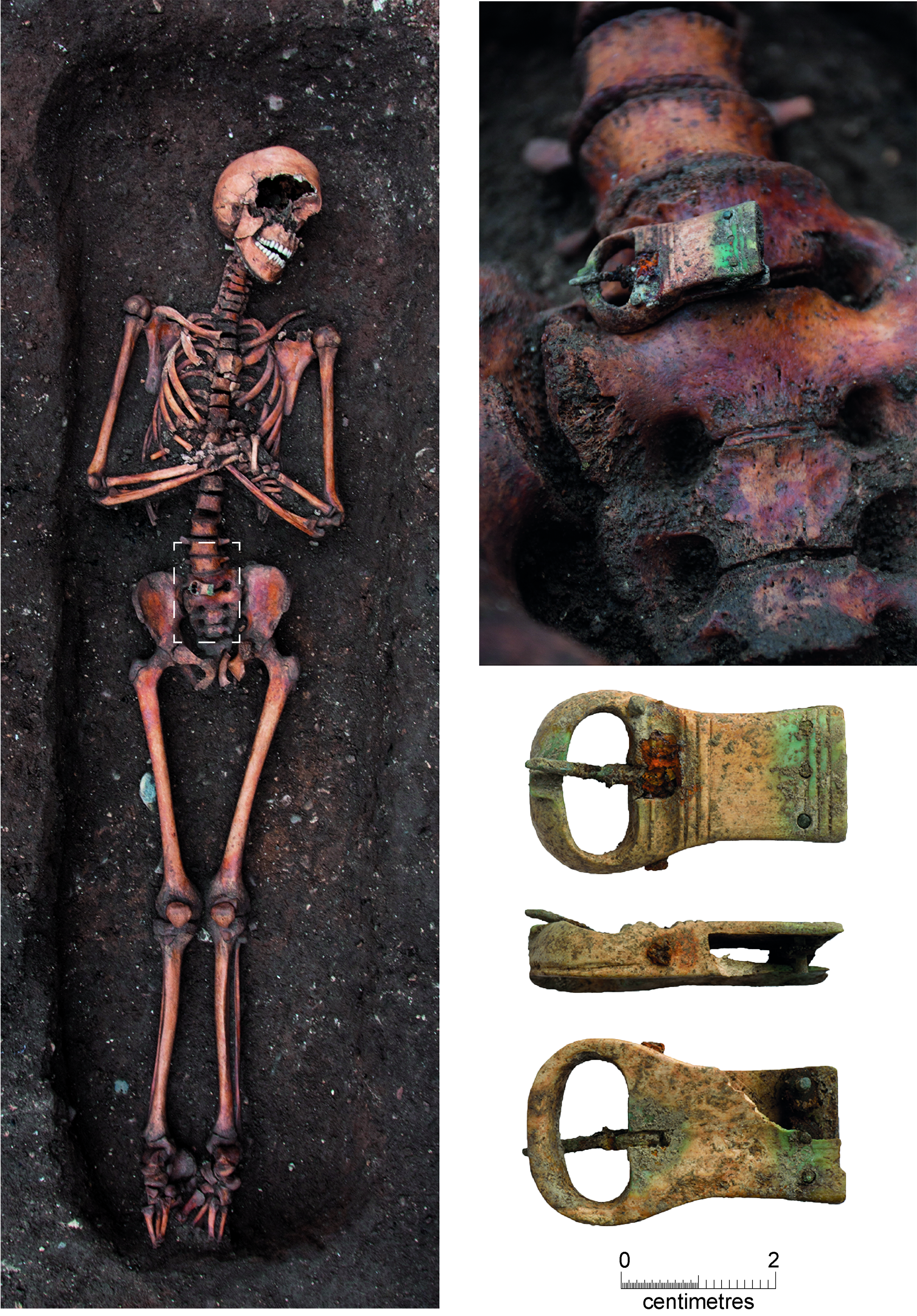

Supplement: Supplemental Material [file RAIJ_A_2090675_SM0285.zip › Supplementary text and figures/Figure_S35 F347.tif]

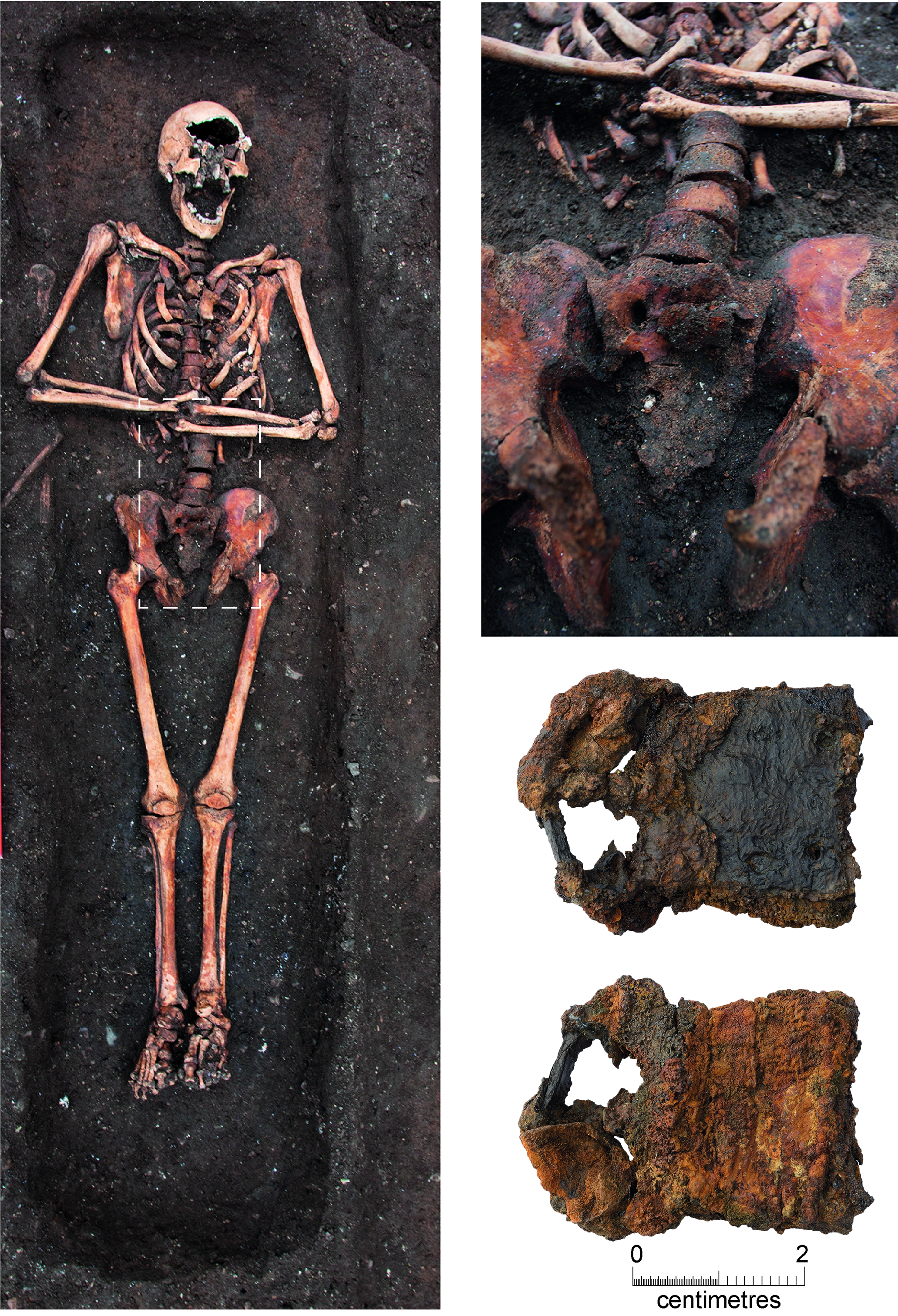

Supplement: Supplemental Material [file RAIJ_A_2090675_SM0285.zip › Supplementary text and figures/Figure_S36 F348.tif]

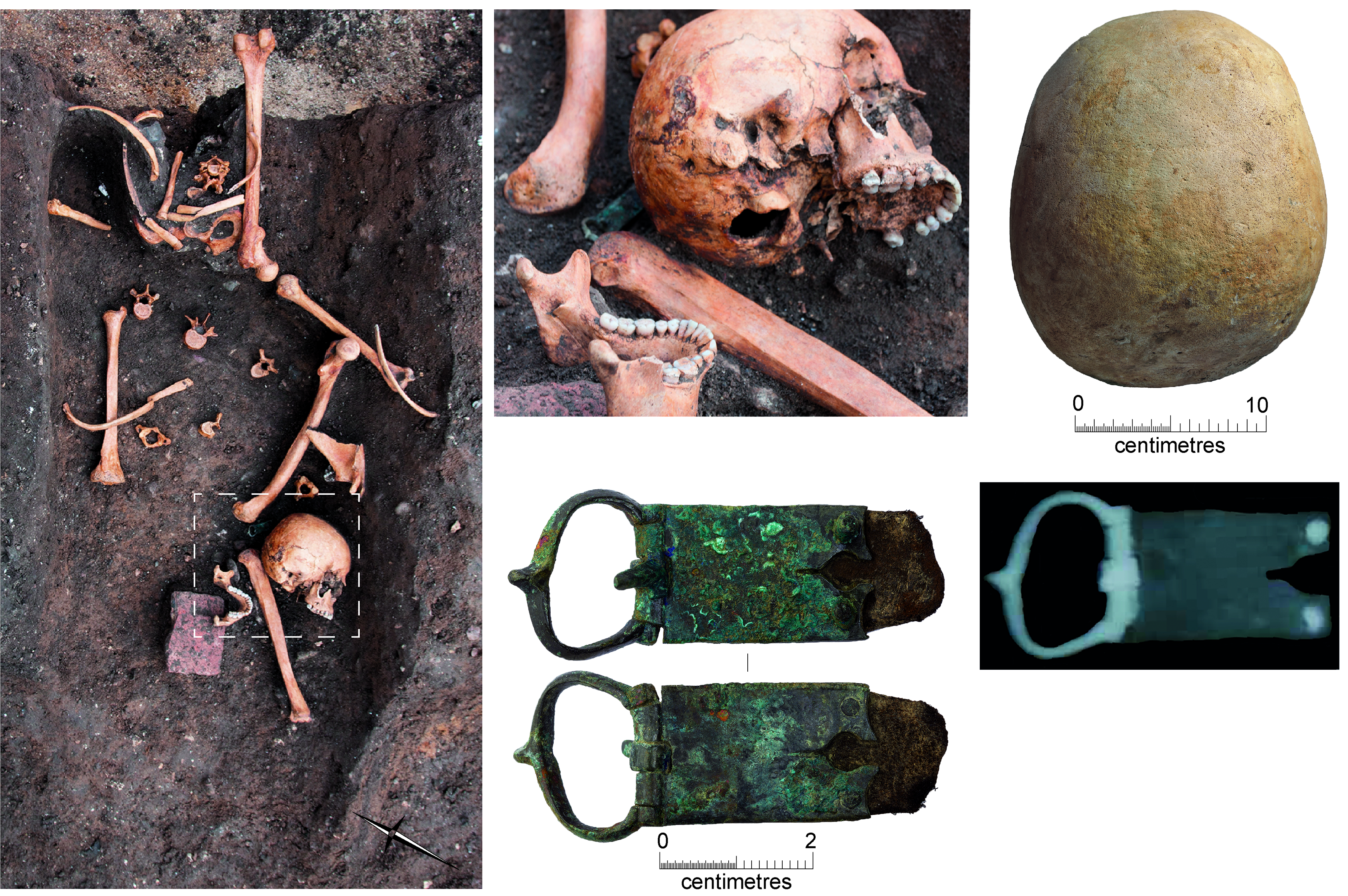

Supplement: Supplemental Material [file RAIJ_A_2090675_SM0285.zip › Supplementary text and figures/Figure_S37 F352.tif]

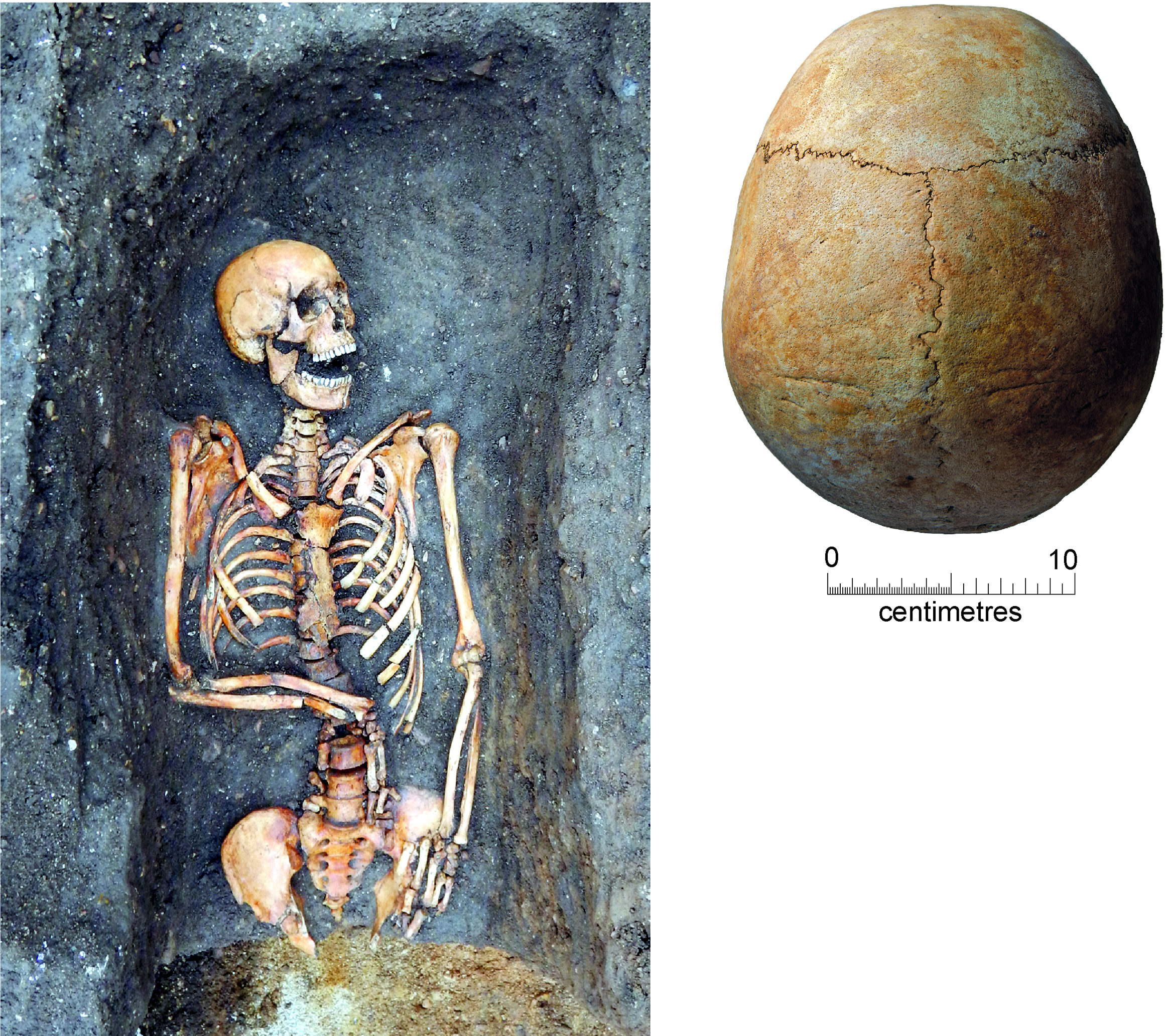

Supplement: Supplemental Material [file RAIJ_A_2090675_SM0285.zip › Supplementary text and figures/Figure_S38 F355.tif]

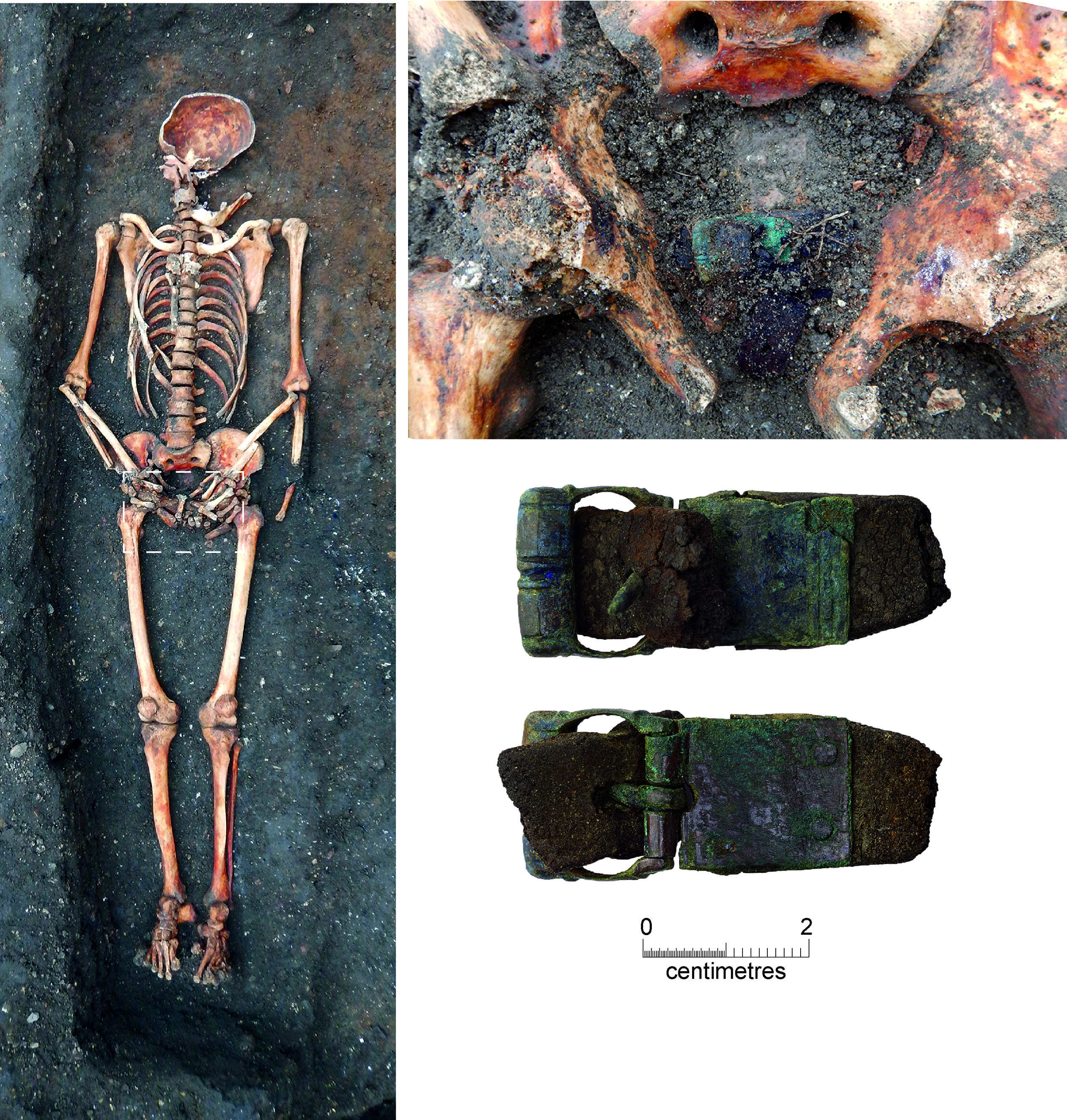

Supplement: Supplemental Material [file RAIJ_A_2090675_SM0285.zip › Supplementary text and figures/Figure_S39 F367.tif]

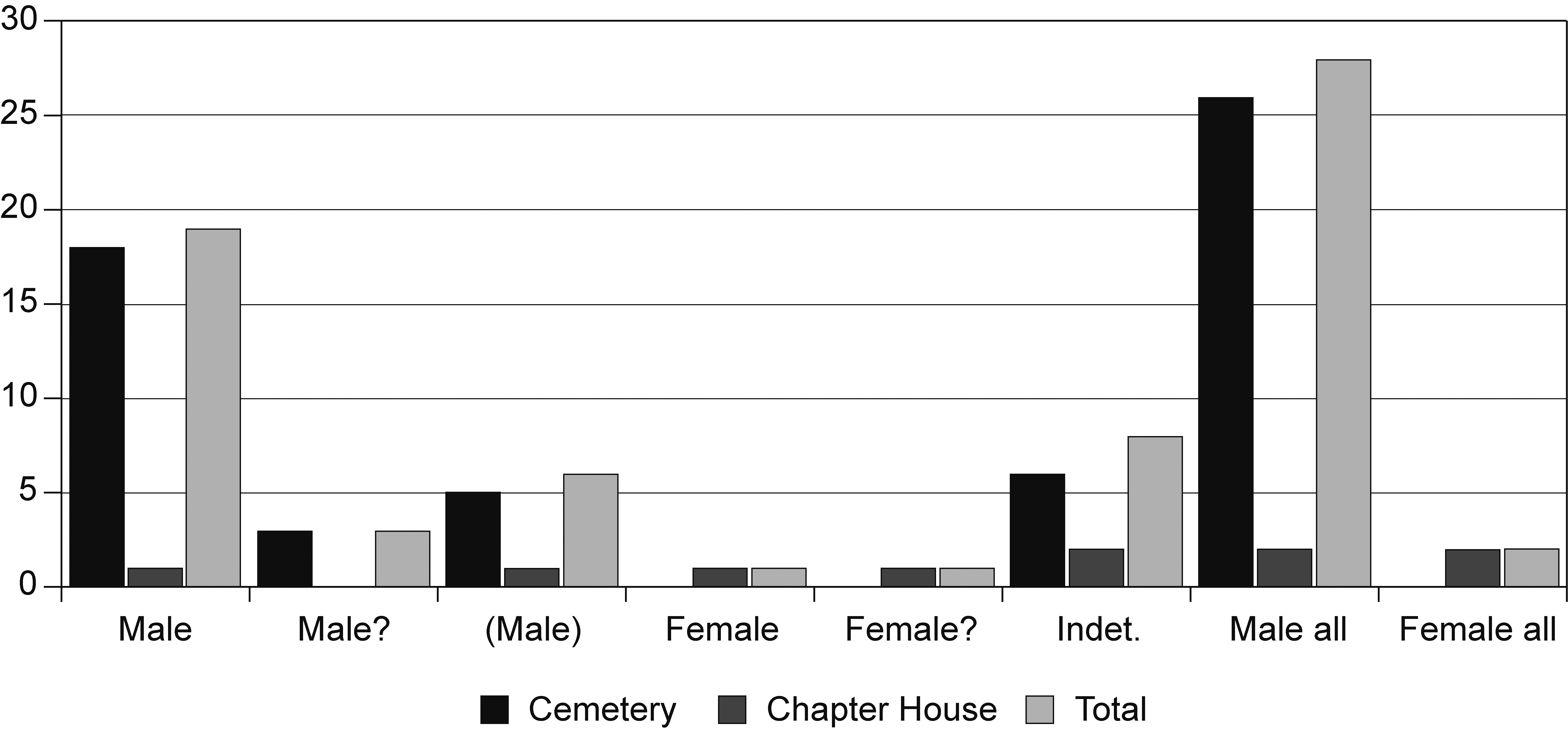

Supplement: Supplemental Material [file RAIJ_A_2090675_SM0285.zip › Supplementary text and figures/Figure_S4 Osteological sex graph.tif]

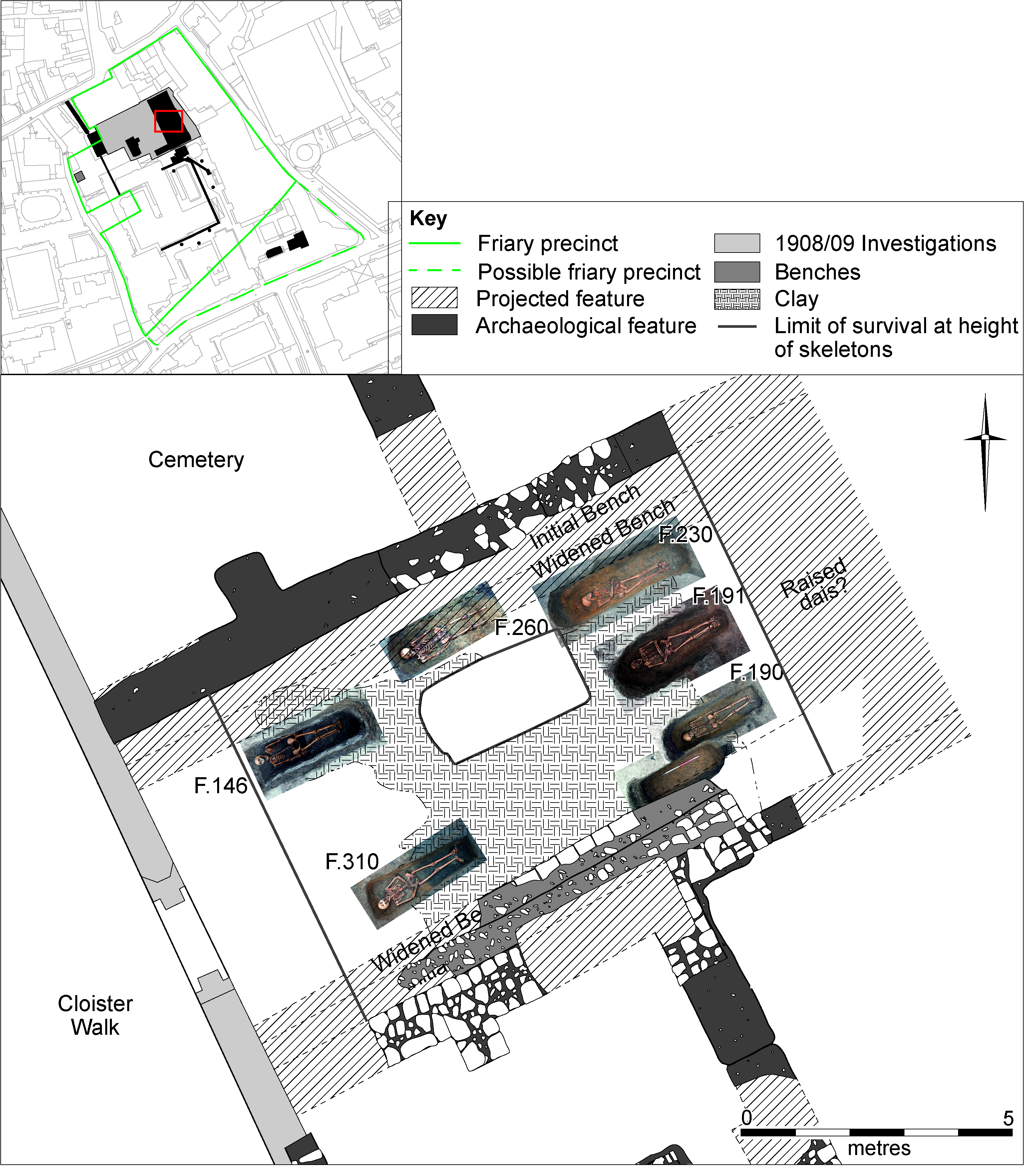

Supplement: Supplemental Material [file RAIJ_A_2090675_SM0285.zip › Supplementary text and figures/Figure_S40 Plan of Chapter House with photos.tif]

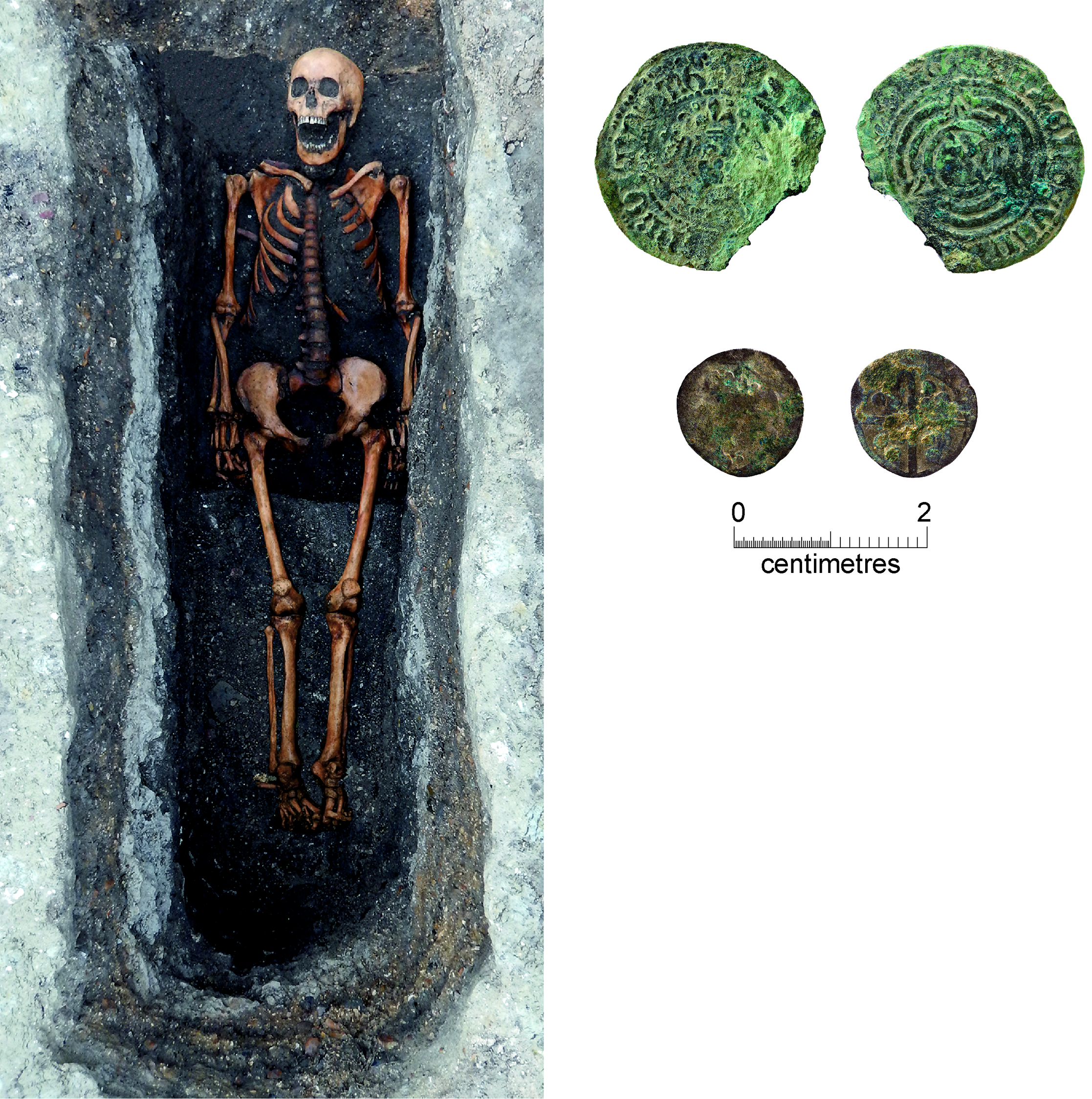

Supplement: Supplemental Material [file RAIJ_A_2090675_SM0285.zip › Supplementary text and figures/Figure_S41 F146.tif]

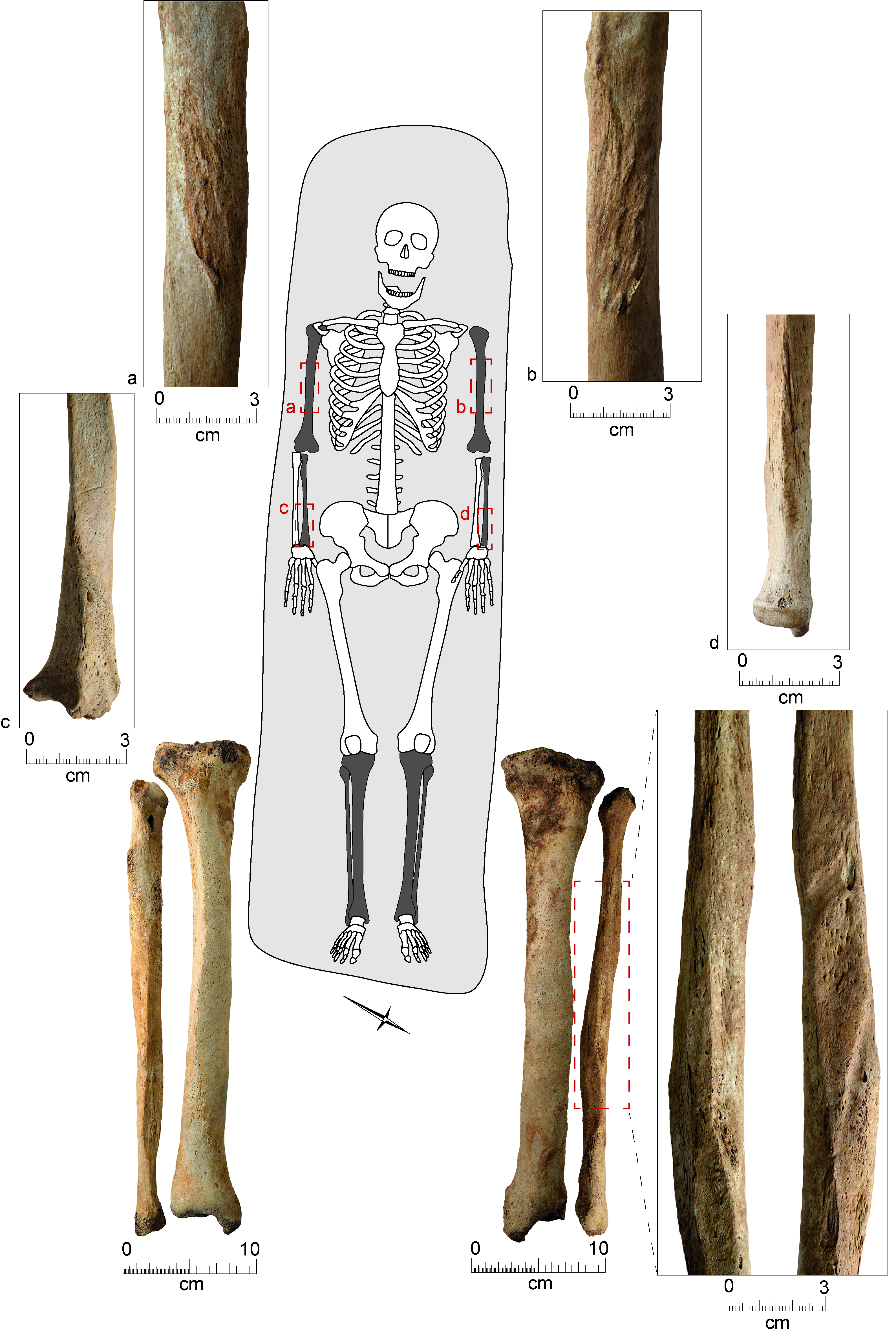

Supplement: Supplemental Material [file RAIJ_A_2090675_SM0285.zip › Supplementary text and figures/Figure_S42 F146 pathology.tif]

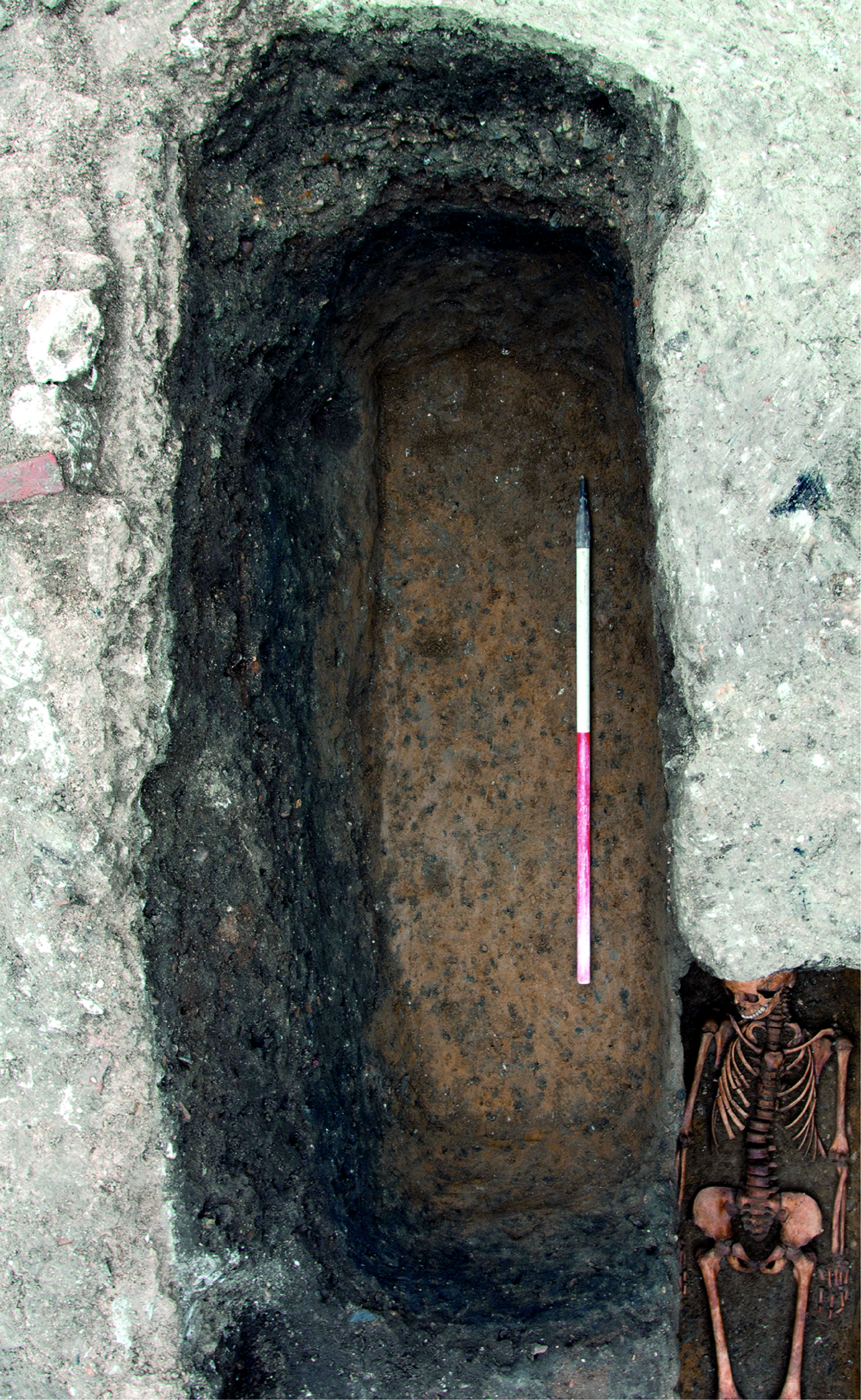

Supplement: Supplemental Material [file RAIJ_A_2090675_SM0285.zip › Supplementary text and figures/Figure_S43 F189.tif]

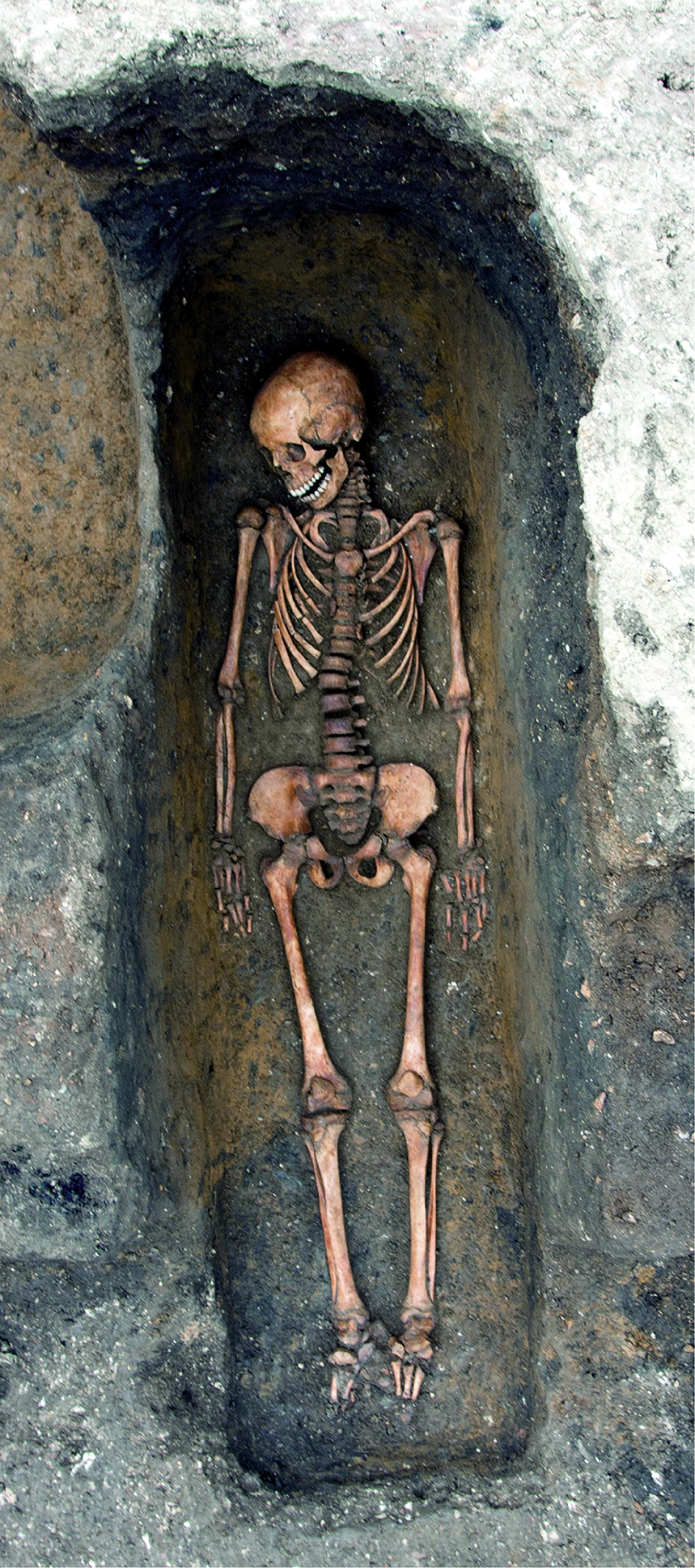

Supplement: Supplemental Material [file RAIJ_A_2090675_SM0285.zip › Supplementary text and figures/Figure_S44 F190.tif]

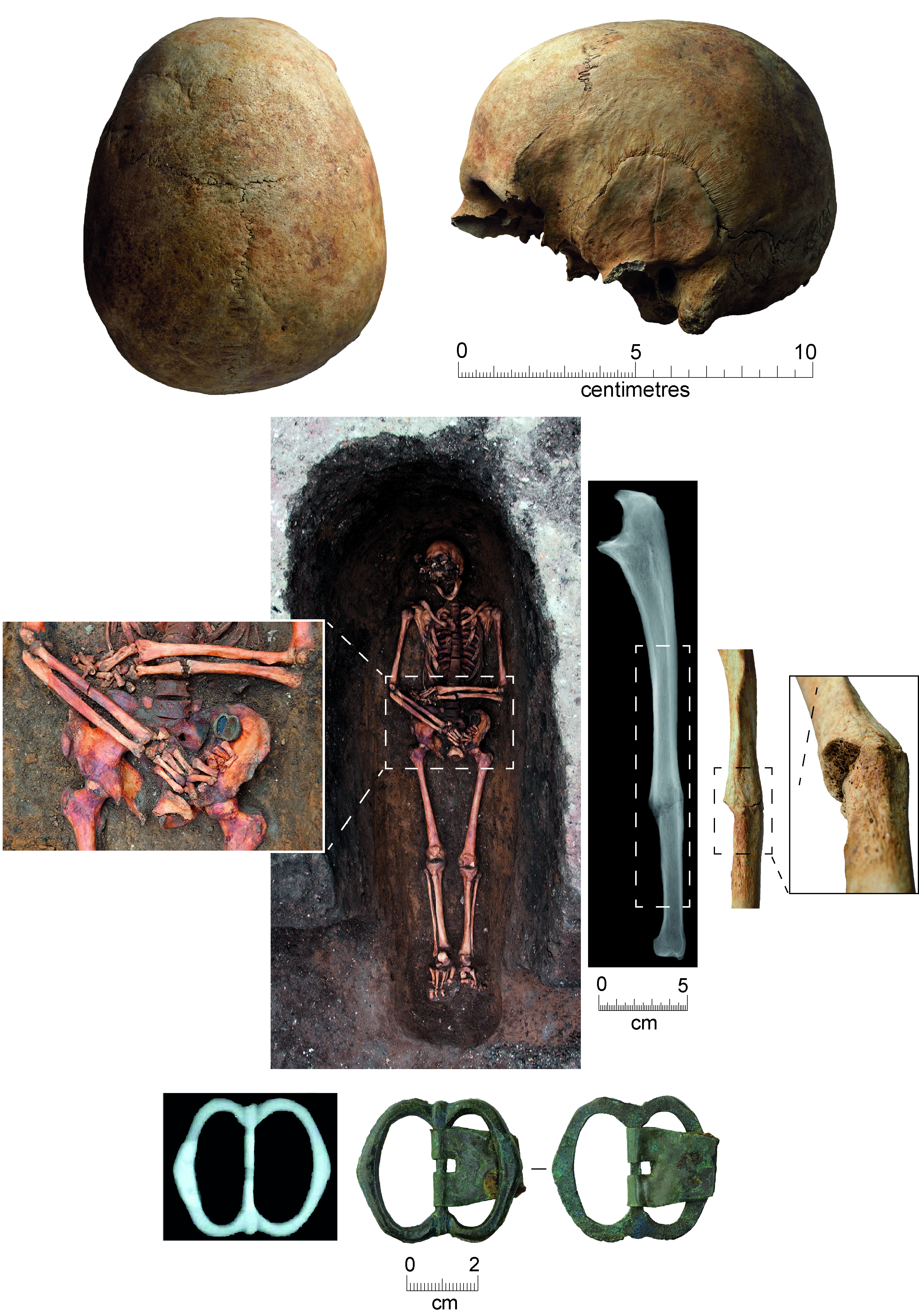

Supplement: Supplemental Material [file RAIJ_A_2090675_SM0285.zip › Supplementary text and figures/Figure_S45 F191.tif]

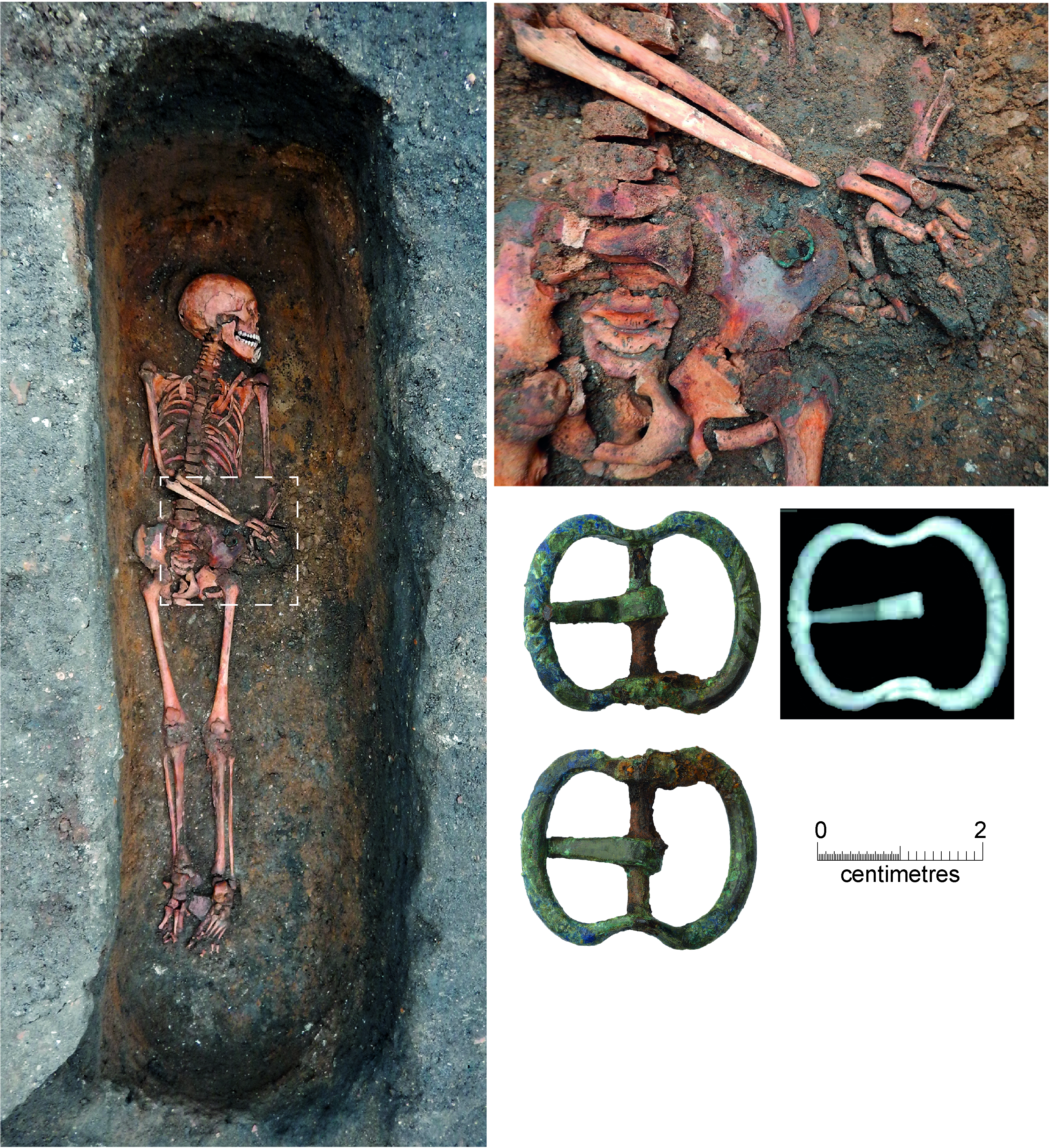

Supplement: Supplemental Material [file RAIJ_A_2090675_SM0285.zip › Supplementary text and figures/Figure_S46 F230.tif]

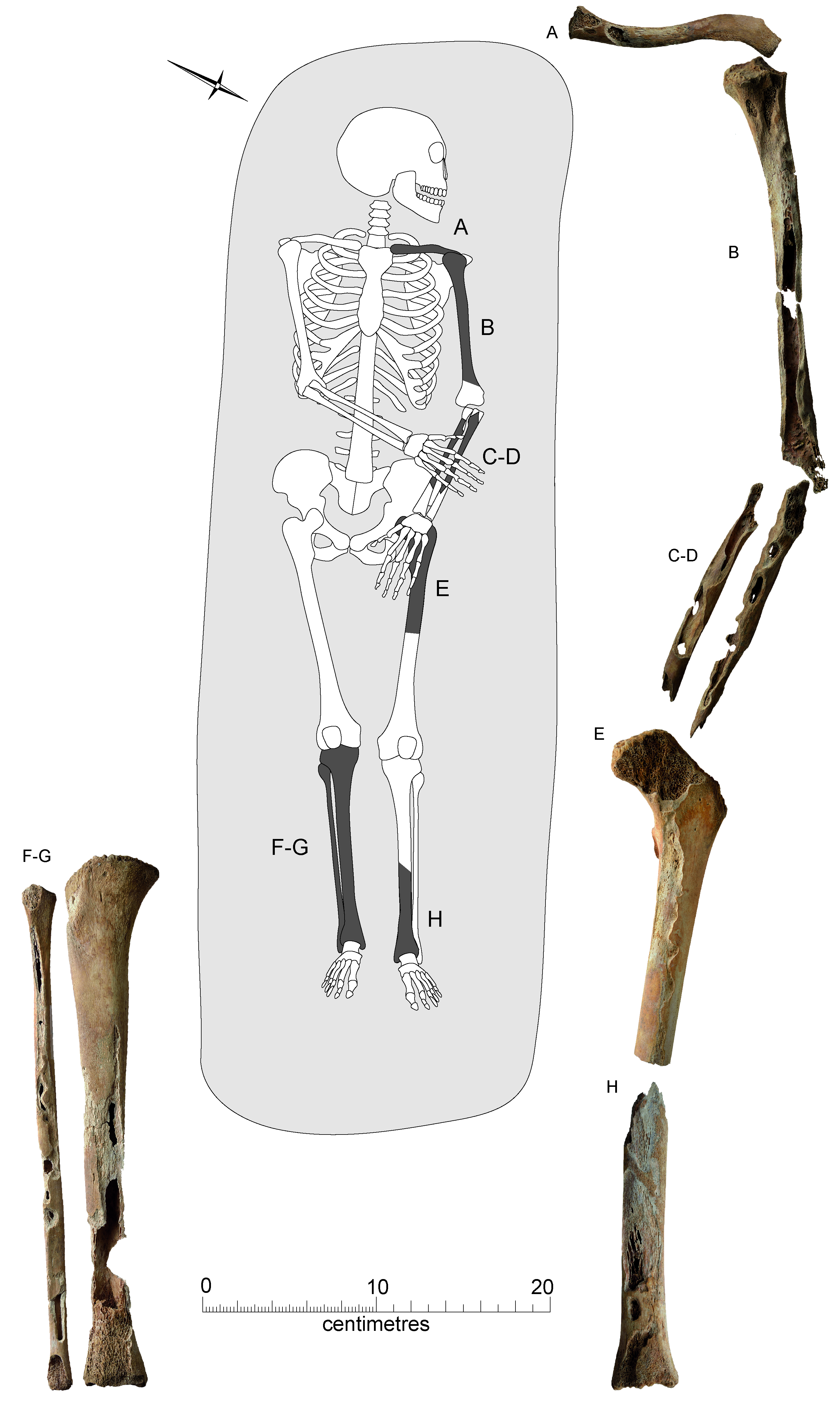

Supplement: Supplemental Material [file RAIJ_A_2090675_SM0285.zip › Supplementary text and figures/Figure_S47 F230 pathology.tif]

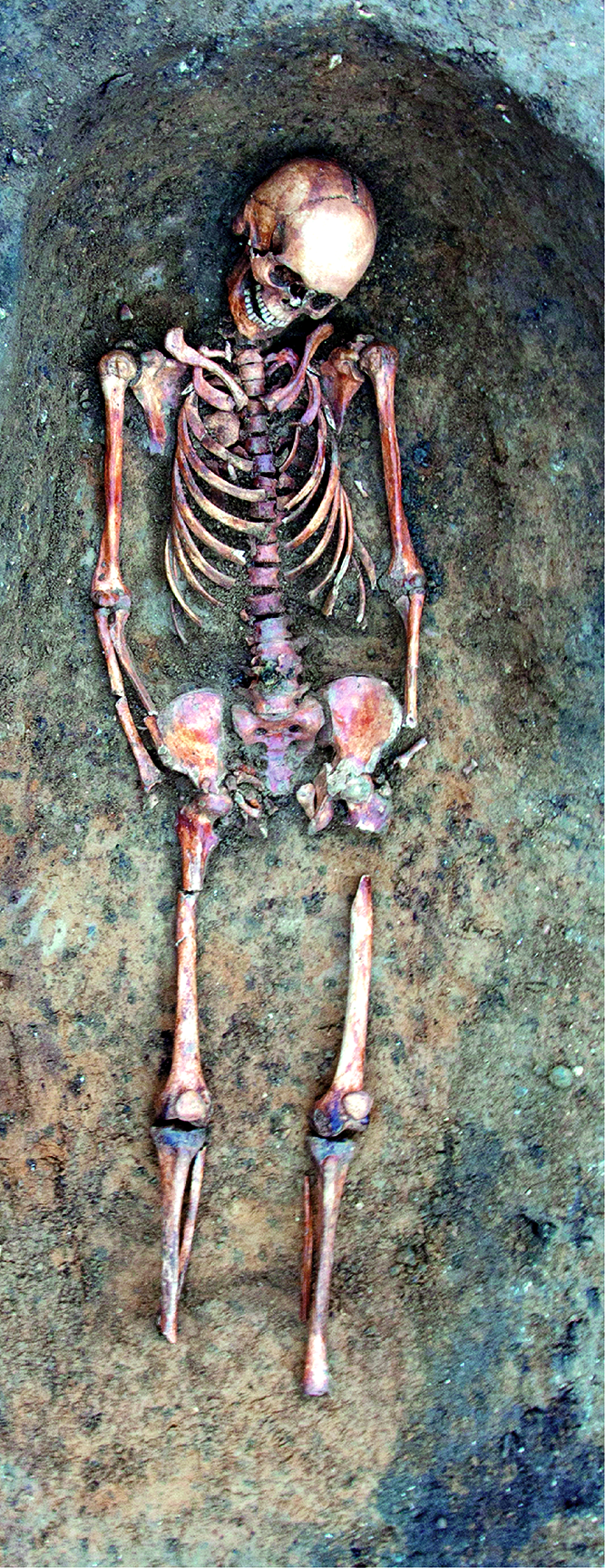

Supplement: Supplemental Material [file RAIJ_A_2090675_SM0285.zip › Supplementary text and figures/Figure_S48 F260.tif]

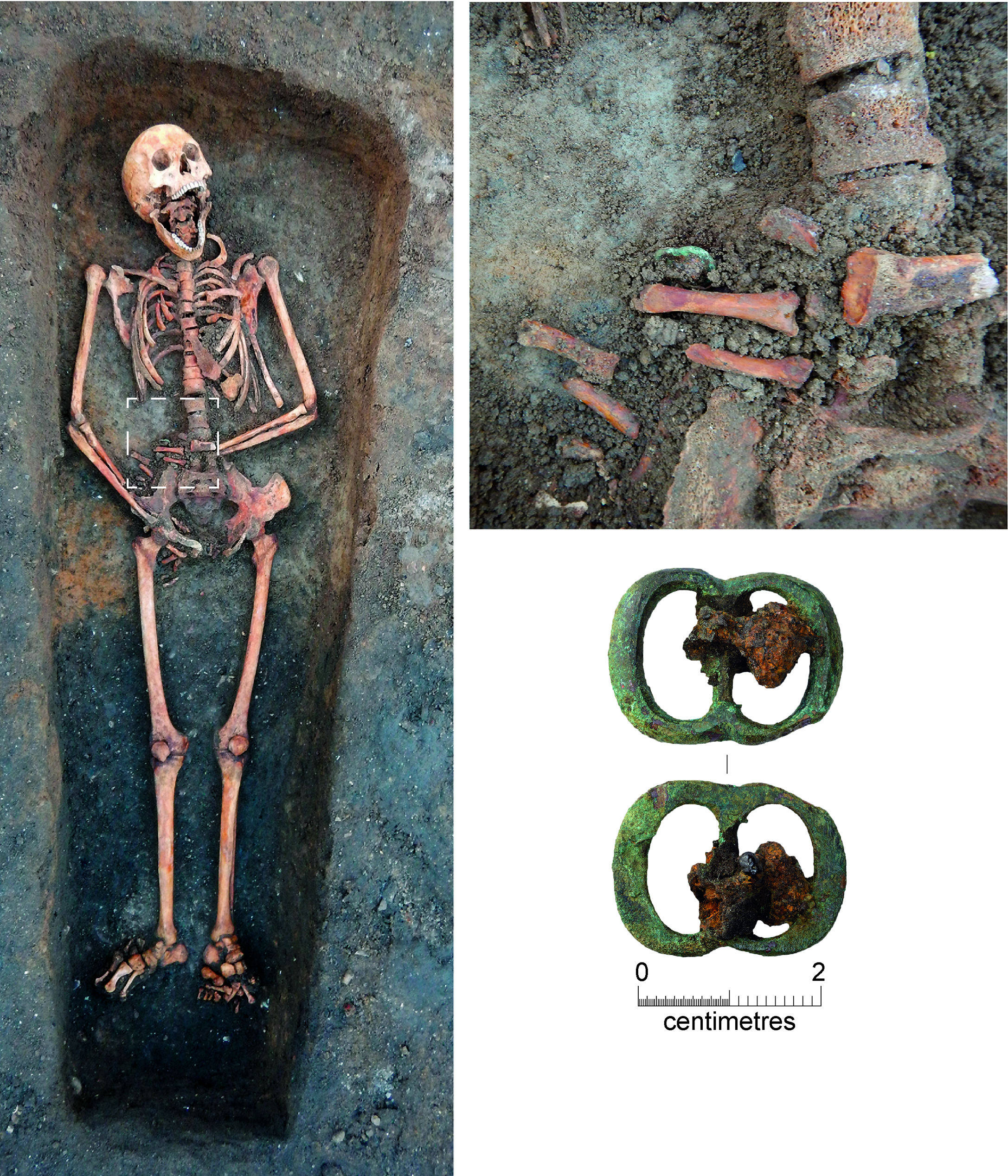

Supplement: Supplemental Material [file RAIJ_A_2090675_SM0285.zip › Supplementary text and figures/Figure_S49 F310.tif]

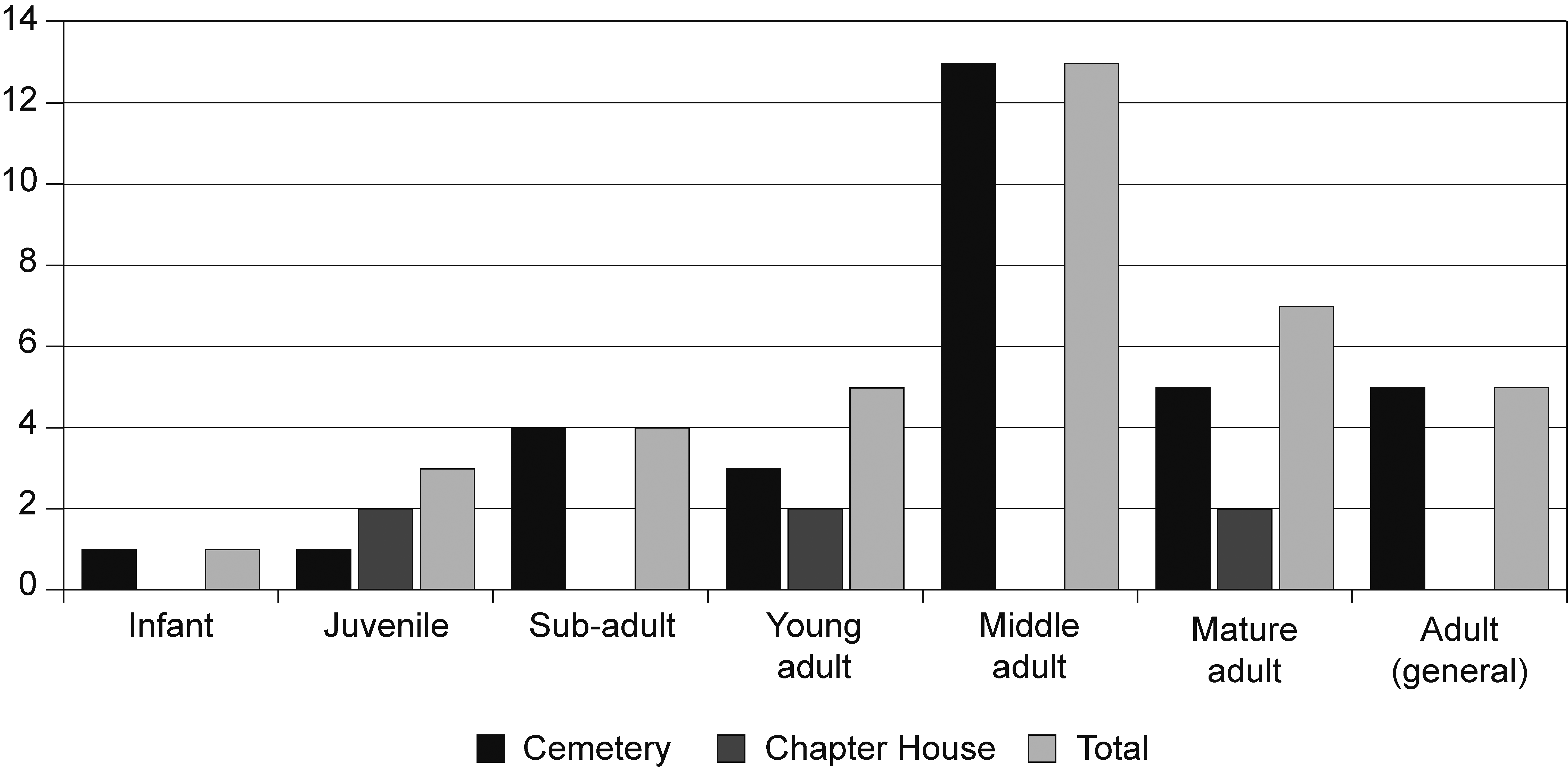

Supplement: Supplemental Material [file RAIJ_A_2090675_SM0285.zip › Supplementary text and figures/Figure_S5 Osteological age graph.tif]

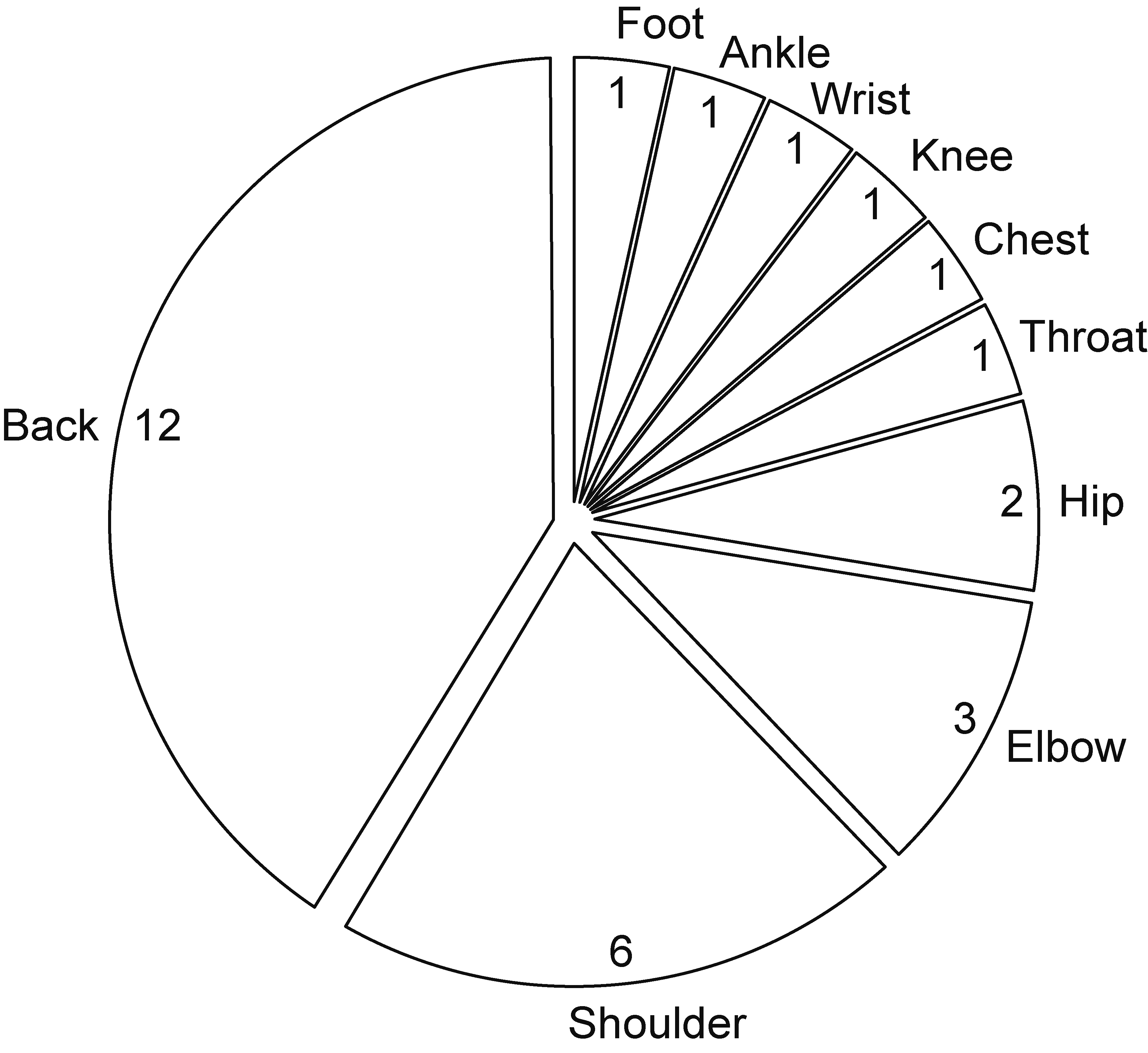

Supplement: Supplemental Material [file RAIJ_A_2090675_SM0285.zip › Supplementary text and figures/Figure_S6 Degeneration chart.tif]

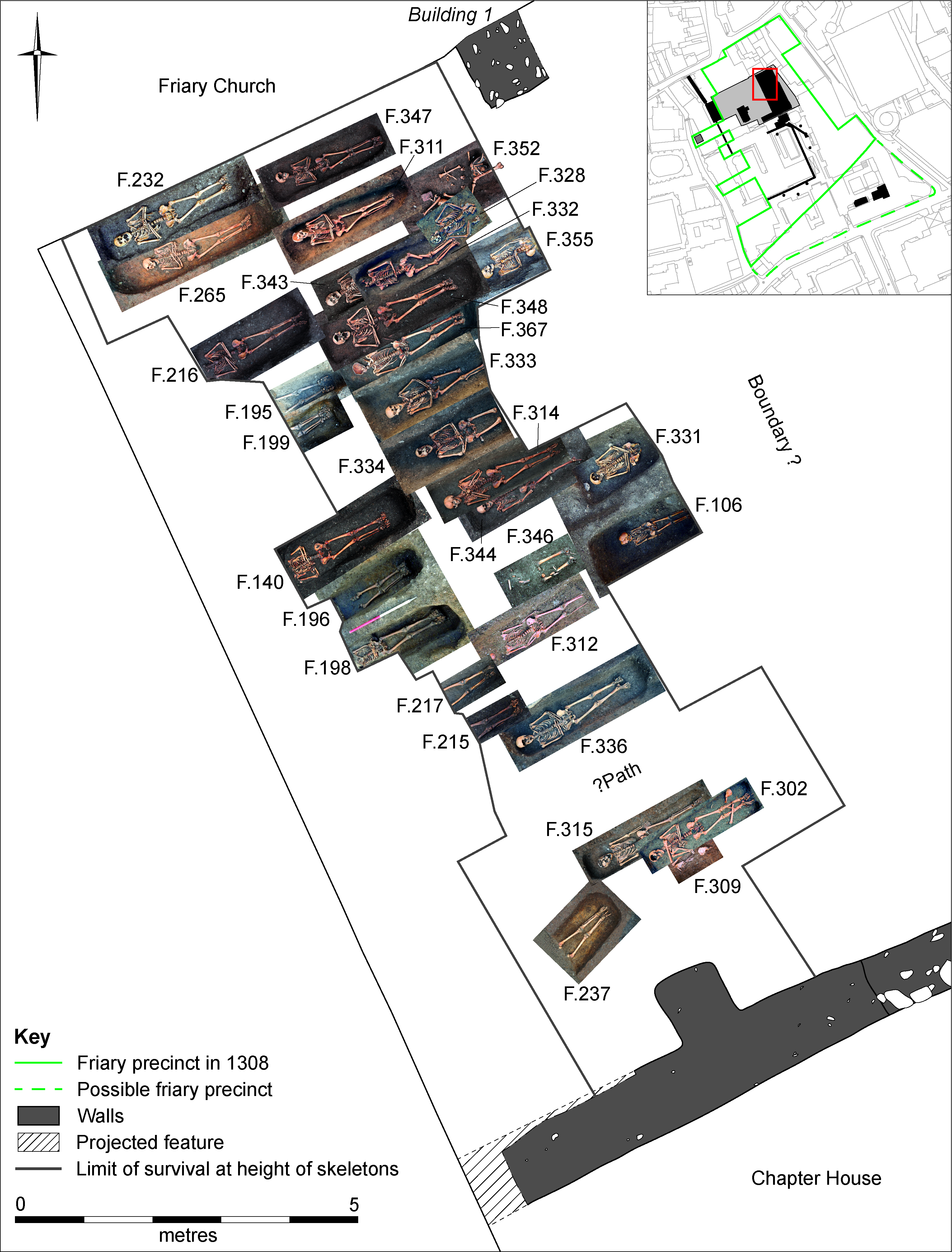

Supplement: Supplemental Material [file RAIJ_A_2090675_SM0285.zip › Supplementary text and figures/Figure_S7 Plan of Cemetery with photos.tif]

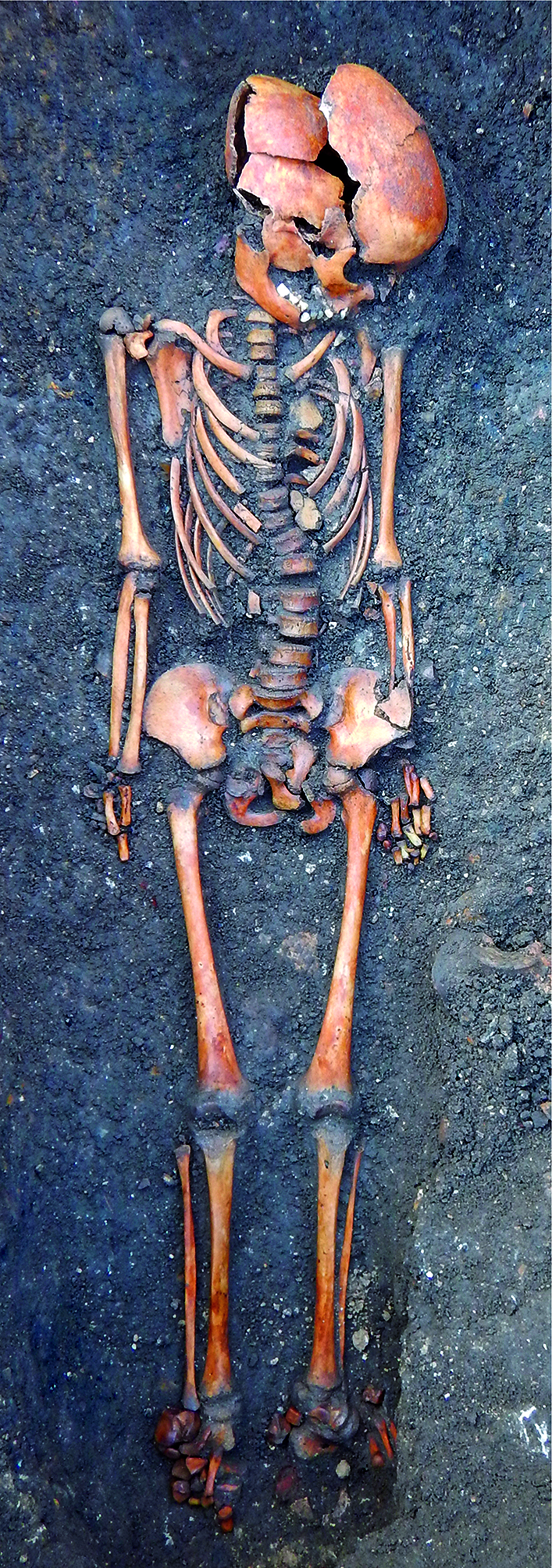

Supplement: Supplemental Material [file RAIJ_A_2090675_SM0285.zip › Supplementary text and figures/Figure_S8 F106.tif]

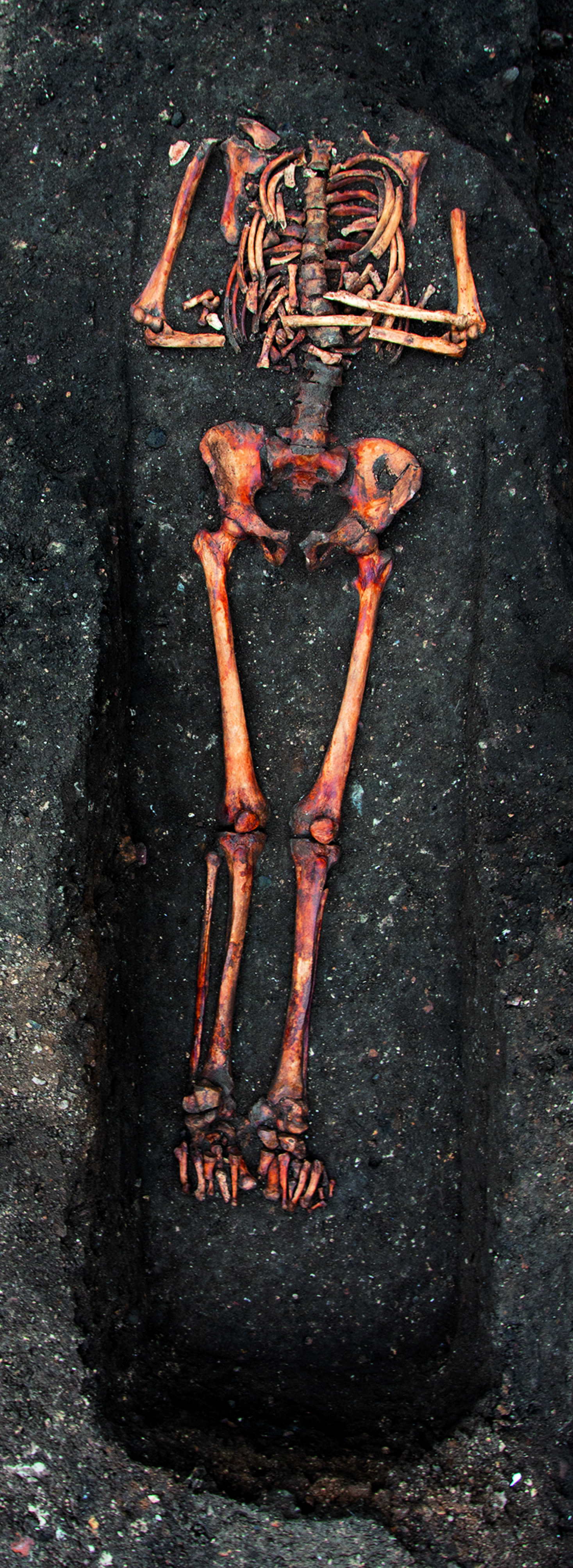

Supplement: Supplemental Material [file RAIJ_A_2090675_SM0285.zip › Supplementary text and figures/Figure_S9 F.140 mod.tif]
